# Predicting phytochemical diversity of medicinal and aromatic plants (MAPs) across eco-climatic zones and elevation in Uttarakhand using Generalized Additive Model

**DOI:** 10.1038/s41598-023-37495-1

**Published:** 2023-07-05

**Authors:** Deepti Tiwari, Pushpa Kewlani, Kailash S. Gaira, Indra D. Bhatt, R. C. Sundriyal, Veena Pande

**Affiliations:** 1 G. B. Pant National Institute of Himalayan Environment, Kosi-Katarmal, Almora, Uttarakhand 263 643 India; 2 G.B. Pant National Institute of Himalayan Environment, Sikkim Regional Centre, Pangthang, Gangtok, Sikkim India; 3 Department of Forestry and Natural Resources, HNB Garhwal University, Srinagar, Garhwal, 249169 Uttarakhand India; 4 Department of Biotechnology, Kumaun University, Nainital, Uttarakhand India

**Keywords:** Plant sciences, Natural variation in plants

## Abstract

The present study uses a systematic approach to explore the phytochemical composition of medicinal plants from Uttarakhand, Western Himalaya. The phytochemical composition of medicinal plants was analyzed based on (i) the presence of different chemical groups and (ii) bioactive compounds. The Generalized Additive Model **(**GAM) analysis was used to predict the occurrence of chemical groups and active compounds across different eco-climatic zones and the elevation in Uttarakhand. A total of 789 medicinal plants represented by 144 taxonomic families were screened to explore the phytochemical diversity of the medicinal plants of Uttarakhand. These medicinal plant species are signified in different life forms such as herbs (58.86%), shrubs (18.24%), trees (17.48%), ferns (2.38%), and climbers (2.13%). The probability of occurrence of the chemical groups found in tropical, sub-tropical, and warm temperate eco-climatic zones, whereas active compounds have a high Probability towards alpine, sub-alpine, and cool temperate zones. The GAM predicted that the occurrence of species with active compounds was declining significantly (p < 0.01), while total active compounds increased across elevation (1000 m). While the occurrence of species with the chemical group increased, total chemical groups were indicated to decline with increasing elevation from 1000 m (p < 0.000). The current study is overwhelmed to predict the distribution of phytochemicals in different eco-climatic zones and elevations using secondary information, which offers to discover bioactive compounds of the species occurring in the different eco-climatic habitats of the region and setting the priority of conservation concerns. However, the study encourages the various commercial sectors, such as pharmaceutical, nutraceutical, chemical, food, and cosmetics, to utilize unexplored species. In addition, the study suggests that prioritizing eco-climatic zones and elevation based on phytochemical diversity should be a factor of concern in the Himalayan region, especially under the climate change scenario.

## Introduction

The growing consumer interest in natural products is gaining popularity all across the globe. The use of these products in pharmaceuticals, cosmetics industries, food, and dietary supplements and their significant biological effect on the human body is widely acknowledged. It has been predicted that the global plant extract market will reach USD 59.4 billion by 2025^1^. Among others, the medicinal and aromatic plants (MAPs) are considered good healers and therapeutic agents utilized from ancient times in various common and severe disorders^2,3^. For instance, the *De Materia Medica* written by a Greek physician and pharmacologist was the first extensively documented pharmacopeia, containing information about 600 plants and 53 prescriptions^4^. Similarly, Ayush (Ayurveda, Unani, Siddha, and Homeopathy) in India is a well-established traditional Indian healthcare system that recognizes medicinal plants for various therapeutic activities. In Ayurveda, approximately 2000 medicinal plant species, Siddha (1121 species), Unani (751 species), and Homeopathy (422 species), are documented for different medicinal formulations and drug development^5^.

The therapeutic and healing activities of the MAPs are due to the presence of biologically active compounds known as phytochemicals^6^. These chemical constituents are produced and stored in plants as secondary metabolites, which exhibit physiological effects on organisms and have been extensively investigated for their therapeutic activities across the world^7,8^. These secondary metabolites naturally occur in plants in different parts such as roots, leaves, stems, flowers, and bark, which defend against pathogen attack, abiotic stress and UV radiation without affecting normal growth, development, and reproduction^9^. The secondary metabolites can be classified into different natural compounds based on their definite chemical structure (containing sugar or rings), composition (nitrogen-containing or not), and biosynthetic pathways. The major classification of secondary metabolites includes three major groups: nitrogen-containing compounds, phenolic compounds, and terpenoids^10–12^. The classification of bioactive compounds in different classes and subclasses is provided in Supplementary Fig. 1. These compounds are obtained from natural resources by various extraction methods, which involve the separation of bioactive compounds from their origin. The common extraction techniques include soxhlet extraction, hydrodistillation, solvent extraction, supercritical fluid extraction (SFE), microwave-assisted extraction (MAE), ultrasound-assisted extraction (UAE), pulsed electrical field extraction (PEF), enzyme-assisted extraction (EAE), accelerated solvent extraction (ASE) and high hydrostatic pressure extraction (HHP)^13^. The obtained extracts usually occur in a combination of various types of compounds. Therefore, the isolation, identification and characterization of pure compounds can be obtained by various chromatographic techniques, namely, column chromatography (CC), thin layer chromatography (TLC), high performance- thin layer chromatography (HP-TLC), high-performance liquid chromatography (HPLC), flash chromatography, and sephadex chromatography. Besides, several non-chromatographic techniques such as phytochemical screening, fourier-transform infrared spectroscopy (FTIR) and immunoassay are also used to separate the desired compounds from the mixture^14^. The isolated, purified compounds are further characterized for structural elucidation using ultraviolet (UV), mass, nuclear magnetic resonance (NMR) and fourier transform infrared (FTIR). Finally, the pharmacological activities of purified compounds lead to the discovery and development of novel drugs from plants^14,15^. The therapeutic potency of such bioactive compounds is reported for diverse pharmacological activities such as antiulcer, anti-inflammatory, antioxidant, cytotoxic, antitumor, antispasmodic, and antidepressant activities^16–19^. Currently, various drugs are available in the market which has been derived from plant-based secondary metabolites known to possess significant pharmacological activities^20–38^.

Uttarakhand is known for its diverse and rich floristic wealth, cultural heritage, and herbal resources. The significant elevation variations and wide range of climatic zones favor the ample growth of diversified and rich vegetation. The state harbors 964 species of medicinal plants from 158 families, including trees (160), shrubs (190), and herbs (614), and they are used to cure about 135 ailments^39^. Several studies on Uttarakhand's MAPs are available to evaluate their bioactive compounds and phytochemical composition^40–42^. However, the potential sequence of the medicinal plants for the presence of secondary metabolites or bioactive compounds and their occurrence within different eco-climatic zones has not yet been studied. In view of the above, the present study is focused on reviewing the medicinal plants of the Uttarakhand state of western Himalaya and their phytochemical diversity within eco-climatic regions and across the elevation.

The objectives of this study are to (i) estimate the occurrence of the different active compounds and chemical groups in MAPs of Uttarakhand, and (ii) predict the occurrence of phytochemical diversity in the MAPs across the eco-climatic and elevation zones.

## Methods

### Data collection and compilation

In the preliminary phase of this study, we compiled a comprehensive list of more than 900 MAPs of Uttarakhand state, India. For this, popular search engines, namely, Google Scholar, PubMed, Science Direct, SpringerLink, and Scopus, were used to retrieve information in research articles published in journals, conference papers, books, and scientific reports of regional, national, and international organizations. A detailed species inventory was prepared and arranged as per family alphabetically (Supplementary Table 1). The medicinal plant species within the different taxonomic families are also arranged alphabetically. The multiple synonyms of the species and correct scientific names were manually checked and confirmed through online databases, i.e. Tropicos (http://www.tropicos.org/), Annotated checklist of flowering plants of Nepal (http://www.efloras.org/) and book Flowering Plants of Uttarakhand: A Checklist^43^. After compiling a comprehensive list of more than 900 MAPs, we gathered information on the phytochemical composition of each plant. We manually checked published research articles using keywords such as 'phytochemical component', 'major active compounds', 'principle chemical constituents', 'phytochemical analysis,' and ‘secondary metabolites’ to explore relevant articles. We gathered information on the phytochemical composition of 789 medicinal plants (Supplementary Table 1).

### Data analysis

#### Phytochemical composition of MAPs

The analysis was performed in two ways: (i) distribution of phytochemicals as a specific chemical class (flavonoids, alkaloids, terpenoids, and phenolic acids), and (ii) distribution of bioactive compounds such as quercetin, kaempferol, caryophyllene, gallic acid, chlorogenic acid, catechin across the MAPs. The chemical classification of bioactive compounds was determined using ClassyFire (http://classyfire.wishartlab.com/) to explore their chemistry (Supplementary Fig. 1). ClassyFire provides a hierarchical classification of compounds into the kingdom (organic or inorganic), followed by superclasses, classes, and subclasses. Superclasses include 26 organic and 5 inorganic categories, such as organic acids and derivatives, organometallic compounds, homogeneous metal compounds, phenylpropanoids, and polyketides. Classes include more specific and recognizable features of compounds such as flavonols, actinide salts, pyrimidine nucleosides, and benzazepines. The Sub-classes consist of > 10,000 known compounds, and there are 1729 known sub-classes in the current phytochemical taxonomy.

#### Generalized Additive Model (GAM): predicting the occurrence of the MAPs

The information on the active compound and chemical groups was gathered for individual species. Simultaneously, each species were arranged to their elevation ranges and categorized in different eco-climatic zones, i.e., tropical (< 500 m asl); sub-tropical (500–1500 m asl); warm temperate (1500–2500 m asl); cool temperate (2500–3000 m asl); sub-alpine (3000–3500 m asl) and alpine (> 3500 m asl)^44^. The StatSoft STATISTICA (8.0.360) software was used for data analysis. The occurrence of the species with and without chemical compounds and the chemical group were estimated within eco-climatic zones, and the significance of the probability of the occurrence was tested using Z-test. Also, to predict the occurrence of the chemical compound and chemical group along the elevation, we considered the medium of the range of the species elevation data for the analysis as an explanatory variable. In contrast, the total occurrence of species across active compounds and chemical groups and the total numbers of compound and chemical groups were considered the dependent or response variable. The response variable assumes as non-normal and non-linear. To deal with nonparametric and non-linear statistical analysis, the Generalized Additive Model (GAM) was considered best suited especially for secondary database analysis^45^ (Supplementary Fig. 2). The GAM is considered flexible in two significant respects such as (i) the distribution of the response variable can be non-normal (explicitly), and (ii) the response variable values are predicted from a linear combination of predictor variables, which are connected to the response variable via a link function (e.g., logarithmic, identity). The total occurrence of bioactive compounds and chemical groups was considered the dependent variable, which was linearly associated with values on the '*X'* explanatory variables (e.g., elevation). The GAM is a semi-parametric extension of GLM^46^ and deals with highly non-linear and non-normal relationships between the response and the set of explanatory variables^45^. GAM includes the estimation of smoothing terms in the additive model and general algorithm added in the model as partial residuals (i.e. ${j}^{th}$ set of partial residuals).

$$R_{j} = Y - s_{0} - \sum\limits_{k \ne j} {s_{k} \;(X_{k} )}$$

The partial residuals remove the effects of all the other variables from *Y* (depending on the variable, i.e. occurrence of species with active compounds and groups and total active compounds and groups), therefore, the *Y* can be used to model the effects against *X*_*j*_ (elevation).

## Results

### Diversity of medicinal plants across taxonomic families and habits

In the present study, 789 medicinal plants belonging to 144 taxonomic families were reported for phytochemical diversity widely distributed in different regions of Uttarakhand state (Supplementary Table 1). Among 144 taxonomic families, Asteraceae represented the maximum number (64) of medicinal plants, followed by Lamiaceae (59), Fabaceae (54), and Rosaceae(34) (Supplementary Fig. 3A). Distribution pattern of the medicinal plants in different life forms as herbs (58.86%) > shrubs (18.24%) > trees (17.48%) > ferns (2.38%) > climbers (2.13%) > others (0.88%) is depicted in Supplementary Fig. 3B.

### Distribution of phytochemicals in medicinal plants

**Figure 1:**
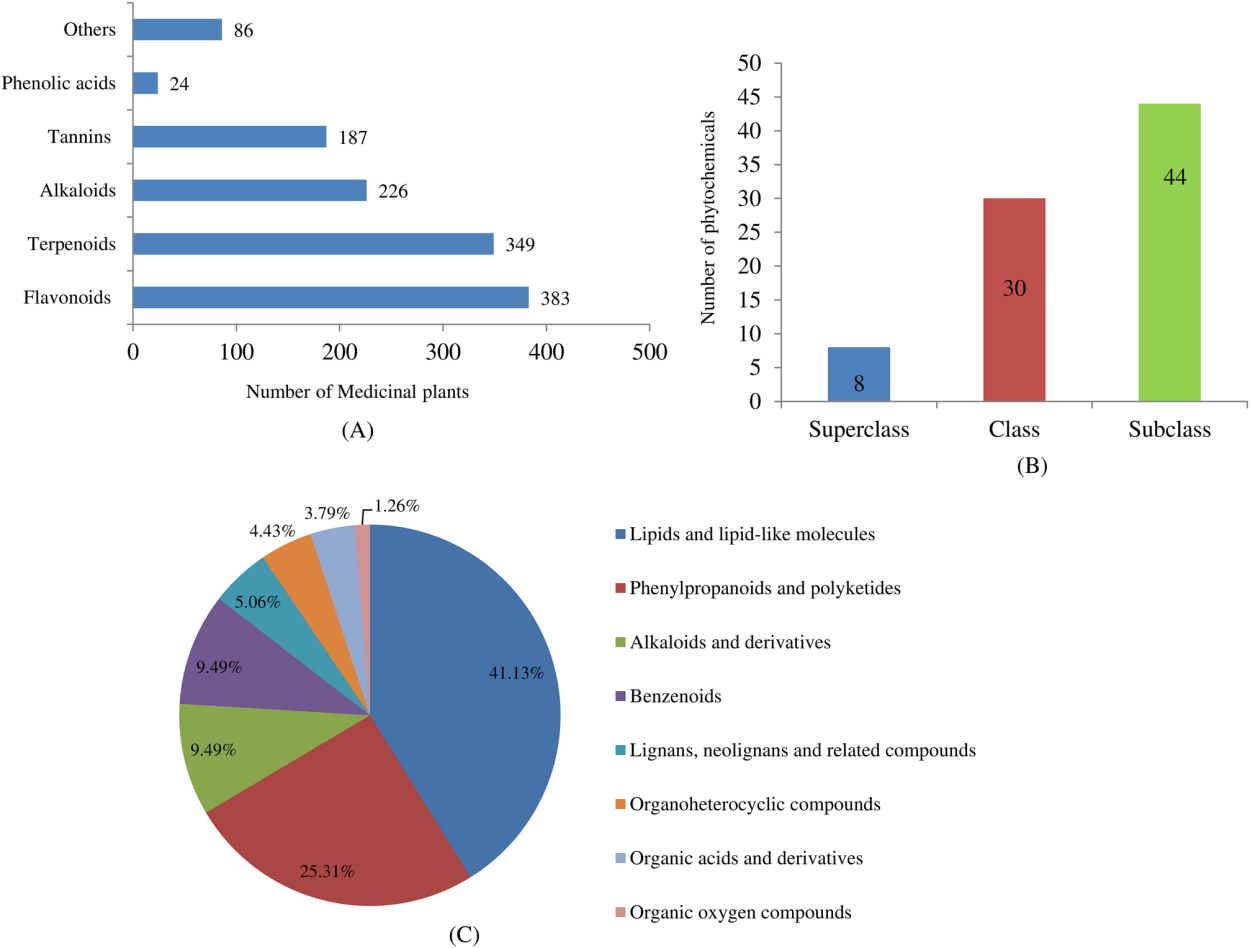
Distribution of phytochemicals in medicinal plants across (**A**) specific classes of phytochemicals (**B**) superclass, class and subclass (**C**) superclass.

Considering the specific classes of phytochemicals, phenolic compounds emerged as a highly investigated group in 789 plants. Among them, the flavonoid is a highly abundant class of phytochemicals represented by 383 medicinal plants followed by terpenoids (349), alkaloids (226), tannins (187), phenolic acids (24) and others such as lignans, stilbenes and anthraquinones (86) (Fig. 1A). For analysis of the major bioactive compounds ClassyFire an online database was used to collect information of active compounds (Supplementary Fig. 2). A total of 207 bioactive compounds associated with 789 medicinal plants distributed across 8 superclasses, 30 classes and 44 subclasses (Fig. 1B). Among the 8 superclasses, lipids and lipid-like molecules were highly abundant (41.13%) followed by phenylpropanoids and polyketides (25.31%), alkaloids and derivatives, and benzenoids (9.49% each) (Fig. 1C).

**Figure 2:**
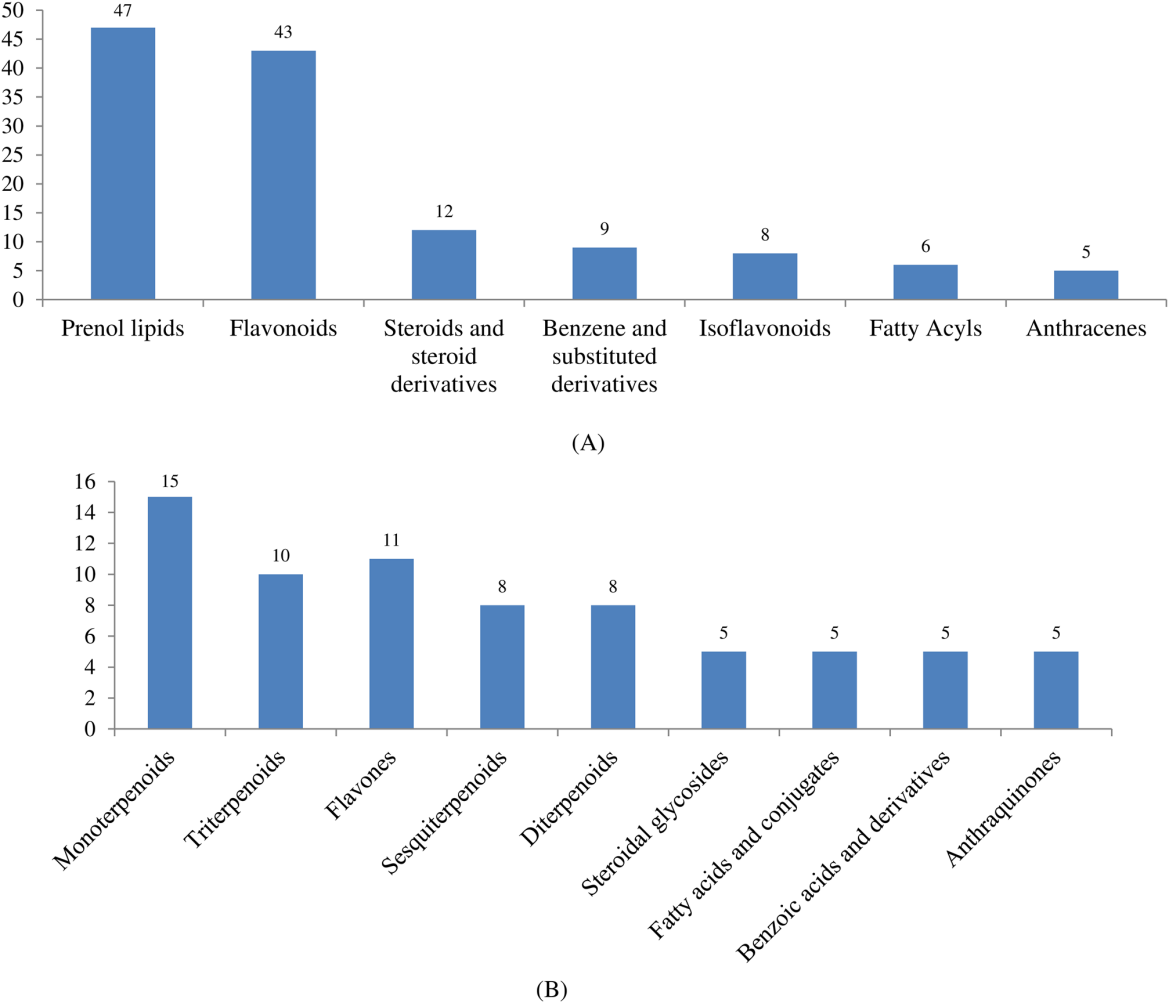
Distribution of phytochemicals across (**A**) chemical class (**B**) subclass.

The distribution of phytochemicals in 30 chemical classes shows that prenol lipids contain the highest numbers (47), followed by flavonoids and steroids (43), steroid derivatives (12) and other chemicals classes represent less than 10 (Fig. 2A). The distribution of phytochemicals across the subclasses shows the presence of monoterpenoids, flavones and triterpenoids with 15, 11 and 10, respectively (Fig. 2B).

### Prediction of the occurrence of different chemical groups and active compounds

The occurrence of the bioactive compounds and chemical groups was calculated and tested. The probability of occurrence of bioactive compounds was observed to be significantly higher in sub-alpine, cool temperate, and alpine zones as compared to population probability, i.e. (p = 0.5235). In contrast, low probability was observed in tropical, warm temperate, and sub-tropical zones (p < 0.01) (Table 1). In view of the chemical group, the probability of the occurrence was indicated as high in tropical, alpine, and sub-tropical as compared to the population probability (p = 0.7415), while low probability was indicated for warm temperate and cool temperate zone significantly (p < 0.002) (Table 1). The insignificance of the occurrence of the chemical group was observed for sub-alpine (p > 0.05) (Table 1). The results revealed a high probability of active compounds to be found in alpine, sub-alpine, and cool temperate zones. At the same time, the chemical group may have more probability to be found in tropical, sub-tropical, and warm temperate eco-climatic zones.

**Table 1:** Test the significance of the probability of species occurrence with chemical compounds and chemical groups in the different eco-climatic zone of Uttarakhand.

Eco-climatic zones	N	Chemical compounds			Chemical groups		
		p	1 − p	Z score (p-value)	P	1 − p	Z score (p-value)
Tropical	33	0.4546	0.5454	− 4.55271 (0.0001)	0.8182	0.1818	5.783988 (0.0001)
Sub-tropical	368	0.5190	0.4810	− 3.26279 (0.01)	0.7636	0.2364	18.61415 (0.0001)
Warm Temperate	213	0.4836	0.5164	− 17.0084 (0.0001)	0.6948	0.3052	− 22.6719 (0.0001)
Cool Temperate	63	0.6032	0.3968	10.05637 (0.00001)	0.7143	0.2857	− 3.90715 (0.0001)
Sub-alpine	44	0.6364	0.3636	9.947346 (0.00001)	0.7273	0.2727	− 1.42371 (0.07727)
Alpine	68	0.5588	0.4412	4.8161 (0.00001)	0.7647	0.2353	3.613348 (0.0002)
Total	789	0.5234	0.4766		0.7415	0.2585	

*n* number of species, *p* probability of the occurrence of chemical compounds and groups; 1 − p = probability of the non-occurrence of chemical compounds and groups.

**Figure 3:**
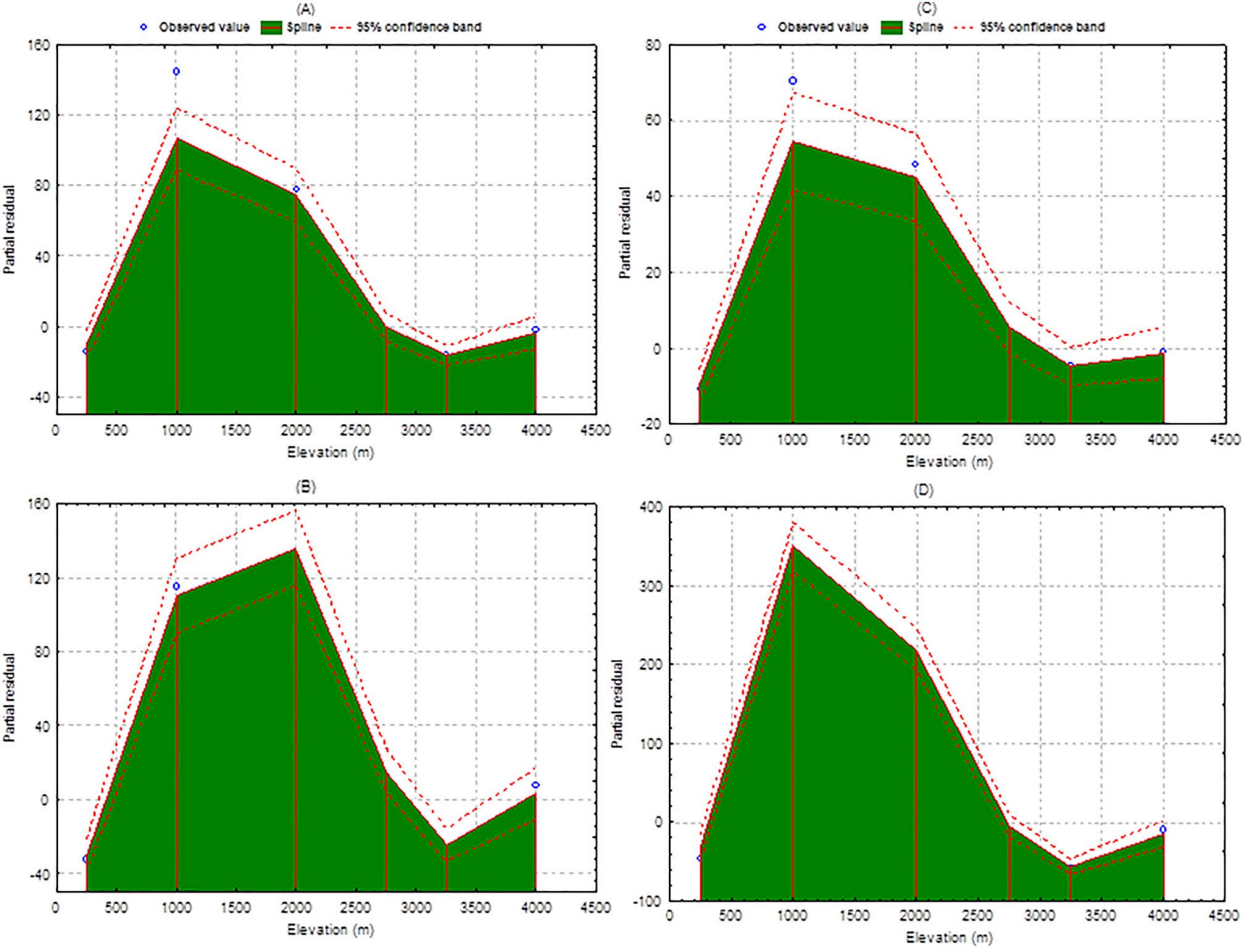
GAM predicted: (**A**) occurrence of species with active compounds, (**B**) total active compounds, (**C**) occurrence of species with chemical groups, and (**D**) total chemical groups along the elevation with spline line and 95% confidence band for elevation (m).

The GAM estimated species occurrence with different chemical groups and bioactive compounds and total bioactive compounds and chemical groups along the elevation. The analysis revealed that 2–6 species were found declining with active compounds across the 1000 m asl (β = − 0.0037; SE = 0.0017; R^2^ = 96.10; p < 0.01), while ~ 4 total bioactive compounds were found increasing as per 1000 m asl (β = 0.00206; SE = 0.00218; R^2^ = 96.13; p < 0.01). In contrast, the ~ 2 species with chemical groups found to increase per 1000 m elevation (β = 0.0013; SE = 0.00107; R^2^ = 97.08; p < 0.01) while 1–2 chemical groups found less as per the 1000 m elevation (β = − 0.0156; SE = 0.0031; R^2^ = 94.81; p < 0.01) (Fig. 3). The results indicate that declining the occurrence of species across the elevation producing more chemical compounds while increasing the occurrence of species across the elevation representing less chemical groups.

## Discussion

Using medicinal plant diversity as herbal medicine is a promising bridge between affordable health care, economic development, and biodiversity conservation in Uttarakhand^3^. Due to the remoteness, poverty, and lack of modern healthcare facilities, the people of this region strongly believe in this age-old traditional cure system^3,39^. In the context of the pandemic Coronavirus disease (COVID-19), medicinal plants are found to be very effective^47–49^. Many people affected by the COVID-19 have recovered without hospitalization as they use medicinal plants of their region. For example, *Nigella sativa* contains a bioactive component with strong antiviral activity, which blocks the entry of the virus. Augmentation of Zn supplement with *Nigella sativa* could improve immunity and provide ionophore Zn2^+^ to the host immune system against SARS-CoV-2 by inhibiting the replication^50^. The crude extract or purified compounds of several other herbs, namely A*rtemisia annua*, *Astragalus membranaceus*, *Cassia alata*, *Tinospora cordifolia, Tribulus terrestris*, *Cullen corylifolium*, and *Paulownia tomentosa* showed promising preventive activity against SARS-CoV-2 infection^51,52^. The global forecast indicated that the medicinal plant extract would be used as a food, health, and wellness supplement for curing COVID-19. The botanical extracts market is expected to reach $10 billion by 2028 at a CAGR of 9.3%^53^. These estimates validated the importance of medicinal plants and their extracts for maintaining the healthcare of human beings.

The therapeutic activity of medicinal plants often relies on the presence of phytochemical compounds or secondary metabolites. These naturally occur in medicinal plants and are considered good free radical scavengers with multiple biological activities^54–60^. For example, rosmarinic acid, an important phenolic compound found majorly in Boraginaceae and Lamiaceae family, is reported to exert a broad spectrum of biological activities such as antiviral, antibacterial, anti-inflammatory, astringent, and antioxidant^61,62^. Similarly, curcumin, the main active constituent derived from *Curcuma longa* and *Rheum ribes* has emerged as a new source of cucumin, which possesses antimicrobial, anticancer, antioxidant, neuroprotective, anti-inflammatory, and antidiabetic activities^63,64^. In the present review, the presence of different phytochemical groups and > 200 specific biologically active compounds showed the potential of medicinal plants in different commercial sectors such as pharmaceutical, nutraceutical, chemical, food, and cosmetic. Examples of such commercially important phytochemicals groups are alkaloids, flavonoids, phenolics, steroids, saponins, and more specific bioactive compounds such as resveratrol, quercetin, rutin, myricetin, quercetin, kaempferol, luteolin, apigenin, gallic acid, ellagic acid, and taxol^65–71^. Despite this, many species remain unexplored for detailed phytochemical investigation. Out of 900 medicinal plants reported from the region, the information on phytochemical diversity was retrieved only in 789 species.

The GAM estimated a declining rate of the occurrence of species with active compounds (2–6 species) and an increasing rate of the total chemical compound across the elevation (per 1000 m), which indicates that active chemical compounds are predominant more in high elevation species. The occurrence of species with chemical groups was found increasing number while total chemical groups were represented declining with increasing elevation. The results indicate that declining species occurrence across the elevation produces more bioactive compounds while increasing species across the elevation represents fewer chemical groups. Interesting inferences indicated for active compounds which projected high elevation species have higher compounds because they generate high tolerance compounds against sensitive and extreme climatic conditions. A recent review also suggests that the low-elevation habitats are stable and productive which creates higher biotic pressure among species, favoring higher phytochemical diversity. On the contrary, high-elevation habitats have less competition and hostile interactions, but habitat heterogeneity is much larger, leading to declining phytochemical diversity but selecting specific molecules essential for survival in stressful, sensitive and extreme climatic conditions^72^. In recent decades, the high-elevation region has been characterized by unfavorable climatic conditions such as temperature fluctuation, salinity, high UV radiation, high wind velocity, O_2_ deficiency, and low nutrient supply^73^. These changing climatic conditions influence the physiological, morphological, molecular and biochemical responses of plants^73^. The plant species of high-elevation areas have adapted different mechanisms for synthesizing secondary metabolites that make them survive under such adverse climatic conditions. This could also be a major reason for accumulating diverse and specific chemical compounds^74^.

Various environmental factors such as elevation, temperature, water and nutrient availability, soil characteristics, O_2_ and CO_2_ level, salinity, pollutants and radiation (light, ultra-violet and ionization radiation) are known to influence the composition and concentration of bioactive compounds of plants^75–77^. In the present scenario, climate change is a global issue characterized by alteration in various environmental variables such as temperature, atmospheric CO_2_ concentration and elevated ultraviolet B (UV-B) radiation^78^. The Inter-governmental Panel on Climate Change (IPCC) estimated that the mean temperature is increasing more rapidly to the rate of 0.06–0.1 °C per year with an increase in CO_2_ level of 407.4 ppm per year globally^79^. The IPCC predicted the rise of 4.2 °C average temperature worldwide by the end of the twenty-first century. Such changes in climatic conditions adversely affect the growth, morphology, productivity, physiology, and ultimately, production of secondary metabolites in medicinal plants. Some of the environmental factors affecting the biosynthesis and accumulation of secondary metabolites in plants are described below.

### Elevation

Among various environmental factors, elevation is important in plant metabolites^80–82^. As such, environmental conditions influenced by altitude have proven to be an important factor inducing variations in the secondary metabolite composition of hemp, indicating that plants grown at different elevations exhibited a variation in terpenes^83^. The secondary metabolite production of turmeric (curcumin, R^2^ = 0.2236) and ginger (10-gingerol, R^2^ 0.6979) increased with increasing altitude^84^. The impact of varying elevation on five medicinal plants (*Artemisia judaica*, *Achillea fragrantissima*, *Teucrium polium*, *Lavandula pubescens*, and *Retama raetam*) showed increased accumulation of secondary metabolites at higher elevation^85^. *Zataria multiflora* collected from 14 different populations of Iran showed high variability in the accumulation of major active compounds such as carvacrol, thymol, and linalool among the investigated populations^86^. The concentration of major active compounds (crocin, picrocrocin, and safranal) of *Crocus sativus* increased at high elevation population^87^. In *Coleus forskohlii* collected from 5 different populations of varying altitudes, a significant increase was observed in the phenol, flavonoid, and terpenoid content with the increasing altitude^88^. The variation in the structural and functional attributes of a species growing in diverse conditions is expected.

### Temperature

Temperature is another important environmental factor strongly influencing metabolic activities and the accumulation of secondary metabolites in medicinal plants. Different plant species show variation in their response to low and high temperatures. Many studies suggest that the accumulation of secondary metabolites increases in elevated temperature, but in others, reduced temperature leads to enhanced production of secondary metabolites. The cultivation temperature of *Panax quinquefolius* increased by 5 °C, resulting in increased production of ginsenosides (49%) in roots^89^. In *Salvia miltiorrhiza*, accumulation of tanshinones increased with increasing temperature^90^. The alkaloid content in six cultivars of *Lupinus angustifolius* grown in field conditions and greenhouse at different temperatures (10, 20, and 30 °C) was found to increase with rising temperature^91^. In *Daucus carota,* root determination of fifteen terpenes showed increasing values with an increase in temperature except for one terpene (α-terpinolene), which significantly decreased with an increase in temperature^92^. Artemisinin content in *Artemisia annua* is increased when exposed to a transient pre-chilling treatment^93^. In *Glycine max* roots, the level of phenolic acids and isoflavonoids (genistein, daidzein, and genistin) increased after exposure to low temperature for 24 h^94^. In *Camellia japonica,* the concentration of unsaturated fatty acid (α-linolenic acid) and jasmonic acid are believed to be involved in the cold acclimation process in response to low temperature^95^.

### Radiation

The light quality (intensity, range, and duration) influences medicinal plants growth and metabolite production because each plant requires appropriate photoperiod and intensity of light for secondary metabolite synthesis. The light quality adversely affects the plant at the cellular level and ultimately leads to the death of the plant. The callus cultures of *Rhodiola imbricata* exposed to different light conditions: 100% red, 100% blue, 100% green, 40% red: 40% Green: 20% blue and 100% white showed that the callus culture grew under blue light accumulated maximum amount of salidroside, total phenolics and total flavonoids^96^. The shade-developed leaves of *Erigeron breviscapus* transferred to full sunlight exhibited a significantly higher concentration of flavone glycoside (scutellarin)^97^. In different populations of *Centella asiatica*, light exposure affected the content of terpenes, and 70% of shade-grown plants contained the highest asiatic acid and minimum asiaticoside content^98^. The *C. asiatica* grown in full daylight exposure exhibited more asiaticoside, madecassoside, chlorogenic acid, and flavonoids compared to 50% shade-grown plants^99^. In the plant of *Solanum lycopersicum* exposed to high-frequency radiation, a significant decline in total phenolics, flavonoids, vitamin C, and antioxidant activities was observed. In addition, increased synthesis of lycopene content indicated a harmful effect on fruit skin and release of increased lycopene due to softening of fruit skin^100^.

Ultraviolet B (UV-B) radiation is considered a potential elicitor for the biosynthesis of secondary metabolites in plants. Suspension cultures of *Catharanthus roseus* were irradiated with UV-B for 5 min and showed the increased synthesis of secondary metabolites catharanthine and vindoline^101^. The effect of UV radiation over the other environmental variable showed a higher accumulation of cynaroside in *Capsicum annuum*, whereas higher graveobioside A contents were found under salt stress conditions^102^. In *Glycine max*, the concentration of total flavonoids, rutin and quercetin increased under the stress of enhanced UV radiation (1.73 kJ h^−1^ m^−2^), elevated O_3_ (110 ± 10 h mol mol^−1^) and combined stress (UV + O_3_) at flowering and podding stage. In contrast, at the branching stage, the content of total flavonoids, rutin and quercetin decreased under the three treatments^103^. UV-B-induced accumulation of total flavonoid, quercetin, kaempferol, and isorhamnetin was also reported in *Ginkgo biloba* leaves^104^. Causes of such variations in the phytochemicals in varying environmental conditions generally depend on to genetic attributes of the plant^105^, altitude^106^, season/developmental stages^107^, harvesting time and micro-climatic conditions^108^ of the growing location of a plant. However, it is not definite to ascertain a particular factor responsible for such variations^80^.

## Conclusion

Uttarakhand is a reservoir of medicinal plants and has been validated by the presence of 964 medicinally important species, which are effectively used to cure several ailments in various parts of the state. However, the full potential is yet to be harnessed. The presence of secondary metabolites (phenolics, alkaloids, terpenoids) and more than 200 bioactive constituents in the medicinal plants which are distributed across varied eco-climatic zones and elevations, indicate much scope to explore more phytochemicals responsible for diverse therapeutic activities. The GAM analysis in the present review highlighted that fewer species represented the presence of active compounds and chemical groups, and these species need to be further promoted for conservation. The result indicates that the high-elevation areas are a rich source of medicinal plants with diverse and unique chemical constituents, but detailed chemical profiling of many species remains unexplored. This study further extended to investigate the more phytochemical composition of unexplored medicinal plants, providing a new base for research and development. Moreover, the response of medicinal plants acclimation towards the changing climatic conditions needs to be addressed.

## Management practices and future aspect

1. Identify promising populations in different altitudinal zones for harnessing the use of medicinally and economically significant secondary metabolites.
2. Develope agro-techniques for large scale cultivation of high-value medicinal plants.
3. Conserve sensitive and medicinal plants rich habitats degraded by over-harvesting, and other natural pressures.
4. Record long-term ecological observations (seasonal, biannual and annual) of medicinal plants in the changing environmental scenario. Such studies are highly recommended for high-elevation medicinal plants, which have proven to be an active source of the most economically important secondary metabolites. The high elevation zones are primarily affected by varying climatic conditions due to reduced oxygen levels, higher light intensity, low temperature, nutrient deficiency, light intensity and UV-radiation.
5. Measure the impact of different environmental factors on secondary metabolite for identifying favourable habitats of medicinal plants in Uttarakhand.
6. Promote coordination among botanists, ecologists, environmentalists, biochemists, and relevant institutions dedicated to biodiversity conservation for developing sustainable strategies for conservation and utilization MAPs resources.
7. There is an urgent need to validate the indigenous uses of Himalayan medicinal plants through in-depth screening and chemical characterization and identify candidate species for developing novel drugs.

## Supplementary Information

Supplementary Figure 1.

Supplementary Figure 2.

Supplementary Figure 3.

Supplementary Table 1.

## Data Availability

This article and its supplementary information files include all data generated or analyzed during this study.
