# Supplementary material for: Predicting phytochemical diversity of medicinal and aromatic plants (MAPs) across eco-climatic zones and elevation in Uttarakhand using Generalized Additive Model
Source: Sci Rep. 2023 Jul 5;13:10888. doi: 10.1038/s41598-023-37495-1 (PMC10322824; doi:10.1038/s41598-023-37495-1)
Supplement: Supplementary file 1 — Supplementary Figure 1. [file 41598_2023_37495_MOESM1_ESM.pptx]

## Slide 1
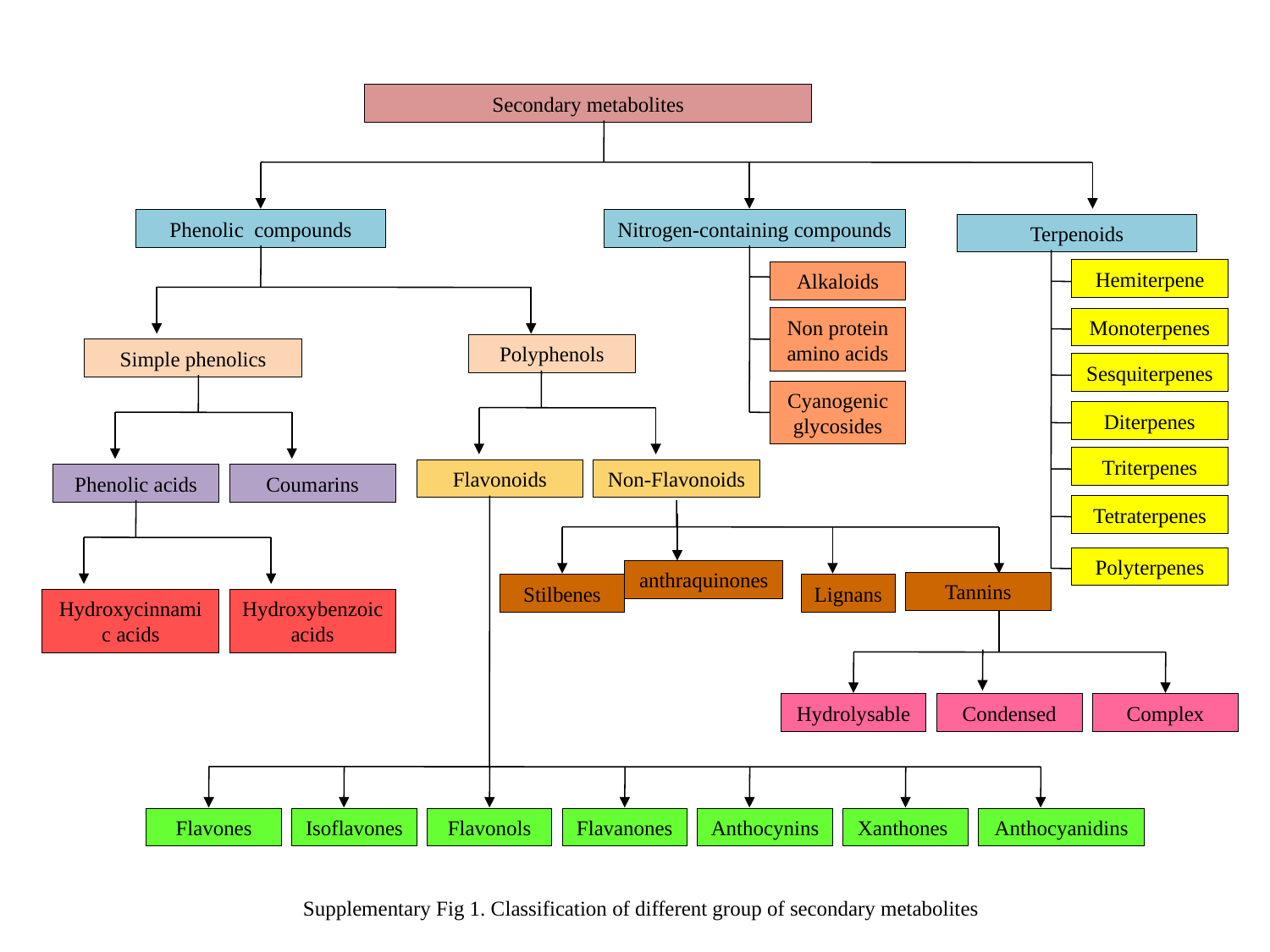

Secondary metabolites
Phenolic compounds
Nitrogen-containing compounds
Terpenoids
Hemiterpene
Alkaloids
Non protein amino acids
Monoterpenes
Polyphenols
Simple phenolics
Sesquiterpenes
Cyanogenic glycosides
Diterpenes
Triterpenes
Flavonoids
Non-Flavonoids
Phenolic acids
Coumarins
Tetraterpenes
Polyterpenes
Tannins
Lignans
Stilbenes
Hydroxybenzoic acids
Hydroxycinnamic acids
Hydrolysable
Condensed
Complex
Flavones
Isoflavones
Flavonols
Flavanones
Xanthones
Anthocynins
Anthocyanidins
anthraquinones
Supplementary Fig 1. Classification of different group of secondary metabolites
