# Supplementary material for: Predicting phytochemical diversity of medicinal and aromatic plants (MAPs) across eco-climatic zones and elevation in Uttarakhand using Generalized Additive Model
Source: Sci Rep. 2023 Jul 5;13:10888. doi: 10.1038/s41598-023-37495-1 (PMC10322824; doi:10.1038/s41598-023-37495-1)
Supplement: Supplementary file 2 — Supplementary Figure 2. [file 41598_2023_37495_MOESM2_ESM.pptx]

## Slide 1
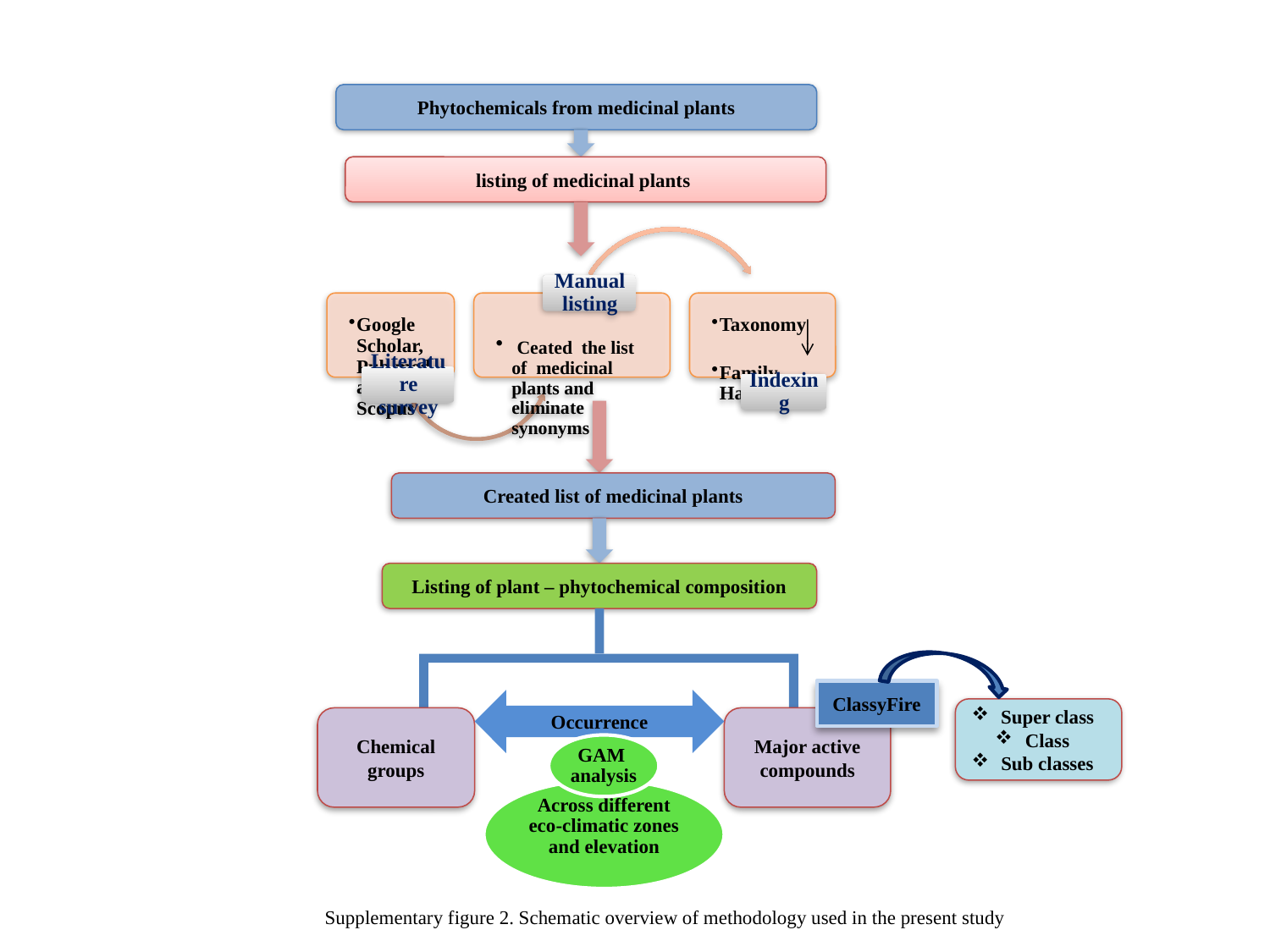

Phytochemicals from medicinal plants
listing of medicinal plants
Created list of medicinal plants
Listing of plant – phytochemical composition
ClassyFire
Occurrence
Super class
Class
Sub classes
Chemical groups
Major active compounds
GAM analysis
Across different eco-climatic zones and elevation
Supplementary figure 2. Schematic overview of methodology used in the present study
