# Supplementary material for: Predicting phytochemical diversity of medicinal and aromatic plants (MAPs) across eco-climatic zones and elevation in Uttarakhand using Generalized Additive Model
Source: Sci Rep. 2023 Jul 5;13:10888. doi: 10.1038/s41598-023-37495-1 (PMC10322824; doi:10.1038/s41598-023-37495-1)
Supplement: Supplementary file 3 — Supplementary Figure 3. [file 41598_2023_37495_MOESM3_ESM.pptx]

## Slide 1
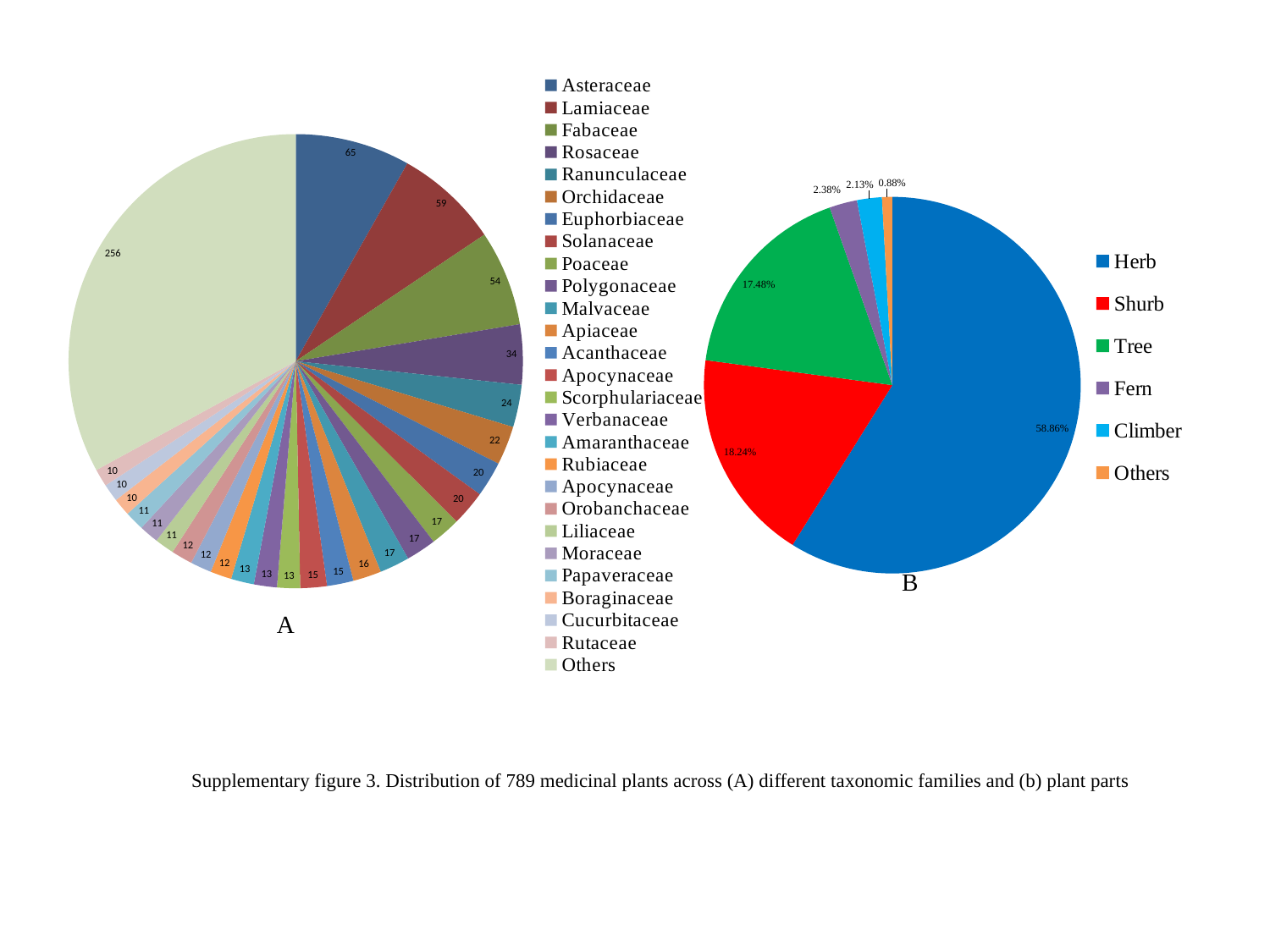

### Chart
| Category | |
|---|---|
| Asteraceae | 65.0 |
| Lamiaceae | 59.0 |
| Fabaceae | 54.0 |
| Rosaceae | 34.0 |
| Ranunculaceae | 24.0 |
| Orchidaceae | 22.0 |
| Euphorbiaceae | 20.0 |
| Solanaceae | 20.0 |
| Poaceae | 17.0 |
| Polygonaceae | 17.0 |
| Malvaceae | 17.0 |
| Apiaceae | 16.0 |
| Acanthaceae | 15.0 |
| Apocynaceae | 15.0 |
| Scorphulariaceae | 13.0 |
| Verbanaceae | 13.0 |
| Amaranthaceae | 13.0 |
| Rubiaceae | 12.0 |
| Apocynaceae | 12.0 |
| Orobanchaceae | 12.0 |
| Liliaceae | 11.0 |
| Moraceae | 11.0 |
| Papaveraceae | 11.0 |
| Boraginaceae | 10.0 |
| Cucurbitaceae | 10.0 |
| Rutaceae | 10.0 |
| Others | 262.0 |
### Chart
| Category | |
|---|---|
| Herb | 0.5886 |
| Shurb | 0.18238 |
| Tree | 0.1748 |
| Fern | 0.0238 |
| Climber | 0.021300000000000006 |
| Others | 0.008800000000000025 |B
A
Supplementary figure 3. Distribution of 789 medicinal plants across (A) different taxonomic families and (b) plant parts
