# Supplementary material for: Predicting phytochemical diversity of medicinal and aromatic plants (MAPs) across eco-climatic zones and elevation in Uttarakhand using Generalized Additive Model
Source: Sci Rep. 2023 Jul 5;13:10888. doi: 10.1038/s41598-023-37495-1 (PMC10322824; doi:10.1038/s41598-023-37495-1)
Supplement: Supplementary file 4 — Supplementary Table 1. [file 41598_2023_37495_MOESM4_ESM.docx]

**Supplimentary Table 1 : Diversity of Medicinal plants having phytochemical association**

| **S. N.** | **Scientific Name** | **Habit** | **Active compounds** | **References** |
| --- | --- | --- | --- | --- |
| **Family: Acanthaceae** | | | | |
| 1 | *Andrographis paniculata* (Burm. f.) Nees | H | Diterpenes (andrographolide), 14-deoxyandrographolide,14-deoxy-11,12-didehydroandrographolide | Kumar et al., 2004 |
| 2 | *Barleria cristata* L. | H | Flavonoids, apigenin, quercetin, quercetin-3-o-β-D-glucoside, naringenin, luteolin, apigenin glucuronide | Gambhire et al., 2009 |
| 3 | *Barleria prionitis* L. | H | Barlerinoside, shanzhiside methyl ester, 6-o-trans-p-coumaroyl-8-o-acetylshanzhiside methyl ester, barlerin, acetylbarlerin, 7 methoxydididerroside, lupulinoside | Ata et al., 2009 |
| 4 | *Blepharis maderaspatensis* (L.) B. Heyne ex Roth | H | Tannins, saponins, steroids, terpenoids, flavonoids, alkaloids, phenols, glycosides, amino acids, phlobatannins, anthroquinones, sterols, q `uinines, diethylphthalate, n- hexadeconic acid, avlo topic acid, butyl phosphoric acid, ethyl 4-methoxy-2- methyl butyl ester, cyclopentyl methyl phenyl phosphine oxide, 2-methyl -7H, 1,3,4-thiadiazolo (3,2-a) pyrimidin-7-one, 1 H -4- azocyclo prop indene, octahydro -4- methyl- thiazolo (3,2-a) pyridinum, 3- carboxy2,3- dihydro-8- hydroxy hydroxide, phosphoramidic acid, N,N- di methyl mono ethyl ester | Rajasekaran et al., 2012; Prabakar and Wembonyama, 2016 |
| 5 | *Dicliptera chinensis* (L.) Juss. | H | Ferulic acid, daucosterol, gardenoside, chlorogenic acid, epicatechin, kaempferol | Huang et al., 2012 |
| 6 | *Dicliptera roxburghiana* Nees | S | Flavonoids, phenolics, tannins, alkaloids, saponins, tarpenoids, coumarines | Ahmad et al., 2013 |
| 7 | Eranthemum pulchellum Andrews | S | Eranthemoside | Fischer et al., 1987 |
| 8 | *Indoneesiella echioides (*L.) Sreem | H | Flavonoids, terpenes, triterpenoid saponins, saponins, steroids, phenolic compounds, amino acids | Elaiyaraja and Chandramohan, 2016 |
| 9 | *Justicia adhatoda* L. | S | Palmatine, berberine, vasinone, vasicine, vasicinone, vasicicoline | Gulfraz et al., 2005; Khursheed et al., 2010 |
| 10 | *Justicia procumbens* L. Don. | H | Neojusticin A, justicidin B, Justicidin A, taiwanin E methyl ether, neojusticin B, chinensinaphthol, diphyllin, taiwanin E | Chen et al., 1996 |
| 11 | *Lepidagathis cristata* Willd. | H | Oleic acid, 3-(octadecyloxy) propyl ester | Abubacker and Devi, 2014. |
| 12 | *Lepidagathis cuspidata* Nees | S | Steroids, tannins, phenolics, saponins, alkaloids, flavonoids | Rattan et al., 2016 |
| 13 | *Peristrophe paniculata* (Forsk.) Brumitt | H | Steroids, terpenoids, flavonoids, alkaloids, glycosides, phenols, tannins, carbohydrates | Srikanth et al., 2018 |
| 14 | *Phlogacanthus thyrsiformis* (Hardw.)Mabb. | S | Phenol, 2, 4-bis (1, 1-dimethylethyl), 1-hexadecene, 1-hexadecanol, hexadecanoic acid, octadecanoic acid methyl ester, 1-nonadecene | Devi and Singh, 2013 |
| 15 | *Ruellia tuberosa* L. | H | E-phytol, tributylacetyl citrate, heptacosane, m-xylene, p-xylene, heptanes, borneol, sexton, heneicosane, 2-methyl-2-pentanol, 1- methyl-1-cyclopentanol | Moronkola et al., 2015 |
|  | **Amaranthaceae** | | | |
| 16 | *Achyranthes aspera* L. | H | Saponins, oleonolic acid, dihydroxy ketones, alkaloids | Srivastav et al., 2011 |
| 17 | *Achyranthes bidentata* Bl. | H | Triterpenoid saponin, steroid ketone, polysaccharide, polypeptide, alkaloids, flavonoides, iridoid, organic acid, volatile oil | Shen et al., 2011 |
| 18 | *Alternanthera sessilis* (L.) R. Br. Ex DC. | H | Polyphenol, catechin, rutin, ellagic acid, quercetin | Mondal et al., 2014 |
| 19 | *Amaranthus hybridus* L. | H | Flavonoides, steroids, terpenoids, cardiac glycosides | Maiyo et al., 2010 |
| 20 | *Amaranthus spinosus* L. | H | Flavonoides, steroids, terpenoids, cardiac glycosides | Maiyo et al., 2010 |
| 21 | *Amaranthus tricolor* L. | H | Galactosyl diacylglycerols (1-3) | Jayaprakasam et al., 2004 |
| 22 | *Celosia argentea* L. | H | Flavonoids, phenol | Fa, 2013; Talagari and Hullatti, 2015 |
| 23 | *Chenopodium album* L. | H | Cinnamic acid amides ( N-trans-feruloyl 4' –omethyldopamine, N-trans-feruloyl 3' -*O*-methyldopamine, N-trans-feruloyl tyramine, N-trans-4-*O-* methylferuloyl 3',4' -*O*-dimethyldopamin, N-trans-4-O-Methylcaffeoyl 3' -*O*-methyldopamine, N-trans-feruloyl tryptamine, N-trans-4-*O*-methylferuloyl 4' -*O*-methyldopamine | Cutillo et al., 2003 |
| 24 | *Dysphania* ambrosioides (L.) Mosyakin & Clemants | H | *p*-cymene, (*Z*)-ascaridole, (*E*)-ascaridole , limonene, transpinocarveol, aritasone, b-pynene, myrcene, phelandrene, alcanphor, α –terpineol | Gadano et al., 2002; Jardim et al., 2008 |
| 25 | *Chenopodium* antarcticum (Hook. f.) Hook. f. | H | Phenolics, flavonoids, saponins, ecdysteroids, triterpenoids, sesquiterpenes ( α & β-eudesmol) | El-Sayed et al., 1989; Kokanova-Nedialkova et al., 2009 |
| 26 | *Chenopodium foliosum* (Moench) Aschers. | H | Phenolics, flavonoids, saponins, ecdysteroids, triterpenoids, Flavonol glycosides (6-methoxykaempferol-3-*O*-*β*-gentiobioside, gomphrenol-3-*O*-*β*-gentiobioside, gomphrenol-3-O-*α*-l-rhamnopyranosyl-(1 → 2)[*β*-d-glucopyranosyl-(1 → 6)]-*β*-d-glucopyranoside), patuletin-3-*O*-*β*-gentiobioside, spinacetin-3-*O*-*β*-gentiobioside | Kokanova-Nedialkova et al., 2009; Kokanova-Nedialkova et al., 2011 |
| 27 | *Cyathula tomentosa* (Roth) Moq. | S | Flavanone, phenolic acid, 7-sitosterol | Prasad and Sati, 2010, Agarwal, 2011 |
| 28 | *Digera muricata* (L.) Mart. | H | Flavonoids, tannins, alkaloids, saponins, phenols, terpenes | Khan and Younus, 2011 |
|  | **Amaryllidaceae** | | | |
| 29 | *Allium cepa* L. | H | Flavonol glucosides, quercetin 3, 4ʹ-O-diglucosides and quercetin 4ʹ-O-monoglucosides, phenolics, sulphur compounds | Price et al., 1997 |
| 30 | *Allium consanguineum* Kunth | H | Phenolics, saponins, sulphur compounds | Keusgen et al., 2006 |
| 31 | *Allium fedschenkoanum* Regal | H | Phenolics, saponins, sulphur compounds | Keusgen et al., 2006 |
| 32 | *Allium humile* Kunth | H | Phenolics, saponins, sulphur compounds | Keusgen et al., 2006 |
| 33 | *Allium sativum* L. | H | Phenolics, saponins, sulphur compounds, allicin, alliin, terpenoids, flavanoids, reducing sugars | Hughes and Lawson,1991; Keusgen et al., 2006; Gyawali, 2011 |
| 34 | *Allium wallichii* Kunth. | H | Phenolics, saponins, sulphur compounds, terpenoids, flavanoids, reducing sugars | Keusgen et al., 2006; Gyawali, 2011 |
|  | **Anacardiaceae** | | | |
| 35 | *Cotinus coggygria* Scop. | S | Disulfuretin, sulfuretin, sulfurein, gallic acid, methyl gallate, pentagalloyl glucose | Westenburg et al., 2000 |
| 36 | *Mangifera indica* L. | T | Saponin, steroids, tannin, flavonoid, reducing sugars, cardiac glycosides, anthraquinone, gallic acid, 3,4-dihydroxy benzoic acid, gallic acid methyl ester, mangiferin, catechin, epicatechin, benzoic acid, benzoic acid propyl ester | Aiyelaagbe and Paul, 2009; Núñez Sellés et al., 2002 |
| 37 | *Pistacia integerrima* (Stew.) Rech. F. | H | Quercetin-3-*O*-β-d-glucopyranoside, kaempferol-3-*O*-β-d-glucopyranoside,quercetin-3-*O*-(6″-*O*-syringyl)-β-d-glucopyranoside, kaempferol-3-*O*-(4″-*O*-galloyl)-α-l-arabinopyranoside, rutin, aglycons, quercetin, kaempferol,apigenin | Ahmad et al., 2008 |
| 38 | *Rhus wallichii* Hook. f. | S | Kaempferol, quercetin, myricetin, gallic acid, quercetin-3-O-arabinopyranoside, quercetin-3-O-xyloside, quercetin-3-O-galactoside, β-sitosterol, glucoside, p-sitosterol | Sinha et al., 1986 |
| 39 | *Rhus parviflora* Roxb. ex DC. | S | Biflavonoids mesuaferrone B, rhusflavon, agathisflavone, sulfuretin, aureusidin, aureusidin-6-o-β-D-galactopyranoside, cupressuflavone | Shrestha et al., 2012; Shrestha et al., 2013 |
| 40 | *Semecarpus anacardium* L.f. | T | Tetrahydroamentoflavone | Selvam and Jachak, 2004 |
| 41 | *Spondias pinnata* (L.f.) Kurz | T | Phenolics, flavonoids | Maisuthisakul et al., 2008 |
|  | **Annonaceae** | | | |
| 42 | *Annona Squamosa* L. | T | Glycoside, alkaloids, saponins, flavonoids, tannins, carbohydrates, proteins, phenolic compounds, phytosterols, amino acids, anonaine, aporphine, coryeline, isocorydine, norcorydine, glaucine | Pandey and Barve, 2011 |
|  | **Apiaceae** | | | |
| 43 | *Angelica glauca* Edgew | H | coumarins, acetylenic compounds, chalcones, sesquiterpenes and polysaccharides | Sarker and Nahar, 2004 |
| 44 | *Apium graveolens* L. | H | Apigenin | Hamza and Amin, 2007 |
| 45 | *Bepleurum falcatum L.* | H | Triterpene saponins (saikosaponins), lignans, flavonoids, essential oils and polysaccharides | Pan, 2006; Ashour and Wink, 2011 |
| 46 | *Bupleurum hamiltonii* N. P. Balakr. | H | Triterpene saponins (saikosaponins), lignans, flavonoids , essential oils and polysaccharides | Pan, 2006; Ashour and Wink, 2011 |
| 47 | *Carum carvi* L. | H | Monoterpene hydrocarbons, oxygenated monoterpenes, oxygenated sesquiterpenes, saturated and unsaturated fatty acids, aldehydes, ketones, esters, triacylglycerols, polysaccharides, lignin | Johri, 2011 |
| 48 | *Centella asiatica* (L.) Urban | H | Triterpenoid compounds, glycosides (asiaticoside and madecassoside), aglycones (asiatic acid and madecassic acid) | Wu et al., 2012 |
| 49 | *Coriandrum sativum* L. | H | 2*E*-decenal, decanal, 2*E*-decen-1-ol, *n*-decanol, 2*E*-tridecen-1-al, 2*E*-dodecenal, dodecanal, undecanol, undecanal | Lo et al., 2004 |
| 50 | *Daucus carota* L. | H | Luteolin, luteolin 3′-O-β-D-glucopyranoside, luteolin 4′-O-β-D-glucopyranoside, flavones, polyacetylenes, carotenoids | Kidmose et al., 2004; Kumarasamy et al., 2005 |
| 51 | *Foeniculum vulgare* Mill. | H | Alkaloids, flavonoids, tannins, saponins, cardiac glycosides | Kaur and Arora, 2009 |
| 52 | *Heracleum candicans* Wall. ex DC. | H | Prenylated coumarin suberosin, tschimgin (bornyl *p*-hydroxybenzoate), tschimganin (bornyl vanillate), ferutinin (ferutinol *p*-hydroxybenzoate), tefernin (ferutinol vanillate), furocoumarin, heraclenin | Sharma et al., 1964; Trusheva et al., 2010 |
| 53 | *Heracleum lanatum* Michaux | H | Coumarins,  linear-furocoumarins [psoralen, xanthotoxin, bergapten, isopimpinellin, angular-furocoumarin | Matsuda et al., 2005 |
| 54 | *Hydrocotyle sibthorpioides* Lam. | H | *Trans-* β*-farnesene,*  triterpenoidal saponins, hydrocosisaponins A–F, hydrocotyloside VII | Asakawa et al., 1982; Huang et al., 2008 |
| 55 | *Pimpinella acuminata* (Edgew.) Cl. | H | Parsley apiole, coniine, n-pentadecane, 1-methyl-2-pentyl piperidene, heptadecane, apiole, β-caryophyllene, dill apiole, myristicin | Mohan and Melkani, 2016 |
| 56 | *Pimpinella diversifolia* DC. | H | Trans-anethole, (E)-anethole, methyleugenol, trans-isoosmorhizole, dictamnol | Tabanca et al., 2007 |
| 57 | *Pleurospermum angelicoides* DC. | H | Terpenoid, nothoapiole (5-allyl-4,6,7-trimethoxy-benzo[1,3] dioxide | Mathela et al., 2015 |
| 58 | *Trachyspermum ammi* (L.) Spr. | H | Alkaloids, flavonoids, tannins, saponins, cardiac glycosides, Phenolic terpenoids, cymene r-terpinene, thymol | Kaur and Arora, 2009; Zahin, et al., 2010, Goudarzi, et al., 2010 |
|  | **Apocynaceae** | | | |
| 59 | *Alstonia scholaris* (L.) R. Br. | T | Alkaloid, bisindole *O*-acetylmacralstonine | Keawpradub et al., 1999 |
| 60 | *Asclepias curassavica* L. | S | Coroglaucigenin, 12β-hydroxycoroglaucigenin, calotropagenin, desglucouzarin, 6′-*O*-feruloyl-desglucouzarin, calotropin, uscharidin, asclepin, 16α-hydroxyasclepin, 16α-acetoxycalotropin, 16α-acetoxyasclepin, cardenolide | Li et al.,2009 |
| 61 | *Calatropis gigantea* (L.) Br. | S | Triterpenes, flavonoid glycosides, steroidal saponins, alkaloids, tannins, phenol, sterols, anthraquinones, proteins, quinones | Kiuchi et al.,1998; Dhivya and Manimegalai, 2013 |
| 62 | *Calotropis procera* (Ait.)R.Br | S | Proteolytic enzymes, cardenolides, alkaloids, tannins, triterpenes, flavonoids | Nenaah, 2013 |
| 63 | *Carissa carandas* L. | S | Phenolic acids, flavanols, flavonols, anthocyanins, cardiac glycosides, triterpenoidal constituent carissone , β-sitosterol, pectin, alkaloids, tannins, cardiac glycosides, steroids,3-hydroxy-4, 7-megastigmadiene-9-one, awabukinol, 1-oxo-erythrodiol, lupeol, 10-acetylpatrinoside | Hegde et al., 2009; Siddiqi et al., 2011; Vaghasiya et al., 2011; Shafeeq et al., 2014 |
| 64 | *Carissa opaca* Stapf. ex Haines | S | Phenolics, flavonoids, imonene, 2'-hydroxyacetophenone, vanillin, naphthalenone, 2,3,3-trimethyl-2-(3-methylbuta-1,3-dienyl)-6-methylenecyclo-hexanone, 2-benzene -dicarboxylic acid, mono(2-ethylhexyl) ester, β-sitosterol, vitamin E, rutin, quercetin, lupeol, epigallocatechin | Sahreen et al., 2011; Ahmed et al., 2015 |
| 65 | *Thevetia* peruviana (Pers.) K. Schum. | T | Alkaloids, phenols, flavonoids, steroids, tanins, coumarins, reducing sugars, triterpenoids, essential oils | Sowjanya et al., 2013 |
| 66 | *Lochnera* rosea (L.) Rchb. ex Spach | S | Alkaloids ( vincamine ), tannins | Nayak et al., 2007 |
| 67 | *Cryptolepis buchanani* Roem. & Schult. | S | Alkaloid (buchanine), triterpenes,  α and β amyrin, cardenolide cryptosin | Maurya et al., 2003 |
| 68 | *Cryptolepis dubia* (Burm. f.) M.R. Almeida | S | Alkaloid (Buchananine) | Sriset et al., 2017 |
| 69 | *Gymnema syvestre* (Retz.) R. Br. | S | Carbohydrates, saponins, phytosterols, phenols, flavonoids, Terpenoids, tannins | Khanna and Kannabiran, 2007 |
| 70 | *Holarrhena antidysenterica* Linn. | T | Phenols, tannins, saponin, alkaloids, flavonoids | Ahmad and Beg, 2001; Parekh and Chanda, 2007 |
| 71 | *Marsdenia roylei* Wight & Arn. | S | Pregnanes (desacylkondurangogenin C, deniagenin), pregnane glycosides (denin, marsin) | Gupta et al., 2003 |
| 72 | *Nerium oleander* L. | S | Polysaccharides, cardenolides, glycosides, triterpenoids | Gupta and Mittal, 2010 |
| 73 | *Pergularia daemia* (Forsk.) Chiov. | S | Alkaloids, flvonoides, tannins, terpenoids, carbohydrates, proteins | Karthishwaran et al., 2010 |
| 74 | *Quirivelia frutescens* (L.) M. R.Br. & S. M. Almeida | C | Polyphenols, terpenoids, alkaloids, phytosterols, carbohydrates, coumarins, glycosides, flavonoids | Kumarappan, et al., 2015 |
| 75 | *Rauvolfia serpentina* (L.) Benth. Ex Kurz | S | Alkaloids (vomilenine and reserpine), phenolics | Madhusudanan, et al., 2008; Nair et al., 2013 |
| 76 | *Tabernaemontana divaricata* (L.) R. Br. ex Roem. & Schult. | S | Alkaloids, terpenoids, steroids, flavonoids, phenyl propanoids, phenolic acids | Pratchayasakul , et al., 2008 |
| 77 | *Vallaris solanacea* (Roth) Kuntze | S | Glycosides vallarisoside , 3β-O-(α-acofriosyl), benzyl 2-O-β-apiofuranosyl-(1→2)-β- D-glucopyranosyl-2, O- acetyl-solanoside (O-acetyl-acofreosyl digitoxigenin) | Karmakar et al., 2011 |
| 78 | *Vincetoxicum hirundinaria* Medic. Subsp. Glaucum (Wall. ex Wt) Hara | S | Monoterpenoids, sesquiterpenoids, fatty acid derivatives, benzenoids, phenylpropanoids, nitrogen-bearing compound | Jürgens et al., 2008 |
| 79 | *Wrightia arborea* (Dennst.) Mabb. | T | Steroids, saponins, alkaloids, phenol, flavonoids, tannins, terpenoids | Zahan et al., 2013 |
|  | **Aquifoliaceae** | | | |
| 80 | *Ilex dipyrena* Wallich | T | Saponins, flavonoids, aldehydes, hemiterpene glycosides, triterpenes, alkanes, anthocyanins, pentyl esters, hexyl esters | Kothiyal et al., 2012 |
| **Araceae** | | | | |
| 81 | *Acorus calamus* L. | H | β- asarone, α-asarone | Mukherjee et al., 2007 |
| 82 | *Amorphophallus paeoniifolius* (Dennst.) Nicolson. | H | Carbohydrates, proteins, steroids, flavonoids | Hurkadale et al., 2012 |
| 83 | *Arisaema intermedium* Bl. | H | Lectins | Kaur et al., 2005 |
| 84 | *Arisaema jacquemontii* Blum | H | Alkaloids, tannins, rotenoids, phenolic acids, flavonoids, anthocyanidins | Singh et al., 2006; Sudan et al., 2014 |
| 85 | *Arisaema tortuosum* (Wall.) Schott Hook.f. | H | Flavonoids, alkaloids, saponins, triterpenoids and lectins | Nile and Park 2014. |
| 86 | *Colocasia esculenta* (L.) Schott | H | Favonoids, β-sitosterol, steroids, fatty acids, sterols, lignans | Prajapati et al.,2011; Kim et al.,2010 |
|  | **Araliaceae** | | | |
| 87 | *Hedera nepalensis* K. Koch. | H | Flavonoids, steroids, tannins, terpenoids, cardiac glycosides | Jafri et al.,2017 |
|  | **Arecaceae** | | | |
| 88 | *Calamus tenuis* Roxb. | S | Alkaloid, tannin, flavonoid, steroid, saponin | Ahmed et al.,2014 |
|  | **Asparagaceae** | | | |
| 89 | *Agave americana* L. | S | Saponins, sapogenins, cutin | Matic, 1956 |
| 90 | *Asparagus adscendens* Roxb. | H | Steroidal saponins, essential oils, asparagine, arginine, tyrosine, flavonoids (kaempferol, quercetin, rutin), resin, tannin | Negi et al., 2010 |
| 91 | *Asparagus curillus* Buch.-Ham. Ex Roxb. | S | Steroidal saponins, essential oils, asparagine, arginine, tyrosine, flavonoids (kaempferol, quercetin, rutin), resin, tannin | Negi et al., 2010 |
| 92 | *Asparagus filicinus* Buch.-Ham. Ex D. Don | S | Steroidal saponins, essential oils, asparagine, arginine, tyrosine, flavonoids (kaempferol, quercetin, rutin), resin, tannin | Negi et al., 2010 |
| 93 | *Asparagus racemosus* Willd | S | Thiophene, thiazole, aldehyde, ketone (vanillin), quercetin, rutin, hyperoside, diosgenin, quercetin-3-glucuronide, saponins | Negi et al., 2010 |
| 94 | *Chlorophytum tuberosum* Baker | H | Alkaloids, triterpenoids, steroids, glycosides, saponins | Mandal and Nandi, 2013 |
| 95 | *Drimia indica* (Roxb.) Jessop | H | Flavonoids, tannins, coumarins, phenols | Chittoor et al., 2012 |
| 96 | *Polygonatum cirrhifolium* (Wall.) Royle | H | Saponins, phytohormones, glycosides, flavonoids and alkaloids | Khan et al., 2012 |
| 97 | *Polygonatum multiflorum* (L.) Allioni | H | Flavones, quinones, stilbenes | Lin et al., 2015 |
| 98 | *Polygonatum verticillatum* L. | H | Saponins, alkaloids, phenols, flavonoids | Khan et al., 2010; Khan et al., 2012 |
|  | **Asphodelaceae** | | | |
| 99 | *Aloe vera* (L.) Burm. f. | H | Sterols (β-Sitosterol, campesterol, stigmasterols) | Tanaka et al., 2006 |
|  | **Aspleniaceae** | | | |
| 100 | *Asplenium nidus* L. | Fn | Phenols,flavonoids  Gliricidin7-O-hexoside, quercetin-7-O-rutinoside, keampferol-3-O-rutinoside, myricetin-3-O-rhamnoside | Jarial et al., 2016 |
| 101 | *Asplenium trichomanes* L. | Fn | Glycosides, carbohydrate, proteins & free amino acids, phenolic compounds, tannins, terpenoids, flavonoids, Phlobatannins | Mir et al., 2013 |
| **Asteraceae** | | | | |
| 102 | *Adenostemma lavenia* (L.) kuntz. | H | Amines, aldehydes, fatty acids, terpenoids-steroids, alkaloids, aromatic & aliphatic hydrocarbons, phenolics, oligopeptides | Fauzan et al., 2018 |
| 103 | *Ageratum conyzoides* L. | H | 5,6,7,8,3 , 4 , 5 –heptamethoxyflavone, coumarin, 5,6,7,8,3 -pentamethoxy-4 , 5 – methylenedioxyflavone | Moreira et al., 2007 |
| 104 | *Anacyclus pyrethrum* (L.) Lag. | H | Alkylamide, Decadien, dodecadien, tetradecadien-tyramide, anacyclin, dehydroanacyclin | Greger, 1984; Sharma et al., 2010 |
| 105 | *Anaphalis contorta* D. Don. Hook. F. Kuntze | H | Quercetin, apigenin, Kaempferol, flavonoid glycoside, tiliroside, anaphaloside, isoquercitrin, astragalin | Lin et al., 1976; Lin, 1993 |
| 106 | *Anaphalis margaritaceae* (L) L.N: Bakol | H | Flavonoids, flavonols, diterpenes, hydroxylactones | Borchardt et al., 2008; Ren et al., 2009 |
| 107 | *Anaphalis triplinervis* (Sims.) C.B. Clarke | H | Polyacetylenide | Dembitsky et al., 2003 |
| 108 | *Arctium lappa* L. | H | Tannin, arctigenin, arctin, β-eudesmol, caffeic acid, chlorogenic acid, inulin, trachelogenin 4, sitosterol-β-D-glucopyranoside, lappaol, diarctigenin | Chan et al., 2011 |
| 109 | *Artemisia annua* L. | H | Artemisinin, Artemisinic acid, arteanniun B, phenolic compounds, flavones | Bilia et al., 2006; Bora and Sharma 2011 |
| 110 | *Artemisia capillaris* Thunb. | H | scoparone (SCO, 6,7-dimethoxycoumarin), capillarisin, flavonoids | Wang et al., 2006; Bora and Sharma 2011 |
| 111 | *Artemisia gmelinii* Webb. ex. Stechm. | H | Artemisia ketone, 1,8-cineole | Mathela et al., 1994 |
| 112 | *Artemisia maritima* L. | H | Santonin , α-Thujone, β-thujone | Mathela et al.,1994, Bora and Sharma 2010 |
| 113 | *Artemisia nilagirica* (Cl) Pamp. | S | Terpenoids, flavonoids, alkaloids, amino acids, quinines, tannins | Bora and Sharma 2011 |
| 114 | *Artemisia roxburghiana* Wall ex Besser | S | α-Thujone, β-thujone | Mathela  *et al.,* 1994 |
| 115 | *Artemisia indica* Willd | H | Terpenoids, flavonoids, coumarins, caffeoylquinic acids, sterols, acetylenes | Bora and Sharma 2011 |
| 116 | *Aster albescens* (DC.) Hand.-Mazz. | H | Germacrene D, β- phellandrene, δ- selinene, β-eudesmol, flavonoids, acetylenes, coumarins, monoterpenes, saponins | Kumar  *et al.,* 2017, Qin, 1998 |
| 117 | *Aster peduncularis* Wall | H | Flavonoids, acetylenes, coumarins, monoterpenes, saponins | Qin, 1998 |
| 118 | *Aster flaccidus* Bung. | H | Flavonoids, acetylenes, coumarins, monoterpenes, saponins | Qin, 1998 |
| 119 | *Bidens pilosa L.* | H | Flavonoids, acetylenes, 1-phenyl-1,3-diyn-5-en-7-ol-acetate | Andrade‐Neto et al., 2004 |
| 120 | *Blumea lacera* (Burm. f.) DC. | H | β-caryophyllene, thymol hydroquinone dimethyl ether, caryophyllene oxide, a-humulene, and E-b-farnesene, glycosides, flavonoids, flavones, campesterol | Ragasa and Rideout, 2007 |
| 121 | *Caesulia axillaris* Roxb. | H | Limonene, euasarone, asaronaldehyde, trans-caryophyllene | Mishra et al., 2012 |
| 122 | *Carthamus tinctorius* L. | H | Flavonoids,  guanosine, syringing, linoleic acid, tocols (tocopherols and tocotrienols) | Bozan and Temelli, 2008; Fan et al., 2009 |
| 123 | *Centipedia minima* L. | H | Taraxasteryl acetate, taraxaserol, stigmasterol, β-stitosterol, arnidiol, flavonoids, sesquiterpene lactones, amides | Wu et al., 1991 |
| 124 | *Cichorium intybus* L. | H | Flavonoids, anthocyanins, caffeoyl derivatives, Chicoric acid | Innocenti et al., 2005 |
| 125 | *Erigeron bonariensis*L. | H | Phenolic acids, flavonoids, tannins, triterpenoids (lupeol), betulin, betulinic acid, oleanolic acid, ursolic acid | Shahwar et al., 2012 |
| 126 | *Erigeron canadensis*L. | H | Phenolic compounds (gallic acid, vanillic acid, catechol, syringic acid) | Queiroz et al., 2012 |
| 127 | *Dahlia* pinnata Cav. | H | Anthocyanins | Robinson and Robinson, 1931 |
| 128 | *Echinops echinatus* Roxb. | H | Isobutylamides, steroidal glycoalkaloids | Semwal et al., 2007 |
| 129 | *Eclipta erecta*L. | H | Flavonoid, phenolcarboxylic acids, coumestans wedelolactone, demethyl-wedelolactone, wedelolactone, sitosterol, stigmasterol | Wagner et al., 1986; Mors et al., 1989 |
| 130 | *Emilia sonchifolia* (L.) DC. | H | Alkaloids, flavonoids, terpernes, kaempherol-3-d-galactoside, quercitrin, quercetin, rutin, ursolic acid, senkirkine, doronine, β-sitosterol, stigmasterol | Couto et al., 2011 |
| 131 | *Ageratina adenophora* (Spreng.) R.M. King & H. Rob. | H | Terpenes (monoterpenes, sesquiterpenes), cadinene | Baruah et al., 1994; Zhao et al., 2008 |
| 132 | *Eupatorium cannabinum* L. | H | Germacrene D, α-farnesene, δ-2-carene, elemol, α-cadinol | Senatore et al., 1994 |
| 133 | *Chromolaena* odorata (L.) R.M. King & H. Rob. | S | Tannins, phenols, saponin, α-pinene, cadinene, camphor, limonene, β-caryophyllene, cadinol isomer | Inya-Agha et al., 1987 |
| 134 | *Eupatorium perfoliatum* L. | H | Sesquiterpene lactones, guaianolide, germacranolides. flavonoids (aglycones eupafolin, hispidulin, patuletin, kaempferol) | Maas et al., 2011 |
| 135 | *Galinsoga parviflora* | H | Flavonoids, patulitrin, quercimeritrin, quercitagetrin, caffeoyl derivatives, galinsosides A & B, flavanone glucosides | Ferheen et al., 2009; Bazylko et al., 2012 |
| 136 | *Pseudognaphalium* luteoalbum (L.) Hilliard & B.L. Burtt | H | Flavonoids, sesquiterpenes, diterpenes, triterpenes, phytosterols, anthraquinones, caffeoylquinic acid derivatives | Zheng et al., 2013 |
| 137 | *Pseudognaphalium* hypoleucum (DC.) Hilliard & B.L. Burtt | H | Flavonoids (luteolin-4′-*O*-glucoside, luteolin), phytosterols | Zheng et al., 2013; Zhang et al., 2016 |
| 138 | *Gnaphalium polycaulon* Per. | H | Flavonoid, alkaloids, tannins, saponinis | Kaminidevi et al., 2014 |
| 139 | *Helianthus annus* L. | H | Lignans, tanegool, inoresinol, lariciresinol, dihydro-dehydrodiconiferilic alcohol, l-(4‘-hydroxy-3‘-methoxyphenyl)-2-[4‘ ‘-(3hydroxypropyl)-2‘ ‘-methoxyphenoxy] propane-l,3-diol | Macías et al., 2004 |
| 140 | *Duhaldea* cappa (Buch.-Ham. ex D. Don) Pruski & Anderb. | S | Sesquiterpene lactones, friedelin, epifriedelanol (3),7) α-amyrin (4), β-amyrin, oleanolic acid, ursolic acid, stigmast-4-en-3-one, stigmasta-4,22- dien-3-one, β-sitosterol, stigmasterol, 7- oxo-β-sitosterol, stigmast-5-ene-3b,7b-diol, stigmasta-5,22-diene-3b,7b-diol, stigmast-5-ene-3b,7adiol, stigmasta-5,22-diene-3b,7a-diol, daucosterol | Xie et al., 2007 |
| 141 | *Inula cuspidata* (DC.) C.B. Clarke | S | Sesquiterpenoids, monoterpenoids, benzenoid components | Verma et al., 2014 |
| 142 | *Jurinea dolomaea* Boiss. | H | Sesquiterpene lactones, triterpenes, flavonoid, phenolic compounds | Shah et al., 2014; Singh et al., 2015 |
| 143 | *Paramicrorhynchus* procumbens (Roxb.) Kirp. | H | Alkaloids, phenols, tannins, flavonoids, steroids, glycosides and triterpenes | Reddy and Mishra, 2012. |
| 144 | *Chamomilla* *recutita*(L.) Rauschert | H | Terpenoids α-bisabolol, azulenes, chamazulene, coumarins ( herniarin, umbelliferone ), phenylpropanoids ( chlorogenic acid, caffeic acid), flavones ( apigenin, apigenin7-O-glucoside, luteolin, luteolin-7-O-glucoside), flavonols ( quercetin, rutin), flavanone ( naringenin) | Gupta et al., 2010 |
| 145 | *Parthenium hysterophorus* L. | H | Sesquiterpene lactones, parthenin | Datta and Saxena, 2001 |
| 146 | *Aucklandia* costus Falc. | H | Costunolide, dehydrocostus lactone, sesquiterpene lactones | Chen et al., 1995; Pandey et al., 2007 |
| 147 | *Sassurea obvallata* (DC.) Edgew. | H | Akaloids, flavonoids, terpenoids, saponin, phenols, Glycosides Proteins | Semwal et al., 2014 |
| 148 | *Senecio nudicaulis* Buch.-Ham ex D Don | H | β- caryophyllene, α- humulene, germarcrene, linoleic acid | Kumar et al., 2017 |
| 149 | *Solidago virgaurea* L. | H | α-tocopherol quinone, trans-phytol, 2-methoxybenzyl2,6-dimethoxybenzoate | Gross et al., 2002 |
| 150 | *Picrosia* longifolia D. Don | H | Carotenoids, catechin, rutin, quercetin | Khan et al., 2012 |
| 151 | *Sonchus brachyotus* DC. | H | Ascorbic acid, phenolic acids, catechin, rutin, quercetin, myrecetin, apigenin, Kaempferol | Seal, 2016 |
| 152 | *Sonchus olereaceus* L. | H | Caftaric acid, chlorogenic acid, chicoric acid, caffeic acid, flavones (apigenin, luteolin), flavonols (Kaempferol, quercetin) | Gatto et al., 2011; Ou et al., 2013 |
| 153 | *Sphaeranthus senegalensis* DC. | H | Glycosides, saponins and tannins | Amos et al., 2001 |
| 154 | *Acmella* paniculata (Wall. ex DC.) R.K. Jansen | H | Alkamides (Spilanthol) | Tiwari et al., 2011 |
| 155 | *Tagetes erecta* L. | H | Gallic acid, gallicin, quercetagetin, 6-hydroxykaempferol-O-hexoside, patuletin-O-hexoside, quercetin, limonene, β‐caryophyllene, piperitone | Hethelyi et al., 1986; Gong et al., 2012 |
| 156 | *Tagetes minuta* L. | H | β‐ocimene, dihydrotagetone, tagetone, and (E)‐ocimenone | Hethelyi et al., 1986 |
| 157 | *Hippolytia* dolicophylla (Kitam.) K. Bremer & Humphries | H | Monoterpenes | Özek, 2018 |
| 158 | *Ajania* nubigena (Wall. ex DC.) C. Shih | H | Monoterpenes, sesquiterpenes, 1,8- cineole, linolool, citronellal | Haider et al., 2015; Özek, 2018 |
| 159 | *Hippolytia* tomentosa (DC.) Tzvelev | H | Monoterpenes | Özek, 2018 |
| 160 | *Taraxacum ofﬁcinale* Weber. | H | Alkaloids, flavonoids, steroids, saponins, tannins, triterpenoid, hydroxycinnamic acids, chicoric acid, monocaffeyltartaric acid, chlorogenic acid, coumarins, cichoriin, aesculin | Williams et al., 1996; Mir et al., 2013 |
| 161 | *Tridax procumbens* L. | H | α-Terpinene, α-Terpineol, β-Pinene | Rajkumar and Jebanesan, 2007 |
| 162 | *Baccharoides* anthelmintica (L.) Moench | H | Trivernolin, 1, 3-divernolin, vernolic acid | Krewson et al., 1962 |
| 163 | *Vernonia cinerea* L. | H | 8 α-tigloyloxy-hirsutinolide-13-O-acetate, 8 α-tigloyloxyhirsutinolide, 8 α-(4-hydroxymethacryloyloxy)-hirsutinolide-13-O-acetate, lactones (8 α-epoxymethacryloyloxy-hirsutinolide-13-O-acetate, hirsutinolide-13-O-acetate, piptocarphin D, stigmasterol, stigmasterol‐β‐D‐glucopyranoside, luteolin‐7‐0‐glucopyranoside, dotriacontanoic acid | Tandon et al., 1998; Chea et al., 2006 |
| 164 | *Allardia glabra*Decne. | H | α-bisabolol, valeranone, chamazulene, spathulenol, β-caryophyllene, caryophyllene oxide | De et al., 2017 |
| 165 | *Xanthium indicum* Koenig. | H | Sesquiterpene lactones, glycoside, phenols, polysterols, alkaloids | Kamboj and Saluja, 2010 |
|  | **Athyriaceae** | | | |
| 166 | *Allantodia* maxima (D. Don) Ching | Fn | Glycosides, carbohydrate, proteins & free amino acids, resins, saponins phenolic compopunds & tannins, volatile oils, terpenoids, flavonoids, phlobatannins | Mir et al., 2013 |
| 167 | *Diplazium esculentum* Sw. | Fn | Steroids, triterpenoids, phenols, flavones, flavonoids (myrcetin, alphatocopherol) | Akter et al., 2014 |
|  | **Balsaminaceae** | | | |
| 168 | *Impatiens* *walleriana*Hook. f. | H | 2-methoxy-1,4-naphthoquinone | Ding et al., 2008 |
| 169 | *Impatiens bicolor* Royle. | H | Alkaloids, tannins, steroids, saponins, flavonoids | Anwer et al., 2013 |
| 170 | *Impatiens sulcata* Wall. | H | Napthoquinones, flanonoids, glycosides, sapogenins | Singh et al., 2017 |
|  | **Basellaceae** | | | |
| 171 | *Basella alba* L. Var. Rubra (L.) Stew | H | Alkaloids, flavonoids, carbohydrate, Saponin, tannin, mucilage, steroids, triterpenoids, phenols | Abdul Kalam et al., 2013; Priya et al., 2015 |
|  | **Begoniaceae** | | | |
| 172 | *Begonia picta* Smith | H | Flavonoids (vitexin, iso-vitexin, orientin, iso-orientin, 1,3- dihydroxy-6,7-dimethoxyxanthone) | da Silva et al., 2018 |
|  | **Berberidaceae** | | | |
| 173 | *Berberis aristata* Dc. | S | Berberin, taxilamine, karachine, aromoline, palmatine, oxyberberine, berbamine, pakistanine | Potdar et al., 2012, Bhardwaj and Kaushik, 2012 |
| 174 | *Berberis asiatica* Roxb | S | Berberine, berbamine, tannin, triterpenoids, resins, β- carotene, lycopene, condensed tannins, ascorbic acid | Srivastava, et al., 2004, Bhardwaj and Kaushik, 2012; Pal et al., 2013 |
| 175 | *Berberis chitria Buch.-Ham.ex Lindl.* | S | Berberine, berbamine, O-methylcorydine-N-oxide, pahnatine, jatrorrhizine,oxyacanthine | Hussaini and Shoeb, 1985; Bhardwaj and Kaushik, 2012 |
| 176 | *Berberis lycium* Royle | S | Berberine, berbamine, palmatine, anthocyanin, β-carotene, and ascorbic acid | Khan et al., 2010, Bhardwaj and Kaushik, 2012 |
| 177 | *Berberis osmastonii* Dunn. | S | Berberine, berbamine | Bhardwaj and Kaushik, 2012 |
| 178 | *Berberis pseudoumbellata* Parker | S | Berberine, berbamine | Bhardwaj and Kaushik, 2012 |
| 179 | *Mahonia napaulensis* DC. | S | Protoberberines, bisbenzylisoquinolines | Mai et al., 2014 |
| 180 | *Podophyllum hexandrum* Royle | H | Podophyllotoxin, resin | Giri and Narasu, 2000 |
|  | **Betulaceae** | | | |
| 181 | *Alnus nepalensis* D.Don | T | Terpenoids, flavonoids, diarylheptanoids, phenols, steroids, tannins, oregonin, taraxerone, taraxerol, betulin, betulinic acid, lupeol, β-sitosterol | Sati et al., 2011 |
| 182 | *Betula alnoides* Buch.-Ham. Ex D. Don | T | Methyl salicylate, phenolics, flavonoids | Vikrant and Arya, 2011; Ghimire et al., 2012 |
| 183 | *Betula utilis* D. Don | T | Betulin, lupeol, oleanolic acid, acetyloheanolic acid, betulitc acid, lupenone, sitosterol, methyle betulonate, methyl betulate, triterpenoid, (karachic acid) | Kumaraswamy et al., 2008 |
|  | **Bignoniaceae** | | | |
| 184 | *Jacaranda mimosifolia* D. Don | T | Triterpenes, flavonoids, acetosides, quinones, phenylpropanoid derivatives, fatty acid, anthocyanins | Sidjui et al., 2014 |
| 185 | *Oroxylum indicum* (L.) Vent. | T | Flavonoids (chrysin, oroxylin, baicalein), baicalein glycosides, benzoic acid, fatty acids | Kumar et al., 2007 |
|  | **Bixaceae** | | | |
| 186 | *Bixa orellana* L. | S | Bixin, norbixin, terpenoids, tocotrienols, arenes, Luteolin, apigenin, bisulfates sesquiterpenes, ishwarane, triterpene tomentosic acid | Shilpi et al., 2006 |
|  | **Blechnaceae** | | | |
| 187 | *Blechnum orientale* L. | Fn | Tannins, terpenoids, flavonoids | Lai et al., 2010 |
|  | **Boraginaceae** | | | |
| 188 | *Arnebia benthami* (Don) John. | H | Shikonin | Ganie et al., 2014 |
| 189 | *Arnebia euchroma* (Royle) Johnston | H | Sikonofuran, arnebinol, des-*O* –methyllasiodiplodin | Yao et al., 1991 |
| 190 | *Cordia dichotoma* G. Forst. | T | Pyrrolizidine alkaloids, coumarins, flavonoids, saponins, terpenes, sterols, Arabinoglucan, D-Glucose, L-Arabinose, γ-linolinic acid, Quercetin, quercitrin | Thirupathi et al., 2008; Jamkhande et al., 2013 |
| 191 | *Cordia obliqua* Willd. | T | Hesperetin-7-rhamnoside, lupa-20, 29-ene-3-o- β- D-maltoside, toxifolin-3, 5-dirhamnoside, natural gums, mucilages | Thirupathi et al., 2008 |
| 192 | *Cynoglossum glochidiatum* Wall. ex Benth | H | Phenolic acids, flavonoids | Li et al., 2010 |
| 193 | *Cynoglossum lanceolatum* Forssk. | H | Pyrrolizidine alkaloids, phenolic acids, rosmarinic acid, flavonoids | FU et al., 2002; Li et al., 2010 |
| 194 | *Cynoglossum petiolatum* (Hook) DC. | H | Phenolic acids, flavonoids | Li et al., 2010 |
| 195 | *Cynoglossum zeylanicum* (Vahl ex Hornem.) Thunb. ex Lehmann | H | Pyrrolizidine alkaloids, 9,12-oetadecadienoic acid, n-hexadecanoic acid, borazine,2,4,6-trimethyl, oleic acid, 9,12-octadecadienoyl chloride, isosorbide, ethanamine, N-ethyl-N-nitro, 2- Furancarboxaldehyde, 5-(hydroxyl methyl), phytol | FU et al., 2002; Anitha et al., 2012 |
| 196 | *Ehretia laevis* Roxb. | T | Acontanes, decanoic acids, phthalic acid, phytol, α and β amyrin, piperazine, phenylephrine | Velappan and Thangaraj, 2014 |
| 197 | *Maharanga emodi* (Wall.) DC. | H | Phenolic acids, flavonoids , shikonin derivatives, Pyrrolizidine alkaloids | Roeder and Wiedenfeld, 2009; Li et al., 2010 |
|  | **Botrychiaceae** | | | |
| 198 | *Botrychium ternatum*(Thunb.) Sw. | H | Flavonoid (ternatin) | Wang et al., 2016 |
|  | **Brassicaceae** | | | |
| 199 | Brassica juncea (L.) Czern. | H | Flavonoid (Sorhamnetin diglucoside), Glucosinolates | Fahey et al., 2001; Yokozawa et al., 2002 |
| 200 | *Brassica rapa* L. | H | Phenolics (kaempferol 3-O-sophoroside-7-O-glucoside, kaempferol 3-O-(feruloyl/caffeoyl)-sophoroside-7-O-glucoside, isorhamnetin 3,7-O-diglucoside, isorhamnetin 3-O-glucoside), organic acids (aconitic, citric, ketoglutaric, malic, shikimic, fumaric acids), Glucosinolates | Fahey et al., 2001; Fernandes et al., 2007 |
| 201 | *Cardamine impatiens* L. | H | Glucosinolates, indoles, oxazolidinethiones | Matthäus and Fiebig, 1996 |
| 202 | *Lepidium sativum* L. | H | *β*-caryophyllene, eugenol, eucalyptol, α-terpinyl acetate, (*E*)-anethole | Kimbaris et al., 2012 |
| 203 | *Rorippa nasturtium auuaticum* (L) Hayek | H | Carbohydrates, α- linolenic acid, palmitic acid, linoleic acid | Pereira et al., 2011 |
| 204 | *Raphanus sativus* L. | H | Glucosinolates ( glucoraphasatin, glucoraphenin) | Fahey et al., 2001; Barillari et al., 2005 |
|  | **Buxaceae** | | | |
| 205 | *Sarcococca saligna* (D.Don) Muell.-Arg. | S | Steroidal compounds (saracocine, saracodine, saracorine), alkaloid | Gilani et al., 2005 |
|  | **Cactaceae** | | | |
| 206 | *Opuntia cochenillifera* (L.) Salm-Dyck | S | Isorhamnetinglucoside, querecetrin, kaempferol, rutin, luteolin, galactose, xylose, arabinose, rahmnose, betalain, flavonols, carotenoids, tocopherols, biothiols | Verma et al., 2017 |
|  | **Calophyllaceae** | | | |
| 207 | *Mesua ferrea* L. | T | Trans-caryophyllene, β-caryophyllene oxide, α-humulene, δ-cadinene, γ-muurolene, γ-cadinene, β-selinene, germacrene, β –bisabolene | Keawsa-ard and Kongtaweelert, 2012 |
|  | **Campanulaceae** | | | |
| 208 | *Lobelia pyramidalis* Wall. | H | Perilla ketone, camphorquinone, dibutyl phthalate, allyl nonanoate | Joshi et al., 2011 |
|  | **Cannabaceae** | | | |
| 209 | *Cannabis sativa* L. | H | Cannabinol, cannabidiol, olivetol, γ-1-tetrahydrocannabinol | Formukong et al., 1988 |
| 210 | *Celtis australis* L. | T | Methyl oleate, methyl palmitate, methyl tricosanoate, methyl lineolate, methyl dotriacentanoate, methyl 14-acetylhydroxypalmitate | Badoni et al., 2010 |
|  | **Cannaceae** | | | |
| 211 | *Canna indica* L. | H | Alkaloids, carbohydrates, proteins, flavonoids, terpenoids, cardiac glycosides, steroids, tannins, saponins, anthocyanin pigments, phlobatanins | Al-Snafi, 2015 |
|  | **Capparaceae** | | | |
| 212 | *Crateva adansonii* DC. | T | Phenolics, alkaloids, flavonoids and saponins | Abdullahi et al., 2012 |
|  | **Caprifoliaceae** | | | |
| 213 | *Dipsacus inermis* Wall. Var. | H | Alkaloids, saponins, coumarins, reducing sugars, glycosides, triterpenoid | Shrestha et al., 2015 |
| 214 | *Lonicera hypoleuca* Dcne | S | Anthocyanins | Jurikova et al., 2011 |
| 215 | *Lonicera myrtillus* Hook. F. & Thomson | S | Anthocyanins | Jurikova et al., 2011 |
| 216 | *Nardostachys*  *jatamansi* (D. Don) DC. | H | Sesquiterpene, coumarins, lignans, neolignans, alkaloids, patchoulol, 𝛼-bulnesene, isovaleric acid,𝛼-guaiene,3-methylvaleric acid, sesquiterpenoids, iridoids (valepotriates), flavonoids, lignans | Liu et al., 2013; Purnima and Kothiyal, 2015 |
| 217 | *Valeriana dioica* L. | H | Maaliol, valeranone, kessane, *α*‐kessyl acetate | Mathela et al., 2005 |
| 218 | *Valeriana hardwickii* Wall. ex Roxb. | H | Patchouli alcohol, 8‐acetoxypatchouli alcohol | Mathela et al., 2005 |
|  | **Caricaceae** | | | |
| 219 | *Carica papaya* L. | H | Saponins, cardenolides, alkaloids, tannins, ferulic acid, caffeic acid, rutin, carotenoids | Oloyede, 2005; Rivera‐Pastrana et al., 2010 |
|  | **Caryophyllaceae** | | | |
| 220 | *Drymaria diandra* Blume | H | Phenolics, *O*-dihydric phenols, tannins, flavonols | Mandal et al., 2009 |
| 221 | *Vaccaria* hispanica (Mill.) Rauschert | H | Bisdesmosidic saponins, monodesmosidic saponins, vaccarosides, phenolic vaccarin, cyclopeptide segetalin | Balsevich et al., 2012 |
| 222 | *Oberna* behen (L.) Ikonn. | H | Benzenoids, isoprenoids, lilac compounds | Jürgens et al., 2002 |
| 223 | *Stellaria media* (L.) Vill. | H | saponins, cardiac glycosides, flavonoids, phenolics, terpenoid | Shah et al., 2014 |
|  | **Celastraceae** | | | |
| 224 | *Celastrus paniculatus* Willd. | S | Quinonoid triterpene, pristimerin | Pavanandt et al., 1989 |
| 225 | *Euonymus tingens* Wall. | T | Dihydroagarofuran, sesquiterpene polyol esters, chlorogenic acid, friedooleanans, lupenediol, pyridine alkaloids | Sharma et al., 2012 |
| **Clavicipitaceae** | | | | |
| 226 | *Cordyceps sinensis* (Berk.) Sacc | Fg | Adenosine, guanosine, uridine, polysaccharides | Li et al., 2003 |
| **Cleomaceae** | | | | |
| 227 | *Cleome gynandra* L. | H | Dammarane triterpenoid ( cleogynol), alkaloids, flavonoids, steroids, saponins, carbohydrates, proteins, phenols, glycosides | Narendhirakannan et al., 2005; Bala et al., 2010 |
| 228 | *Cleome viscosa* L. | H | Flavonoids, tannins, alkaloids, aromatic acids, Lactam nonanoic acid | Jana and Biswas, 2011 |
|  | **Colchicaceae** | | | |
| 229 | *Gloriosa superba* L. | H | Colchicine, colchicoside, superbine, gloriosine, lumicolchicine, 3-demethyl-N-deformyl-N-deacetylcolchicine, 3-demethylcolchicine, N-formyl deacetylcolchicine. | Jana and Shekhawat, 2011 |
|  | **Combritaceae** | | | |
| 230 | *Combretum* *indicum* (L.) DeFilipps | S | Rutin, trigonelline, L-proline, laspargine, quisqualic acid, pelargonidin-3-glucoside, linoleic, oleic, palmitic, stearic, arachidic acids, ellagitannins, quisqualin A & B, linalool oxides (furanoid, pyranoid), 2,2,6-trimethyl- 6- vinyl-3-oxo tetrahydropyran, (E,E)-alphafarnesene, (Z)-3-hexenyl benzoate, benzyl benzoate, Diphhenyl propanoids | Yadav et al., 2011 |
| 231 | *Terminalia arjuna* (Roxb) ex. DC | T | Gallic acid, ethyl gallate, luteolin | Pettit et al., 1996 |
| 232 | *Terminalia bellirica* (Gaertn.) Roxb | T | Ermilignan, thannilignan, 7-hydroxy-3‘,4‘-(methylenedioxy)flavan, anolignan B | Valsaraj et al., 1997 |
| 233 | *Terminalia chebula* Retz. | T | Ellagic acid, 2,4-chebulyl-β-d-glucopyranose,chebulinic acid, gallic acid, ethyl gallate, luteolin, tannic acid | Saleem et al., 2002 |
|  | **Commelinaceae** | | | |
| 234 | *Commelina benghalensis* L. | H | Phlobatannins, carbohydrates, tannins, glycosides, volatile oils, resins, balsams, flavonoids, saponins | Ibrahim et al., 2010 |
|  | **Convolvulaceae** | | | |
| 235 | *Argyreia nervosa* Sw. | H | Ergotalkaloid lysergic acid amide | Paulke et al., 2013 |
| 236 | *Cuscuta reflexa* Roxb. | C | Vanillic acid, rutin, quercetin, gallic acid | Tanruean et al., 2017 |
| 237 | *Evolvulus alsinoides* (L.) | H | Piperine, octodeconoic acids, hexadecanoic acid, squalene | Gomathi et al., 2015 |
| 238 | *Volvulopsis* nummularium (L.) Roberty | H | Alkaloids, flavonoids, carbohydrates, glycosides, tannins, terepenoids, cardinolides, anthraquinones, phenolic compounds, | Tamilarasan et al., 2015 |
| 239 | *Ipomoea* fistulosa Mart. ex Choisy | S | Ergoline alkaloids, polyhydroxylated alkaloids, swainsonine, 2-epi-lentiginosine, calystegines B1, B2,B3 & C1, N-methyl-trans-4-hydroxy-L-proline | Haraguchi et al., 2003; Meira et al., 2012 |
| 240 | *Ipomoea nil* (L.)Roth. | S | Ergoline alkaloids, spermidine alkaloid, peonidins, anthocyan HBA, N1, N10-ditigloylspermidine | Meira et al., 2012 |
| 241 | *Ipomoea pestigridis* L. | H | Ergoline alkaloids, flavonoids, fatty acids, mucilage, resins, tannins, astringents, cardiac glycosides, saponins, carbohydrates | Meira et al., 2012,; Sandhya et al., 2013 |
| 242 | *Ipomoea* congesta R. Br. | C | Ipopurpuroside, methylbutyric acid, marubajalapins I-XV, cyanidins, pelargonidins | Meira et al., 2012 |
|  | **Costaceae** | | | |
| 243 | *Costus speciosus* (Koenig. Ex Retz.) J.E. Smith. | H | Alkaloids, flavanoids, phenolic compounds, cardiac glycosides, saponins, sterols, tannins, anthraquinone glycosides | Saraf, 2010 |
|  | **Crassulaceae** | | | |
| 244 | *Kalanchoe* *pinnata* (Lam.) Pers. | H | Alkaloids, triterpenes, glycosides, flavonoids, steroids, bufadienolides, saponin, bryophyllin A & B, anthraquinones, xanthones, lipids, organic acids, | Akinsulire et al., 2007; Kamboj and Saluja, 2009 |
| 245 | *Rhodiola bupleuroides* Wall. ex Hook. f. & Thoms. | H | Salidroside (p-hydroxyphenethyl-bD-glucoside), flavonoids, phenylpropanoids, phenylethanol/benzyl alcohol derivatives, cyanogenic glycosides, terpenoids. | Chen et al., 2008; Li et al., 2017 |
| 246 | *Rhodiola quadrifida* (Pall.) Fisch. & C. A. Mey. | H | Salidroside (p-hydroxyphenethyl-bD-glucoside), flavonoids, phenylpropanoids, phenylethanol/benzyl alcohol derivatives, cyanogenic glycosides, terpenoids. | Chen et al., 2008; et al., 2017 |
|  | **Cucurbitaceae** | | | |
| 247 | *Benincasa hispida* Thunb. | H | Volatile oils , flavonoids, glycosides, sacchrides, proteins, carotenes, ß-sitosterin, uronic acid, triterpenes, phenolics, sterols, glycosides | Zaini et al., 2011; Al-Snafi, 2013 |
| 248 | *Coccinia grandis* (L.) Voigt | H | Heptacosane, cephalandrol, β -sitosterol, alkaloids cephalandrins A & B, β- Amyrin acetate, lupeol, cucurbitacin B, taraxerone, taraxerol, β-carotene, lycopene, cryptoxanthin, xyloglucan, carotenoids, stigma-7-en-3-one, resin, starch, fatty Acids, carbonic acid, triterpenoid, saponin coccinoside, flavonoid Glycoside, β-amyrin, taraxerol | Pekamwar et al., 2013 |
| 249 | *Cucumis sativus* L. | C | Phenolic compounds (p-coumaric acid, caffeic acid, ferulic acid) | Daayf et al., 2000 |
| 250 | *Diplocyclos palmatus* L. | C | Volatile oil, flavonoids, alkaloids, tannins, phenolic compounds, carbohydrates, proteins, amino acids, glycosides, fats & oils, steroids, saponins, triterpenoids | Kadam et al., 2013 |
| 251 | *Luffa acutangula* (L.) Roxb. | C | *p*-coumaric acid, 1-*O*-feruloyl-β-d-glucose, 1-*O*-*p*-coumaroyl-β-d-glucose, 1-*O*-caffeoyl-β-d-glucose, 1-*O*-(4-hydroxybenzoyl)glucose, diosmetin-7-*O*-β-d-glucuronide methyl ester, apigenin-7-*O*-β-d-glucuronide methyl ester, luteolin-7-*O*-β-d-glucuronide methyl ester | Du et al., 2006 |
| 252 | *Solena amplexicaulis* (Lam.) Gandhi | C | Alkaloids, flavonoids, glycosides, saponins, terpenoids, Diterpene (phytol), terpene ( carane ), aliphatic amine (1-octanamine ), 4-(4-ethoxyphenyl) but-3-en-2-one, 9,17-octadecadienal | Karthika et al., 2014; Krishnamoorthy and Subramaniam, 2014 |
| 253 | *Momordica charantia* L. | H | Cucurbitane triterpenoids, 5b,19-epoxy3b,25-dihydroxycucurbita-6, 23(E)-diene, 7b,25-trihydroxycucurbita-5,23(E)-dien-19-al | Harinantenaina et al., 2006 |
| 254 | *Trichosanthes cucumerina* L. | C | Triterpenoid saponins (cucurbitacins) | Kirana and Srinivasan, 2008 |
| 255 | *Trichosanthes tricuspidata* Lour. | C | Carbohydrates, proteins, glycosides and terpenoids, pentacyclic triterpene (trichotetrol) | Kulandaivel et al., 2013 |
|  | **Cupressaceae** | | | |
| 256 | *Cupressus torulosa* D.Don | T | Flavonoids, alkaloids, phenols, saponins, steroids, tannins, terpenoids | Bisht et al., 2016 |
| 257 | *Juniperus communis* L. | S | sesquiterpene (longifolene), diterpenes (totarol, trans-communic acid) | Gordien et al., 2009 |
| 258 | *Juniperus indica* Bartel | S | Amentoflavone, phenols, flavonoids | Bais and Prashar, 2015 |
| 259 | *Juniperus recurva* Buch.-Ham. | S | Podophyllotoxin | Kour et al., 2008 |
| 260 | *Thuja occidentalis* L. | T | Essential oil, reducing sugar, polysaccharides, tannic agents, thujone, isothujone, fenchone, sabines, monoterpenes ( pinen, origanol, carvotanacetone, origanes, myrcene, camphen) | Naser et al., 2005 |
|  | **Cyperaceae** | | | |
| 261 | *Cyperus rotundus* L. | H | Essential oils, flavonoids, terpenoids, mono & sesquiterpenes, coumarins, tannins | Kilani  *et al*., 2008 |
|  | **Dilleniaceae** | | | |
| 262 | *Dillenia indica* L. | T | Betulinic acid, Terpinoids, tannins | Kumar et al., 2010; Savithramma et al., 2011 |
|  | **Dioscoreaceae** | | | |
| 263 | *Dioscorea belophylla* (Prain) Voigt ex Haines | H | Steroidal sapoin (diosgenin) | Njogu, 2007 |
| 264 | *Dioscorea* dodecaneura Vell. | H | Steroid saponins, epicatechin, isovanillic acid, vanillic acid, myricetin | Tang et al., 2006; Sautour et al., 2007 |
| 265 | *Dioscorea deltoidea* Wal.ex Kunth. | H | Steroid saponins (diosgenin) | Vanisree et al., 2004; Sautour et al., 2007 |
| 266 | *Dioscorea kemaonensis* Kunth | H | Steroidal saponin (diosgenin) | Njogu, 2007 |
|  | **Dipterocarpaceae** | | | |
| 267 | *Shorea robusta* Gaertn. | T | Alkaloids, carboxylic acids, fatty acids, phenols, saponins and steroids | Murthy et al., 2011 |
|  | **Elaeagnaceae** | | | |
| 268 | *Elaeagnus parvifolia* Wallich ex Royle | T | Purpurin, tannic acid, quercetin, catechin, reserpine,rutin, carbohydrates, ascorbic acid, tannins | Safeer et al., 2017 |
| 269 | *Hippophae rhamnoides* D. Don | S | Isorhamnetin | Teng et al., 2006 |
| 271 | *Hippophae salicifolia* D. Don | T | Carotenoids, phenolics, flavonoids, sterols | Goyal et al., 2011 |
|  | **Ephederaceae** | | | |
| 271 | *Ephedra gerardiana* Wall. ex Stapf | H | Alkaloids (ephedrine, pseudoephedrine, norpseudoephedrine) | Parsaeimehr et al., 2010 |
|  | **Equisetaceae** | | | |
| 272 | *Equisetum ramosissimum* Desf. | Pter | Glutathione, malonyldialdehyde, superoxide, hydroxyl radicals, flavonoid, soluble protein, carotenoid | Štajner et al., 2009 |
|  | **Ericaceae** | | | |
| 273 | *Gaultheria trichophylla* Royle. | H | Gallic acid, rutin, quercetin. | Alam and Saqib, 2015 |
| 274 | *Lyonia ovalifolia* (Wall.) Drude | T | Lyonin A, secorhodomollolides A & D | Wu et al., 2011 |
| 275 | *Rhododendron* *hypenanthum* Balf. f. | S | Monoterpenes (α-pinene, β- pinene), limonene, sesquiterpene (δ-cadinene) | Innocenti et al., 2010 |
| 276 | *Rhododendron arboreum* Sm. | T | Phenols, saponins, steroids, tannin, xanthoprotein, coumarin | Kiruba et al., 2011 |
| 277 | *Rhododendron campanulatum* D. Don | S | Baccharis oxide, betuligenol, α & β-amyrin, geranyl acetate, (R)-(-)-14-methyl-8-hexadecyn-1-ol, phthalic acid | Painuli et al., 2015 |
|  | **Euphorbiaceae** | | | |
| 278 | *Acalypha indica* L. | H | Tannins, flavanoids and glycosides | Jagatheeswari et al., 2013 |
| 279 | *Baliospermum solanifolium* (Geiseler) Suresh | S | Steroids, triterpenoids, glycosides, saponins, alkaloids, flavanoids, phenolic compounds, tannins, sugars | Johnson et al., 2011 |
| 280 | *Croton bonplandianus* Baill. | S | Diterpenoids (cembranoid, clerodane, neoclerodane, halimane, isopimarane, kaurane, secokaurane, labdane, phorbol, trachylobane, triterpenoids (pentacyclic or steroidal) | Nath et al., 2013 |
| 281 | *Chamaesyce* hirta (L.) Mills | H | Triterpenes (β-amyrin, 24-methylencycloartenol, β-sitosterol), flavonol glycosides (afzelin, quercitrin, myricitrin), diterpenes | Martínez Vázquez et al., 1999; Liu et al., 2007; Vasas and Hohmann, 2014 |
| 282 | *Euphorbia hispida* Boiss. | H | Diterpenes | Vasas and Hohmann, 2014 |
| 283 | *Euphorbia neriifolia* L. | S | Flavonoids, phlobatannins, saponin, tannins, cardenoloids, phenol, terpenoids, diterpenes | Pokharen et al., 2011; Vasas and Hohmann, 2014 |
| 284 | *Euphorbia pilosa* L. | H | Diterpenes | Vasas and Hohmann, 2014 |
| 285 | *Euphorbia prolifera* Buch.-Ham. ex D. Don | H | Myrsinol diterpenes, euphorbiaproliferins | Xu et al., 2011 |
| 286 | *Euphorbia royleana* Boiss. | S | Diterpenes, phenolics, flavonoids | Vasas and Hohmann, 2014; Ashraf et al., 2015 |
| 287 | *Chamaesyce* thymifolia (L.) Millsp. | H | Diterpenes | Vasas and Hohmann, 2014 |
| 288 | *Excoecaria acerifolia* F. Diedr. | H | 3α,18-dihydroxy-3β,20- epoxykaur-15-ene, 3β,20-epoxy-3α,6α-dihydroxy-18-norbeyer-15-ene, catechin, kaempferol, quercetin, 5,7-dihydroxy-3,4'- dimethoxyflavone, aromadendrin, texifolin, 6-dimethoxy-7- dihydroxycoumarin, trihydroxybenzoic acid, progallin A, shikimic acid, (E)-p-coumatic acid, m-hydroxybenzoic acid Me ester, 3,4-dihydroxy-benzoic acid, phytol | Zhao et al., 2010 |
| 289 | *Jatropha curcas* L. | S | Saponin, steroids, tannin, glycosides, alkaloids, flavonoids | Igbinosa et al., 2009 |
| 290 | *Mallotus philippensis* (Lam.) Muell.- Arg. | T | Amino acids,  carbohydrates,  flavonoids,  gum, oil  &  resins,  proteins,  phenolic  groups,  saponins,  steroids,  tannins, terpenoids. | Velanganni et al., 2011 |
| 291 | *Ricinus communis* L. | S | Flavonoids, saponins, glycosides, alkaloids, steroids | Jena and Gupta, 2012 |
| 292 | *Trewia nudiflora* L. | T | 17‐hydroxy‐ent‐atisan‐19‐oic acid, 17‐hydroxy‐ent‐atisan‐19‐oic acid methyl ester, and 16α,17‐dihydroxy‐ent‐atisan‐19‐al, gallic acid, ethyl gallate, protocatechuic acid, 3,4,4′‐tri‐O‐methylellagic acid, α‐tocopherol, and trans‐cinnamic acid, taraxerone, | Du et al., 2004 |
|  | **Fabaceae** | | | |
| 293 | *Abrus precatorius* L. | C | Abrusogenin, monoglyceride, triglyceride, β-sitosterol, stigmasterol | Ragasa et al., 2013 |
| 294 | *Acacia catechu* (L.f.) | T | Catechuic acid, catechutannic acid, acacatechin, catechu red, quercetin, catechin, epicatechin, phlabotannin, quercitrin, fisetin, cyanodol, tannins, polyphenols | Rahmatullah et al., 2013 |
| 295 | *Albizia amara* (Roxb.) B. Boiv. | T | Alkaloid (budmunchiamines), flavonoids, steroids, phenols, tannins | Rajkumar and Sinha, 2010 |
| 296 | *Albizia* lebbekoides (DC.) Benth. | T | Budmunchiamines, quercetin, kaempferol, 3-O-α-rhamnopyranosyl (1→6)-β-glucopyranosyl(1→6)-β-galactopyranosides, Albiziasaponins | Kokila et al., 2013 |
| 297 | *Albizia procera* (Roxb.) Benth. | T | Saponins, steroids, tannins, glycosides, flavonoids | Islam et al., 2013 |
| 298 | *Alysicarpus vaginalis* (L.) DC | H | Saponins, alkaloids, flavonoids, phenols, quinines, terpenoids | Kurian et al., 2017 |
| 299 | *Caragana* aegacanthoides (R. Parker) L.B.Chaudhary & S.K. Srivast. | H | Triterpenoid saponins (astragalosides), gum (tragacanth), polysaccharides, saponins and phenolics | Rios and Waterman 1997; Golmohammadi, 2013 |
| 300 | *Astragalus* canadensis L. | H | Triterpenoid sapponins (astragalosides), gum (tragacanth), polysaccharides, saponins and phenolics | Rios and Waterman 1997; Golmohammadi, 2013 |
| 301 | *Astragalus chlorostachys* Lindley | H | Triterpenoid sapponins (astragalosides), gum (tragacanth), polysaccharides, saponins and phenolics | Rios and Waterman 1997; Golmohammadi, 2013 |
| 302 | *Bauhinia purpurea* L. | T | Phenolics, flavonoids, saponins, glycosides, tannins | Sharanabasappa et al., 2007 |
| 303 | *Bauhinia vahlii* (Wt.& Arn.) Benth. | C | Phenolics, tannins, flavonoids | Sowndhararajan and Kang, 2013 |
| 304 | *Bauhinia variegata* L. | T | Tannins, alkaloids, saponins, oil, fat glycoside, carbohydrates, Phenolics, lignin, flavonoids, terpinoids | Parekh et al., 2006; Dhale , 2011 |
| 305 | *Butea minor* Ham. | S | Flavonoids (Butein, Butrin, Isobutrin, Plastron, coreipsin, Isocoreipsin) | Rana and Avijit, 2012 |
| 306 | *Butea monosperma* (Lam.) | T | Alkaloids, flavonoids ( Butein, Butrin, Isobutrin, Plastron, coreipsin, Isocoreipsin), diterpenoids, tannins, terpeniods, carbohydrate | Rana and Avijit, 2012; Thooyavan and Karthikeyan, 2016 |
| 307 | *Cajanus cajan* L. | S | Steroids, terpens, flavonoids, saponins, hexadecanoic acid methyl ester, α-amyrin, β -sitosterol, pinostrobin, longistylins A & C | Tona et al., 1998; Ashidi et al., 2010 |
| 308 | *Cajanus scarabaeoides* (L.) Thouars | H | Alkaloids, glycosides, flavonoids, steroids | Pattanayak et al., 2009 |
| 309 | *Cassia auriculata* L. | H | Sterols, triterpenoids, flavonoids, tannins, lavonoids, terpenoids, phenols, amino acids, essential oil, proteins | Jobitha et al., 2012; Makheswari et al., 2012 |
| 310 | *Cassia fistula* L. | T | Tannins, flavonoids, glycosides, phenolic compounds, saponins, triterpenoids, steroids, anthraquinones, reducing sugars, amino acids, | Voon et al., 2012 |
| 311 | *Cicer arietinum* L. | H | Tannins, flavonoids, phenolics | Nithiyanantham et al., 2012 |
| 312 | *Clitoria ternatea* L. | H | Triterpenoids (taraxerol, taraxerone) | Kumar et al., 2008 |
| 313 | *Crotalaria juncea* L. | H | Alkaloids, dehydropyrrolizidine alkaloid diesters, junceine, trichodesmine | Colegate et al., 2012 |
| 314 | *Crotalaria retusa* L. | H | Alkaloids, saponins, tannins, cardiac glycosides, steroids, flavonoids, anthocyanins | Mpiana et al., 2009; Dhole et al., 2011 |
| 315 | *Crotalaria verrucosa* L. | H | Flavonoids, phenolic compounds, alkaloids, tannin, steroids, glycosides, anacrotine, crotaverrine, O^12^-acetyl-crotaverine | Roeder and Wiedenfeld, 2013; Ahmed, 2016 |
| 316 | *Dalbergia sissoo* Roxb. | T | Isoflavones, flavones, flavonols, neoflavones, coumarins. | Dixit et al., 2012 |
| 317 | *Desmodium elegans* DC | S | Flavonoids, alkaloids, terpenoids, steroids, phenols, phenylpropanoids, glycosides, volatile oils | Ma et al., 2011 |
| 318 | *Desmodium gangeticum* (L.) DC | S | Polyphenol, flavonoid, flavonols, alkaloids, pterocarpnoid, flavones, isoflavanoid glycosides | Govindarajan et al., 2003; Tsai et al., 2011 |
| 319 | *Desmodium heterocarpon* (L.)DC. | H | Polyphenol, flavonoid, flavonol | Tsai et al., 2011 |
| 320 | *Desmodium oojeinense* (Roxb.) H. Ohashi | H | Polyphenol, flavonoid, flavonol , alkaloids, astragalin, cosmossiin, tectorigenin, 2-O-glucosylvitexin, 2-O-β- xylosylvitexin, vitexin, genistin, aliphatic alcohols, aliphatic acids, ursolic acid, oleanolic acid, campesterol, stigmasterol, β-sitosterol, campesterol-3-O-β-D-glucose, stigmasterol3-O-β-D-glucose, sitosterol-3-O-β-D-glucose, (+)-pinitol, | Lai et al., 2010; Tsai et al., 2011 |
| 321 | *Erythrina variegata* L. | T | Alkaloids, flavonoids, erythrabyssin II, dihydrofolinin, octacosyl ferulate, wax alcohol, wax acids, alkyl ferulates, alkyl phenolates | Kumar et al., 2010 |
| 322 | *Flemingia strobilifera* (L.)W.T. Aiton | H | Flavonoids, flavonoid glycosides, chalcones, epoxychromenes, pterocarpans | Madan et al., 2013 |
| 323 | *Glycine max* (L.) Merr. | H | Soyasaponins, β‐sitosterol, lecithin | Bau et al., 2000; Rupasinghe et al., 2003 |
| 324 | *Glycyrrhiza glabra* L. | H | Glycyrrhizin, glabridin, glabrene, glabrol, licoflavonol, glycyrol, licoricone, formononetin, phaseollinisoflavan, hispaglabridin, 3-hydroxy glabrol, 3 -methoxy glabridin, glabranin isomer, narigenin, lupiwightenone | Gupta et al., 2008 |
| 325 | *Indigofera cassioides* Rottl. ex DC. | S | Glucoside indicant | Chanayath et al., 2002 |
| 326 | *Indigofera heterantha* Wall. ex Brandis | S | Glucoside indican, alkaloids, phenols, polyphenols, saponins, tannins, anthraquinones, steroids, diterpenes | Chanayath et al., 2002 |
| 327 | *Indigofera hirsuta* L. | S | Glucoside indicant | Chanayath et al., 2002; Arfan et al., 2011 |
| 328 | *Indigofera linifolia* (L.f.) Retz. | H | Glucoside indicant | Chanayath et al., 2002 |
| 329 | *Indigofera* suffruticosa Mill. | S | Glucoside indican, indirubin, carbohydrates, glycosides, alkaloids, tannins, flavanoids, steroid | Chanayath et al., 2002; Balamurugan and Selvarajan, 2009 |
| 330 | *Macrotyloma uniflorum* (Lamk) Verd. | H | Carbohydrates, proteins, amino acids, steroids, triterpenoids, glycosides, flavonoids, polyphenols, tannins, alkaloids | Ramesh et al., 2011 |
| 331 | *Medicago* nigra (L.) Krock. | H | Triterpene saponins, sugars | Tava et al., 2011 |
| 332 | *Melilotus albus* Medik. | H | Coumarins, triterpene saponins, total phenols, flavonoids, proanthocyanidins | Stefanović et al., 2015 |
| 333 | *Millettia extensa* (Benth.) Baker | S | Lupenone, lupeol, stigmastenone, palmitic acid, daidzein dimethylether, formononetin, afromorsin, secundiferol, 2′-hydroxyformononetin, pisatin, flemichapparin B, dihydrocoumestrol dimethyl ether, variabilin | Zingue et al., 2016 |
| 334 | *Stizolobium* pruriens (L.) Medik. | S | Alkaloids (mucunine, mucunadine, prurienidine, nicotine), β-sitosterol, glutathione, lecithin, vernolic acid, gallic acid. | Ahmad et al., 2008 |
| 335 | *Ougeinia oojeinensis* (Roxb.) Hochr. | T | Flavonoids, alkaloids, saponin, steroids, tannins, triterpenoids | Velmurugan et al., 2011; Samyal et al., 2008 |
| 336 | *Phaseolus vulgaris* L. | H | Monoterpenoids (*p*-cymene, α-pinene, camphor, linalool, terpineol, cuminaldehyde, cinnamaldehyde, anethole, carvacrol, thymol, estragole, eugenol), phenolics | Regnault-Roger and Hamraoui, 1995; Rocha-Guzmán et al., 2007 |
| 337 | *Derris* indica (Lam.) Bennet | T | Flavonoids, karanjin, pongapin, pongaglabrone, kanugin, desmethoxykanugin, pinnatin, furanoflavonoid glucosides, flavonol glucoside | Sikarwar and Patil, 2010 |
| 338 | *Pterocarpus marsupium Roxb.* | T | Tannins, saponins, 3-0-methyl-d-glucose, n-Hexadecanoic acid, 1,2-Benzene-dicarboxylic acid, diisooctyl ester, tetradecanoic acid, 9,12 – octadecadienoic acid (Z,Z), D-Friedoolean -14-en-3-one, lupeal. | Gayathri and Kannabiran 2009; Maruthupandian and Mohan, 2011 |
| 339 | *Pueraria tuberose* (Roxb.ex Willd.) DC. | S | Flavones, isoflavones (Puerarone), coumstan (tuberostan, puerarostan), epoxychalcanol pterocarpanoids,tuberosin | Pandey and Tripathi, 2010 |
| 340 | *Senna occidentalis* L. | H | Anthraquinones, carbohydrates, glycosides, cardiac glycosides, steroids, flavanoids, saponins, phytosterols, gums, mucilages | Arya et al., 2010 |
| 341 | *Senna tora* L. | S | Anthraquinones, chrysophanol, emodin, rhein | Wu and Yen; 2004 |
| 342 | *Tephrosia purpuria* Pers. | H | Isoflavone (7,4‘-dihydroxy-3‘,5‘-dimethoxyisoflavone chalcone, purpurin, pongamol, lanceolatin B, maackiain, 3-hydroxy-4-methoxy-8,9-methylenedioxypterocarpan, medicarpin | Chang et al., 1997 |
| 343 | *Trifolium repens* L. | H | Isoflavone, biochanin A | Cassady et al., 1988 |
| 344 | *Trigonella foenum-graecum* L. | H | Steroids ( sapogenin), N-compounds, polyphenolic substances, volatile constituents, amino acids, diosgenin, trigonelline, fenugreekine, galactomannan and 4-hydroxy isoleucine | Skaltsa, 2003; Zandi et al., 2017 |
| 345 | *Vigna mungo* (L.) Hepper | H | Phytic acid, p-coumaric acid | Duhan et al., 1989; Kojima and Takeuchi, 1989 |
| 346 | *Saraca asoca* (Roxb.) Willd. | T | Carbohydrates, tannin, flavonoid, saponin, glycosides, proteins, steroids | Saha et al., 2012; Sharma and Patel, 2009; Gomashe et al., 2014 |
| 347 | *Tamarindus indica* L. | T | Phenolic compounds, cardiac glycosides, mallic acid, tartaric acid, mucilage, pectin, arabinose, xylose, galactose, glucose, uronic acid | Bhadoriya et al., 2011 |
| 348 | *Vachellia* *nilotica* (L.) P.J.H. Hurter & Mabb. | T | Alkaloids, volatile essential oils, glycosides, resins, oleoresins, steroids, tannins, terpenes, phenols | Malviya et al., 2011 |
| 349 | *Leucaena* leucocephala (Lam.) de Wit | T | Flavonoid (quercetin), 2(H)-benzofuranone5,6,7,7a-tetrahydro-4,4,7a-trimethyl, pentadecanoic acid-14-methyl-methyl ester | Salem et al., 2011 |
| 350 | *Mimosa* *balansae* Micheli | H | Terpenoids, flavonoids, glycosides, alkaloids, quinines, phenols, tannins, saponins, coumarin | Gandhiraja et al., 2009 |
| 351 | *Pithecellobium dulce* (Roxb.) Benth. | H | Flavonoids, saponins, phenolics, steroids | Manna et al., 2011 |
|  | **Gentianaceae** | | | |
| 352 | *Gentiana albicalyx* Burkill | H | Iridoids, triterpenoids, flavonoids, alkaloids | Shabir et al., 2017 |
| 353 | *Gentiana stipitata* Edgew. | H | Iridoids, triterpenoids, flavonoids, alkaloids | Shabir et al., 2017 |
| 354 | *Gentiana tubiflora* (G. Don) Griseb. | H | Iridoids, triterpenoids, flavonoids, alkaloids | Shabir et al., 2017 |
| 355 | *Hoppea dichotoma* Willd. | H | Glucosyloxyflavans ( diffutin), dichotosin and dichotosinin | Ghosal et al.,1985 |
| 356 | *Swertia alata* (Royale ex D. Don) C.B.Clarke | H | Flavonoids, iridoid glycosides, triterpenoids  swertisin, sertiamarin, beldifolin | Dutt et al., 1996; Negi et al., 2011 |
| 357 | *Swertia angustifolia* Buch.-Ham. ex D. Don | H | Flavonoids, iridoid glycosides, triterpenoids, etroxygenated, *P*-oxygenated Xanthones, *O*-glucosides. | Dutt et al., 1996 |
| 358 | *Swertia chirayita* Roxb. ex Flem | H | Flavonoids, iridoid glycosides and triterpenoids  ophelic acid, chiratin, amarogentin, gentiocrucine, enicoflavin, palmitic acid, swerchirine, gentianine, oleic acid, stearic acid, phytosterol | Dutt et al., 1996 |
| 359 | *Swertia ciliata* Burtt. | H | Xanthones, flavonoids, iridoid glycosides, triterpenoids, mangiferin, amaroswerin and amarogentin | Dutt et al., 1996; Chauhan and Dutt, 2013 |
| 360 | *Swertia cuneata* D. Don | H | Flavonoids, iridoid glycosides, triterpenoids | Dutt et al., 1996 |
|  | **Geraniaceae** | | | |
| 361 | *Geranium nepalense* Sw. | H | Flavonoids (kaempferol, kaempferol7-O-β-D-glucopyranoside, quercetin-7-O-α- rhamnopyranoside), tannins(pyrogallol, gallic acid), lignan(epipinoresinol) | Lu et al., 2012 |
| 362 | *Geranium polyanthes* Edgew. & Hook. f. | H | Tannins | Bautista et al., 2015 |
| 363 | *Geranium ocellatum* Cambess. | H | Tannins | Bautista et al., 2015 |
| 364 | *Geranium* wallichianum D.Don ex Sweet | H | Tannins, ursolic acid, β-sitosterol, stigmasterol, β-sitosterol galactoside, herniarin, 2, 4, 6-trihydroxyethylbenzoate | Bautista et al., 2015 |
|  | **Gesneriaceae** | | | |
| 365 | *Didymocarpus pedicellatus* R. Br. | H | Chalcones (pashanone), polyterpenes, (didymocarpol,didymacarpenol), flavonoids, dicarboxylic acid, essential oils, didymocarpene | Singh, 2007 |
|  | **Grossulariaceae** | | | |
| 366 | *Ribes alpestre* Wall ex. Decne | H | Anthocyanins and flavonols | Brennan, 2008 |
|  | **Hypericaceae** | | | |
| 367 | *Hypericum elodeoides* Choisy | H | Meroterpenoids, xanthones, benzophenone glycosides, chromones, polyprenylated acylphloroglucinols, polyprenylated benzophenones (hypelodins A & B) | Tanaka et al., 2016 |
| 368 | *Hypericum oblongifolium* Choisy | S | Flavone (folicitin), lactone (folenolide), xanthones (hypericorin C &D, 3,4-dihydroxy-5-methoxyxanthone) 2,3-dimethoxyxanthone, 3,4-dihydroxy-2-methoxyxanthone, 3,5-dihydroxy-1-methoxyxanthone,3-acetylbetulinic acid, 10*H*-1,3-dioxolo[4,5-*b*]xanthen-10-one, 3-hydroxy-2-methoxyxanthone, 3,4,5-trihydroxyxanthone,betulinic acid | Ali et al., 2014; Raziq et al., 2015 |
| 369 | *Hypericum japonicum* Thunb.ex Murray | H | Flavonoids ( quercitrin, isoquercitrin) | Li et al., 2008 |
| 370 | *Hypericum uralum* Buch.-Ham. ex D. Don | S | Hyperuralones, 1,9-seco-bicyclic polyprenylated acylphloroglucinols, bicyclo[3.3.1]nonane-2,4,9-trione core, attenuatumione B | Zhang et al., 2015 |
|  | **Hypoxidaceae** | | | |
| 371 | *Curculigo orchioides* Gaerth | H | Flavones, glycosides, steroids, saponins, triterpenoids, 2,6-dimethoxy benzoic acid, curculigoside A & B,Curculigine A & D,3,3′,5,5′-tetramethoxy-7,9′:7′,9-diepoxylignan-4,4′-di-*O*-β-d-glucopyranoside | Jiao et al., 2009; Nagesh and Shanthamma, 2009 |
|  | **Iridaceae** | | | |
| 372 | *Iris kumaonensis* Wall. ex Don | H | Irisoquin, isoflavones (tectoregenin, iristectorin iriginen, irisdin, iris kumonin) | Wani et al., 2012 |
|  | **Juglandaceae** | | | |
| 373 | *Juglans regia* L. | T | Tannins, phenolics, linoleic acid,y oleic acid, linolenic acid, palmitic acid | Pereira et al., 2008; Zhang et al., 2009 |
|  | **Lamiaceae** | | | |
| 374 | *Ajuga bracteosa* Wall.ex Benth. | H | Cyanogenic glycosides, glucosinolates, terpenes, saponins, tannins, anthraquinones, polyacetylenes, limonene, α-humulene, β-myrcene, elemol, camphene, β- caryophellene, α-phellendrene. | Vohra and Kaur, 2011 |
| 375 | *Ajuga macrosperma* Wall. ex Benth. | H | Jugacetalsterones C & D, breviflorasterone,, 20-hydroxyecdysone, cyasterone, makisterone A, 20-hydroxyecdysone 3-acetate, 20-hydroxyecdysone 2-acetate. | Laekeman and Vlietinck, 2013 |
| 376 | *Ajuga parviflora* Benth. | H | Carbohydrates, glycosides, tannins, alkaloids, polyphenols, quinines, dions, aminophenols, steroids, sterols, flavonoids, terpenoids | Rahman et al., 2013 |
| 377 | *Anisochilus carnosus* (L.f.) Wall. ex Benth. | H | Flavonoids (luteolin, apigenin), phytosterols, triterpenoids, saponins, tannins, essential oil components (carvacrol, α-cis-bergamotene, caryophyllene, β-selinene, camphor) | Bhagat et al., 2014 |
| 378 | *Anisomeles indica* (L.) Kuntze | H | Acteoside,isoacteoside, cistanoside F, campneoside II, flavonoids (apigenin,apigenin 7-O-glucoronide,terniflorin),sterol (β-sitosterol, β-sitosterol 3-O-b-Dglucoside), phenyl propanoids calceolarioside, betonyoside A | Rao et al., 2012 |
| 379 | *Callicarpa arborea* Roxb. | H | Terpenoids, flavonoids | Tu et al., 2013 |
| 380 | *Callicarpa macrophylla* Vahl | S | Terpenoids, flavonoids, calliterpenone, calliterpenone monoacetate | Verma et al., 2009; Tu et al., 2013 |
| 381 | *Clerodendrum cordatum* D. Don. | S | Steroids, terpenoids, iridoids, flavonoids | Shrivastava and Patel, 2007 |
| 382 | *Clerodendrum serratum* (L.) Moon | H | β-sitosterol, 24(S)-ethylcholesta-5, 25-trien-3 β-ol, 5-hydroxy-7,4'-dimethoxy flavone, luteolin, apigenin, scutellarien, ursolic acid, steroids, terpenoids, iridoids, flavonoids | Shrivastava and Patel, 2007; Bhujbal et al., 2009 |
| 383 | *Colebrookea oppositifolia* Smith | S | Alkaloids, glycosides, flavonoids, sterols, triterpenoids, tannins. | Ishtiaq et al., 2016 |
| 384 | *Plectranthus* *amboini*cus (Lour.) Spreng. | H | Essential oil components thymol, 1,8-cineole, p-cymene, spathulenol, terpinen-4-ol | Singh et al., 2002 |
| 385 | *Coleus* *forskohlii* (Willd.) Briq. | H | Taxol, vincristine, vinblastine, colchicines, forskolin | Kavitha et al., 2010 |
| 386 | *Colquhounia coccinea* Wall. | S | Alkaloids, glycosides, terpenoids, steroids, flavonoids, tannins | Chhetri et al., 2008 |
| 387 | *Elsholtzia flava* Benth. | S | Flavonoids, phenylpropanoids, terpenoids, phytosterols, cyanogenic glycosides | Guo et al., 2012 |
| 388 | *Elsholtzia fruticosa* (D. Don) Rehder. | S | Flavonoids, phenylpropanoids, terpenoids, phytosterols, cyanogenic glycosides | Guo et al., 2012 |
| 389 | *Elsholtzia strobilifera* Benth. | H | Flavonoids, phenylpropanoids, terpenoids, phytosterols, cyanogenic glycosides | Guo *et al.*, 2012 |
| 390 | *Mesosphaerum* suaveolens (L.) Kuntze | H | Alkaloids, tannins, saponins, flavonoids, phenols | Edeoga et al., 2006 |
| 391 | *Lamium album* L. | H | Phenolic acids, iridoids, flavonoides, triterpenes, fatty acids, polysaccharides, saponines, phytoecdysteroids, amines, essential oils, tannins, mucilage | Paduch et al., 2008 |
| 392 | *Leonotis nepetifolia* Br. | H | Iridoid glycosides, phenylethanoid glycosides, labdanoid diterpenoid, coumarins, laballenic acid, allenic acid, saponins, flavonoids, phenolics, tannins, carbohydrates & proteins, | Trivedi et al., 2011 |
| 393 | *Leucas cephalotes* (Roth) Spreng. | H | Glycoside β-sitosterol, iridoid glycosides,laballenic ester, lignans, flavonoids, coumarins, steroids, terpenes, fatty acids | Parveen et al., 2002; Chouhan and Singh, 2011 |
| 394 | *Leucus indica* (L.) R. Br. Ex Vatke | H | Lignans, flavonoids, coumarins, steroids, terpenes, fatty acids , triterpene (leucolactone), sterols (sitosterol, campesterol, stigmasterol) phenolic compound | Chouhan and Singh, 2011; Divya et al., 2014 |
| 395 | *Leucas lanata* Benth. | H | Lignans, flavonoids, coumarins, steroids, terpenes, fatty acids, gallic acid, protocatechuic acid, chlorogenic acid, caffeic acid,ferulic acid | Chouhan and Singh, 2011; Dixit et al., 2015 |
| 396 | *Leucas mollissima* Wall. ex Benth. | H | Lignans, flavonoids, coumarins, steroids, terpenes, fatty acids, (−)epi-marmelo lactone, (2 S, 4R, 6 S)-2,6-dimethyl-6 hydroxy-7-ene-4-olide, schensianol A, anillin, β-hydroxy propiovanillone,lanost-9,25-diene3β,24β-diol, lanost-9(11),23E(24)-diene-3β,25-diol | Chouhan and Singh, 2011; Chinchansure et al., 2015 |
| 397 | *Mentha* *canadensis* L. | H | Menthol, L-methone, isomenthone, menthyl acetate | Naeem et al., 2011 |
| 398 | *Mentha longofolia* L. | H | Menthol, isomenthol, neomenthol, neoisomenthol | Al-Bayati, 2009 |
| 399 | *Mentha piperita* L. | H | Menthol, menthone | İşcan et al., 2002 |
| 400 | *Mentha spicata* L. | H | Arvone, *cis*-carveol, limonene | Govindarajan et al., 2012 |
| 401 | *Satureja* *biflora* (Buch.-Ham. ex D. Don) Briq. | H | Thymol, iso thymol, gurjurene,β-caryophyllene | Mishra et al., 2010 |
| 402 | *Mosla dianthera* (Buch.-Ham. ex Roxb.) Maxim. | H | Elemicin, thymol, β-caryophyllene, iso-elemicin, asarone, α-caryophyllene | Wu et al., 2012 |
| 403 | *Nepeta ciliaris* Wall. ex Benth. | H | Monoterpenoids, sesquiterpene hydrocarbons sesquiterpenoids, diterpene hydrocarbons, α-ylangene, β-caryophyllene, guaiacol, α-humulene, isolongifolene, trans-α-bergamotene, β-cyclocitral | Gautam et al., 2016 |
| 404 | *Nepeta discolor* Royle ex Benth. | H | 1,8-Cineole, β-Caryophyllene, p-Cymene | Bisht et al., 2010 |
| 405 | *Nepeta elliptica* Royle ex Benth. | H | (7R)-trans,trans-Nepetalactone, isoiridomyrmecin | Bisht et al., 2010 |
| 406 | *Nepeta eriostachya* Benth. | H | Terpenoid, terpenes (mono, sesquiterpenes) | Sharma et al., 2013 |
| 407 | *Nepeta graciliflora* Benth. | H | Terpenoid, terpenes (mono, sesquiterpenes) | Sharma et al., 2013 |
| 408 | *Nepeta hindostana* (Roth.) Haines. | H | Sesquiterpene hydrocarbons, β- farnesene ageratochromene, β –caryophyllene, spiro [4.5] decan-6-ol, 6-methyl | Pandey et al., 2015 |
| 409 | *Nepeta leucophylla* Benth. | H | Iridodial β-monoenol acetate, dihydroiridodial diacetate, iridodial dienol diacetate | Bisht et al., 2010 |
| 410 | *Ocimum americanum* L. | H | α-pinene, β-pinene, myrcene, α-terpinene, limonene, 1,8-cineole, camphor, linalool, linalyl acetate, farnesene, β-isabolene, methyl chavicol, terpineol, methyl eugenol, eugenol, iso eugenol, farnesol | Shadia et al., 2007 |
| 411 | *Ocimum basilicum* L. | H | Linalool, epi-α-cadinol, α-bergamotene, cadinene, germacrene D, camphor | Hussain et al., 2008 |
| 412 | *Ocimum gratissimum* L. | S | Monoterpenes (eugenol, methyl eugenol, cimene, trans-ocimene, β-pinene, camphor, sesquiterpenes (germacrene D,trans-caryophyllene) | Matasyoh et al., 2007 |
| 413 | *Ocimum kilimandscharicum* Guerke | H | Camphor, β-myrcene, α-phellandrene, α-terpinene, limonene, 1,8-cineole, γ-terpinene, δ-3-Carene *p*-Cymene, α-Terpinolene, terpinen-4-ol, α-terpineol, germacrene D | Carović-Stanko et al., 2010 |
| 414 | *Ocimum tenuiflorum* L. | H | Eugenol, methyl eugenol, terpinolene, germacrene D | Joshi, 2013 |
| 415 | *Origanum vulgare* L. | H | Tricyclene, α-thujene, α- camphene, sabinene, β-pinene 1-octen-3-ol, octan-3-one, myrcene, δ^2^ -carene, α-phellandrene, δ^3^-carene | De Martino et al., 2009 |
| 416 | *Premna barbata* Wall.ex Schauer. | T | 1-octen-3-ol, epi-α-bisabolol, Flavanoids, terpenoids, alkaloids, polysaccharides | Chanotiya et al., 2009; Kabra et al., 2015 |
| 417 | *Premna latifolia* Roxb | T | Iridoids, glycosides, diterpenes, saponins, alkaloids, carbohydrates, amino acids, steroids, flavonoids, tannins, phenolic compounds | Mali and Bhadane, 2010; Kabra et al., 2015 |
| 418 | *Perilla frutescens* (L.) Britton | H | luteolin 7-O-[β-glucuronosyl(2→1)β-glucuronide], apigenin 7-O-[β-glucuronosyl(2→1)b-glucuronide], scutellarin, rosmarinic acid | Makino et al., 2001 |
| 419 | *Isodon* rugosus (Wall. ex Benth.) Codd | S | Spatulenol, germacene D, β-caryophyllene | Irshad et al., 2012 |
| 420 | *Prunella vulgaris* L. | H | Phenols (caffeic acid, rosmarinic acid, rutin, quercetin) | Feng et al., 2010 |
| 421 | *Rosemarinus officinalis* L. | H | Phenolic diterpenes (carnosic acid, carnosol, 12-*O*-methylcarnosic acid), caffeoyl derivatives (rosmarinic acid), flavones (isoscutellarein 7-*O*-glucoside, genkwanin) | del Baño et al., 2003 |
| 422 | *Roylea cinerea* D.Don | H | 4-methoxybenzo[b]azet-2(1H)-one, 3β-hydroxy-35-(cyclohexyl-5′-propan-7′-one)-33-ethyl-34-methyl-bacteriohop-16-ene, stigmasterol, β-sitosterol | Bhatt et al., 2018 |
| 423 | *Salvia hians* Royle ex Benth. | H | β-pinene, limonene, β -trans-ocimene, eucalyptol, thujone, bornyl acetate, β –chamigrene, cadin-3,9 –diene, murolene, isocaryophyllene | Sajewicz et al., 2009 |
| 424 | *Salvia longifolia* Nutt. | H | Diterpenoids, phenolics, monoterpenes | Jash et al., 2016 |
| 425 | *Salvia nubicola* Wallich ex Sweet | H | Sesquiterpene lactones (nubiol, bisnubidiol) | Ali et al., 2007 |
| 426 | *Salvia* cana Wall. ex Benth. | H | Diterpenoids, phenolics, monoterpenes | Jash et al., 2016 |
| 427 | *Salvia moorcroftiana* Wall. ex Benth. | H | Diterpenoids, phenolics, monoterpenes, flavonoids | Ahmad et al., 2000; Jash et al., 2016 |
| 428 | *Salvia plebeia* R. Br. | H | Hispidulin-glucuronide,hispidulin-7-*O*-d-glucoside,6-methoxy-luteolin-7-glucoside, β-sitosterol, 2′-hydroxy-5′-methoxybiochanin A, coniferyl aldehyde | Weng and Wang, 2000 |
| 429 | *Scutellaria scandans* Buch.Ham. ex D. Don | H | Flavonoids (Baicalein, Baicalin, chrysin, wogonin) | Parajuli et al., 2009 |
| 430 | *Tectona grandis* L. | T | Lapachol, tectoquinone, desoxylapachol, isodesoxylapachol, squalene, tectol, palmitic acid | Lukmandaru and Takahashi, 2009 |
| 431 | *Thymus linearis* Benth. | H | Thymol, carvacrol, thymyl acetate, β-caryophyllene | Hussain et al., 2013 |
| 432 | *Vitex negundo* L. | S | Alkaloid, flavonoid, carbohydrates, glycosides, proteins, amino acids, steroids | Thatoi and Dutta, 2009 |
|  | **Lauraceae** | | | |
| 433 | *Cinnamomum tamala* Nees & Eberm. | T | Mono- & sesqui-terpenes (pinene, phellandrene, linalool, geraniol) | El-Baroty et al., 2010 |
| 434 | *Cinnamomum* verum J. Presl | T | Flavonoids, isoflavones, flavones, anthocyanin, catechin, trans-cinnamaldehyde | Jayaprakasha et al., 2007; El-Baroty et al., 2010 |
| 435 | *Lindera pulcherrima* (Nees) Benth. | T | Sesquiterpenoids (furanosesquiterpenoids, furanodienone, curzerenone, furanodiene, isofuranogermacrene) | Joshi and Mathela, 2012 |
| 436 | *Litsea glutinosa* (Lour.) Robinson | T | Flavonoids, terpenoids Steroids, Carbohydrate | Haque et al., 2014 |
| 437 | *Litsea monopetala* (Roxb.) Pers. | T | Alkaloids, carbohydrates, tannins, flavonoids, steroids | Hasan et al., 2016 |
| 438 | *Neolitsea pallens* (D. Don) Moniyama & Hara | T | Furanosesquiterpenoids ( furanodienone, furanogermenone, curzerenone, isofuranogermacrene, furanodiene) | Joshi, 2014 |
| 439 | *Persea duthiei* King ex. Hook. | T | Flavonoids, saponins, terpenoids, tannins, reducing sugar, cardiac glycosides | Ahmad et al., 2012 |
| 440 | *Phoebe lanceolata* (Nees) Nees | T | Flavonoids, steroids, alkaloids & their glycosides | Semwal et al., 2009 |
|  | **Liliaceae** | | | |
| 441 | *Fritillaria roylei* Hook. | H | Alkaloids (peimine, peiminine,  peimisine, propeimin), sterol | Bisht et al., 2016 |
| 442 | *Lilium polyphyllum* Don | H | Sugar, steroids, saponins. | Sahu et al., 2016 |
| 443 | *Trillidium govanianum* (D. Don) Kunth. | H | Glycosides, saponins, sterols, flavonoids, carbohydrates | Rahman et al., 2015 |
|  | **Linaceae** | | | |
| 444 | *Linum usitatissimum* L. | H | Linolenic acid, linoleic acid, lignans, cyclic peptides, polysaccharides, alkaloids, cyanogenic glycosides, cadmium | Shim et al., 2014 |
| 445 | *Reinwardtia indica* Dumort. | H | Alkaloids, glycosides, steroids, flavonoids, terpenoids, carbohydrates, saponins. | Abha et al., 2013 |
|  | **Linderniaceae** | | | |
| 446 | *Torenia indica* Saldanha | H | Monoterpene | Shimoda et al., 2012 |
| 447 | *Torenia* crustacea (L.) Cham. & Schltdl. | H | Cucurbitane glycoside ( linderside A), cucurbitane triterpenoid (  lindersin B) | Cheng et al., 2017 |
|  | **Loranthaceae** | | | |
| 448 | *Dendrophthoe falcata* (L.f.) Ettingsh. | S | Carbohydrates, glycosides, steroids, tannins, phenolic compounds, flavonoids, triterpenes. | Sahu et al., 2010 |
|  | **Lythraceae** | | | |
| 449 | *Lagerstroemia indica* L. | T | Phenolic compounds, flavonoids, triterpenoids, alkaloids, glycoside compounds | Diab et al., 2012 |
| 450 | *Lawsonia inermis* L. | S | Carbohydrates, proteins, flavonoids, tannins, phenolic compounds, alkaloids, terpenoids, quinones, coumarins, xanthones, fatty acids | Chaudhary et al., 2010 |
| 451 | *Punica granatum* L. | T | Flavonoids (flavonons, flavanols and anthocyanins), condensed tannins (proanthocyanidins), hydrolysable tannins, (ellagitannins, gallotannins), ellagic acid, gallic acid, punicalagin | Carda et al., 2003; Larrosa et al., 2006; Dahham et al., 2010 |
| 452 | *Woodfordia fruticosa* L. | S | Tannins (isoschimawalin, woodfordin, oenthein), flavonoids (quercetin, myricetin, kaempferol, naringenin, pelargonidin, cyanidin), hecogenin, betulin, octacosanol, β-sitosterol | Chaturvedi et al., 2012 |
|  | **Malvaceae** | | | |
| 453 | *Hibiscus* *esculentus* L. | H | Flavones, polysaccharide, pectin, amino acids, phenolic acid,flavanoidal glycosides, quercetin, coumarin scopoletin | Liao et al., 2012; Lakshmi et al., 2016 |
| 454 | *Abutilon indicum* (L.) Sweet | H | Flavonoids (quercetin), saponins, alkaloids, tannins, phenolic compounds, terpenoids, monoterpenoids (linalool), glycoproteins, polysaccharides | Dashputre and Naikwade, 2011 |
| 455 | *Bombax ceiba* L. | T | Sesquiterpene lactone, flavonol glycoside, shamimicin, lupeol | Saleem et al., 2003 |
| 456 | *Gossypium arboreum* L. | S | Steroids, tannins,flavonoids, reducing sugar, terpenoids | Kazeem et al., 2013 |
| 457 | *Gossypium* herbaceum L. | S | Trachorogenin, quercetin, catechin, N-(*p*-Coumaroyl)serotonin, 5-*O*-glucoside, serotonin derivatives, 2-hydroxyarctiin, matairesinol glycoside, sesquiterpene glucosides | Nagatsu et al., 2012 |
| 458 | *Grewia optiva* J.R. Drum ex Burret | T | Uranic acid, lignins, adhesive pectins, hemicelluloses | Singha and Rana, 2012 |
| 459 | *Hibiscus rosa- sinensis* L. | H | Flavonoids, flavonoid glycosides, hibiscetin, cyanidine, cyanidin glucosides, taraxeryl acetate, β-sitosterol, campesterol, stigmasterol, ergosterol, citric acid, tartaric acid, oxalic acids, cyclopropenoids, anthocyanin | Maganha et al., 2012 |
| 460 | *Hibiscus sabdariffa* L. | H | Alkaloids, ascorbic acid, β-carotene, anisaldehyde, arachidic acid, citric acid, malic acid, tartaric acid, glycinebetaine, trigonelline, anthocyanins (cyanidin-3-rutinoside, delphinidin, hibiscin), flavonol glycosides (hibiscetin-3-monoglucoside, gossypetin-3-glucoside, gossypetin7-glucoside, gossypetin-8-glucoside, sabdaritrin), quercetin, protocatechuic acid, pectin, polysaccharides, mucopolysaccharides | Maganha et al., 2012 |
| 461 | *Malva neglecta* Wallr. | H | Malvidin, catchin, cinnamic acid, tannic acid | Ghanati et al., 2013 |
| 462 | *Malva verticillata* L. | H | Polysaccharides, acetylated glucomannans, glucans | Paulsen, 2002 |
| 463 | *Malvastrum coromandelianum* Garcke. | S | Alkaloids, fixed oils, saponins, phenolic, tannins, carbohydrates, protein | Sanghai et al., 2013 |
| 464 | *Sida* carpinifolia L. f. | S | Alkaloids, steroids | Karou et al., 2005 |
| 465 | *Sida cordata* (Burm. f.) Borss.-Waalk. | S | Flavonoids, terpenoids, phenolic compounds, psuedotannins, choline, oxalic acid | Manisha et al., 2009 |
| 466 | *Sida cordifolia* (Burm.f) Boss. | S | Reducing sugar, alkaloids, steroid, saponins | Momin et al., 2014 |
| 467 | *Sida rhombifolia* L. | S | Tannins, polyphenols, alkaloids, glycosides, flavonoids, saponins | Dzoyem et al., 2010 |
| 468 | *Triumfetta rhomboidea* Jacq. | S | Carbohydrate, glycosides, phytosterol & steroids flavonoids, tannin & phenolic compounds, triterpenoids | Devmurari et al., 2010 |
| 469 | *Urena* sinuata L | H | Sterols, tannins, flavonoids | Mathappan et al., 2010 |
|  | **Marattiaceae** | | | |
| 470 | *Angiopteris yunnanensis Hieron.* | Fn | Angiopteroside, steroids, phenolics | Sultana et al., 2014; Ho et al., 2011 |
|  | **Marsileaceae** | | | |
| 471 | *Marsilea* *vestita* Hook. & Grev | Fn | Flavonoid, tannin, coumarin and carbohydrate, steroid, saponin, xanthoprotein, protein | Mithraja et al., 2011 |
|  | **Martyniaceae** | | | |
| 472 | *Martynia annua* L. | S | Alkaloids, glycosides, flavonoids, fats, tannins,phenolic compound | Kenwat et al., 2014 |
|  | **Melanthiaceae** | | | |
| 473 | *Paris polyphylla* Smith | H | Steroid saponins ( polyphyllin D, diosgeninscin, gracillin, trillin, methylmotogracillin) | Yan et al., 2009 |
|  | **Meliaceae** | | | |
| 474 | *Azadirachta indica* L. | T | Phenols, unsaturated sterols, triterpenes, saponins, phenolic diterpenoids, limonoids), csecomeliacins, csecolimonoids, polysachharides | Joshi et al., 2011 |
| 475 | *Melia aze*darach L. | T | Azadirachtin, toosendanin, 1- cinnamoyl-3-feruoyl-11-hydroxymeliacarpin, volkensin, d-limonene, menthol, 1,8-cineole,citronellal, eugenol, p-menthane-3,8-diol, thymol | Isman, 2006 |
| 476 | *Toona ciliata* Roem. | T | Alkaloids, flavonoids, tannins, saponins, coumarins | Nisa et al., 2013 |
|  | **Menispermaceae** | | | |
| 477 | *Cissampelos* *owariensis* P. Beauv. ex DC. | H | Alkaloid (bebeerines, hayatin, hayatinin), quercitol, sterols | Amresh et al., 2007; Surendran et al., 2011 |
| 478 | *Cocculus hirsutus* (L.) Diels | S | Saponins, steroids, tannins | Savithramma et al., 2011 |
| 479 | *Stephania elegans* Hook. f. & Thoms | H | Alkaloids, epihernandolinol, N-methylcorydalmine, hasubanonin, aknadinin, cyclanoline, magnoflorine, isotetrandrine, isochondodendrine, cycleanine | Semwal et al., 2010 |
| 480 | *Stephania glabra* (Roxb) Mierr. | C | Flavonoids, steroids, alkaloids & their glycosides, pronuciferine, gindarine, gindaricine, gindarinine, hyndarine, magnoflorine, N-thyloxystephanine, N-methylhydoxystepharine, remerine, stephararine, cycleanine | Semwal et al., 2009, Semwal et al., 2010 |
| 481 | *Tinospora cordifolia* (Willd.) Miers. | C | Sesquiterpene tinocordifolin, sesquiterpene glycoside (tinocordifolioside),arabinogalactan, phytoecdysones (ecdysterone,makisterone), alkaloids (berberine) | Srinivasan et al., 2008 |
| 482 | *Tinospora sinensis* (Lour.) Merr. | C | Berberine, tinosporicide, menispermacide, palmatine, (+)− malabarolide, tinosinen I | Srinivasan et al., 2008 |
| **Moraceae** | | | | |
| 483 | *Artocarpus heterophyllus* Lam. | T | Reducing sugars, flavones, and polyphenols, stilbenes, lignans, condensed tannins | Jiang et al., 2013 |
| 484 | *Artocarpus lacucha* Buch.-Ham. | T | Phenols, flavonoids, rutin, resocinol | Singhatong et al., 2010 |
| 485 | *Ficus auriculata* Lour | T | Betulinic acid, lupeol, stigmasterol, bergapten, scopoletin, β-sitosterol-3-O-β-D-glucopyranoside, myricetin, quercetin-3-O-β-D glucopyranoside | El-Fishawy et al., 2011 |
| 486 | *Ficus benghalensis* L. | T | Glucoside, 20-tetratriaconthene-2-one, 6-heptatriaconthene10-one, pentatriacontan-5-one, beta sitostirolalpha-D-glucose, meso-inositol, caoytchoue,resin, albumin, cerin, sugar, malic acid | Govindarajan, 2010 |
| 487 | *Ficus carica* L. | T | Ficusin, bergaptene | Ahmad and Beg, 2001 |
| 488 | *Ficus palmata* Forsk. | T | Carbohydrates,glycosides, alkaloid, flavonoids, saponins, tannins, unsaturated triterpenoids, sterol, resin | Saklani and Chandra, 2011 |
| 489 | *Ficus racemosa* L. | T | Tannin, wax, saponin gluanol acetate, β-sitosterol, leucocyanidin- 3 – O – β – D - glucopyrancoside, leucopelargonidin – 3 – O – β – D - glucopyranoside, leucopelargonidin – 3 – O – α – L - rhamnopyranoside, lupeol, ceryl behenate, lupeol acetate, α-amyrin acetate, leucoantho cyaniding | Joseph and Raj, 2010 |
| 490 | *Ficus religiosa* L. | T | Bergaptol, bergapten | Ahmad and Beg, 2001 |
| 491 | *Ficus rumphii* Blume | T | β sitosterol | Bishnoi et al., 2017 |
| 492 | *Ficus semicordata* Buch.Ham ex J.E Smith | T | 4‐methylanisole, fatty acid derivatives, mono‐ & sesquiterpenoids, shikimic compounds, | Chen et al., 2009 |
| 493 | *Morus alba* L. | T | Alkaloids, flavonoids, flavones, flavanones, stilbenes, benzophenones, coumarin derivatives, terpenoids | Chen et al., 2013 |
|  | **Morchellaceae** | | | |
| 494 | *Morchella esculenta* L. | Fg | Carbohydrates, proteins, organic acids, phenolic compounds, tocopherols. | Heleno et al., 2013 |
|  | **Morinaceae** | | | |
| 495 | *Morina longifolia* Wall. ex DC. | H | Alkaloids, tannins, flavanoids, saponin steroids, cardiac glycosides, reducing Sugars | Yousuf et al., 2014 |
| 496 | *Moringa oleifera* Lam. | Fg | α & γ tocopherols,phenolic compounds, β-carotene, proteins, unsaturated fatty acids, (oleic acid), carbohydrates | Ferreira et al., 2008 |
|  | **Musaceae** | | | |
| 497 | *Musa paradisiaca* L. | T | Carbohydrates, catecholamines (norepinephrine, serotonin, dopamine), tryptophan, indole compounds, pectin, flavonoids & related compounds (leucocyanidin, quercetin, 3-O galactoside, 3-O-glucoside, 3-O-rhamnosyl glucoside), serotonin, nor-epinephrine, tryptophan, indole compounds, tannin, starch, iron, sugars, albuminoids, sitoindoside-I, II,III & IV , steryl glycosides | Imam and Akter, 2011 |
|  | **Myricaceae** | | | |
| 498 | *Myrica esculenta* Buch.-Ham | T | Sugars, tannins, gallic acid, catechin, chlorogenic acid, ρ–coumaric acid, myricanol, myricanone, epigallocatechin 3-O-gallate, prodelphinidin dimmers (epigallocatechin-(4β→8)- epigallocatechin 3-O-gallate, 3- Ogalloyl epigallocatechin-(4β→8)-epigallocatechin3-O-gallate), castalagin, 4-hydroxy-1,8-cineole 4-O-β- Dapiofuranosyl-(1→6)-β-D-glucopyranosie, (1S,2S,4R)-2-hydroxy1,8-cineole β-D-glucopyranoside, corchoionoside C, (6S,9R)- roseoside, myricanol, 5-O-β-D-glucopyranosyl myricanol, arjunolic acid, arjunglucoside, 3-epi-ursonic acid, 3-O-(E)-caffeoylursonic acid, myricetin, myricitrin | Panthari et al., 2012 |
|  | **Myristicaceae** | | | |
| 499 | *Myristica fragrans* Houtt. | T | Mono & sesquiterpenes, diterpenes, aliphatic hydrocarbons, coumarins, homologues of phenylpropanoids, terpenes | Dorman and Deans, 2000 |
|  | **Myrtaceae** | | | |
| 500 | *Eucalyptus tereticornis* Sm. | T | α-pinene, 1,8-cineole,β-citronellal, (−)-isopulegol, (+)-β-citronellol (10.73%) | Singh et al., 2009 |
| 501 | *Psidium* pyriferum L. | T | Carbohydrates, fats, proteins, hexanal, γ –butyrolactone, (E)-2-hexenal, (E,E)-2,4-hexadienal, (Z)-3-hexenal, (Z)- 2-hexenal, (Z)-3-hexenyl acetate,phenols, 3-caryophyllenen, nerolidol, 3-phenylpropyl acetate,caryophyllene oxide, 3-penten-2-ol, 2-butenyl acetate | Gutiérrez et al., 2008 |
| 502 | *Syzygium aromaticum* (L.) Merr. & Perry | T | Eugenol, β-caryophyllene, eugenyl acetate | Rana et al., 2011 |
| 503 | *Syzygium cumini* (L.) Skeel | T | Anthocyanins, glucoside, ellagic acid, isoquercetin, kaemferol, myrecetin | Ayyanar and Subash-Babu, 2012 |
|  | **Nephrolepidaceae** | | | |
| 504 | *Nephrolepis cordifolia* (L.) Presl | Fn | Flavonoids, tannins, alkaloids, reducing sugars, triterpenoids, steroids | Rani et al., 2010 |
|  | **Nyctaginaceae** | | | |
| 505 | *Boerhavia* coccinea Mill. | H | Flavonoids, alkaloids, steroids, triterpenoids, lipids, lignins, carbohydrates, proteins, glycoproteins, punarnavine, hypoxanthine 9-L-arabinofuranoside, ursolic acid, punarnavoside, liirodendrin, flavonoids, saponins, glycosides, phenols | Awasthi and Verma, 2006, Suriyavathana et al., 2012 |
| 506 | *Mirabilis jalapa* L. | H | Rotenoids (mirabijalones A–D, boeravinones C & F), isoquinoline derivate,terpenoids, steroids, phenolic compounds, stigmasterol, β-sitosterol, ß-sitosterol- β-d-glucoside, ursolic acid, mirabalisoic acid, mirabalisol, trigonellin | Aoki et al., 2008 |
|  | **Oleaceae** | | | |
| 507 | *Fraxinus micrantha* Lingelsheim | T | Coumarins, Secoiridoids, lignans, flavones, flavonols, kaempferol, quercetin, phenolic compounds | Kostova and Iossifova, 2007 |
| 508 | *Jasminum humile* L. | S | Alkaloids, tannins, flavonoids, coumarins glycosides | Gyawali, 2011 |
| 509 | *Jasminum multiflorum* (Burm. f.) Andrews | S | Lactone secoiridoids, jasmolactones A-D, bicyclic 2-oxo-oxepano [4,5-c] pyran | Shen and Chen,1989 |
| 510 | *Nyctanthes arbor-tristis* L. | T | Saponins, phenols, tannins, flavanoids, terpenoids, carbohydrates, phenolic compounds | Priya and Ganjewala, 2007 |
|  | **Onagraceae** | | | |
| 511 | *Epilobium angustifolium* L. | H | Llagitannins, ellagitannins (coriariin A), oenothein B | Schepetkin et al., 2009 |
|  | **Ophioglossaceae** | | | |
| 512 | *Helminthostachys zeylanica* (L.) Hook. | Fn | Stilbenes, phenolics, flavonoids ( Ugonin M) | Wong et al., 2014; Wu et al., 2017 |
|  | **Orchidaceae** | | | |
| 513 | *Acampe carinata* (Griff.) Panigrahi | H | Alkaloids, triterpenoids, flavonoids,stilbenoids | Singh and Duggal, 2009 |
| 514 | *Calanthe tricarinata* Lindl. | H | Alkaloids, triterpenoids, flavonoids,stilbenoids | Singh and Duggal, 2009 |
| 515 | *Coelogyne cristata* Lindl. | H | Phenanthrenes, coeloginanthridin*(*3,5,7-trihydroxy-1,2-dimethoxy-9,10-dihydrophenanthrene)*,* coeloginanthrin*(*3,5,7-trihydroxy-1,2-dimethoxyphenanthrene*), stilbenoids (*coeloginone*, c*oeloginanthrone*)* | Mitra et al., 2017 |
| 516 | *Cypripedium cordigerum* D. Don | H | Alkaloids, triterpenoids, flavonoids,stilbenoids | Singh and Duggal, 2009 |
| 517 | *Dactylorhiza hatagirea* D. Don | H | Glucoside, starch, mucilage, albumen, dactylorhins A - E, dactyloses A and B, lipids | Ranpal, 2009 |
| 518 | *Epipactis helleborine* (L.) Crantz | H | Alkaloids, triterpenoids, flavonoids,stilbenoids | Singh and Duggal, 2009 |
| 519 | *Eulophia dabia* (Don) Hochr. | H | Tetracosanoic acid, behenic acid,linoleic acid, palmitic acid, eruccic acid, oleic acid, arachidic acid, stearic acid | Nisar et al., 2013 |
| 520 | *Habenaria edgeworthii* Hook. f. ex Collett | H | Alkaloids, saponins, carotenoids, anthocyanins, polyphenols | Giri et al., 2012 |
| 521 | *Habenaria intermedia* D.Don | H | Gallic acid (3,4,5-trihydroxybenzoic acid), scopoletin (7-hydroxy-6-methoxy-2H-1-benzopyran-2-one) | Habbu et al., 2012 |
| 522 | *Habenaria pectinata* (J.E. Smith) D. Don | H | Alkaloids, flavonoids, carotenoids, anthocyanins, sterols | Jagtap et al., 2014 |
| 523 | *Malaxis acuminata* D.Don | H | Fatty acids, α-hydroxy acids, phenolic acids, sterols, sugars, glycosides | Bose et al., 2017 |
| 524 | *Malaxis cylindrostachya* O. Kuntze | H | Alkaloids, triterpenoids, flavonoids,stilbenoids | Singh and Duggal, 2009 |
| 525 | *Malaxis muscifera* (Lindl.) Kuntze. | H | Alkaloid, glycoside, flavonoids | Kumar, 2014 |
| 526 | *Oberonia falconeri* Hook.f. | H | Alkaloids, triterpenoids, flavonoids,stilbenoids | Singh and Duggal, 2009 |
| 527 | *Pholidota articulata* Lindl. | H | Isoflavidinin, iso-oxoflavidinin, modified 9,10-dihydrophenanthrenes | Majumder et al., 1982 |
| 528 | *Ponerorchis chusua* (D. Don) Soo | H | Alkaloids, triterpenoids, flavonoids,stilbenoids | Singh and Duggal, 2009 |
| 529 | *Orchis habenarioides* King & Pant | H | Alkaloids, triterpenoids, flavonoids,stilbenoids | Singh and Duggal, 2009 |
| 530 | *Pholidota articulate* Lindl. | H | Alkaloids, triterpenoids, flavonoids,stilbenoids | Singh and Duggal, 2009 |
| 531 | *Rhychostylis retusa* (L.) Bl. | H | Alkaloid, terpenoids, flavonoids, tannins, steroids, coumerins, amino acides carbohydrates | Bhattacharjee and Islam, 2015 |
| 532 | *Satyrium nepalense* D.Don | H | Alkaloids, carbohydrates/glycosides, flavonoids, unsaturated sterols/triterpenes | Saklani et al., 2011 |
| 533 | *Spiranthes sinensis* (Pers.) Ames. | H | Alkaloids, triterpenoids, flavonoids,stilbenoids | Singh and Duggal, 2009 |
| 534 | *Vanda tessellata* (Roxb.) Hook. f. Ex G. Don. | H | Alkaloid, glucoside, tannins, β-sitosterol, γ-sitosterol, aliphatic compound, fatty oils, resins, tetracosyl ferrulate, β-sitosterol-D-glucoside, 2,7,7-tri methyl bicyclo [2.2.1] heptanes, 17-β-hydroxy-14,20-epoxy-1-oxo-[22R]-3β-[O-β-d-glucopyranosyl]-5,24-withadienolide, melian | Chowdhuryet al., 2014 |
|  | **Orobanchaceae** | | | |
| 535 | *Lindenbergia indica* (L.) Vatke | H | β-setosterol, β-setosterolpalmitate, β-setosterol- β-D-glucosidase, mannitol, apigenin, Carbohydrate, Glycosides, Glucosides, Saponins, Steroids, tannins | Jahan, 2016 |
| 536 | *Orobanche alba* stephon | H | Tricin (5,7,4′-trihydroxy-3′,5′-dimethoxyflavone) | Zhou and Ibrahim, 2010 |
| 537 | *Pedicularis gracilis* Wall. ex Benth. | H | Phenols, phenylethanoids, terpenoids, tannins, phenylpropanoids, flavonoids, iridoids, lignans, alkaloids | Yatoo et al., 2017 |
| 538 | *Pedicularis hoffmeisteri* Klotz. | H | Phenols, phenylethanoids, terpenoids, tannins, phenylpropanoids, flavonoids, iridoids, lignans, alkaloids | Yatoo et al., 2017 |
| 539 | *Pedicularis longiflora Rudolph subsp. tubiformis* (Klotz.) Pennell | H | Phenols, phenylethanoids, terpenoids, tannins, phenylpropanoids, flavonoids, iridoids, lignans, alkaloids, glycosides, boschnaloside, alyssonoside, leucosceptoside A, isoverbascoside, leucosceptoside B, verbascoside | Yatoo et al., 2017; Lan et al., 2018 |
| 540 | *Pedicularis pectinata* Wall.ex Benth | H | Phenols, phenylethanoids, terpenoids, tannins, phenylpropanoids, flavonoids, iridoids, lignans, alkaloids | Yatoo et al., 2017 |
| 541 | *Pedicularis punctata* Decne | H | Phenols, phenylethanoids, terpenoids, tannins, phenylpropanoids, flavonoids, iridoids, lignans, alkaloids | Yatoo et al., 2017 |
|  | **Oxalidaceae** | | | |
| 542 | *Averrhoa carambola* L. | T | Saponin, alkaloid, tungstosilicic acid, steroid, tannin, flavonoid | Vasconcelos et al., 2005 |
| 543 | *Biophytum* abyssinicum Steud. ex A. Rich. | H | γ sitosterol terpenoids, steroids, tannins, flavonoids, saponins | Oraon and Sinha, 2012 |
| 544 | *Xanthoxalis* corniculata (L.) Small | H | Carbohydrates, glycosides, phytosterols, phenolic compounds/tannins, flavonoids, proteins, amino acids, volatile oils | Raghavendra et al., 2006 |
|  | **Paeoniaceae** | | | |
| 545 | *Paeonia emodi* Wall ex Hooker.f. | H | ß- glucuronidase-inhibiting triterpene, 1,3,5,23,24- pentahydroxy-30-12,20(29)-dien-28-oic acid, oleanolic acid, betulinic acid, ethyl gallate, methyl grevillate, 1,5-dihydroxy-3-methylanthraquinone, monoterpene glycosides, wurdin,benzoylwurdin, paeoniflorin, lactiflorin oxypaeoniflorin,emodinol | Khan et al., 2005 |
|  | **Pandanaceae** | | | |
| 546 | *Pandanus odoratissimus* L. | S | Steroids, saponins, terpinoids, glycosides, tannins, flavonoids, phenolics | Raju et al., 2011 |
|  | **Papaveraceae** | | | |
| 547 | *Argemone maxicana* L. | H | Alkaloids,terpenoids, flavonoids, phenolic Acids (tannic acid,caffeic acid**,**ferulic acid), aliphatic & aromatic compounds | Brahmachari et al., 2013 |
| 548 | *Dicranopteris linearis* (Burm. f.) Und. | Fn | Flavonoids, saponins, tannins, steroids, triterpenes | Zakaria et al., 2006 |
| 549 | *Meconopsis aculeata* Royle. | H | Terpenoids, phlobatannins, flavonoids, alkaloids | Ahmad et al., 2016 |
| 550 | *Meconopsis robusta* Royle | H | Isoquinoline alkaloids and flavonoids. | Guo et al., 2016 |
| 551 | *Papaver somniferum* L. | H | Alkaloids ( morphine, codeine, thebaine, papaverine, narcotine), coumarins, flavonoids, saponins, tannins | Singh et al., 2000, Pascual et al., 2002 |
| 552 | *Corydalis cashemeriana* Royle | H | Alkaloids | Jeong et al., 2012 |
| 553 | *Corydalis cornuta* Royle | H | Alkaloids | Jeong et al., 2012 |
| 554 | *Corydalis govaniana* Wall. | H | Alkaloids ( govaniadine, caseadine, caseamine, protopine) | Jeong et al., 2012; Shrestha et al., 2013 |
| 555 | *Corydalis meifolia* Wall. | H | Alkaloids | Jeong et al., 2012 |
| 556 | *Fumaria indica* (Hausk.) Pugsly | H | Alkaloids (protopine), phenolics, flavonoids | Gupta et al., 2012; Ivanov et al., 2014 |
| 557 | *Fumaria officinalis* L. | H | Phenolics, flavonoids | Ivanov et al., 2014 |
|  | **Passifloraceae** | | | |
| 558 | *Passiflora foetida* L. | H | Harmaline  alkaloids, flavonoid (hydrocyanic  acid) | Santosh et al., 2011 |
|  | **Parmeliaceae** | | | |
| 559 | *Everniastrum cirrhatum* (Fr.)Hale | Fg | Alkaloids, saponins, tannins, terpenoids. | Ramamoorthy et al., 2004 |
| 560 | *Parmotrema reticulatum* (Taylor) M.Choisy | Ln | Tannins, flavonoids, alkaloids, proteins carbohydrates | Rashmi and Rajkumar, 2014 |
|  | **Pedaliaceae** | | | |
| 561 | *Sesamum* *indicum* L. | H | Phenolic compounds, flavonoid, flavonol | Mohdaly et al., 2011 |
|  | **Pentaphylacaceae** | | | |
| 562 | *Eurya acuminata* DC. | T | Linalool, (9Z)-tricosene, nonanal, α-terpineol, geraniol | Motooka et al., 2015 |
|  | **Phyllanthaceae** | | | |
| 563 | *Phyllanthus amarus* Schumach. & Thonn. | H | Alkaloids, flavonoids, lactones, steroids, triterpenes, lignans, tannins, dotriacontanyl docosanoate, triacontanol and a mixture of oleanolic acid and ursolic acid | Ali et al., 2006; Cui et al., 2010 |
| 564 | *Leptopus* chinensis (Bunge) Pojark. | S | Alkaloids, flavonoids, lactones, steroids, triterpenes, lignans, tannins | Cui et al., 2010 |
| 565 | *Phyllanthus emblica* L. | T | Proanthocyanidin polymer (phyllemtannin), Alkaloids, flavonoids, lactones, steroids, triterpenes, lignans, tannins, gallic acid, geraniin | Zhang et al., 2004; Lin et al., 2008; Cui et al., 2010 |
| 566 | *Phyllanthus fraternus* Webster. | H | Alkaloids (morphine, boldine), tannins, saponin, terpenoid, steroid | Cui et al., 2010; Kavit et al., 2013 |
| 567 | *Phyllanthus urinaria* L. | H | Alkaloids, flavonoids, lactones, steroids, triterpenes, lignans, tannins,geraniin, 1,3,4,6-tetra-O-galloyl-b-D-glucose | Lin et al., 2008; Cui et al., 2010 |
| 568 | *Bridelia retusa* (L.) Sprengel | T | Isoflavone, 4-[(E)-6-methyl-4-oxohept-2-en-2-yl)] benzoic acid, 4-[(E)-6-methyl-4-oxohept-2,5-dien-2-yl)] benzoic acid, 4-[(R)-6-methyl-4-oxohept-5-en-2-yl)] benzoic acid , 4-[(R)-6-methyl-4-oxoheptan-2-yl)] benzoic acid, (−)-isochaminic acid, 5-allyll1,2,3-trimetoxy-benzene (elemicin), (+)-sesamin, 4-isopropylbenzoic acid, cyanogenic glycosides, triterpenes, ketone, tannins | Ngueyem et al., 2009 |
|  | **Phytolaccaceae** | | | |
| 569 | *Phytolacca* *latbenia* (Moq.) H. Walter | H | Triterpene saponins, phytolaccoside F | Strauss et al., 1995 |
|  | **Pinaceae** | | | |
| 570 | *Abies pindrow* Royle | T | Glycosides, terpenoids, flavonoids | Singh, 2005 |
| 571 | *Abies spectabilis* Spach. | T | Proantocyanidin, prodelphynidin B4, cyclograndisolide, trans-docosanil ferulate | Dall Acqua et al., 2012 |
| 572 | *Cedrus deodara* Roxb.ex D. Don | T | α- terpineol, linalool, limonene, anethole, caryophyllene, eugenol | Zeng et al., 2012 |
| 573 | *Picea smithiana* (Wall.) Boiss. | T | Quercetin, dihydroquercetin, dihydromyricetin | Bashir et al., 2017 |
| 574 | Pinus roxburghii Sarg. | T | Resin acids, flavanoid, α-pinene, β-pinene, car-3-ene, longifolene | Kaushik et al., 2013 |
| 575 | *Pinus wallichiana* Jacks. | T | β-pinene,α-pinene | Dar et al., 2012 |
|  | **Piperaceae** | | | |
| 576 | *Peperomia* *reflexa* (L. f.) A. Dietr. | H | Alkaloids, flavonoids, glycosides, phenols, saponins, sterols, carbhohydrates, terpenoides, tannins, aminoacids, gums, mucilage | Nishanthi et al., 2012 |
| 577 | *Piper longum* L. | H | Alkaloids (piperine), volatile oil, starch, protein, saponins, carbohydrates, amygdalin, palmitic acid, hexadecenoic acid, stearic acid, linoleic acid, oleic acid, arachidic acid, behenic acid | Zaveri et al., 2010 |
| 578 | *Piper nigrum* L. | H | E, 4E, 8Z-*N*-isobutyleicosatrienamide,pellitorine, trachyone, pergumidiene, isopiperolein B | Reddy et al., 2004 |
|  | **Plantaginaceae** | | | |
| 579 | *Bacopa monnieri* L. | H | Triterpenoid saponins, jujubogenins, pseudojujubogenin. Bacosides | Deepak et al., 2005 |
| 580 | *Plantago depressa* Willd. | H | Caffeic acid, acteoside, isoacteoside, chlorogenic acid | Olennikov et al., 2011 |
| 581 | *Plantago himalaica* Pingler. | H | Plantamajoside, verbascoside | Ravn et al., 2015 |
| 582 | *Plantago lanceolata* L. | H | Plantain | Stewart,1996 |
| 583 | *Plantago major* L. | H | Polysaccharides, lipids, caffeic acid derivatives, flavonoids, iridoid glycosides,terpenoids | Samuelsen, 2000 |
| 584 | *Plantago ovata* Forsk | H | Crude fiber, iridoids, phenols, polysaccharides, sterols, alkaloids, cumarines | Motamedi et al., 2015 |
| 585 | *Picrorhiza kurrooa* Royle ex Benth. | H | Picroliv, picrosides II &III, cucurbitacins, apocynin, drosin | Verma et al., 2009 |
| 586 | *Scoparia dulcis* L. | H | Scoparic acid A & B, scopadulcic acid A & B, scopadulciol, scopadulin, alkaloids, tannins, carbohydrate, gum, glycoside | Zulfiker et al., 2010 |
| 587 | *Veronica anagallis aquatica* L. | H | Aquaticoside A, aquaticoside B, aquaticoside C, veronicoside, catalposide, verproside, verminoside, martynoside, aucubin, catalpol, benzoic & cinnamic acid esters | Küpeli et al., 2005 |
|  | **Platanaceae** | | | |
| 588 | *Platanus orientalis* L. | T | Flavonoids, pentacyclic triterpenoids, tannins, caffeic acid | Hajhashemi et al., 2011 |
|  | **Plumbaginaceae** | | | |
| 589 | *Plumbago zeylanica* L. | H | Phenols, flavonoids, coumarin, naphthoquinones ( Plumbagin), binaphthoquinones, anthroquinone | Tilak et al., 2004 |
|  | **Poaceae** | | | |
| 590 | *Cenchrus biflorus* Roxb. | S | 2,3-dihydro-3,5-di hydroxyl-6- methyl-4H-pyran, 2-methoxy-4-vinylphenol, naphthalene, 1-tetradecene, heptadecane, dodecanoic acid, hexadecanoic acid, squalene, octadecanoic acid | Arora and Kumar, 2018 |
| 591 | *Cymbopogon martini* (Roxb.) Wats. | H | Monoterpenes ( geraniol,isomenthyl acetate, linalool, Geranial), | Katiki et al., 2011 |
| 592 | *Capriola* dactylon (L.) Kuntze | H | Alkaloids, anthroquinone, flavonoids, saponins, steriods, triterpenoids, tannins, amino acids, α- amylase, β - amylase, carbohydrate, glutamine, protein, prolin | Suresh, 2008 |
| 593 | *Dendrocalamus hamiltonii* Nees et Arn. ex Munro | H | Holocellulose, α, β & γ cellulose, pentosan | Sharma et al., 2011 |
| 594 | *Desmostachya bipinnata* (Linn.) Stapf. | H | Alkaloids, carbohydrates, proteins, tannins, phenolic compounds, flavonoids, triterpenoids, glycosides. | Golla et al., 2011 |
| 595 | *Echinochloa* crusgalli var. frumentacea (Link) W. Wight | H | Malic acid, trans-aconitic acid, (+)-isocitric acid, 5-O-caffeoylquinic acid, 4-O-caffeoylquinic acid, isocarlinoside, 2″-O-rhamnosylisoorientin, 7-O-(2″-O-glucuronosyl)glucuronosyltricin | Kim et al., 2008 |
| 596 | *Eleusine coracana* (L.) Gaertn. | H | Phenolic acids (protocatechuic, gallic acid, caffeic acid,  ferulic acid, coumaric acids) | Subba and Muralikrishna, 2002 |
| 597 | *Eleusine indica* (L.) Gaertn. | H | C-glycosylflavones, lavonoids, phenols, phenolic glycosides, saponins, cyanogenic glycosides, unsaturated lactones, glucosinolates | Iqbal and Gnanaraj, 2012 |
| 598 | *Eulaliopsis binata* (Retz.) Hubb. | H | Holocellulose, α-cellulose, lignin | Gautam et al., 2016 |
| 599 | *Hordeum vulgare* L. | H | Phenolics, flavan‐3‐ols (proantocyanidin), flavonols, phenolic acids, polar esters, procyanidin B3, prodelphinidin B3, carotenoids (lutein, zeaxanthin), tocopherols (α, δ & γ). | Goupy et al., 1999 |
| 600 | *Imperata cylindrica* L. | H | Carbohydrates, glycosides, triterpenoids, phenolic compounds / tannins, flavonoids, proteins, volatile oils | Padma et al., 2013 |
| 601 | *Oryza sativa* L. | H | g-oryzanol, tocopherols, tocotrienols | De Mira et al., 2009 |
| 602 | *Panicum miliaceum* L. | H | Phenolic compounds ( salicylic acid, β-naphthol, chlorogenic acid, tannic acid | Datta and Nanda, 1985 |
| 603 | *Saccharum spontaneum* L. | H | Alkaloids, flavonoids, reducing sugar, tannins, saponin | Vhuiyan et al., 2008 |
| 604 | *Triticum aestivum* L. | H | *p*-Hydroxybenzoic, trans-*p*-coumaric acid, cis-*p*-coumaric acid, syringic acid, vanillic acid, trans-ferulic acid, cis-ferulic acid, 2,4-dihydroxy-7-methoxy-1,4-benzoxazin-3-on | Wu et al., 2000 |
| 605 | *Chrysopogon* zizanioides (L.) Roberty | H | 5, 10-pentadecadiyn-1-ol, α-curcumene, hydroxy junipene, (+) cycloisosativene, valencine, selino 3,7 (11)-diene | Gupta et al., 2012 |
| 606 | *Zea mays* L. | H | cis-α-terpineol, 6,11-oxidoacor4-ene, citronellol, trans-pinocamphone, eugenol, neo-iso-3- thujanol, cis-sabinene hydrate | El-Ghorab et al., 2007 |
|  | **Polygonaceae** | | | |
| 607 | *Aconogonum tortuosum* (D. Don) Hara | H | Flavonoids, phenolic compound, saponin compounds | Kumar and Rana, 2017 |
| 608 | *Polygonum* affine D. Don | H | Flavonols (quercetin, isorhamnetin), flavonol C-glycosides ( luteolin 6-C-β-D-glucopyranoside, luteolin 8-C-β-D-glucopyranoside, apigenin 6-C- β-D-glucopyranoside, apigenin 8-C-β-D-glucopyranoside, triacetin 8-C-β-D-glucopyranoside) | Voronkova and Vysochina, 2014 |
| 609 | *Polygonum* amplexicaule D. Don var. amplexicaule | H | Phenolcarbonic acids, flavonoids, triterpenoids, steroids | Voronkova and Vysochina, 2014 |
| 610 | *Polygonum* viviparum L. var. viviparum | H | Tannins, carotene, steroids (β-citosterine, daucosterin), phenolcarboxylic acids (ferulic acid,caffeic acid, chlorogenic acid, gallic acid) | Voronkova and Vysochina, 2014 |
| 611 | *Fagopyrum dibotrys* (D. Don) Hara | H | Phenols ( 3-methyl-gossypetin 8-*O*-β-d-glucopyranoside, diboside A | Wang et al., 2005 |
| 612 | *Fagopyrum esculentum* Moench | H | Phenols, flavonoids, total flavanols, oligomeric proanthocyanidins | Quettier-Deleu et al., 2000 |
| 613 | *Fagopyrum tataricum* Gaertn. | H | Flavonoids (rutin, quercitrin, quercetin) | Fabjan et al., 2003 |
| 614 | *Oxyria digyna* (L.) Hill | H | Flavonoids (vitexin) | Orhan et al., 2009 |
| 615 | *Polygonum* nepalense Meisn. | H | Alkaloid (1-ethenyl-4,8-dimethoxy-9H-pyrido-3,4-β) | Lepcha et al., 2010 |
| 616 | *Polygonum* polystachyum Wall. ex Meisn. var. polystachyum | H | Aliphatic ester, pentacosanyl heptacosanoate, E-sitosterol, E-sitosterol-E-D-glucoside, quercetin, quercetin-3-O-L-rhamnopyranoside, flavon-3-ol glycosides, catechin | zda et al., 2008 |
| 617 | *Rheum australe* D. Don | H | Anthraquinone (aloe-emodin, emodin, physcion, chrysophanol, rhein, piceatannol), resveratrol | Rokaya et al., 2012 |
| 618 | *Rheum* spiciforme Royle | H | Alkaloids, phenols, flavonoids, terpenoids, naphthoquinones, essential oils | Dar et al., 2016 |
| 619 | *Rheum webbianum* Royle | H | Anthraquinones (rhein, emodin, aloe-emodin, physcion, chrysophanol) | Rashid et al., 2014 |
| 620 | *Rumex acetosa* L. | H | Anthraquinones (emodin, chrysophanol, physcion) | Lee et al., 2005 |
| 621 | *Rumex hastatus* D. Don | H | Hastatusides A & B, resveratrol, rumexoside, torachrysone-8-yl β-D-glucopyranoside, rutin, nepodin, orientaloside | Sahreen et al., 2005 |
| 622 | *Rumex nepalensis* Spreng | H | Rumexneposides A & B, physcion, chrysophanol-8-O-b-Dglucopyranoside, torachrysone, emodin-8-O-b-D-glucopyranoside, emodin-8-O-b-D-(60 -O-acetyl)glucopyranoside, chrysophanol, emodin, citreorosein, resveratrol, nepodin-8-O-b-D-glucopyranoside, torachrysone-8-O-b-D-glucopyranoside, chrysophanol-8-O-b-D-(60 -O-acetyl)glucopyranoside | Liang et al., 2010 |
|  | **Portulaccaceae** | | | |
| 623 | *Portulaca oleracea* L. | H | Organic acids, alkaloids, coumarins, flavonoids, cardiac glycosides, anthraquinone glycosides, alanine, catechol, saponins, tannins. β-sitosterol, β-sitosterol-glucoside, N,N`- dicyclohexylurea, allantoin | Rasheed et al., 2004 |
|  | **Primulaceae** | | | |
| 624 | *Anagallis arvensis* L. | H | Volatile oil, saponins, tannins, primin, cyclamen, | Al-Sultan et al., 2003 |
| 625 | *Myrsine africana* L. | S | Myrsine saponin | Li and McLaughlin, 1989 |
| 626 | *Myrsine semiserrata* Wall. | S | Tannin, flavonoid, embelin | Wangchuk et al., 2011; Shuveksh et al., 2017 |
| 627 | *Primula denticulata* Sm. | H | Flavonoids, saponins | Semwal et al., 2007 |
| 628 | *Primula elliptica* Royle | H | Flavonoid, saponins, Protoprimulagenin A, priverosaponin B, Macrophyllicin | Najmus-Saqib et al., 2009 |
| 629 | *Primula macrophylla* D. Don | H | 2-phenylchromone, flavonoid, saponins, Protoprimulagenin A, priverosaponin B, Macrophyllicin | Najmus-Saqib et al., 2009 |
|  | **Pteridaceae** | | | |
| 630 | *Adiantum capillus-veneris* L. | Fn | Flavonoids, alkaloids, tannins, saponins, cardiac glycosides, tarpenoids, steroids, reducing sugars | Nyarko *et al*., 2012 |
| 631 | *Cheilanthes bicolor* (Roxb.in Griff.) | Fn | Flavonoid compounds ( kaempferol 3, 5 dimethyl ether, kaempferol -3- methyl ether) | Kabdwal and Verma, 2010 |
| 632 | *Pteris biaurita* L. | Fn | Eicosense, heptadecanes, pterosins  (indanone derivatives) | Syrchina and Semenov, 1982; Dalli *et al*., 2007 |
| 633 | *Pteris vittata* L. | Fn | Alkaloids, phenolic compounds, flavonoids, saponins, tannins, pterosins  (indanone derivatives) | Syrchina and Semenov, 1982; Gracelin et al., 2013 |
| 634 | *Pteris wallichiana* Agardh, Recens. | Fn | Pterosins  (indanone derivatives) | Syrchina and Semenov, 1982 |
| **Putranjivaceae** | | | | |
| 635 | Putranjiva roxburghii Wall. | T | Phenols, alkaloids, saponins, steroids, flavonoids, glycosides | Raghavendra et al., 2010 |
| **Ranunculaceae** | | | | |
| 636 | *Aconitum balfourii* stapf. | H | Alkaloids, flavonoids, polysaccharide, free fatty acids | Nyirimigabo et al., 2015 |
| 637 | *Aconitum falconeri* Stapf. | H | Alkaloids (faleoconitine, 3‘-methoxyacoforestinine), karakoline, 3-hydroxy-2-methyl-4H-pyran-4-one, 3,4-dimethoxymethylbenzoate, pseudaconitine | Fatima et al., 2000 |
| 638 | *Aconitum ferox* Wall | H | Alkaloids, Flavonoids, polysaccharide, free fatty acids | Nyirimigabo et al., 2015 |
| 639 | *Aconitum heterophyllum* Wall ex Royle | H | Diterpene alkaloids (heterophylline A & B), Flavonoids, polysaccharide | Nyirimigabo et al., 2015 |
| 640 | *Aconitum violaceum* Jacq. | H | Alkaloid (aconitine),flavonoids | Rawat et al., 2013 |
| 641 | *Anemone obtusiloba* D. Don | H | Saponins (obtusilobinin, obtusilobin, obtusilobicinin) | Kaushik et al., 2016 |
| 642 | *Anemone rivularis* Ham. | H | Oleanane saponins (huzhangosides A- D), saponins, steviol glycosides | Mizutani et al., 1984; Yamaguchi et al., 1986 |
| 643 | *Anemone vitifolia* Buch.-Ham. ex DC. | H | Oleanane saponins | Bai et al., 2017 |
| 644 | *Clematis buchananiana* DC. | S | Triterpenoid saponins, flavonoids, coumarins, alkaloids | Hao et al., 2013 |
| 645 | *Clematis gouriana* Roxb. ex DC. | S | Steroids, emodins | Savithramma et al., 2011 |
| 646 | *Clematis grata* Wall | S | Triterpenoid saponins, flavonoids, coumarins, alkaloids | Hao et al., 2013 |
| 647 | *Clematis montana* Buch.-Ham. | S | Triterpenoid saponins, flavonoids, coumarins, alkaloids | Hao et al., 2013 |
| 648 | *Clematis orientalis* L. | S | Triterpenoid saponins ( leontoside,, cauloside D, kizutasaponin K12, clematibetoside C, hederacholichiside F,asperosaponin VI | Zhang et al., 2013 |
| 649 | *Delphinium brunonianum* Royle | H | Triterpene (β-amyrin), sterols (β-sitosterol, β-sitosterol glucoside), anthriscifoldine | Tripathee et al., 2012 |
| 650 | *Delphinium caeruleum* Jacq. | H | Diterpenoid alkaloid (caerunine), talitine B, delpheline, delbrunine, 4-acetyldelcosine | Wang et al., 1996 |
| 651 | *Delphinium denudatum* Wall.ex Hook. f & Thoms | H | Alkaloids, carbohydrate, steroids, phenolic groups, protein, amino acid, terpenoids, flavonoids | Mohanapriya and Vijaiyansiva, 2013 |
| 652 | *Delphinium pyramidale* Royle | H | Saponin, alkaloid | Vysochina et al., 2011 |
| 653 | *Delphinium vestitum* Wall. ex Royle | H | Diterpenoid alkaloids | Yunusov, 1991 |
| 654 | *Ranunculus arvensis* L. | H | Alkaloid, phenol, flavonoid, saponin | Hussain et al., 2011 |
| 655 | *Ranunculus sceleratus* L. | H | Sesquiterpene-lactones | Cuellar et al., 1998 |
| 656 | *Thalictrum alpinum* L. | H | Bisbenzyltetrahydroisoquinoline alkaloids, northalrugosidine | Naman et al., 2015 |
| 657 | *Thalictrum cultratum* Wall. | H | Aporphine-benzylisoquinoline alkaloids, thalicultratines A–K, tetrahydroprotoberberine-aporphine alkaloid, thalicultratine L, | Li et al., 2017 |
| 658 | *Thalictrum foliolosum* DC. | H | Bisbenzylisoquinoline alkaloid, thalifendine, berberine | Kumar et al., 2016 |
| 659 | *Thalictrum javanicum* Bl. | H | Phenolic compounds, condensed tannins (proanthocyanidins), flavonoids, ascorbic acid, saponin | Gurunathan et al., 2014 |
| **Rhamnaceae** | | | | |
| 660 | *Ziziphus mauritiana* Lam. | S | Tannins, saponins, phenols | Najafi,2013 |
| 661 | *Ziziphus xylopyrus* Willd. | S | Quercetin, Kempferol-4’-methylether, kempferol, cyclo peptide alkaloids (amphibineH, nummularine-K) | Sharma et al., 2009 |
| **Rosaceae** | | | | |
| 662 | *Agrimonia pilosa* Ledebour | H | Flavonols (tiliroside, 3-methoxy quercetin , quercitrin, quercetin), tannin ( agrimoniin) | Miyamoto et al., 1987; Jung and Park, 2007 |
| 663 | *Cotoneaster bacillaris* Wall. Kurz ex Lindl. | S | Ursolic acid, scopoletin, kaemferol, quercetrin, rutin | Rajendran et al., 2016 |
| 664 | *Fragaria* *indica* Andrews | H | Phenolic acids, ellagic acids, brevifolin carboxylic acid, flavonoids, tannins, organic acids, coumarins, polyprenols | Tomczyk et al., 2010; Zhu et al., 2015 |
| 665 | *Fragaria daltoniana* Gay. | H | Flavonol glycoside (quercetin 3-glucuronide), anthocyanins , Methyl butanoate, ethyl butanoate, methyl hexanoate, *cis*-3-hexenyl acetate, linalool | Azodanlou et al., 2003; Määttä-Riihinen et al., 2004 |
| 666 | *Fragaria nubicola* Lindley | H | Agrimoniin, proanthocyanidin, 5-(4-hydroxy-3-methoxyphenethyl)-7-methoxy-2H-chromen-3-ol, 5-(4-hydroxy-3-methoxyphenethyl)-4,7-dimethoxy-2H-chromen-3-ol , Methyl butanoate, ethyl butanoate, methyl hexanoate, *cis*-3-hexenyl acetate, linalool | Azodanlou et al., 2003; Naz et al., 2017 |
| 667 | *Fragaria rubiginosa* Lacaita | H | Methyl butanoate, ethyl butanoate, methyl hexanoate, *cis*-3-hexenyl acetate, linalool | Azodanlou et al., 2003; Azodanlou et al., 2003 |
| 668 | *Geum elatum* Wall. | H | Fatty acids (oleic acid, linoleic acid, 20:1n-7 acid), tocopherol, squalene | Matthaus and Özcan, 2014 |
| 669 | *Malus baccata* (L.) Borkh. Ssp *himalaica* (Maxim.) Likh. | T | Hlorogenic acid, quercetin 3-galactoside, phloridzin, cinnamic acid | Wang et al., 2013 |
| 670 | *Malus* *domestica* (Suckow) Borkh. | T | Phenolics, flavonoids, arnesol, cis & trans α-farnesene, ethyl hexanoate, ethyl 9-decenoate, ethyl 3-methylbutanoate, guaiacol and (Z) 3-hexen-1-ol | Genovese et al., 2004; Marinova et al., 2005 |
| 671 | *Potentilla* *argyrophylla* var. *atrosanguinea* (Lodd., G. Lodd. & W. Lodd.) Hook. f. | H | Chlorogenic acid, catechin, caffeic acid, p-coumaric acid, quercetin, Cyanidin-3-*O*-β-d-glucoside, Cyanidin-3-*O*-β-d-rutinoside | Kalia et al., 2008; Tomczyk and Latté, 2009 |
| 672 | *Potentilla cuneifolia* Bertol. | H | Organic acids, coumarins, polyprenols, tannins, flavonoids | Tomczyk et al., 2010 |
| 673 | *Potentilla gerardiana* Wall. ex Lindley | H | Organic acids, coumarins, polyprenols, tannins, flavonoids | Tomczyk et al., 2010 |
| 674 | *Potentilla lineata* Trevir. ex Reich. | H | Organic acids, coumarins, polyprenols, tannins, flavonoids | Tomczyk et al., 2010 |
| 675 | *Potentilla sundaica* (Blume) Kuntze | H | Organic acids, coumarins, polyprenols, tannins, flavonoids | Tomczyk et al., 2010 |
| 676 | *Prinsepia utilis* Royle | S | Limonene, 1-8-cineole, o-cymene, bergamal, cis-linalooloxide, cis-sabinene hydrate, linalool, rans-terpineol, 2-undeca-none, isomenthol, α-terpineol, 2-dodecanol and tridecanone | Pu et al., 2014 |
| 677 | *Armeniaca* *vulgaris* Lam. var. *vulgaris* | T | Linalool, ocimenol, α-terpineol, nerol, geraniol, cis & trans linalol oxide, γ-Decalactone, γ-dodecalactone, ethyl cinnamate | Genovese et al., 2004 |
| 678 | *Cerasus* *cerasoides* (Buch.-Ham. ex D. Don) S.Y. Sokolov | T | Alkaloids, steroids, terpenoids, flavonoids, phenolics, tannins, saponins, glycosides, carbohydrate, protein, amino acids | Joseph et al., 2016 |
| 679 | *Padus* *cornuta* (Wall. ex Royle) Carrière | T | Steroids, terpenoids, alkaloids, tannin, saponin, carbohydrate, protein | Purohit *et al*., 2017 |
| 680 | *Prunus domestica* L. | T | Phenolics, *leuco* anthocyanins, flavanols , Esters( butyl acetate,hexyl acetate, propyl acetate, ethyl butanoate, hexyl hexanoate | Hillis and Swain, 1959; Pino and Quijano, 2012 |
| 681 | *Amygdalus* *persica* L. | T | Ascorbic acid, citric acid, R- & γ-tocopherol, quinines | Carbonaro et al., 2002 |
| 682 | *Pyracantha crenulata* (D.Don.) M. Reom. | S | β- carotene, lycopene, condensed tannins, ascorbic acid | Pal et al., 2013 |
| 683 | *Pyrus communis* L. | T | Ascorbic, citric acids, R- & γ-tocopherol, quinines | Carbonaro et al., 2002 |
| 684 | *Pyrus pashia* Buch. & Ham. | T | Phenolic glycoside (4-*O*-*Z*-coumaroylarbutin), phenolic compounds, hydroquinone | He et al., 2015 |
| 685 | *Rosa brunonii* Lindley | S | Flavonoids, tannins, phlobatannis, triterpenes, quercetin, kaempferol & their glycosides | Kumar et al., 2009; Moteriya et al., 2015 |
| 686 | *Rosa centifolia* L. | S | Phenyl ethanol, geranyl acetate, geraniol, linalool, benzyl alcohol, benzaldehyde, nerol, citronellyl acetate | Jitendra et al., 2012 |
| 687 | *Rosa* *chinensis* Jacq. | S | Flavonoids, tannins, phlobatannis, triterpenes | Moteriya et al., 2015 |
| 688 | *Rosa macrophylla* Lindl. | S | Flavanoids, tannins, phlobatannis, triterpenes, fatty acids | Moteriya et al., 2015; Jain et al., 2018 |
| 689 | *Rosa moschata* Herm. | S | Flavanoids, tannins, phlobatannis, triterpenes, quercetin | Moteriya et al., 2015; Ouerghemmi et al., 2016 |
| 690 | *Rosa sericea* Lindle | S | Flavanoids, tannins, phlobatannis, triterpenes | Moteriya et al., 2015 |
| 691 | *Rubus biflorus* Buch. Ham. Ex Sm. | S | Octadeca-9,12-dienoic acid ethyl ester, diphenyl carbonate, 2-propenoic acid, 3-(4-methoxyphenyl)-2-ethylhexyl ester | Kou et al., 2008 |
| 692 | *Rubus ellipticus* Sm. | S | Phenolics, flavonoids, monomeric anthocyanins, ascorbic acid, β-carotene | Badhan et al., 2015 |
| 693 | *Rubus niveus* Thunb | S | Phenolics, flavonoids, monomeric anthocyanins, ascorbic acid, β-carotene | Badhan et al., 2008 |
| 694 | *Sorbaria tomentosa* (Lindl.) Rehder | S | D- Sorbitol | Plouvier,1963 |
| **Rubiaceae** | | | | |
| 695 | *Breonia* *chinensis* (Lam.) Capuron | T | Saponins, terpenes, sesquiterpenes glycosides, alkaloids | Umachigi et al., 2007 |
| 696 | *Catunaregam spinosa* (Thunb.) Tirveng. | S | Triterpenoid saponins (catunarosides A–D, swartziatrioside, aralia-saponin V, araliasaponin) | Gao et al., 2011 |
| 697 | *Galium* *spurium* subsp. *africanum* Verdc. | H | Anthraquinones, iridoids, alkanes, flavonoids, tannins, polyphenolic acids | Bokhari et al., 2013 |
| 698 | *Haldina cordifolia* (Roxb.) Ridsdale | T | 7-hydroxycoumarin-1, 7-β-Dglucosylcoumarin-2 | Sharma et al., 2012 |
| 699 | *Himalrandia tetrasperma* (Roxb.) Hook.f. | S | Alkaloids, saponins, terpenoids, flavonoids, steroids,reducing sugars | Salman et al., 2015 |
| 700 | *Hymenodictyon orixense* (Roxb.) Mabb. | T | Iridoid glycoside (loganin), coumarins (scopoletin, scopolin, hymexelsin, scopoletin 7-*O*-β-D-xylopyranosyl-(1→6)-β-D-glucopyranoside | Suchaichit et al., 2017 |
| 701 | *Ixora arborea* Roxb. ex Sm. | T | Betulin, erythrodiol, lupeol, stigmasterol | Aktar et al., 2009 |
| 702 | *Ixora coccinea* L. | T | Aromatic acrid oil, tannin, fatty acids, flavonols, kaemferol, quercetin, proanthrocyanidines, phenolic acids, ferulic acids, cyanidins, flaconboides | Maniyar et al., 2010 |
| 703 | *Mitragyna parvifolia* (Roxb.) Korth | T | Alkaloids, triterpenoids, flavonoids | Gong et al., 2012 |
| 704 | *Pavetta indica* L. | T | Isovaleric acid, isovaleraldehyde, armomadandrene, pavonene, α-terpenene, azulene, pavonenol, β-pinene, β-eudesmol, tricyclene | Prasad et al., 2011 |
| 705 | *Rubia cordifolia* L. | C | Anthraquinone glycoside (l-acetoxy-6-hydroxy-2-methyl anthraquinone-3- O--rhamnosyl (1 → 4)—glucoside), furomollugin, mollugin, dehydro-lapchone | Gupta et al., 1999 |
| 706 | *Spermadictyon suaveolens* Roxb | T | Flavonoids, tannins, coumarins, procyanidins | Ajaib et al., 2014 |
| **Rutaceae** | | | | |
| 707 | *Aegle marmelos* (L.) Correa ex Roxb | T | Steroids, terpenoids, flavonoids, phenolic compounds, lignin, fats, carbohydrates, saponins,oils,  inulin,  proteins, cardiac glycosides | Rajan et al., 2011 |
| 708 | *Boenninghausenia albiflora*  (Hook.) Rchb. ex Meisn. L | H | Terpenoids, alkaloids, coumarins | Khulbe and Sati, 2009 |
| 709 | *Citrus aurantifolia* (Christm.) Swingle | T | 5-geranyloxypsoralen, 5-geranyloxy-7-methoxycoumarin, 5,7-dimethoxycoumarin, 5-methoxypsoralen,5,8-dimethoxypsoralen | Sandoval-Montemayor et al., 2012 |
| 710 | *Citrus limon* (L.) Burm. f. | T | Limonene, β-pinene, γ –terpinene | Tampieri et al., 2005 |
| 711 | *Glycosmis pentaphylla* (Retz.) DC. | S | Alkaloids, flavonoids, terpenes, sterols | Sreejith et al., 2012 |
| 712 | *Murraya koenigii* (L.) Spreng | T | Linalool, elemol, geranyl acetate, myrcene, allo-ocimene, α-terpinene, (E)-β-ocimene, neryl acetate, monoterpenes, monoterpene hydrocarbons, oxygenated sesquiterpenes, sesquiterpenes hydrocarbon | Rajendran et al., 2014 |
| 713 | *Murraya paniculata* (L.) Jack. | T | Phenolics, flavonoids | Gautam et al., 2012 |
| 714 | *Skimmia anquetilia* N. P.Taylor and Airy Shaw | S | Linalool, geraniol, pinene, scopoletin, skimmianine, umbelliferone[ | Kumar et al., 2012 |
| 715 | *Zanthoxylum armatum* DC. | T | Alkaloids, flavonoids, sterols, phenolics, lignins, coumarins, terpenoids, | Singh and Singh, 2011 |
| 716 | *Zanthoxylum rhetsa (*Roxb) DC. | T | Terpenoid, xanthyletin, sesamin, alkaloids, flavonoids, sabinene | Alphonso and Saraf, 2012 |
| **Salicaceae** | | | | |
| 717 | *Casearia elliptica* Willd. | T | Clerodane diterpenoids, sesquiterpenoids, phenylpropanoids | Xia et al., 2015 |
| 718 | *Flacourtia indica* (Burm. f.) Merr. | T | Flavanoids, phenolics, caffeic acid, ferulic acid, p-coumaric acid | Ndhlala et al., 2007 |
| **Santalaceae** | | | | |
| 719 | *Viscum articulatum* Burm.f. | H | Flavonoids, triterpenoids, organic acids, flavanones, benzenoids, inositol | Leu et al., 2004 |
| **Sapindaceae** | | | | |
| 720 | *Aesculus indica* Colebr. ex (Cambess.) Hook. | T | Flavonoids, saponins, coumarins, tannins | Bibi et al., 2012 |
| 721 | *Cardiospermum halicacabum* L. | H | Phenol, tannins, saponins, steroids, sugars, flavonoids, terpenoids (Benzene, acetone), amino acids | Viji and Murugesan, 2010 |
| 722 | *Litchi chinensis* Sonn. | T | Flavonoids ( luteolin, epicatechin, kaempferol 3-O-b-glucoside, kaempferol 3-O-a-rhamnoside, procyanidin A_2_, rutin), anthocyanins, flavones, flavonols, chalcones, dihydrochalcones, dihydroflavonols, isoflavonoids, | Wen et al., 2014 |
| 723 | *Sapindus mukorossi* Gaertn. | T | Saponins, sugars, mucilage | Ibrahim et al., 2006 |
| **Sapotaceae** | | | | |
| 724 | *Diploknema butyracea* (Roxb.) H. J. Lam | T | Saponins, prosapogenins | Saha et al., 2010 |
| 725 | *Madhuca longifolia* (Koen.) Mac. | T | Sapogenins triterpenoids, saponins, steroids, flavonoids | Jyothi and Seshagiri, 2012 |
| **Saxifragaceae** | | | | |
| 726 | *Bergenia ciliata* Raizada | H | Bergenin, catechin, gallic acid,steroid, terpenoids, saponins, flavonides, anthraquinone | Dhalwal et al., 2008; Uddin et al., 2012 |
| 727 | *Bergenia ligulata* (Wall.)Engl. | H | Bergenin, catechin, gallic acid, β-sitosterol, β-sitosterol-D-glucoside, (+) –afzelechim, leucocyanidin, methyl gallate, paashaanolactone | Reddy et al.,1999; Dhalwal et al., 2008 |
| 728 | *Bergenia stracheyi* (Hk. F. & Th.) Engler | H | Bergenins, bergenin derivatives (  bergecins A & B) | Siddiq et al., 2012 |
| **Scorphulariaceae** | | | | |
| 729 | *Buddleja asiatica* Lour. | S | Triterpenoid Saponin, iridoid, flavonoid glycosides | Houghton, P. J. 1984; Houghton and Mensah, 1999 |
| 730 | *Verbascum thapsus* L. | H | Polysaccharides, iridoid glycosides, harpagoside, harpagide, aucubin,flavonoids, methylguercitin, hesperedin, verbascoside, saponins, volatile oils | Turker and Gure, 2005 |
| **Selaginaceae** | | | | |
| 731 | *Selaginella bryopteris* (L.) Bak. | Pter | Amentoflavone & hinokiflavone derivatives, alkaloids, flavonoids, tannins, saponins,terpenoids, steroid | Antony and Thomas, 2011 |
| **Smilacaceae** | | | | |
| 732 | *Smilax aspera* L. | S | Steroidal saponins | Belhouchet et al., 2008 |
| **Solanaceae** | | | | |
| 733 | *Datura* *suaveolens* Humb. & Bonpl. ex Willd. | S | Scopolamine, hyoscyamine,atropine | Parker et al., 2007 |
| 734 | *Capsicum annuum* L. | H | Phenolics, flavonoids,phenolic acids, capsaicinoids, p-coumaryl, caffeoyl, 3,4-dimethoxycinnamoyl glucoside | Materska and Perucka, 2005 |
| 735 | *Datura inoxia* Mill. | H | Alkaloids (L-atropine, L-scopolamine, atropine-d3 , atropine) | Kintz et al., 2006 |
| 736 | *Datura stramonium* L. | H | Tigloidin, aposcopolamine, apoatropin, hyoscyamine N-oxide, scopolamine N-oxide, 6α-ditigloyloxytropane, 7-hydroxyhyoscyamine, saponins, tannins, glycosides, Alkaloids hyoscyamine, scopolamine | Ajungla et al., 2009; Soni et al., 2012 |
| 737 | *Hyoscyamus niger* L. | H | Coumarinolignans (cleomiscosin A methyl ether, cleomiscosin A & B, cleomiscosin A-9′-acetate, cleomiscosin B-9′-acetate) | Begum et al., 2010 |
| 738 | *Solanum* *lycopersicum* L. | H | Glycoalkaloid ( dehydrotomatine, α-tomatine), aglycones | Friedman, 2004 |
| 739 | *Nicandra physalodes* Gaertn. | H | Alkaloid (3-O-α -D-glucopyranosyl-calystegine) | Jung et al., 2006 |
| 740 | *Nicotiana plumbaginifolia* Viviani | H | N-2-(5-chloro-pyridyl) aminomethylene bisphosphonic acid | Forlani et al., 2000 |
| 741 | *Nicotiana rustica* L. | H | Alkaloid (nicotine) | Morita et al., 2009 |
| 742 | *Physalis angulata* L. var. angulata | H | Steroids(physalins (D, I, G, K, B, F, E), physagulins (E, F, G), anolides, flavonoids | Bastos et al., 2006 |
| 743 | *Physalis divaricata* D. Don | H | Steroidal lactones (withanolides), quercetin, luteolin | Namjooyan et al., 2007; Namjoyan et al., 2016 |
| 744 | *Solanum anguivi* Lam. | H | Steroidal alkaloids, solavetivone, solafuranone, scopoletin, N-(*p-trans*-coumaroyl)tyramine, N-*trans*-feruloyltyramine | Barbosa Filho et al., 1991; Syu et al., 2001 |
| 745 | *Solanum* *granulosoleprosum* Dunal | S | α-terpinolene, α-phellandrene, p-cymene, β-pinene, α-humulene, humulene epoxide II, caryophyllene oxide, methyl salicylate, β-caryophyllene | Essien et al., 2012 |
| 746 | *Solanum ferox* L. | H | Steroidal alkaloids | Barbosa Filho et al., 1991 |
| 747 | *Solanum melongena* L. | H | Anthocyanin ( delphinidin-3-rutinoside), phenolis, flavonoids | Sadilova et al., 2006; Nisha et al., 2009 |
| 748 | *Solanum nigrum* L. | H | Glycoalkaloids, glycoproteins, polysaccharides, gallic acid, catechin, protocatechuic acid, caffeic acid, epicatechin, rutin, naringenin | Jain et al., 2011 |
| 749 | *Solanum* *rudepannum* Dunal | H | Triacontane derivatives, chlorogenone, neochlorogenone, isoflavonoid sulfate, steroidal glycosides, 22-β-*O*-spirostanol oligoglycosides, 26-β-*O*-glucosidase | Balachandran et al., 2012 |
| 750 | *Solanum tuberosum* L. | H | Glycoalkaloids (α –chaconine,α –solanine), anthocyanins, flavonoids (rutin, kaempferol ‐3‐rutinoside), quercetin‐ rhamnose‐glucosides, phenolic acids, pelagonidin‐3‐ (*p*‐coumaroyl ‐rutinoside)‐5‐glucoside | Lewis et al., 1998; Friedman, 2004, |
| 751 | *Solanum virginianum* L. | H | Steroidal alkaloids (solanacarpine, solanacarpidine, solancarpine, solasonine, solamargine), caffeic acid, coumarins (aesculetin,aesculin), steroids carpesterol, diosgenin, campesterol, daucosterol, triterpen (cycloartanol, cycloartenol) | Hussain et al., 2012 |
| 752 | *Withania somnifera* Dunal | H | Alkaloids (isopelletierine, anaferine), steroidal lactones (withanolides, withaferins), saponins (sitoindoside VII & VIII), withanolides (sitoindoside IX & X) | Mishra et al., 2000 |
| **Symplocaceae** | | | | |
| 753 | *Symplocos paniculata* (Thunb.) Miq. | T | Triterpenoids, flavonoids, lignans, steroids, alkaloids, triterpenoid saponins, symplocososides L−S | Fu et al., 2006; Semwal et al., 2011 |
| **Taxaceae** | | | | |
| 754 | *Taxus wallichiana* (Zucc.) Pilger | T | Taxane esters (taxol, cephalomannine), lignans, flavonoids, steroids, sugar | McLaughlin et al., 1981; Parmar et al., 1999 |
| **Tectariaceae** | | | | |
| 755 | *Tectaria* *gemmifera* (Fée) Alston | Fn | Octadec-9-enoic acid (oleic acid), n-hexadecanoic acid (palmitic acid), octadecanoic acid (stearic acid), Di-n-octyl phthalate, hexadecanoic acid methyl ester, hexadecanoic acid ethyl ester | Dubal et al., 2013 |
| **Theaceae** | | | | |
| 756 | *Camellia sinensis*(L.) Kunize | S | Catechins, caffeine, flavonols (myricetin, caempherol, quercetin), proanthocyanidins, | Perva-Uzunalić et al., 2006 |
| **Thelypteridaceae** | | | | |
| 757 | *Cyclosorus* *aridus* (D. Don) Tagawa | Fn | Terpenoids, phenolics, flavonoids, alkaloids | Ho et al., 2011 |
| **Thymelaeaceae** | | | | |
| 758 | *Daphne papyracea* Wall. ex Steud. | S | Coumarins, flavonoids, lignans, steroids | Sovrlić *et al*., 2015 |
| **Tiliaceae** | | | | |
| 759 | *Corchorus* *tridens* L. | H | Triterpenoids | Khan et al., 2015 |
| **Ulmaceae** | | | | |
| 760 | *Holoptelea integrifolia* (Roxb.) | T | Alkaloids, flavonoids, saponins, tannins, terpenoids (2,4 dinitrophenylhydrazine), glycosides, steroids, anthraquinones | Shrinivas et al., 2009 |
| 761 | *Ulmus wallichiana* Planch. | T | Quercetin ( quercetin-6-C-A-D-glucopyranoside | Siddiqui et al., 2011 |
| **Urticaceae** | | | | |
| 762 | *Boehmeria rugulosa* Wedd. | T | Phenolics, flavonoids | Sharma et al., 2017 |
| 763 | *Debregeasia longifolia* (Burm. f.) Wedd. | S | Phenolics,flavonoids,flavonols | Seal and Chaudhuri, 2015 |
| 764 | *Girardinia diversifolia* (Link) Friis | S | Cardiac glycosides, saponins, tannins, sterols | Njogu, 2007 |
| 765 | *Pouzolzia zeylanica* (L.) Benn. & R. Br | H | Alkaloid, flavonoids, phenolics | Hossain et al., 2017 |
| 766 | *Urtica dioica* L. | S | Lignans (+)-neoolivil, (-)-secoisolariciresinol, dehy-drodiconiferyl alcohol, isolariciresinol, pinoresinol, 3,4-divanillyltetrahydrofuran | Kanter et al., 2005 |
| 767 | *Urtica hyperborea* Jacq. | S | Terpenoids, flavonoids, lignans, sterols, polyphenols | Ibrahim et al., 2018 |
| 768 | *Urtica mairei* H. Lew. | S | Terpenoids, flavonoids, lignans, sterols, polyphenols | Ibrahim et al., 2018 |
| **Verbanaceae** | | | | |
| 769 | *Duranta* *repens* L. | S | Isoprenylated flavonoids, 5-hydroxy-3,6,7,49-tetramethoxyflavone, rosenonolactone, 6,7-dimethoxycoumarin, 5a,8a-epidioxyergosta-6,22-dien-3b-ol, 5a,8a-epidioxyergosta-6,9(11), 22-trien-3b-ol | Anis et al., 2002 |
| 770 | *Lantana camara* L. | S | Mono- & sesquiterpenes, triterpenes, iridoid glycosides, furanonaphthoquinones, flavonoids, phenyl ethanoid glycosides, steroids, β-sitosterol, campesterol, stigmasterol, β sitosterol glucoside | Ghisalberti, 2000 |
| 771 | *Lantana indica* Roxb | S | Trans-caryophyllene, α-selinene, globulol, trans-caryophyllene oxide, α-guaiene, valencene, humulene,β-eudesmene, oleanolic acid, 3- ketooleanolic acid, (+)-24-hydroxy-3-oxoolean-12-en28-oic acid, 3 β,24-dihydroxyolean-12-en-28-oic acid,3,24-dioxo-olean-12-en-28-oic acid | Hussain et al., 2011 |
| 772 | *Lippia alba* (Mill.) N.E. Br. ex. Britton & P. Wilson | H | Monoterpenes, citral, β-myrcene,limonene, chemotype I & II, carvone | Vale et al., 1999 |
| 773 | *Phyla nodiflora* (L.) Greene | H | 3,7,4',5'-tetrahydroxy-3'-methoxyflavone, nodifloretin, 4' –hydroxywogonin, onopordin, cirsiliol, 5,7,8,4' -tetrahydroxy-3' –methoxyflavone, eupafolin, hispidulin, larycitrin, 𝛽-sitosterol | Lin et al., 2014 |
| **Viburnaceae** | | | | |
| 774 | *Viburnum nervosum* D.Don | T | Flavonoids, iridoids glycosides, sesquiterpenes, vibsane diterpenes, lupan type triterpene (butilinol, oleanolic acid, ursolic acid, butilin, β-sitosterol, butilinic acid, α-amyrin, germanicol | Awan et al., 2013; Shah et al., 2014 |
| **Violaceae** | | | | |
| 775 | *Viola betonicifolia* Smith | H | Alkaloid, flavonoids, saponins, tannins, phenols, sterols, triterpenoids, proteins, phenolic compounds | Muhammad et al., 2012; Muhammad et al., 2012 |
| 776 | *Viola biflora* L. | H | Cyclotides | Herrmann et al., 2008 |
| 777 | *Viola canescens* Wall. | H | Alkaloids, phenolic compounds, tannins, saponins, phytosterols, flavonoids | Muhammad et al., 2012 |
| 778 | *Viola odorata* L. | H | Cyanidin 3-glycosides, 3-O-[-L-rhamnopyranosyl-(1→6)-β-D glucopyranoside], cycloViolacin O_1_-O_11,_  flavonoids (isorhamnetin, luteolin) | Herrmann et al., 2008; Muhammad et al., 2012 |
| 779 | *Viola pilosa* Blume | H | Alkaloids, proteins, tannins, carbohydrates, sterols, flavonoids, saponins, fats & oils | Bakht et al., 2017 |
| **Vitaceae** | | | | |
| 780 | *Ampelocissus latifolia* (Roxb.) Planch. | C | Tannins, terpenoids, saponins, flavonoids, carbohydrates, anthraquiones, alkaloids, glycosides | Chaudhuri and Ray, 2015 |
| 781 | *Cissus* *carnosa* Lam. | S | Alkaloid, flavonoids, tannins, saponins, phenolic compounds | Perumal et al., 2012 |
| 782 | *Cissus quadrangularis* L. | S | Flavanoids, triterpenoids,stilbene derivatives, resveratrol, piceatannol, pallidol perthenocissin, phytosterols, ascorbic acid, triterpene, β-sitosterol, ketosteroid | Mishra et al., 2010 |
| **Zingiberaceae** | | | | |
| 783 | *Curcuma aromatica* Salisb. | H | Xanthorrhizol, 1H-3a, 7–methanoazulene, curcumen, p-cymene, ,8-cineole | Singh et al., 2002; Choochote et al., 2005 |
| 784 | *Curcuma longa* L. | H | Ar-turmerone, ar-turmerol, curcumin (diferuloylmethane), demethoxycurcumin, bisdemethoxycurcumin, tumerone, atlantone, zingiberone, sugars, proteins, resins | Singh et al., 2002, Jurenka, 2009 |
| 785 | *Elettaria cardamomum* L. | H | 1,8-cineole, α-terpineol, borneol, camphor, limonene, α-terpenyl acetate, α-pinene | Kaushik et al., 2010 |
| 786 | *Hedychium spicatum* Buch.-Ham. ex Sm | H | 1,8-cineol, eudesmol, cubenol, spathulenol, α-cadinol | Mishra et al., 2016 |
| 787 | *Roscoea purpurea* J. E. Sm. | H | Protocatechuic acid, syringic acid, ferulic acid, rutin, apigenin, kaempferol | Srivastava et al., 2015 |
| 788 | *Zingiber officinale Rosc.* | H | Monoterpenoids ( 𝛽 -phellandrene, (+)-camphene, cineole, geraniol, curcumene, citral, terpineol, borneol), sesquiterpenoids (a-zingiberene, 𝛽 –sesquiphellandrene, 𝛽-bisabolene, (E-E)-a-farnesene, arcurcumene, zingiberol) | Ali et al., 2008 |
| **Zygophyllaceae** | | | | |
| 789 | *Tribulus terrestris* L. | H | Saponins, flavonoids, glycosides, alkaloids, tannins | Chhatre et al., 2014 |

Footnote: H-Herb; S- Shrub, T- Tree, C- Climber, Fn- Fern, Fg-Fungi; Pter- Pteridophyte

**References Supplimentary table 1**

Abdul Kalam, A., Sulaiman, W., Azizi, W. M., Labu, Z. K., & Zabin, S. (2013). An overview on phytochemical, anti-inflammatory and anti-bacterial activity of *Basella alba* leaves extract. *Middle-East Journal of Scientific Research*, *14*(5),650-655.

Abdullahi, A., Hamzah, R. U., Jigam, A. A., Yahya, A., Kabiru, A. Y., Muhammad, H., ... & Kolo, M. Z. (2012). Inhibitory activity of xanthine oxidase by fractions Crateva adansonii. *Journal of Acute Disease*, *1*(2), 126-129.

Abha, S., Swati, V., & Shukla, R. K. (2013). Preliminary phytochemical screening, antibacterial and nitric oxide radical scavenging activities of Rinwardtia indica leaves extract. *Int J PharmTech Res*, *5*(4), 1670-1678.

Abubacker, M. N., & Devi, P. K. (2014). In vitro antifungal potentials of bioactive compound oleic acid, 3-(octadecyloxy) propyl ester isolated from Lepidagathis cristata Willd.(Acanthaceae) inflorescence. *Asian Pacific journal of tropical medicine*, *7*, 190-193.

Agarwal, R. *Phytochemical analysis of some plants of family Lamiaceae and Amaranthaceae and screening of their biological activities* (Doctoral dissertation, GB Pant University of Agriculture and Technology, Pantnagar-263145 (Uttarakhand)).

Ahmad, B., Khan, M. R., Shah, N. A. & Khan, R. A. (2013). In vitro antioxidant potential of *dicliptera roxburghiana*. *BMC Complementary and Alternative Medicine*, *13*(1), 140.

Ahmad, I., & Beg, A. Z. (2001). Antimicrobial and phytochemical studies on 45 Indian medicinal plants against multi-drug resistant human pathogens. *Journal of ethnopharmacology*, *74*(2), 113-123.

Ahmad, M. K., Mahdi, A. A., Shukla, K. K., Islam, N., Jaiswar, S. P., & Ahmad, S. (2008). Effect of *Mucuna pruriens* on semen profile and biochemical parameters in seminal plasma of infertile men. *Fertility and Sterility*, 90(3), 627-635.

Ahmad, M., Kaloo, Z. A., Ganai, B. A., Ganaie, H. A., & Singh, S. (2016). Phytochemical screening of meconopsis *aculeata royle* an important medicinal plant of Kashmir Himalaya: A perspective. *Research Journal of Phytochemistry*, *10*, 1-9.

Ahmad, N. S., Farman, M., Najmi, M. H., Mian, K. B., & Hasan, A. 2008. Pharmacological basis for use of *Pistacia integerrima* leaves in hyperuricemia and gout. *Journal of ethnopharmacology*, *117*(3), 478-482.

Ahmad, S., Khader, J. A., Ullah, R., AbdEIslam, N. M., ud Din, S., Khan, R., & Mehmood, S. (2012). Phytochemical analysis and antimicrobial activity of *Persea duthiei*. *African Journal of Pharmacy and Pharmacology*, 6(48), 3302-3304.

Ahmad, V. U., Ali, Z., Zahid, M., Alam, N., Saba, N., Khan, T., Qaisar, M., & Nisar, M. (2000). Phytochemical study of Salvia moorcroftiana. *Fitoterapia*, *71*(1), 84-85.

Ahmed, D., Saeed, R., Shakeel, N., Fatima, K., & Arshad, A. (2015). Antimicrobial activities of methanolic extract of *Carissa opaca* roots and its fractions and compounds isolated from the most active ethyl acetate fraction. *Asian Pacific Journal of Tropical Biomedicine*, *5*(7), 541-545.

Ahmed, Z. S. (2016). Investigation of in-vitro antioxidant potential in *Crotalaria verrucosa* along with Identification and Quantification of its polyphenolic compounds.

Ahmed, Z. U., Bithi, S. S., Khan, M. M. R., Hossain, M. M., Sharmin, S. & Rony, R. (2014). Phytochemical screening, antioxidant and cytotoxic activity of fruit extracts of *Calamus tenuis* Roxb." *Journal of Coastal Life Medcine*, *2*(8), 645-650.

Aiyelaagbe, O. O. & Paul, M. (2009). Phytochemical screening for active compounds in Mangifera indica leaves from Ibadan, Oyo state. *Plant Sci Res*, *2*(1), 11-13.

Ajaib, M., Hanif, U., & Khalid, S. (2014). *Spermadictyon suaveolens*: A potential natural antimicrobial and antioxidant source. *International Journal of Phytomedicine*, *6*(2), 256267.

Ajungla, L., Patil, P. P., Barmukh, R. B., & Nikam, T. D. (2009). Influence of biotic and abiotic elicitors on accumulation of hyoscyamine and scopolamine in root cultures of *Datura metel* L. *Indian Journal of Biotechnology*, *8*, 317-322.

Akinsulire, O. R., Aibin, I. E., Adenipekun, T., Adelowotan, T., & Odugbemi, T. (2007). In vitro antimicrobial activity of crude extracts from plants *Bryophyllum pinnatum* and *Kalanchoe crenata*. *African Journal of Traditional, Complementary and Alternative Medicines*, *4*(3):, -344.

Aktar, F., Kaisar, M. A., Kabir, A. H., Hasan, C. M., & Rashid, M. A. (2009). Phytochemical and Biological Investigations of *Ixora arborea* Roxb. *Dhaka University Journal of Pharmaceutical Sciences*, *8*(2), 161-166.

Akter, S., Hossain, M. M., Ara, I., & Akhtar, P. (2014). Investigation of in vitro antioxidant, antimicrobial and cytotoxic activity of *Diplazium esculentum* (Retz). Sw. *International Journal of Advances in Pharmacy, Biology and Chemistry*, *3*, 723-733.

Alam, F., & Saqib, Q. N. (2015). Pharmacognostic standardization and preliminary phytochemical studies of Gaultheria trichophylla. *Pharmaceutical biology*, *53*(12), 1711-1718.

Al-Bayati, F. A. (2009). Isolation and identification of antimicrobial compound from *Mentha longifolia* L. leaves grown wild in Iraq. *Annals of clinical microbiology and antimicrobials*, *8*(1), 20.

Ali, B. H., Blunden, G., Tanira, M. O., & Nemmar, A. (2008). Some phytochemical, pharmacological and toxicological properties of ginger (*Zingiber officinale* Roscoe): a review of recent research. *Food and chemical Toxicology*, *46*(2), 409-420.

Ali, H., Houghton, P. J., & Soumyanath, A. (2006). α-Amylase inhibitory activity of some Malaysian plants used to treat diabetes; with particular reference to *Phyllanthus amarus.* *Journal of ethnopharmacology*, *107*(3), 449-455.

Ali, M. S., Ibrahim, S. A., Ahmed, S. and Lobkovsky, E. (2007). Guaiane sesquiterpene lactones from Salvia nubicola (Lamiaceae). *Chemistry & Biodiversity*, *4*(1), 98-104.

Ali, M., Latif, A., Zaman, K., Arfan, M., Maitland, D., Ahmad, H., & Ahmad, M. (2014). Anti-ulcer xanthones from the roots of *Hypericum oblongifolium* Wall. *Fitoterapia*, *95*, 258-265.

Alphonso, P., & Saraf, A. (2012). Chemical profile studies on the secondary metabolites of medicinally important plant *Zanthoxylum rhetsa* (Roxb.) DC using HPTLC. *Asian Pacific Journal of Tropical Biomedicine*, *2*(3), 1293-1298.

Al-Snafi, A. E. 2013. The Pharmacological importance of *Benincasa hispida*. A review. *Int Journal of Pharma Sciences and Research*, *4*(12), 165-170.

Al-Snafi, A. E. 2015. Bioactive components and pharmacological effects of *Canna indica*-An Overview. *International Journal of Pharmacology and toxicology*, *5*(2), 71-75.

Al-Sultan, S. I., Hussein, Y. A., & Hegazy, A. (2003). Toxicity of *Anagallis arvensis* plant. *Pakistan Journal of Nutrition*, *2*(3), 116-122.

Amos, S., Adzu, B., Binda, L., Wambebe, C., & Gamaniel, K. (2001). Neuropharmacological effect of the aqueous extract of *Sphaeranthus senegalensis* in mice. *Journal of ethnopharmacology*, *78*(1), 33-37.

Amresh, G., Zeashan, H., RAO, V., & Singh, P. N. (2007). Prostaglandin mediated anti-inflammatory and analgesic activity of *Cissampelos pareira*. *Acta Pharmaceutica Sciencia*, *49*(2).

Andrade‐Neto, V. F., Brandão, M. G., Oliveira, F. Q., Casali, V. W., Njaine, B., Zalis, M. G. Oliveira, L.A. & Krettli, A. U. (2004). Antimalarial activity of *Bidens pilosa* L.(Asteraceae) ethanol extracts from wild plants collected in various localities or plants cultivated in humus soil. *Phytotherapy Research*, *18*(8), 634-639.

Anis, I., Ahmed, S., Malik, A., Yasin, A., & Choudary, M. I. (2002). Enzyme inhibitory constituents from *Duranta repens*. *Chemical and pharmaceutical bulletin*, *50*(4), 515-518.

Anitha, M., Paulpriya, K., Muthukumarasamy, S., & Mohan, V. R. (2012). GC-MS Analysis of Bioactive Components of *Cynoglossum zeylanicum* (Vahl Ex Hornem) Thunb. Ex. Lehm.(Boraginaceae). *Current Pharma Research*, *2*(4), 615.

Antony, R., & Thomas, R. (2011). A mini review on medicinal properties of the resurrecting plant *Selaginella bryopteris* (Sanjeevani). *International Journal of Pharmacy & Life Sciences*, *2*(7), 933-939.

Anwer, N., Waqar, M. A., Iqbal, M., Mushtaq, M., & Sobia, A. (2013). Phytochemical analysis, free radical scavenging capacity and antimicrobial properties of *Impatiens bicolor* plant. *International Food Research Journal*,  *20*(1).

Aoki, K., Cortés, A. R., del Carmen Ramirez, M., Gómez-Hernández, M., & López-Muñoz, F. J. (2008). Pharmacological study of antispasmodic activity of *Mirabilis jalapa* Linn flowers. *Journal of ethnopharmacology*, *116*(1), 96-101.

Arfan, T. U. R. M., Mohammad, I. K. G., & Choudhary, M. I. (2011). In-vitro pharmacological investigations of aerial parts of *indigofera heterantha*. *Journal of Medicinal Plants Research*, *5*(24), 5750-5753.

Arora, S., & Kumar, G. (2018). Phytochemical screening of root, stem and leaves of *Cenchrus biflorus* Roxb. *Journal of Pharmacognosy and Phytochemistry*, *7*(1), 1445-1450.

Arya, V., Yadav, S., Kumar, S., & Yadav, J. P. 2010. Antimicrobial activity of *Cassia occidentalis* L (leaf) against various human pathogenic microbes. *Life Sci Med Res*, *9*(1), 12.

Asakawa, Y., Matsuda, R., & Takemoto, T. (1982). Mono-and sesquiterpenoids from Hydrocotyle and *Centella* species. *Phytochemistry*, *21*(10), 2590-2592.

Ashidi, J. S., Houghton, P. J., Hylands, P. J., & Efferth, T. (2010). Ethnobotanical survey and cytotoxicity testing of plants of South-western Nigeria used to treat cancer, with isolation of cytotoxic constituents from *Cajanus cajan* Millsp. leaves. *Journal of ethnopharmacology*, *128*(2), 501-512.

Ashour, M. L., & Wink, M. (2011). Genus *Bupleurum*: a review of its phytochemistry, pharmacology and modes of action. *Journal of pharmacy and pharmacology*, *63*(3), 305-321.

Ashraf, A., Sarfraz, R. A., Rashid, M. A., & Shahid, M. (2015). Antioxidant, antimicrobial, antitumor, and cytotoxic activities of an important medicinal plant (*Euphorbia royleana*) from Pakistan. *journal of food and drug analysis*, *23*(1), 109-115.

Ata, A., Kalhari, K. S. & Samarasekera, R. (2009). Chemical constituents of *Barleria prionitis* and their enzyme inhibitory and free radical scavenging activities. *Phytochemistry Letters*, *2*(1), 37-40.

Awan, Z. A., Rehman, H. U., Minhas, F. A., & Awan, A A. (2013). Antiplasmodial activity of compounds isolated from *Viburnum nervosum*. *International Journal of Pharmaceutical Science Invention, 2*(5), 19-24.

Awasthi, L. P., & Verma, H. N. (2006). *Boerhaavia diffusa*–A wild herb with potent biological and antimicrobial properties. *Asian Agri-History*, *10*(1), 55-68.

Ayyanar, M., & Subash-Babu, P. (2012). *Syzygium cumini* (L.) Skeels: A review of its phytochemical constituents and traditional uses. *Asian Pacific journal of tropical biomedicine*, *2*(3), 240-246.

Azodanlou, R., Darbellay, C., Luisier, J. L., Villettaz, J. C., & Amadò, R. (2003). Quality assessment of strawberries (*Fragaria* species). *Journal of Agricultural and Food Chemistry*, *51*(3), 715-721.

Badhani, A., Rawat, S., Bhatt, I. D., & Rawal, R. S. (2015). Variation in chemical constituents and antioxidant activity in yellow Himalayan (*Rubus ellipticus* smith) and hill raspberry (*Rubus niveus* thunb.). *Journal of food biochemistry*, *39*(6), 663-672.

Badoni, R., Semwal, D. K., & Rawat, U. (2010). Fatty acid composition and antimicrobial activity of *Celtis australis* L. fruits. *Journal of Scientific Research*, *2*(2), 397-402.

Bai, C., Ye, Y., Feng, X., Bai, R., Han, L., Zhou, X., Yang, X., Tu, P., & Chai, X. (2017). Anti-Proliferative Effect of Triterpenoidal Glycosides from the Roots of *Anemone vitifolia* through a Pro-Apoptotic Way. *Molecules*, *22*(4), 642.

Bais, S., & Prashar, Y. (2015). Identification and characterization of amentoflavone from six species of *Juniperus* against H2O2 induced oxidative damage in human erythrocytes and leucocytes. *J. Phytochem*, *9*, 41-55.

Bakht, J., Panni, M. K., & Shafi, M. (2017). Antimicrobial potential and phyto chemical analysis of different solvent extracted samples of *viola pilosa*. *Pak. J. Bot*, *49*(4), 1485-1489.

Bala, A., Kar, B., Haldar, P. K., Mazumder, U. K., & Bera, S. (2010). Evaluation of anticancer activity of *Cleome gynandra* on Ehrlich's Ascites Carcinoma treated mice. *Journal of Ethnopharmacology*, *129*(1), 131-134.

Balachandran, C., Duraipandiyan, V., Al-Dhabi, N. A., Balakrishna, K., Kalia, N. P., Rajput, V. S., Khan, I. A., & Ignacimuthu, S. (2012). Antimicrobial and antimycobacterial activities of methyl caffeate isolated from Solanum torvum Swartz. Fruit. *Indian journal of microbiology*, *52*(4), 676-681.

Balamurugan, G., & Selvarajan, S. 2009. Preliminary phytochemical screening and anthelmintic activity of Indigofera *tinctoria* Linn. *International Journal of Drug Development and Research*, *1*(1), 157-160.

Balsevich, J. J., Ramirez-Erosa, I., Hickie, R. A., Dunlop, D. M., Bishop, G. G., & Deibert, L. K. (2012). Antiproliferative activity of *Saponaria vaccaria* constituents and related compounds. *Fitoterapia*, *83*(1), 170-181.

Barbosa Filho, J. M., Agra, M. F., Oliveira, R. A. G., Paulo, M. Q., Trolin, G., Cunha, E. V. L., Ataide, J. R. ,& Bhattacharyya, J. (1991). Chemical and pharmacological investigation of *Solanum* species of Brazil: a search for solasodine and other potentially useful therapeutic agents. *Memórias do Instituto Oswaldo Cruz*, *86*, 189-191.

Barillari, J., Cervellati, R., Paolini, M., Tatibouët, A., Rollin, P., & Iori, R. 2005. Isolation of 4-methylthio-3-butenyl glucosinolate from *Raphanus sativus* sprouts (Kaiware Daikon) and its redox properties. *Journal of Agricultural and Food Chemistry*, *53*(26), 9890-9896.

Baruah, N. C., Sarma, J. C., Sarma, S., & Sharma, R. P. (1994). Seed germination and growth inhibitory cadinenes from *Eupatorium adenophorum* spreng. *Journal of chemical ecology*, *20*(8), 1885-1892.

Bashir, K., Ahmad, B., Rauf, A., Bawazeer, S., Rahman, K. U., Rehman, T., Saleem, M., Ahmed, R. S., Linfang, H., & Ikram, R. (2017). Urease inhibition potential and molecular docking of dihydroquercetin and dihydromyricetin isolated from Picea smithiana (wall) Boiss. *Biomedical Research*, *28*(22).

Bastos, G. N. T., Santos, A. R. S., Ferreira, V. M. M., Costa, A. M. R., Bispo, C. I., Silveira, A. J. A., & Do Nascimento, J. L. M. (2006). Antinociceptive effect of the aqueous extract obtained from roots of Physalis angulata L. on mice. *Journal of ethnopharmacology*, *103*(2), 241-245.

Bau, H. M., Villaume, C. H., & Mejean, L. (2000). Effects of soybean (*Glycine max*) germination on biologically active components, nutritional values of seeds, and biological characteristics in rats. *Food/Nahrung*, *44*(1), 2-6.

Bautista, M., Madrigal-Santillan, E., Morales-González, A., Gayosso-De-Lucio, J. A., Madrigal-Bujaidar, E., Chamorro-Cevallos, G., Aguilar-Faisal, J. L., & Morales-González, J. A. (2015). An alternative hepatoprotective and antioxidant agent: the Geranium. *African Journal of Traditional, Complementary and Alternative Medicines*, *12*(4), 96-105.

Bazylko, A., Stolarczyk, M., Derwińska, M., & Kiss, A. K. (2012). Determination of antioxidant activity of extracts and fractions obtained from *Galinsoga parviflora* and *Galinsoga quadriradiata*, and a qualitative study of the most active fractions using TLC and HPLC methods. *Natural product research*, *26*(17), 1584-1593.

Begum, S., Saxena, B., Goyal, M., Ranjan, R., Joshi, V. B., Rao, C. V., Krishnamurthy, S., & Sahai, M. (2010). Study of anti-inflammatory, analgesic and antipyretic activities of seeds of *Hyoscyamus niger* and isolation of a new coumarinolignan. *Fitoterapia*, *81*(3), 178-184.

Belhouchet, Z., Sautour, M., Miyamoto, T., & Lacaille-Dubois, M. A. (2008). Steroidal Saponins from the Roots of *Smilax aspera* subsp. mauritanica. *Chemical and Pharmaceutical Bulletin*, *56*(9), 1324-1327.

Bhadoriya, S. S., Ganeshpurkar, A., Narwaria, J., Rai, G., & Jain, A. P. (2011). *Tamarindus indica*: Extent of explored potential. *Pharmacognosy reviews*, *5*(9), 73-81.

Bhagat, J., Lobo, R., Kumar, N., Mathew, J. E., & Pai, A. (2014). Cytotoxic potential of *Anisochilus carnosus* (Lf) wall and estimation of luteolin content by HPLC. *BMC complementary and alternative medicine*, *14*(1), 421.

Bhardwaj, D., & Kaushik, N. (2012). Phytochemical and pharmacological studies in genus *Berberis*. *Phytochemistry reviews*, *11*(4), 523-542.

Bhatt, U. P., Sati, S. C., Bahuguna, R. P., Semwal, R. B., & Semwal, D. K. (2018). Two antidiabetic constituents from *Roylea cinerea* (D. Don) Baill. *Natural product research*, *32*(11), 1281-1286.

Bhattacharjee, B., & Islam, S. S. (2015). Assessment of antibacterial and antifungal activities of the extracts of *Rhynchostylis retusa* Blume-A medicinal orchid. *World Journal of Pharmacy and Pharmaceutical Sciences*, *4*(2), 74-87.

Bhujbal, S. S., Kewatkar, S. M. K., More, L. S., & Patil, M. J. (2009). Antioxidant effects of roots of *Clerodendrum serratum* Linn. *Pharmacognosy Research*, *1*(5), 294.

Bibi, Y., Nisa, S., Zia, M., Waheed, A., Ahmed, S., & Chaudhary, M. F. (2012). In vitro cytotoxic activity of *Aesculus indica* against breast adenocarcinoma cell line (MCF-7) and phytochemical analysis. *Pak J Pharm Sci*, *25*(1), 183-187.

Bilia, A. R., de Malgalhaes, P. M., Bergonzi, M. C., & Vincieri, F. F. (2006). Simultaneous analysis of artemisinin and flavonoids of several extracts of *Artemisia annua* L. obtained from a commercial sample and a selected cultivar. *Phytomedicine*, *13*(7), 487-493.

Bishnoi, V. K., Kaushal, K., Sharma, A. K., & Soni, P. (2017). Potential of beta sitosterol in medicinal plants used in BPH: A review.*International Journal of Chemical Science*, *1*(2), 65-68.

Bisht, D. S., Padalia, R. C., Singh, L., Pande, V., Lal, P., & Mathela, C. S. (2010). Constituents and antimicrobial activity of the essential oils of six Himalayan *Nepeta* species. *Journal of the Serbian Chemical Society*, *75*(6), 739-747.

Bisht, R., Sharma, D. & Agrawal, P. K. (2016). Antagonistic and antibacterial activity of endophytic fungi isolated from needle of *Cupressus torulosa* D. Don. *Asian J Pharm Clin Res*, *9*(3), 282-288.

Bisht, V. K., Negi, B. S., Bhandari, A. K., & Kandari, L. S. 2016. *Fritillaria roylei* Hook. in Western Himalaya: species biology, traditional use, chemical constituents, concern and opportunity. Research Journal of Medicinal Plants, 10(6-7):375-381.

Bokhari, J., Khan, M. R., Shabbir, M., Rashid, U., Jan, S., & Zai, J. A. (2013). Evaluation of diverse antioxidant activities of *Galium aparine*. *Spectrochimica Acta Part A: Molecular and Biomolecular Spectroscopy*, *102*, 24-29.

Bora, K. S., & Sharma, A. (2011). The genus *Artemisia*: a comprehensive review. *Pharmaceutical Biology*, *49*(1), 101-109.

Borchardt, J. R., Wyse, D. L., Sheaffer, C. C., Kauppi, K. L., Ehlke, R., Biesboer, D. D., & Bey, R. F. (2008). Antimicrobial activity of native and naturalized plants of Minnesota and Wisconsin. *Journal of medicinal plants research*, *2*(5), 98-110.

Bose, B., Choudhury, H., Tandon, P., & Kumaria, S. (2017). Studies on secondary metabolite profiling, anti-inflammatory potential, in vitro photoprotective and skin-aging related enzyme inhibitory activities of *Malaxis acuminata*, a threatened orchid of nutraceutical importance. *Journal of Photochemistry and Photobiology B: Biology*, *173*, 686-695.

Bozan, B., & Temelli, F. (2008). Chemical composition and oxidative stability of flax, safflower and poppy seed and seed oils. *Bioresource Technology*, *99*(14), 6354-6359.

Brahmachari, G., Gorai, D., & Roy, R. (2013). *Argemone mexicana*: chemical and pharmacological aspects. *Revista Brasileira de Farmacognosia*, *23*(3), 559-567.

Brennan, R. M. (2008). Currants and gooseberries. In *Temperate Fruit Crop Breeding* (pp. 177-196). Springer Netherlands.

Carbonaro, M., Mattera, M., Nicoli, S., Bergamo, P., & Cappelloni, M. (2002). Modulation of antioxidant compounds in organic vs conventional fruit (peach, *Prunus persica* L., and pear, *Pyrus communis* L.). *Journal of agricultural and food chemistry*, *50*(19), 5458-5462.

Carović-Stanko, K., Orlić, S., Politeo, O., Strikić, F., Kolak, I., Milos, M., & Satovic, Z. (2010). Composition and antibacterial activities of essential oils of seven *Ocimum* taxa. *Food Chemistry*, *119*(1), 196-201.

Cassady, J. M., Zennie, T. M., Chae, Y. H., Ferin, M. A., Portuondo, N. E., & Baird, W. M. (1988). Use of a mammalian cell culture benzo (a) pyrene metabolism assay for the detection of potential anticarcinogens from natural products: inhibition of metabolism by biochanin A, an isoflavone from *Trifolium pratense* L. *Cancer research*, *48*(22), 6257-6261.

Cerda, B., Ceron, J.J., Tomas-Barberan, F.A., Espin, J.C., (2003). Repeated oral administra- tion of high doses of the pomegranate ellagitannin punicalagin to rats for 37 day is not toxic. *Journal of Agricultural and Food Chemistry, 51*, 3493–3501.

Chan, Y. S., Cheng, L. N., Wu, J. H., Chan, E., Kwan, Y. W., Lee, S. M. Y., Leung, G. P. H., Yu, H. P. H. & Chan, S. W. (2011). A review of the pharmacological effects of *Arctium lappa* (burdock). *Inflammopharmacology*, *19*(5), 245-254.

Chanayath, N., Lhieochaiphant, S., & Phutrakul, S. 2002. Pigment extraction techniques from the leaves of *Indigofera tinctoria* Linn. and *Baphicacanthus cusia* Brem. and chemical structure analysis of their major components. *Chiang Mai University Journal*, 1(2):149-160.

Chang, L. C., Gerhäuser, C., Song, L., Farnsworth, N. R., Pezzuto, J. M., & Kinghorn, A. D. (1997). Activity-guided isolation of constituents of *Tephrosia purpurea* with the potential to induce the phase II enzyme, quinone reductase. *Journal of natural products*, *60*(9), 869-873.

Chanotiya, C. S., Yadav, A. K., & Singh, A. K. (2009). Leaf Oil Composition of *Premna barbata* Wall. ex. Sch. from Kumaon Region of Uttarakhand. *Journal of Essential Oil Research*, *21*(1), 76-77.

Chaturvedi, P. A., Ghatak, A. A., & Desai, N. S. (2012). Evaluation of radical scavenging potential and total phenol content in *Woodfordia fruticosa* from different altitudes. *Journal of plant biochemistry and biotechnology*, *21*(1), 17-22.

Chaudhary, G., Goyal, S., & Poonia, P. (2010). *Lawsonia inermis* Linnaeus: a phytopharmacological review. *International Journal of Pharmaceutical Sciences and Drug Research*, *2*(2), 91-98.

Chaudhuri, A. & Ray, S. (2015). Antiproliferative activity of phytochemicals present in aerial parts aqueous extract of *Ampelocissus latifolia* (Roxb.) planch. on apical meristem cells. *Int J Pharm Bio Sci*, *6*(2), 99-108.

Chauhan, R. S., & Dutt, P. (2013). Swertia ciliata-A new source of mangiferin, amaroswerin and amarogentin. *Journal of Biologically Active Products from Nature*, *3*(2), 161-165.

Chea, A., Hout, S., Long, C., Marcourt, L., Faure, R., Azas, N., & Elias, R. (2006). Antimalarial activity of sesquiterpene lactones from *Vernonia cinerea*. *Chemical and pharmaceutical bulletin*, *54*(10), 1437-1439.

Chen, C., Hsin, W, Ko, F., Huang, Y., Ou, J. & Teng, C. (1996). Antiplatelet arylnaphalide lignans from *Justicia procumbens. Journal of natural products, 59*, 1149-1150.

Chen, C., Song, Q., Proffit, M., Bessière, J. M., Li, Z., & Hossaert‐McKey, M. (2009). Private channel: a single unusual compound assures specific pollinator attraction in *Ficus semicordata*. *Functional Ecology*, *23*(5), 941-950.

Chen, H. C., Chou, C. K., Lee, S. D., Wang, J. C., & Yeh, S. F. (1995). Active compounds from *Saussurea lappa* Clarks that suppress hepatitis B virus surface antigen gene expression in human hepatoma cells. *Antiviral Research*, *27*(1-2), 99-109.

Chen, S., Zhang, D., Chen, S., Xia, T., Gao, Q., Duan, Y., & Zhang, F. (2008). Determination of salidroside in medicinal plants belonging to the *Rhodiola* L. genus originating from the Qinghai–Tibet Plateau. *Chromatographia*, *68*(3-4), 299-302.

Chen, Y. C., Tien, Y. J., Chen, C. H., Beltran, F. N., Amor, E. C., Wang, R. J., Wu, D. J., Mettling, C., Lin, Y. L., & Yang, W. C. (2013). *Morus alba* and active compound oxyresveratrol exert anti-inflammatory activity via inhibition of leukocyte migration involving MEK/ERK signaling. *BMC complementary and alternative medicine*, *13*(1), 45.

Cheng, L., Ye, Y., Xiang, L., Osada, H., & Qi, J. (2017). Lindersin B from *Lindernia crustacea* induces neuritogenesis by activation of tyrosine kinase A/phosphatidylinositol 3 kinase/extracellular signal-regulated kinase signaling pathway. *Phytomedicine*, *24*, 31-38.

Chhatre, S., Nesari, T., Somani, G., Kanchan, D., & Sathaye, S. (2014). Phytopharmacological overview of Tribulus terrestris. *Pharmacognosy reviews*, *8*(15), 45-51.

Chhetri, H. P., Yogol, N. S., Sherchan, J., Anupa, K. C., Mansoor, S., & Thapa, P. (2008). Phytochemical and antimicrobial evaluations of some medicinal plants of Nepal. *Kathmandu university journal of science, engineering and technology*, *4*(1):49-54.

Chinchansure, A. A., Arkile, M., Shukla, A., Shanmugam, D., Sarkar, D., & Joshi, S. P. (2015). *Leucas mollissima*, a source of bioactive compounds with antimalarial and antimycobacterium activities. *Planta Medica Letters*, *2*(1), 35-38.

Chittoor, M. S., Binny, A. R., Yadlapalli, S. K., Cheruku, A., Dandu, C., & Nimmanapalli, Y. (2012). Anthelmintic and antimicrobial studies of *Drimia indica* (Roxb.) Jessop. bulb aqueous extracts. *Journal of Pharmacy Research,* 5 (5*)*, 3677-3686.

Choochote, W., Chaiyasit, D., Kanjanapothi, D., Rattanachanpichai, E., Jitpakdi, A., Tuetun, B., & Pitasawat, B. (2005). Chemical composition and anti-mosquito potential of rhizome extract and volatile oil derived from *Curcuma aromatica* against Aedes aegypti (Diptera: Culicidae). *Journal of vector ecology*, *30*(2), 302.

Chouhan, H. S., & Singh, S. K. (2011). A review of plants of genus *Leucas*. *Journal of Pharmacognosy and Phytotherapy*, *3*(2), 13-26.

Chowdhury, M. A., Rahman, M. M., Chowdhury, M. R. H., Uddin, M. J., Sayeed, M. A., & Hossain, M. A. (2014). Antinociceptive and cytotoxic activities of an epiphytic medicinal orchid: *Vanda tessellata* Roxb. *BMC complementary and alternative medicine*, *14*(1), 464.

Colegate, S. M., Gardner, D. R., Joy, R. J., Betz, J. M., & Panter, K. E. (2012). Dehydropyrrolizidine alkaloids, including monoesters with an unusual esterifying acid, from cultivated *Crotalaria juncea* (Sunn Hemp cv.‘Tropic Sun’). *Journal of agricultural and food chemistry*, *60*(14), 3541-3550.

Couto, V. M., Vilela, F. C., Dias, D. F., dos Santos, M. H., Soncini, R., Nascimento, C. G. O., & Giusti-Paiva, A. (2011). Antinociceptive effect of extract of *Emilia sonchifolia* in mice. *Journal of Ethnopharmacology*, *134*(2), 348-353.

Cuellar, M. J., Giner, R. M., Recio, M. C., Just, M. J., Manez, S., Cerda, S., & Ríos, J. L. (1998). Screening of antiinflammatory medicinal plants used in traditional medicine against skin diseases. *Phytotherapy Research*, *12*(1), 18-23.

Cui, X., Wang, Y., Kokudo, N., Fang, D., & Tang, W. (2010). Traditional Chinese medicine and related active compounds against hepatitis B virus infection. *Bioscience trends*, *4*(2), 39-47.

Cutillo, F., D'Abrosca, B., DellaGreca, M., Di Marino, C., Golino, A., Previtera, L., & Zarrelli, A. (2003). Cinnamic acid amides from *Chenopodium album*: effects on seeds germination and plant growth. *Phytochemistry*, *64*(8), 1381-1387.

da Silva, A. G., Silva, M. W., Bezerra, G. B., & Ramos, C. S. (2018). The first report of chemical and biological study of essential oil from *Begonia reniformis* leaf (Begoniaceae). *Eclética Química Journal*, *42*(1), 60-64.

Daayf, F., Ongena, M., Boulanger, R., El Hadrami, I., & Bélanger, R. R. (2000). Induction of phenolic compounds in two cultivars of cucumber by treatment of healthy and powdery mildew-infected plants with extracts of *Reynoutria sachalinensis*. *Journal of Chemical Ecology*, *26*(7), 1579-1593.

Dahham, S. S., Ali, M. N., Tabassum, H., & Khan, M. (2010). Studies on antibacterial and antifungal activity of pomegranate (*Punica granatum* L.). *Am.–Eurasian J. Agric. Environ. Sci.*, *9*, 273-281.

Dall’Acqua, S., Minesso, P., Shresta, B. B., Comai, S., Jha, P. K., Gewali, M. B., Greco, E., Cervellati, R., & Innocenti, G. (2012). Phytochemical and antioxidant-related investigations on bark of *Abies spectabilis* (D. don) spach. from Nepal. *Molecules*, *17*(2), 1686-1697.

Dalli, A. K., Saha, G., & Chakraborty, U. (2007). Characterization of antimicrobial compounds from a common fern, *Pteris biaurita*. Indian Journal of experimental Biology, *45*, 285-290.

Dar, K. B., Bhat, A. H., AmIN, S., ANeeS, S. U. H. A. I. L., Masood, A., Zargar, M. I., & Ganie, S. A. (2016). Efficacy of aqueous and methanolic extracts of Rheum spiciformis against pathogenic bacterial and fungal strains. *Journal of Clinical and Diagnostic Research,*  *10*(9).

Dar, M. Y., Shah, W. A., Mubashir, S., & Rather, M. A. (2012). Chromatographic analysis, anti-proliferative and radical scavenging activity of *Pinus wallichina* essential oil growing in high altitude areas of Kashmir, India. *Phytomedicine*, *19*(13), 1228-1233.

Dashputre, N. L., & Naikwade, N. S. (2011). Evaluation of anti-ulcer activity of methanolic extract of *Abutilon indicum* Linn leaves in experimental rats. *Int J Pharm Sci Drug Res*, *3*(2), 97-100.

Datta, K. S., & Nanda, K. K. (1985). Effect of some phenolic compounds and gibberellic acid on growth and development of cheena millet (*Panicum miliaceum* L.). *Indian J. Plant Physiol*, *28*(3), 298-302.

Datta, S., & Saxena, D. B. (2001). Pesticidal properties of parthenin (from *Parthenium hysterophorus*) and related compounds. *Pest Management Science*, *57*(1), 95-101.

De Martino, L., De Feo, V., Formisano, C., Mignola, E., & Senatore, F. (2009). Chemical composition and antimicrobial activity of the essential oils from three chemotypes of *Origanum vulgare* L. ssp. hirtum (Link) Ietswaart growing wild in Campania (Southern Italy). *Molecules*, *14*(8), 2735-2746.

De Mira, N. V. M., Massaretto, I. L., Pascual, C. D. S. C. I., & Marquez, U. M. L. (2009). Comparative study of phenolic compounds in different Brazilian rice (Oryza sativa L.) genotypes. *Journal of Food Composition and Analysis*, *22*(5), 405-409.

De, J., Lu, Y., Ling, L., Peng, N., & Zhong, Y. (2017). Essential Oil Composition and Bioactivities of *Waldheimia glabra* (Asteraceae) from Qinghai-Tibet Plateau. *Molecules*, *22*(3), 460.

Deepak, M., Sangli, G. K., Arun, P. C., & Amit, A. (2005). Quantitative determination of the major saponin mixture bacoside A in *Bacopa monnieri* by HPLC. *Phytochemical Analysis*, *16*(1), 24-29.

del Baño, M. J., Lorente, J., Castillo, J., Benavente-García, O., del Río, J. A., Ortuño, A., Quirin, K. W.,& Gerard, D. (2003). Phenolic diterpenes, flavones, and rosmarinic acid distribution during the development of leaves, flowers, stems, and roots of Rosmarinus officinalis. Antioxidant activity. *Journal of Agricultural and Food Chemistry*, *51*(15), 4247-4253.

Dembitsky, V. M., Tolstikov, G. A., & Tolstikov, A. G. (2003). Natural halogenated polyacetylenides. *Chemistry of Sustainable Development*, *11*, 341-348.

Devi, N. N. & Singh, M. S. (2013). GC-MS analysis of metabolites from endophytic fungus *colletotrichum gloeosporioides* isolated from *phlogacanthus thyrsiflorus* Nees. *Int J Pharm Sci*, *23*(2), 392-395.

Devi, S. L., Kannappan, S., & Anuradha, C. V. (2007). Evaluation of in vitro antioxidant activity of Indian bay leaf, *Cinnamomum tamala* (Buch.-Ham.) T. Nees & Eberm using rat brain synaptosomes as model system. *Indian Journal of Experimental Biology*, *45*, 778-784.

Devmurari, V. P., Ghodasara, T. J., & Jivani, N. P. (2010). Antibacterial activity and phytochemical study of ethanolic extract of *Triumfetta rhomboidea* Jacq. *Int. Jr. of Pharma and BioSci*, *2*(2), 1182-1186.

Dhale, D. A. (2011). Phytochemical screening and antimicrobial activity of *Bauhinia variegata* Linn. *Journal of Ecobiotechnology*, *3*(9), 4-7.

Dhalwal, K., Shinde, V. M., Biradar, Y. S., & Mahadik, K. R. (2008). Simultaneous quantification of bergenin, catechin, and gallic acid from *Bergenia ciliata* and *Bergenia ligulata* by using thin-layer chromatography. *Journal of food composition and analysis*, *21*(6), 496-500.

Dhivya, R., & Manimegalai, K. (2013). Preliminary phytochemical screening and gc-ms profiling of ethanolic flower extract of *Calotropis gigantea* Linn.(Apocyanaceae). *Journal of Pharmacognosy and Phytochemistry*, *2*(3), 28-32.

Dhole, J. A., Dhole, N. A., Lone, K. D., Bodke, S. S., & Dhole, J. A. (2011). Preliminary phytochemical analysis and antimicrobial activity of some weeds collected from Marathwada region. *Journal of research in Biology*, *1*, 19-23.

Diab, Y., Atalla, K., & Elbanna, K. (2012). Antimicrobial screening of some Egyptian plants and active flavones from *Lagerstroemia indica* leaves. *Drug discoveries & therapeutics*, *6*(4), 212-217.

Ding, Z. S., Jiang, F. S., Chen, N. P., Lv, G. Y., & Zhu, C. G. (2008). Isolation and identification of an anti-tumor component from leaves of *Impatiens balsamina*. *Molecules*, *13*(2), 220-229.

Divya, T. M., Soorya, V. C., Amithamol, K. K., Juliet, S., Ravindran, R., Nair, S. N., & Ajithkumar, K. G. (2014). Acaricidal activity of alkaloid fractions of *Leucas indica* Spreng against Rhipicephalus (Boophilus) annulatus tick. *Tropical biomedicine*, *31*(1), 46-53.

Dixit, P., Chillara, R., Khedgikar, V., Gautam, J., Kushwaha, P., Kumar, A., Singh, D., Trivedi, R., & Maurya, R. (2012). Constituents of *Dalbergia sissoo* Roxb. leaves with osteogenic activity. *Bioorganic & medicinal chemistry letters*, *22*(2), 890-897.

Dixit, V., Verma, P., Agnihotri, P., Paliwal, A. K., Rao, C. V., & Husain, T. (2015). Antimicrobial, antioxidant and wound healing properties of *Leucas lanata* Wall. ex Benth. *J. Phytopharm*, *4*(1), 9-16.

Dorman, H. J. D., & Deans, S. G. (2000). Antimicrobial agents from plants: antibacterial activity of plant volatile oils. *Journal of applied microbiology*, *88*(2), 308-316.

Du, Q., Xu, Y., Li, L., Zhao, Y., Jerz, G., & Winterhalter, P. 2006. Antioxidant constituents in the fruits of *Luffa cylindrica* (L.) Roem. *Journal of agricultural and food chemistry*, 54(12):4186-4190.

Du, Z. Z., He, H. P., Wu, B., Shen, Y. M., & Hao, X. J. (2004). Chemical constituents from the pericarp of *Trewia nudiflora*. *Helvetica chimica acta*, *87*(3), 758-763.

Dubal, K. N., Ghorpade, P. N., & Kale, M. V. (2013). Studies on bioactive compounds of *Tectaria coadunata* (Wall. Ex Hook. & Grev.) C. Chr. *Asian J Pharm Clin Res*, *6*(3), 186-187.

Duhan, A., Chauhan, B. M., Punia, D., & Kapoor, A. C. (1989). Phytic acid content of chickpea (*Cicer arietinum*) and black gram (*Vigna mungo*): varietal differences and effect of domestic processing and cooking methods. *Journal of the Science of Food and Agriculture*, *49*(4), 449-455.

Dutt, B., Srivastava, L. J., & Singh, J. M. (1996). *Swertia* spp: a source of bitter compounes for medicinal use. *Ancient Science of life*, *15*(3), 226.

Dzoyem, J. P., Pieme, C. A., & Penlap, V. B. (2010). In vitro antibacterial activity and acute toxicity studies of aqueous-methanol extract of *Sida rhombifolia* Linn.(Malvaceae). *BMC complementary and alternative medicine*, *10*(1), 40.

Edeoga, H. O., Omosun, G., & Uche, L. C. (2006). Chemical composition of *Hyptis suaveolens* and *Ocimum gratissimum* hybrids from Nigeria. *African Journal of Biotechnology*, *5*(10), 892-895.

Elaiyaraja, A., & Chandramohan, G. (2016). Comparative phytochemical profile of *Indoneesiella echioides* (L.) Nees leaves using GC-MS. *Journal of Pharmacognosy and Phytochemistry*, *5*(6), 158-171.

El-Baroty, G. S., El-Baky, H. A., Farag, R. S., & Saleh, M. A. (2010). Characterization of antioxidant and antimicrobial compounds of cinnamon and ginger essential oils. *African Journal of Biochemistry Research*, *4*(6), 167-174.

El-Fishawy, A., Zayed, R., & Afifi, S. (2011). Phytochemical and pharmacological studies of *Ficus auriculata* Lour. *J. Nat. Prod*, *4*, 184-195.

El-Ghorab, A., El-Massry, K. F., & Shibamoto, T. (2007). Chemical composition of the volatile extract and antioxidant activities of the volatile and nonvolatile extracts of Egyptian corn silk (*Zea mays* L.). *Journal of agricultural and food chemistry*, *55*(22), 9124-9127.

El-Sayed, A. M., Al-Yahya, M. A., & Hassan, M. M. (1989). Chemical composition and antimicrobial activity of the essential oil of *Chenopodium botrys* growing in Saudi Arabia. *International Journal of Crude Drug Research*, *27*(4), 185-188.

Essien, E. E., Ogunwande, I. A., Setzer, W. N., & Ekundayo, O. (2012). Chemical composition, antimicrobial, and cytotoxicity studies on *S. erianthum* and *S. macranthum* essential oils. *Pharmaceutical biology*, *50*(4), 474-480.

Fa, O. (2013). Effect of plant maturity on the antioxidant profile of *Amaranthus cruentus* L. and *Celosia argentea* L. *Bull. Env. Pharmacol. Life Sci*., *2*, 18-21.

Fabjan, N., Rode, J., Košir, I. J., Wang, Z., Zhang, Z., & Kreft, I. (2003). Tartary buckwheat (*Fagopyrum tataricum* Gaertn.) as a source of dietary rutin and quercitrin. *Journal of agricultural and food chemistry*, *51*(22), 6452-6455.

Fahey, J. W., Zalcmann, A. T., & Talalay, P. (2001). The chemical diversity and distribution of glucosinolates and isothiocyanates among plants. *Phytochemistry*, *56*(1), 5-51.

Fan, L., Zhao, H. Y., Xu, M., Zhou, L., Guo, H., Han, J. Wang, B.R. & Guo, D. A. (2009). Qualitative evaluation and quantitative determination of 10 major active components in *Carthamus tinctorius* L. by high-performance liquid chromatography coupled with diode array detector. *Journal of Chromatography A*, *1216*(11), 2063-2070.

Fatima, N., Akhtar, F., Choudhary, M. I., & Khalid, A. (2000). New norditerpenoid alkaloids from *Aconitum falconeri.* *Journal of natural products*, *63*(10), 1393-1395.

Fauzan, A., Praseptiangga, D., Hartanto, R., & Pujiasmanto, B. (2018). Characterization of the chemical composition of *Adenostemma lavenia* (L.) Kuntze and Adenostemma *platyphyllum* Cass. In *IOP Conference Series: Earth and Environmental Science*, *102*(1), 12029.

Feng, L., Jia, X., Zhu, M. M., Chen, Y., & Shi, F. (2010). Antioxidant activities of total phenols of *Prunella vulgaris* L. in vitro and in tumor-bearing mice. *Molecules*, *15*(12), 9145-9156.

Ferheen, S., Rehman, A. U., Afza, N., Malik, A., Iqbal, L., Azam Rasool, M. Irfan Ali, M. & Bakhsh Tareen, R. (2009). Galinsosides A and B, bioactive flavanone glucosides from *Galinsoga parviflora*. *Journal of enzyme inhibition and medicinal chemistry*, *24*(5), 1128-1132.

Fernandes, F., Valentão, P., Sousa, C., Pereira, J. A., Seabra, R. M., & Andrade, P. B. (2007). Chemical and antioxidative assessment of dietary turnip (*Brassica rapa* var. *rapa* L.). *Food Chemistry*, *105*(3), 1003-1010.

Ferreira, P. M. P., Farias, D. F., Oliveira, J. T. D. A., & Carvalho, A. D. F. U. (2008). *Moringa oleifera*: bioactive compounds and nutritional potential. *Revista de Nutrição*, *21*(4), 431-437.

Fischer, H., Jensen, W., Jensen, S. R., & Nielsen, B. J. (1987).Eranthemoside, a new iridoid glucoside from Eranthemum pulchellum (Acanthaceae). *Phytochemistry*, *26*(12), 3353-3354.

Forlani, G., Lejczak, B., & Kafarski, P. (2000). The herbicidally active compound N-2-(5-chloro-pyridyl) aminomethylene bisphosphonic acid acts by inhibiting both glutamine and aromatic amino acid biosynthesis. *Functional Plant Biology*, *27*(7), 677-683.

Formukong, E. A., Evans, A. T., & Evans, F. J. (1988). Analgesic and antiinflammatory activity of constituents of *Cannabis sativa* L. *Inflammation*, *12*(4), 361-371.

Friedman, M. (2004). Analysis of biologically active compounds in potatoes (*Solanum tuberosum*), tomatoes (*Lycopersicon esculentum*), and jimson weed (*Datura stramonium*) seeds. *Journal of Chromatography A*, *1054*(1-2), 143-155.

Fu, G., Liu, Y., Yu, S., Huang, X., Hu, Y., Chen, X., & Zhang, F. (2006). Cytotoxic oxygenated triterpenoid saponins from *Symplocos chinensis.* *Journal of natural products*, *69*(12), 1680-1686.

FU, P. P., Yang, Y. C., XIA, Q., CHOU, M. W., CUI, Y. Y., & Lin, G. (2002). Pyrrolizidine Alkaloids-Tumorigenic Components in Chinese Herbal Medicines and Dietary Supplemen. *Journal of Food and Drug Analysis*, *10*(4), 198-211.

Gadano, A., Gurni, A., López, P., Ferraro, G., & Carballo, M. (2002). In vitro genotoxic evaluation of the medicinal plant *Chenopodium ambrosioides* L. *Journal of Ethnopharmacology*, *81*(1), 11-16.

Gambhire, M., Wankhede, S., & Juvekar, A. (2009). Antiinflammatory activity of aqueous extract of *Barleria cristata* leaves. *Journal of Young Pharmacists*, *1*(3), 220.

Gandhiraja, N., Sriram, S., Meenaa, V., Srilakshmi, J. K., Sasikumar, C., & Rajeswari, R. (2009). Phytochemical screening and antimicrobial activity of the plant extracts of *Mimosa pudica* L. against selected microbes. *Ethnobotanical leaflets*, *13*, 618-24.

Ganie, S. A., Dar, T. A., Hamid, R., Zargar, O., Abeer, S. U., Masood, A., Amin, S. & Zargar, M. A. (2014). In vitro antioxidant and cytotoxic activities of *Arnebia benthamii* (Wall ex. G. Don): a critically endangered medicinal plant of Kashmir valley. *Oxidative medicine and cellular longevity,* 2014.

Gao, G., Lu, Z., Tao, S., Zhang, S., & Wang, F. (2011). Triterpenoid saponins with antifeedant activities from stem bark of *Catunaregam spinosa* (Rubiaceae) against Plutella xylostella (Plutellidae). *Carbohydrate research*, *346*(14), 2200-2205.

Gatto, M. A., Ippolito, A., Linsalata, V., Cascarano, N. A., Nigro, F., Vanadia, S., & Di Venere, D. (2011). Activity of extracts from wild edible herbs against postharvest fungal diseases of fruit and vegetables. *Postharvest Biology and Technology*, *61*(1), 72-82.

Gautam, A., Kumar, A., & Dutt, D. (2016). Effects of Ethanol Addition and Biological Pretreatment on Soda Pulping of *Eulaliopsis binata*. *Journal of Biomaterials and Nanobiotechnology*, *7*(2), 78-90.

Gautam, M. K., Gangwar, M., Nath, G., Rao, C. V., & Goel, R. K. (2012). In–vitro antibacterial activity on human pathogens and total phenolic, flavonoid contents of *Murraya paniculata* Linn. leaves. *Asian Pacific Journal of Tropical Biomedicine*, *2*(3), 1660-1663.

Gautam, S. S., Kumar, S., Painuly, D., & Mohan, M. (2016). Volatile Constituents of *Nepeta ciliaris* Benth. roots from Kumaun Himalayas. *National Academy Science Letters*, *39*(6), 465-467.

Gayathri, M., & Kannabiran, K. (2009). Antimicrobial activity of *Hemidesmus indicus*, *Ficus bengalensis* and *Pterocarpus marsupium* roxb. *Indian journal of pharmaceutical sciences*, *71*(5), 578581.

Genovese, A., Ugliano, M., Pessina, R., Gambuti, A., Piombino, P., & Moio, L. (2004). Comparison of the aroma compounds in apricot (Prunus armeniaca, l. cv. pellecchiella) and apple (*malus pumila*, l. cv. annurca) raw distillates. *Italian Journal of Food Science*, *16*(2), 185-196.

Ghanati, F., Khatami, F., & Bemani, E. (2013). Effects of UV B and UV C radiation on viability, growth, and major natural compounds of *Malve neglecta* L. cells. Iranian Journal of Plant physiology, *4*(1), 881-887.

Ghimire, B. K., Tamang, J. P., Yu, C. Y., Jung, S. J., & Chung, I. M. (2012). Antioxidant, antimicrobial activity and inhibition of α-glucosidase activity by *Betula alnoides* Buch. bark extract and their relationship with polyphenolic compounds concentration. *Immunopharmacology and immunotoxicology*, *34*(5), 824-831.

Ghisalberti, E. L. (2000). *Lantana camara* L.(verbenaceae). *Fitoterapia*, *71*(5), 467-486.

Ghosal, S., Jaiswal, D. K., Singh, S. K., & Srivastava, R. S. (1985). Dichotosin and dichotosinin, two adaptogenic glucosyloxy flavans from *Hoppea dichotoma*. *Phytochemistry*, *24*(4), 831-833.

Gilani, A. U. H., Ghayur, M. N., Khalid, A., & Choudhary, M. I. (2005). Presence of antispasmodic, antidiarrheal, antisecretory, calcium antagonist and acetylcholinesterase inhibitory steroidal alkaloids in *Sarcococca saligna*. *Planta Medica*, *71*(02), 120-125.

Giri, A., & Narasu, M. L. (2000). Production of podophyllotoxin from *Podophyllum hexandrum*: a potential natural product for clinically useful anticancer drugs. *Cytotechnology*, *34*(1-2), 17-26.

Giri, L., Dhyani, P., Rawat, S., Bhatt, I. D., Nandi, S. K., Rawal, R. S., & Pande, V. (2012). In vitro production of phenolic compounds and antioxidant activity in callus suspension cultures of *Habenaria edgeworthii*: a rare Himalayan medicinal orchid. *Industrial Crops and Products*, *39*, 1-6.

Golla, U., Gajam, P. K., & Bhimathati, S. S. (2014). Evaluation of diuretic and laxative activity of hydro-alcoholic extract of *Desmostachya bipinnata* (L.) Stapf in rats. *Journal of integrative medicine*, *12*(4), 372-378.

Golmohammadi, F. (2013). A viewpoint toward medical plant of *astragalus* and its main characteristics, products and economical importance in Iran (Case study: Boldaji and lake Choghakhor in Chaharmahal and Bakhtiari Province). *Technical Journal of Engineering and Applied Sciences*, *3*, 3702-3721.

Gomashe, A. V., Gulhane, P. A., Junghare, M. P., & Dhakate, N. A. (2014). Antimicrobial activity of Indian medicinal plants: *Moringa oleifera* and *Saraca indica*. *International Journal of Current Microbiology and Applied Science*, *3*(6), 161-169.

Gomathi, D., Kalaiselvi, M., Ravikumar, G., Devaki, K., & Uma, C. (2015). GC-MS analysis of bioactive compounds from the whole plant ethanolic extract of *Evolvulus alsinoides* (L.) L. *Journal of food science and technology*, *52*(2), 1212-1217.

Gong, F., Gu, H. P., Xu, Q. T., & Kang, W. Y. (2012). Genus *Mitragyna*: Ethnomedicinal uses and pharmacological studies. *Phytopharmacology*, *3*(2), 263-272.

Gong, Y., Liu, X., He, W. H., Xu, H. G., Yuan, F., & Gao, Y. X. (2012). Investigation into the antioxidant activity and chemical composition of alcoholic extracts from defatted marigold (*Tagetes erecta* L.) residue. *Fitoterapia*, *83*(3), 481-489.

Gordien, A. Y., Gray, A. I., Franzblau, S. G., & Seidel, V. (2009). Antimycobacterial terpenoids from Juniperus communis L.(Cuppressaceae). *Journal of ethnopharmacology*, *126*(3), 500-505.

Goudarzi, G. R., Saharkhiz, M. J., Sattari, M., & Zomorodian, K. (2010). Antibacterial activity and chemical composition of Ajowan (*Carum copticum* Benth. & Hook) essential oil. *Journal of Agricultural Science and Technology*, *13*, 203-208.

Goupy, P., Hugues, M., Boivin, P., & Amiot, M. J. (1999). Antioxidant composition and activity of barley (*Hordeum vulgare*) and malt extracts and of isolated phenolic compounds. *Journal of the Science of Food and Agriculture*, *79*(12), 1625-1634.

Govindarajan, M. (2010). Larvicidal efficacy of *Ficus benghalensis* L. plant leaf extracts against *Culex quinquefasciatus* Say. *Aedes aegypti*, *14*, 107-111.

Govindarajan, M., Sivakumar, R., Rajeswari, M., & Yogalakshmi, K. (2012). Chemical composition and larvicidal activity of essential oil from *Mentha spicata* (Linn.) against three mosquito species. *Parasitology research*, *110*(5), 2023-2032.

Govindarajan, R., Rastogi, S., Vijayakumar, M., Shirwaikar, A., Rawat, A. K. S., Mehrotra, S., & Pushpangadan, P. (2003). Studies on the antioxidant activities of *Desmodium gangeticum*. *Biological and pharmaceutical Bulletin*, *26*(10), 1424-1427.

Goyal, A. K., Basistha, B. C., Sen, A., & Middha, S. K. (2011). Antioxidant profiling of *Hippophae salicifolia* growing in sacred forests of Sikkim, India. *Functional Plant Biology*, *38*(9), 697-701.

Gracelin, D. H. S., Britto, A., & Kumar, B. J. R. P. (2013). Qualitative and quantitative analysis of phytochemicals in five Pteris species. *Int J Pharm Pharma Sci*, *5*, 105-7.

Greger, H. (1984). Alkamides: Structural Relationships, Distribution and Biological Activity1. *Planta Medica*, *50*(05), 366-375.

Gross, S. C., Goodarzi, G., Watabe, M., Bandyopadhyay, S., Pai, S. K., & Watabe, K. (2002). Antineoplastic activity of *Solidago virgaurea* on prostatic tumor cells in an SCID mouse model. *Nutrition and cancer*, *43*(1), 76-81.

Gulfraz, M. (2005). Investigation for bioactive compounds of *Berberis lyceum* Royle and *Justicia adhatoda* L. Ethnobotanical Leaflets,

Guo, Q., Bai, R., Zhao, B., Feng, X., Zhao, Y., Tu, P., & Chai, X. (2016). An Ethnopharmacological, Phytochemical and Pharmacological Review of the Genus *Meconopsis*. *The American journal of Chinese medicine*, *44*(03), 439-462.

Guo, Z., Liu, Z., Wang, X., Liu, W., Jiang, R., Cheng, R., & She, G. (2012). *Elsholtzia*: phytochemistry and biological activities. *Chemistry Central Journal*, *6*(1), 147.

Gupta, P. C., Sharma, N., & Rao, C. V. (2012). A review on ethnobotany, phytochemistry and pharmacology of *Fumaria indica* (Fumitory). *Asian Pacific journal of tropical biomedicine*, *2*(8), 665-669.

Gupta, P. P., Srimal, R. C., Verma, N., & Tandon, J. S. (1999). Biological activity of *Rubia cordifolia* and isolation of an active principle. *Pharmaceutical biology*, *37*(1), 46-49.

Gupta, S., Dwivedi, G. R., Darokar, M. P., & Srivastava, S. K. (2012). Antimycobacterial activity of fractions and isolated compounds from *Vetiveria zizanioides*. *Medicinal Chemistry Research*, *21*(7), 1283-1289.

Gupta, V. K., Fatima, A., Faridi, U., Negi, A. S., Shanker, K., Kumar, J. K., Rahuja, N., Luqman, S., Sisodia, B. S., Saikia, D., & Darokar, M. P. (2008). Antimicrobial potential of *Glycyrrhiza glabra* roots. *Journal of Ethnopharmacology*, *116*(2), 377-380.

Gupta, V. S., Kumar, A., Deepak, D., Khare, A., & Khare, N. K. (2003). Pregnanes and pregnane glycosides from *Marsdenia roylei*. *Phytochemistry*, *64*(8), 1327-1333.

Gupta, V., & Mittal, P. (2010). Phytochemical and pharmacological potential of *Nerium oleander*: a review. *Int J Pharm Sci Res*, *1*(3), 21-27.

Gupta, V., Mittal, P., Bansal, P., Khokra, S. L., & Kaushik, D. (2010). Pharmacological potential of *Matricaria recutita*-A review. *Int J Pharm Sci Drug Res*, *2*(1), 12-16.

Gurunathan, A., Subramaniam, P., & Maran, S. (2014). Quantification of certain secondary metabolites and evaluation of antioxidant properties in the traditional medicinal plant, *Thalictrum javanicum* blume. *World Journal of Pharmacy and Pharmaceutical Sciences*, *3*(6), 1856-1873.

Gutiérrez, R. M. P., Mitchell, S., & Solis, R. V. (2008). *Psidium guajava*: a review of its traditional uses, phytochemistry and pharmacology. *Journal of ethnopharmacology*, *117*(1), 1-27.

Gyawali, R. (2011). Comparative study of antibacterial and cytotoxic activity of two Nepalese medicinal plants- *Allium wallichii* Kunth and *Allium sativum* L. *International Journal of Pharmaceutical and Biological Archive*, *2*(5).

Gyawali, R. (2011). Phytochemical screening and anti-microbial properties of medicinal plants of Dhunkharka community, Kavrepalanchowk, Nepal. *International Journal of Pharmaceutical & Biological Archive*, *2*(6), 1663-1667.

Habbu, P. V., Smita, D. M., Mahadevan, K. M., Shastry, R. A., & Biradar, S. M. (2012). Protective effect of *Habenaria intermedia* tubers against acute and chronic physical and psychological stress paradigms in rats. *Revista Brasileira de Farmacognosia*, *22*(3), 568-579.

Haider, S. Z., Mohan, M., Pandey, A. K., & Singh, P. (2015). Repellent and fumigant activities of *Tanacetum nubigenum* Wallich. Ex DC essential oils against *Tribolium castaneum* (Herbst)(Coleoptera: Tenebrionidae). *Journal of oleo science*, *64*(8), 895-903.

Hajhashemi, V., Ghannadi, A., & Mousavi, S. (2011). Antinociceptive study of extracts of *Platanus orientalis* leaves in mice. *Research in pharmaceutical sciences*, *6*(2), 123-128.

Hamza, A. A., & Amin, A. (2007). *Apium graveolens* modulates sodium valproate‐induced reproductive toxicity in rats. *Journal of Experimental Zoology Part A: Ecological Genetics and Physiology*, *307*(4), 199-206.

Hao, D., Gu, X., Xiao, P., & Peng, Y. (2013). Chemical and biological research of Clematis medicinal resources. *Chinese Science Bulletin*, *58*(10), 1120-1129.

Haque, T., Uddin, M. Z., Saha, M. L., Mazid, M. A., & Hassan, M. A. (2014). Propagation, antibacterial activity and phytochemical profiles of *Litsea glutinosa* (Lour.) CB Robinson. *Dhaka University Journal of Biological Sciences*, *23*(2), 165-171.

Haraguchi, M., Gorniak, S. L., Ikeda, K., Minami, Y., Kato, A., Watson, A. A., Nash, R.J., Molyneux, R.J. & Asano, N. (2003). Alkaloidal components in the poisonous plant, *Ipomoea carnea* (Convolvulaceae). *Journal of Agricultural and food chemistry*, *51*(17), 4995-5000.

Harinantenaina, L., Tanaka, M., Takaoka, S., Oda, M., Mogami, O., Uchida, M., & Asakawa, Y. (2006). *Momordica charantia* constituents and antidiabetic screening of the isolated major compounds. *Chemical and Pharmaceutical Bulletin*, *54*(7), 1017-1021.

Hasan, M. F., Iqbal, M. A., & Uddin, M. S. (2016). Antibacterial and antifungal activity of *litsea monopetala* leaves on selected pathogenic strains. *European Journal of Medicinal Plants*, *12*(4), 1-8.

He, J., Yin, T., Chen, Y., Cai, L., Tai, Z., Li, Z., Liu, C., Wang, Y., & Ding, Z. (2015). Phenolic compounds and antioxidant activities of edible flowers of Pyrus pashia. *Journal of Functional Foods*, *17*, 371-379.

Hegde, K., Thakker, S. P., Joshi, A. B., Shastry, C. S., & Chandrashekhar, K. S. (2009). Anticonvulsant activity of *Carissa carandas* Linn. root extract in experimental mice. *Tropical Journal of Pharmaceutical Research*, *8*(2), 117-125.

Heleno, S. A., Stojković, D., Barros, L., Glamočlija, J., Soković, M., Martins, A.,  Queiroz, M. J. R., & Ferreira, I. C. (2013). A comparative study of chemical composition, antioxidant and antimicrobial properties of *Morchella esculenta* (L.) Pers. from Portugal and Serbia. *Food research international*, *51*(1), 236-243.

Herrmann, A., Burman, R., Mylne, J. S., Karlsson, G., Gullbo, J., Craik, D. J., Clark, R. J., & Göransson, U. (2008). The alpine violet, *Viola biflora*, is a rich source of cyclotides with potent cytotoxicity. *Phytochemistry*, *69*(4), 939-952.

Hethelyi, E., Danos, B., Tetenyi, P., & Koczka, I. (1986). GC‐MS analysis of the essential oils of four tagetes species and the anti‐microbial activity of *Tagetes minuta*. *Flavour and fragrance journal*, *1*(4‐5), 169-173.

Hillis, W. E., & Swain, T. (1959). The phenolic constituents of Prunus domestica. II.—The analysis of tissues of the Victoria plum tree. *Journal of the Science of Food and Agriculture*, *10*(2), 135-144.

Ho, R., Teai, T., Bianchini, J. P., Lafont, R., & Raharivelomanana, P. (2011). Ferns: from traditional uses to pharmaceutical development, chemical identification of active principles. In *Working with Ferns* (pp. 321-346). Springer, New York, NY.

Ho, R., Teai, T., Bianchini, J.P., Lafont, R. & Raharivelomanana, P. (2011). Ferns:from traditional uses to pharmaceutical development, chemical identification of active principles. *In Working with Ferns*, pp. 321-346. Springer, New York, NY.

Hossain, M. S., Rahman, M. S., Imon, A. R., Zaman, S., Siddiky, A. B. A., Mondal, M., Sarwar, A., Huq, T.B., Adhikary, B.C., Begum, T., & Tabassum, A. (2017). Ethnopharmacological investigations of methanolic extract of *Pouzolzia Zeylanica* (L.) Benn. *Clinical Phytoscience*, *2*(1):10.

Houghton, P. J. (1984). Ethnopharmacology of some *Buddleja* species. *Journal of Ethnopharmacology*, *11*(3), 293-308.

Houghton, P. J., & Mensah, A. Y. (1999). Biologically active compounds from *Buddleja* species. In *Phytochemicals in Human Health Protection, Nutrition, and Plant Defense* (pp. 343-368). Springer, Boston, MA.

Huang, F., Cui, H., Yu, C. & Shen, Z. (2012). Chemical constituents of dicliptera chinensis. *Chinese Journal of Experimental Traditional Medical Formulae*.

Huang, H. C., Liaw, C. C., Zhang, L. J., Ho, H. U., Kuo, L. M. Y., Shen, Y. C., & Kuo, Y. H. (2008). Triterpenoidal saponins from *Hydrocotyle sibthorpioides*. *Phytochemistry*, *69*(7): 1597-1603.

Hughens, B. G. & Lawson, L. D. (1991). Antimicrobial effects of *Allium sativum* L. (garlic), *Allium ampeloprasum* L. (elephant garlic) and *Allium cepa* L. (onion), garlic compounds and commercial garlic supplement products. *Phytotherapy Research*, *5*(4), 154-158.

Hurkadale, P. J., Shelar, P. A., Palled, S. G., Mandavkar, Y. D., & Khedkar, A. S. (2012). Hepatoprotective activity of *Amorphophallus paeoniifolius* tubers against paracetamol-induced liver damage in rats. *Asian Pacific Journal of Tropical Biomedicine*, *2*(1), 238-242.

Hussain, A. I., Anwar, F., Chatha, S. A., Latif, S., Sherazi, S. T., Ahmad, A., Worthington, J., & Sarker, S. D. (2013). Chemical composition and bioactivity studies of the essential oils from two *Thymus* species from the Pakistani flora. *LWT-Food Science and Technology*, *50*(1), 185-192.

Hussain, A. I., Anwar, F., Sherazi, S. T. H., & Przybylski, R. (2008). Chemical composition, antioxidant and antimicrobial activities of basil (*Ocimum basilicum*) essential oils depends on seasonal variations. *Food chemistry*, *108*(3), 986-995.

Hussain, H., Hussain, J., Al-Harrasi, A., & Shinwari, Z. K. (2011). Chemistry of some species genus *Lantana.* *Pakistan journal of botany*, *43*(3), 51-62.

Hussain, I., Ullah, R., Khurram, M., Ullah, N., Baseer, A., Khan, F. A., Khattak, M.U.R., Zahoor, M., Khan, J., & Khan, N. (2011). Phytochemical analysis of selected medicinal plants. *African Journal of Biotechnology*, *10*(38), 7487-7492.

Hussain, T., Gupta, R. K., Sweety, K., Khan, M. S., Hussain, M. S., Arif, M. D.,  Hussain, A., Faiyazuddin, M. D. & Rao, C. V. (2012). Evaluation of antihepatotoxic potential of *Solanum xanthocarpum* fruit extract against antitubercular drugs induced hepatopathy in experimental rodents. *Asian Pacific journal of tropical biomedicine*, *2*(6), 454-460.

Hussaini, F. A., & Shoeb, A. (1985). Isoquinoline derived alkaloids from *Berberis chitria.* *Phytochemistry*, *24*(3), 633.

Ibrahim, J., Ajaegbu, V. C., & Egharevba, H. O. (2010). Pharmacognostic and Phytochemical Analysis of *Commelina benghalensis* L. *Ethnobotanical Leaflets*, *14*, 610-615.

Ibrahim, M., Khan, A. A., Tiwari, S. K., Habeeb, M. A., Khaja, M. N., & Habibullah, C. M. (2006). Antimicrobial activity of *Sapindus mukorossi* and *Rheum emodi* extracts against H pylori: In vitro and in vivo studies. *World Journal of Gastroenterology:12*(44), 7136-7142.

Ibrahim, M., Rehman, K., Razzaq, A., Hussain, I., Farooq, T., Qadir, M. I., & Akash, M. S. H. (2018). Investigations of phytochemical constituents and their pharmacological properties isolated from genus utrica: Critical review and analysis. *Critical Reviews™ in Eukaryotic Gene Expression*, *28*, 25-66.

Igbinosa, O. O., Igbinosa, E. O., & Aiyegoro, O. A. (2009). Antimicrobial activity and phytochemical screening of stem bark extracts from *Jatropha curcas* (Linn). *African journal of pharmacy and pharmacology*, *3*(2), 58-62.

Imam, M. Z., & Akter, S. (2011). *Musa paradisiaca* L. and *Musa sapientum* L.: A phytochemical and pharmacological review. Journal of Applied Pharmaceutical Science *1*(5), 14-20.

Innocenti, G., Dall’Acqua, S., Scialino, G., Banfi, E., Sosa, S., Gurung, K., Barbera, M. & Carrara, M. (2010). Chemical composition and biological properties of *Rhododendron anthopogon* essential oil. *Molecules*, *15*(4), 2326-2338.

Innocenti, M., Gallori, S., Giaccherini, C., Ieri, F., Vincieri, F. F., & Mulinacci, N. (2005). Evaluation of the phenolic content in the aerial parts of different varieties of *Cichorium intybus* L. *Journal of Agricultural and Food Chemistry*, *53*(16), 6497-6502.

Inya-Agha, S. I., Oguntimein, B. O., Sofowora, A., & Benjamin, T. V. (1987). Phytochemical and antibacterial studies on the essential oil of *Eupatorium odoratum*. *International Journal of Crude Drug Research*, *25*(1), 49-52.

Iqbal, M., & Gnanaraj, C. (2012). *Eleusine indica* L. possesses antioxidant activity and precludes carbon tetrachloride (CCl 4)-mediated oxidative hepatic damage in rats. *Environmental health and preventive medicine*, *17*(4), 307.

Irshad, M., Aziz, S., Habib-ur-Rehman, & Hussain, H. (2012). GC-MS analysis and antifungal activity of essential oils of *Angelica glauca, Plectranthus rugosus*, and *Valeriana wallichii*. *Journal of Essential Oil Bearing Plants*, *15*(1), 15-21.

İşcan, G., Ki̇ri̇mer, N., Kürkcüoǧlu, M., Başer, H. C., & DEMIrci, F. (2002). Antimicrobial screening of *Mentha piperita* essential oils. *Journal of agricultural and food chemistry*, *50*(14), 3943-3946.

Ishtiaq, S., Meo, M. B., Afridi, M. S. K., Akbar, S., & Rasool, S. (2016). Pharmacognostic studies of aerial parts of *Colebrookea oppositifolia* Sm. *Annals of Phytomedicine–An International Journal*, 5(2), 161-167.

Islam, E., Islam, R., Rahman, A. A., Alam, A. K., Khondkar, P., Rashid, M., & Parvin, S. (2013). Estimation of total phenol and in vitro antioxidant activity of *Albizia procera* leaves. *BMC research notes*, *6*(1), 121.

Isman, M. B. (2006). Botanical insecticides, deterrents, and repellents in modern agriculture and an increasingly regulated world. *Annu. Rev. Entomol.*, *51*, 45-66.

Ivanov, I., Vrancheva, R., Marchev, A., Petkova, N., Aneva, I., Denev, P., Georgiev, V. G. & Pavlov, A. (2014). Antioxidant activities and phenolic compounds in Bulgarian *Fumaria* species. *Int. J. Curr. Microbiol. App. Sci*, *3*(2), 296-306.

Jafri, L., Saleem, S., Ullah, N., & Mirza, B. (2017). In vitro assessment of antioxidant potential and determination of polyphenolic compounds of *Hedera nepalensis* K. Koch. *Arabian Journal of Chemistry*, *10*, 3699-3706.

Jagatheeswari, D., Deepa, J., Ali, H. S. J., & Ranganathan, P. (2013). *Acalypha indica* L-An important medicinal plant: A review of its traditional uses and pharmacological properties. *International Journal of research in botany*, *3*(1), 19-22.

Jagtap, S. S., Satpute, R. A., Rahatgaonkar, A. M., & Lanjewar, K. R. (2014). Phytochemical screening, antioxidant, antimicrobial and quantitative multi-elemental analysis of *Habenaria longicorniculata* J. *Graham. J Acad Ind Res*, *3*, 108-117.

Jahan, T. (2016). Phytochemical and biological evaluation of Lindenbergia indica. Dessertation (Bachelor of Pharmacy).

Jain, M., Jain, V. K., & Sharma, S. S. (2018). Rosaceae of Solan District of Himachal Pradesh. *International*Journal*of Scientific Research in Science and Technology*,*4*, 2: 1580-1588.

Jain, R., Sharma, A., Gupta, S., Sarethy, I. P., & Gabrani, R. (2011). *Solanum nigrum*: current perspectives on therapeutic properties. *Altern Med Rev*, *16*(1), 78-85.

Jamkhande, P. G., Barde, S. R., Patwekar, S. L., & Tidke, P. S. (2013). Plant profile, phytochemistry and pharmacology of *Cordia dichotoma* (Indian cherry): A review. *Asian Pacific journal of tropical biomedicine*, *3*(12), 1009-1012.

Jana, A., & Biswas, S. M. (2011). Lactam nonanic acid, a new substance from *Cleome viscosa* with allelopathic and antimicrobial properties. *Journal of biosciences*, *36*(1), 27-35.

Jana, S., & Shekhawat, G. S. (2011). Critical review on medicinally potent plant species: *Gloriosa superba*. *Fitoterapia*, *82*(3), 293-301.

Jardim, C. M., Jham, G. N., Dhingra, O. D., & Freire, M. M. (2008). Composition and antifungal activity of the essential oil of the Brazilian *Chenopodium ambrosioides* L. *Journal of chemical ecology*, *34*(9), 1213-1218.

Jarial, R., Thakur, S., Sakinah, M., Zularisam, A. W., Sharad, A., Kanwar, S. S., & Singh, L. (2016). Potent anticancer, antioxidant and antibacterial activities of isolated flavonoids from *Asplenium nidus*. *Journal of King Saud University-Science*, *30*, 185-192.

Jash, S. K., Gorai, D., & Roy, R. (2016). *Salvia* genus and triterpenoids. *International Journal of Pharmaceutical Sciences and Research*, *7*(12), 4710.

Jayaprakasam, B., Zhang, Y. & Nair, M. G. (2004). Tumor cell proliferation and cyclooxygenase enzyme inhibitory compounds in *Amaranthus tricolor*. *Journal of Agriculture and Food Chemistry*, *52*(23), 6939-6943.

Jayaprakasha, G. K., Negi, P. S., Jena, B. S., & Rao, L. J. M. (2007). Antioxidant and antimutagenic activities of *Cinnamomum zeylanicum* fruit extracts. *Journal of Food Composition and Analysis*, *20(*3-4), 330-336.

Jena, J., & Gupta, A. K. (2012). *Ricinus communis* Linn: a phytopharmacological review. *Int J Pharm Pharm Sci*, *4*(4), 25-29.

Jeong, E. K., Lee, S. Y., Yu, S. M., Park, N. H., Lee, H. S., Yim, Y. H., Hwang, G. S., Cheong, C., Jung, J. H.,  & Hong, J. (2012). Identification of structurally diverse alkaloids in *Corydalis* species by liquid chromatography/electrospray ionization tandem mass spectrometry. *Rapid Communications in Mass Spectrometry*, *26*(15), 1661-1674.

Jiang, X., Sun, D., Zhang, G., He, N., Liu, H., Huang, J., Odoom-Wubah, T. & Li, Q. (2013). Investigation of active biomolecules involved in the nucleation and growth of gold nanoparticles by *Artocarpus heterophyllus* Lam leaf extract. *Journal of nanoparticle research*, *15*(6), 1741.

Jiao, L., Cao, D. P., Qin, L. P., Han, T., Zhang, Q. Y., Zhu, Z., & Yan, F. (2009). Antiosteoporotic activity of phenolic compounds from *Curculigo orchioides*. *Phytomedicine*, *16*(9), 874-881.

Jitendra, J., Vineeta, T., Ashok, K., Brijesh, K., & Singh, P. (2012)*. Rosa centifolia*: plant review. *Int J Res Pharm Chem*, *2*(3), 794-796.

Jobitha, G. D. G., Kannan, C., & Annadura, G. (2012). A facile Phyto-assisted synthesis of silver nanoparticles using the flower extract of *Cassia auriculata* and assessment of its antimicrobial activity. *Drug Invention Today*, *4*(11), 579-584.

Johnson, M., Wesely, E. G., Hussain, M. Z., & Selvan, N. (2010). In vivo and in vitro phytochemical and antibacterial efficacy of *Baliospermum montanum* (Wïlld.) Muell. Arg. *Asian Pacific Journal of Tropical Medicine*, *3*(11), 894-897.

Johri, R. K. (2011). *Cuminum cyminum* and *Carum carvi*: An update. *Pharmacognosy reviews*, *5*(9), 63.

Joseph, B., & Raj, S. J. (2010). Phytopharmacological and phytochemical properties of three *Ficus* species- an overview. *International Journal of Pharma and Bio Sciences*, *1*(4), 246-253.

Joseph, N., Anjum, N., & Tripathi, Y. C. (2016). Phytochemical screening and evaluation of polyphenols, Flavonoids and Antioxidant Activity of *Prunus cerasoides* D. Don Leaves. *Journal of Pharmacy Research Vol*, *10*(7), 502-508.

Joshi, B., Sah, G. P., Basnet, B. B., Bhatt, M. R., Sharma, D., Subedi, K., Janardhan, P., & Malla, R. (2011). Phytochemical extraction and antimicrobial properties of different medicinal plants: *Ocimum sanctum* (Tulsi), *Eugenia caryophyllata* (Clove), *Achyranthes bidentata* (Datiwan) and *Azadirachta indica* (Neem). *Journal of Microbiology and Antimicrobials*, *3*(1), 1-7.

Joshi, R. K. (2013). Chemical composition, in vitro antimicrobial and antioxidant activities of the essential oils of *Ocimum gratissimum*, *O. sanctum* and their major constituents. *Indian journal of pharmaceutical sciences*, *75*(4), 457-462.

Joshi, S. (2014). Spectral characterization, stereochemical assignments and thermal rearrangements of naturally occurring furanogermacranes and furanoelemanes. *J. Nat. Prod. Plant Resour*, *4*(5), 24-31.

Joshi, S. C., & Mathela, C. S. (2012). Antioxidant and antibacterial activities of the leaf essential oil and its constituents furanodienone and curzerenone from *Lindera pulcherrima* (Nees.) Benth. ex hook. f. *Pharmacognosy research*, *4*(2), 80-84.

Joshi, S., Mishra, D., Bisht, G., & Khetwal, K. S. (2011). Essential oil composition and antimicrobial activity of *Lobelia pyramidalis* Wall. *EXCLI journal*, *10*, 274.

Jung, M., & Park, M. (2007). Acetylcholinesterase inhibition by flavonoids from Agrimonia pilosa. *Molecules*, *12*(9), 2130-2139.

Jung, M., Park, M., Lee, H. C., Kang, Y. H., Kang, E. S., & Kim, S. K. (2006). Antidiabetic agents from medicinal plants. *Current medicinal chemistry*, *13*(10), 1203-1218.

Jurenka, J. S. (2009). Anti-inflammatory properties of curcumin, a major constituent of *Curcuma longa*: a review of preclinical and clinical research. *Alternative medicine review*, *14*(2), 141-153.

Jürgens, A., Dötterl, S., Liede-Schumann, S., & Meve, U. (2008). Chemical diversity of floral volatiles in Asclepiadoideae-Asclepiadeae (Apocynaceae). *Biochemical Systematics and Ecology*, *36*(11), 842-852.

Jürgens, A., Witt, T., & Gottsberger, G. (2002). Flower scent composition in night-flowering *Silene* species (Caryophyllaceae). *Biochemical Systematics and Ecology*, *30*(5), 383-397.

Jurikova, T., Rop, O., Mlcek, J., Sochor, J., Balla, S., Szekeres, L., Hegedusova, A., Hubalek, J., Adam, V., & Kizek, R. (2011). Phenolic profile of edible honeysuckle berries (genus *Lonicera*) and their biological effects. *Molecules*, *17*(1), 61-79.

Jyothi, K. S., & Seshagiri, M. (2012). In-vitro activity of saponins of *Bauhinia purpurea*, *Madhuca longifolia*, *Celastrus paniculatus* and *Semecarpus anacardium* on selected oral pathogens. *Journal of Dentistry (Tehran, Iran)*, *9*(4), 216-223.

Kabdwal, L., & Verma, D. L. (2010). 3-Methoxy flavones from *cheilanthes bicolor*. *Nature and Science*, *8*(10), 569-571.

Kabra, A., Kabra, R., & Baghel, U. S. (2015). *Premna* Species: A Review. *Journal of Biological and Chemical Chronicals*, *1*(1), 55-59.

Kadam, P. A. R. A. G., & Bodhankar, S. L. (2013). Analgesic and anti-inflammatory activity of seed extracts of *Diplocyclos palmatus* (L) C. Jeffrey. *International Journal of Pharma and Bio Science*, *4*, 970-978.

Kalia, K., Sharma, K., Singh, H. P., & Singh, B. (2008). Effects of extraction methods on phenolic contents and antioxidant activity in aerial parts of Potentilla atrosanguinea Lodd. and quantification of its phenolic constituents by RP-HPLC. *Journal of agricultural and food chemistry*, *56*(21), 10129-10134.

Kamboj, A., & Saluja, A. (2010). Phytopharmacological review of *Xanthium strumarium* L.(Cocklebur). *International journal of green pharmacy*, *4*(3), 129-139.

Kamboj, A., & Saluja, A. K. (2009). *Bryophyllum pinnatum* (Lam.) Kurz.: Phytochemical and pharmacological profile: A review. *Pharmacognosy Reviews*, *3*(6), 364-374.

Kaminidevi, S., Thangavelu, T., Lakshmanan, A., Gunashekar, D., Chirayil, H. T., Sukumaran, J. K., & Murugan, S. (2014). Preliminary phytochemical screening and antimicrobial activity of fresh plant extract of Indian folk medicinal plant, *Gnaphalium polycaulon*. *International Journal of Phytomedicine*, *6*(1), 82.

Kanter, M., Coskun, O., & Budancamanak, M. (2005). Hepatoprotective effects of Nigella sativa L and *Urtica dioica* L on lipid peroxidation, antioxidant enzyme systems and liver enzymes in carbon tetrachloride-treated rats. *World journal of gastroenterology,* *11*(42), 6684.

Karmakar, U. K., Ghosh, D., & Sadhu, S. K. (2011). Assessment of analgesic, cytotoxic and antioxidant activities of *Vallaris solanacea* (Roth) Kuntze. *Stamford Journal of Pharmaceutical Sciences*, *4*(1), 64-68.

Karou, D., Savadogo, A., Canini, A., Yameogo, S., Montesano, C., Simpore, J., Colizzi, V., & Traore, A. S. (2005). Antibacterial activity of alkaloids from Sida acuta. *African Journal of Biotechnology*, *4*(12), 195-200.

Karthika, K., Jamuna, S., & Paulsamy, S. (2014). TLC and HPTLC Fingerprint Profiles of Different Bioactive Components from the Tuber of *Solena amplexicaulis*. *Journal of Pharmacognosy and Phytochemistry*, *3*(1), 198-206.

Karthishwaran, K., Mirunalini, S., Dhamodharan, G., Krishnaveni, M., & Arulmozhi, V. (2010). Phytochemical investigation of methanolic extract of the leaves of *Pergularia daemia*. *J Biol Sci*, *10*(3), 242-246.

Katiki, L. M., Chagas, A. C. S., Bizzo, H. R., Ferreira, J. F. S., & Amarante, A. F. T. D. (2011). Anthelmintic activity of *Cymbopogon martinii*, *Cymbopogon schoenanthus* and *Mentha piperita* essential oils evaluated in four different in vitro tests. *Veterinary Parasitology*, *183*(1-2), 103-108.

Kaur, G. J., & Arora, D. S. (2009). Antibacterial and phytochemical screening of *Anethum graveolens, Foeniculum vulgare* and *Trachyspermum ammi*. *BMC complementary and alternative medicine*, *9*(1):30.

Kaur, M., Singh, J., Kamboj, S. S., Singh, J., Kaur, A., Sood, S. K., & Saxena, A. K. (2005). Isolation and characterization of two N-acetyl-D-lactosamine specific lectins from tubers of *Arisaema intermedium* Blume and *A. wallichianum* Hook f.

Kaushik, P., Goyal, P., Chauhan, A., & Chauhan, G. (2010). In vitro evaluation of antibacterial potential of dry fruitextracts of *Elettaria cardamomum* Maton (Chhoti Elaichi). *Iranian journal of pharmaceutical research, 9*(3), 287-292.

Kaushik, P., Kaushik, D., & Khokra, S. L. (2013). Ethnobotany and phytopharmacology of *Pinus roxburghii* Sargent: a plant review. *Journal of integrative medicine*, *11*(6), 371-376.

Kaushik, V., Chaudhary, G., Ahmad, S., & Saini, V. (2016). Evaluation of antimicrobial potential of Anemone obtusiloba D. Don.*Der Pharmacia Lettre*, *8*(4), 273-276.

Kavit, M., Patel, B. N., & Jain, B. K. (2013). Phytochemical analysis of leaf extract of Phyllanthus fraternus. *Research Journal of Recent Sciences, 2*, 12-15.

Kavitha, C., Rajamani, K., & Vadivel, E. (2010). *Coleus forskohlii* A comprehensive review on morphology, phytochemistry and pharmacological aspects. *Journal of Medicinal Plants Research*, *4*(4), 278-285.

Kazeem, M. I., Abimbola, S. G., & Ashafa, A. O. T. (2013). Inhibitory potential of *Gossypium arboreum* leaf extracts on diabetes key enzymes,α-amylase and α-glucosidase. *Bangladesh Journal of Pharmacology*, *8*(2), 149-155.

Keawpradub, N., Kirby, G. C., Steele, J. C. P., & Houghton, P. J. (1999). Antiplasmodial activity of extracts and alkaloids of three *Alstonia* species from Thailand. *Planta medica*, *65*(8), 690-694.

Keawsa-ard, S., & Kongtaweelert, S. (2012). Antioxidant, antibacterial, anticancer activities and chemical constituents of the essential oil from *Mesua ferrea* leaves. *Chiang Mai J. Sci*, *39*(3), 455-463.

Kenwat, R., Prasad, P., Sahu, R. K., Roy, A., & Saraf, S. (2014). Preliminary phytochemical screening and in vitro antioxidant efficacy of fruit oil of *Martynia annua*. *UK Journal of Pharmaceutical and Biosciences*, *2*(1), 16-22.

Keusgen, M., Fritsch, R. M., Hisoriew, H., Kurbonova, P. A. & Khassanov, F. O. (2006). Wild *Allium* species (Alliaceae) used in folk medicine of Tajikistan and Uzbekistan. *Journal of Ethnobiology and Ethnomedicine*, *2*(18), 1-9.

Khan, H., Saeed, M., & Muhammad, N. (2012). Pharmacological and phytochemical updates of genus *Polygonatum*. *Phytopharmacology*, *3*(2), 286-308.

Khan, H., Saeed, M., Khan, M. A., Dar, A., & Khan, I. (2010). The antinociceptive activity of *Polygonatum verticillatum* rhizomes in pain models. *Journal of ethnopharmacology*, *127*(2), 521-527.

Khan, H., Saeed, M., Muhammad, N., Ghaffar, R., Khan, S. A., & Hassan, S. (2012). Antimicrobial activities of rhizomes of *Polygonatum verticillatum*: attributed to its total flavonoidal and phenolic contents. *Pakistan journal of pharmaceutical sciences*, *25*(2), 463-467.

Khan, M. & Younus, T. 2011. Prevention of CCL_4_- induced oxidative damage in adrenal gland by *Digera muricata* extract in rat. *Pak. J. Pharm. Sci*. *24*(4), 469-473.

Khan, M. S. Y., Bano, S., Javed, K., & Mueed, M. A. (2006). A comprehensive review on the chemistry and pharmacology of Corchorus species—a source of cardiac glycosides, triterpenoids, ionones, flavonoids, coumarins, steroids and some other compounds. Journal of Scientific and Industrial Research, *65*, 283-298.

Khan, M., Giessrigl, B., Vonach, C., Madlener, S., Prinz, S., Herbaceck, I., Hölzl, C., Bauer, S., Viola, K., Mikulits, W. & Quereshi, R. A. (2010). Berberine and a *Berberis lycium* extract inactivate Cdc25A and induce α-tubulin acetylation that correlate with HL-60 cell cycle inhibition and apoptosis. *Mutation Research/Fundamental and Molecular Mechanisms of Mutagenesis*, *683*(1), 123-130.

Khan, R. A., Khan, M. R., Sahreen, S., & Ahmed, M. (2012). Evaluation of phenolic contents and antioxidant activity of various solvent extracts of *Sonchus asper* (L.) Hill. *Chemistry Central Journal*, *6*(1), 12.

Khan, T., Ahmad, M., Khan, H., & Khan, M. A. (2005). Biological activities of aerial parts of Paeonia emodi Wall. *African Journal of Biotechnology*, *4*(11).

Khanna, V. G., & Kannabiran, K. (2007). Larvicidal effect of *Hemidesmus indicus, Gymnema sylvestre, and Eclipta prostrata* against Culex qinquifaciatus mosquito larvae. *African Journal of Biotechnology*, *6*(3), 307-311

Khulbe, K., & Sati, S. C. (2009). Antibacterial activity of *Boenninghausenia albiflora* Reichb.(Rutaceae). *African Journal of Biotechnology*, *8*(22), 6346-6348.

Khursheed, A., Pathak, D. & Ansari, S. H. (2010). Phytochemical and pharmacological investigations of *Adhatoda zeylanica* (Medic.): A review. *Pharmacognosy Journal*, *2*, 513-519.

Kidmose, U., Hansen, S. L., Christensen, L. P., Edelenbos, M., Larsen, E., & Nørbæk, R. (2004). Effects of genotype, root size, storage, and processing on bioactive compounds in organically grown carrots (*Daucus carota* L.). *Journal of Food Science*, *69*(9).

Kilani, S., Sghaier, M. B., Limem, I., Bouhlel, I., Boubaker, J., Bhouri, W., Skandrani, I., Neffatti, A., Ammar, R. B., Dijoux-Franca, M. G & Ghedira, K. (2008). In vitro evaluation of antibacterial, antioxidant, cytotoxic and apoptotic activities of the tubers infusion and extracts of *Cyperus rotundus*. *Bioresource technology*, *99*(18), 9004-9008.

Kim, C. S., Alamgir, K. M., Matsumoto, S., Tebayashi, S. I., & Koh, H. S. (2008). Antifeedants of Indian barnyard millet, *Echinochloa frumentacea* Link, against brown planthopper, Nilaparvata lugens (Stål). *Zeitschrift für Naturforschung C*, *63*(9-10), 755-760.

Kim, K. H., Moon, E., Kim, S. Y., & Lee, K. R. (2010). Lignans from the tuber-barks of *Colocasia antiquorum* var. *esculenta* and their antimelanogenic activity. *Journal of agricultural and food chemistry*, *58*(8), 4779-4785.

Kimbaris, A. C., Koliopoulos, G., Michaelakis, A., & Konstantopoulou, M. A. (2012). Bioactivity of *Dianthus caryophyllus, Lepidium sativum, Pimpinella anisum*, and *Illicium verum* essential oils and their major components against the West Nile vector Culex pipiens. *Parasitology research*, *111*(6), 2403-2410.

Kintz, P., Villain, M., Bargul, Y., Charlot, J. Y., & Cirimele, V. (2006). Testing for atropine and scopolamine in hair by LC-MS-MS after *Datura inoxia* abuse. *Journal of analytical toxicology*, *30*(7), 454-457.

Kirana, H., & Srinivasan, B. P. (2008). *Trichosanthes cucumerina* Linn. improves glucose tolerance and tissue glycogen in non insulin dependent diabetes mellitus induced rats. *Indian journal of pharmacology*, *40*(3), 103-106.

Kiruba, S., Mahesh, M., Nisha, S. R., Paul, Z. M., & Jeeva, S. (2011). Phytochemical analysis of the flower extracts of *Rhododendron arboreum* Sm. ssp. nilagiricum (Zenker) Tagg. *Asian Pacific Journal of Tropical Biomedicine*, *1*(2), 284-S286.

Kiuchi, F., Fukao, Y., Maruyama, T., Obata, T., Tanaka, M., Sasaki, T., Mikage, M., Haque, M. E. & Tsuda, Y. (1998). Cytotoxic principles of a Bangladeshi crude drug, akond mul (roots of *Calotropis gigantea* L.). *Chemical and pharmaceutical bulletin*, *46*(3), 528-530.

Kojima, M., & Takeuchi, W. (1989). Detection and characterization of p-coumaric acid hydroxylase in mung bean, *Vigna mungo*, seedlings. *The Journal of Biochemistry*, *105*(2), 265-270.

Kokanova-Nedialkova, Z., Bücherl, D., Nikolov, S., Heilmann, J., & Nedialkov, P. T. (2011). Flavonol glycosides from *Chenopodium foliosum* Asch. *Phytochemistry letters*, *4*(3), 367-371.

Kokanova-Nedialkova, Z., Nedialkov, P. T., & Nikolov, S. D. (2009). The genus *Chenopodium*: phytochemistry, ethnopharmacology and pharmacology. *Pharmacognosy Reviews*, *3*(6), 280.

Kokila, K., Priyadharshini, S. D., & Sujatha, V. (2013). Phytopharmacological properties of *Albizia* species: a review. *Int J Pharm Pharm Sci*, *5*(5), 70-73.

Kostova, I., & Iossifova, T. (2007). Chemical components of Fraxinus species. *Fitoterapia*, *78*(2), 85-106.

Kothiyal, S. K, Sati, S. C., Rawat, M. S.M., Sati, M. D., Semwal, D. K., Semwal, R. B., Sharma, A., Rawat, B. & Kumar, A. (2012). Chemical constituents and biological significance of the genus *Ilex* (Aquifoliaceae). *The Natural Products Journal*, *2*(3): 212-224.

Kou, L., Kang, S. H., & Gu, X. B. (2008). Supercritical CO_2 Fluid Extraction of *Rubus biflorus* Buch and GC-MS Analysis [J]. *Journal of Northwest University for Nationalities,* 2.

Kour, A., Shawl, A. S., Rehman, S., Sultan, P., Qazi, P. H., Suden, P., Khajuria, R. K. & Verma, V. (2008). Isolation and identification of an endophytic strain of Fusarium oxysporum producing podophyllotoxin from *Juniperus recurva*. *World Journal of Microbiology and Biotechnology*, *24*(7), 1115-1121.

Krewson, C. F., Ard, J. S., & Riemenschneider, R. W. (1962). *Vernonia anthelmintica* (L.) Willd. trivernolin, 1, 3-divernolin and vernolic (Epoxyoleic) acid from the seed oil. *Journal of the American Oil Chemists Society*, *39*(7), 334-340.

Krishnamoorthy, K., & Subramaniam, P. (2014). Phytochemical profiling of leaf, stem, and tuber parts of *Solena amplexicaulis* (Lam.) Gandhi Using GC-MS. *International scholarly research notices*.

Kulandaivel, S., Bajpai, P., & Sivakumar, T. (2013). Anti-hyperglycemic activity of *Trichosanthes tricuspidata* root extract. *Bangladesh Journal of Pharmacology*, *8*(3), 305-310.

Kumar Roy, M., Nakahara, K., Na Thalang, V., Trakoontivakorn, G., Takenaka, M., Isobe, S., & Tsushida, T. (2007). Baicalein, a flavonoid extracted from a methanolic extract of *Oroxylum indicum* inhibits proliferation of a cancer cell line in vitro via induction of apoptosis. *Die Pharmazie-An International Journal of Pharmaceutical Sciences*, *62*(2), 149-153.

Kumar, A., Chowdhury, S. R., Sarkar, T., Chakrabarti, T., Majumder, H. K., Jha, T., & Mukhopadhyay, S. (2016). A new bisbenzylisoquinoline alkaloid isolated from *Thalictrum foliolosum*, as a potent inhibitor of DNA topoisomerase IB of Leishmania donovani. *Fitoterapia*, *109*, 25-30.

Kumar, A., Lingadurai, S., Jain, A., & Barman, N. R. (2010). *Erythrina variegata* Linn: A review on morphology, phytochemistry, and pharmacological aspects. *Pharmacognosy reviews*, *4*(8):147-152.

Kumar, D., Mallick, S., Vedasiromoni, J. R., & Pal, B. C. (2010). Anti-leukemic activity of *Dillenia indica* L. fruit extract and quantification of betulinic acid by HPLC. *Phytomedicine*, *17*(6), 431-435.

Kumar, N., Bhandari, P., Singh, B., & Bari, S. S. (2009). Antioxidant activityand ultra-performance LC- electrospray ionization-quadrupole time-of-flight mass spectrometry for phenolics- based fingerprinting of Rose species: *Rosa damascene, Rosa bourboniana* and *Rosa damascene*. *Food and Chemical toxicology*, *47*(2), 361-367.

Kumar, R. A., Sridevi, K., Kumar, N. V., Nanduri, S., & Rajagopal, S. (2004). Anticancer and immunostimulatory compounds from *Andrographis paniculata*. *Journal of ethnopharmacology*, *92*(2-3), 291-295.

Kumar, S. (2014). Adulteration and substitution in endangered, costly herbal medicinal plants of India, investigates their active phytochemical constituents. *International Journal of Pharmacy & Therapeutics*, *5*(4), 243-260.

Kumar, S., & Rana, B. K. (2017). Screening and isolation of potential antibacterial compounds from dried leaves of Aconogonum tortuosum. *International Education and Research Journal*, *3*(3), 24-25.

Kumar, V., Bhat, Z. A., Kumar, D., Khan, N. A., & Chashoo, I. A. (2012). Evaluation of anti-inflammatory potential of leaf extracts of *Skimmia anquetilia.* *Asian pacific journal of tropical biomedicine*, *2*(8), 627-630.

Kumar, V., Mathela, C. S., Tewari, G., Panwar, A., & Pandey, V. (2017). In vitro antimicrobial activity of essential oils and their acetylenic constituents. *Indian Journal of Natural Products and Resources, 8*(1), 63-68.

Kumar, V., Mathela, C. S., Tewari, G., Panwar, A., & Pandey, V. (2017). In vitro antimicrobial activity of essential oils and their acetylenic constituents. *Indian Journal of Natural Products and Resources,* *8*(1), 63-68.

Kumar, V., Mukherjee, K., Kumar, S., Mal, M., & Mukherjee, P. K. (2008). Validation of HPTLC method for the analysis of taraxerol in *Clitoria ternatea*. *Phytochemical Analysis*, *19*(3), 244-250.

Kumarappan, C., Srinivasan, R., Jeevathayaparan, S., Rajinikant, R., Kumar, H. S., & Senthilrajan, S. (2015). *Ichnocarpus frutescens*: A valuable medicinal plant. *Pharmacol Online*, 2, 18-37.

Kumarasamy, Y., Nahar, L., Byres, M., Delazar, A., & Sarker, S. D. (2005). The assessment of biological activities associated with the major constituents of the methanol extract of ‘wild carrot’(*Daucus carota* L.) seeds. *Journal of herbal pharmacotherapy*, *5*(1), 61-72.

Kumaraswamy, M., Kavitha, H., & Satish, S. 2008. Antibacterial evaluation and phytochemical analysis of *Betula utilis* D. Don against some human pathogenic bacteria. *Advances in Biological Research*, *2*(1-2), 21-25.

Küpeli, E., Harput, U. S., Varel, M., Yesilada, E., & Saracoglu, I. (2005). Bioassay-guided isolation of iridoid glucosides with antinociceptive and anti-inflammatory activities from *Veronica anagallis-aquatica* L. *Journal of ethnopharmacology*, *102*(2), 170-176.

Kurian, S. U. S. A. N., & Josekumar, V. S. (2017). Phytochemical screening, antimicrobial activity and brine shrimp lethality bioassay of different extracts of *Alysicarpus vaginalis* Var. *Nummularifolius* (DC.) MIQ.(Family: Fabaceae). *Int J Pharm Pharm Sci*, *9*(1), 1-6.

Laekeman, G., & Vlietinck, A. (2013). Phytoecdysteroids: phytochemistry and pharmacological activity. *Journal of Natural Products*, *71*(7), 1294-1296.

Lai, H. Y., Lim, Y. Y., & Kim, K. H. (2010). Blechnum orientale Linn-a fern with potential as antioxidant, anticancer and antibacterial agent. *BMC complementary and alternative medicine*, *10*(1), 15.

Lai, S. C., Ho, Y. L., Huang, S. C., Huang, T. H., Lai, Z. R., Wu, C. R., Lian, K. Y. & Chang, Y. S. (2010). Antioxidant and antiproliferative activities of *Desmodium triflorum* (L.) DC. *The American journal of Chinese medicine*, *38*(2), 329-342.

Lakshmi, K. R., Supraja, M., Mounika, M., & Babu, P. S. (2016). A review on hypoglycaemic activity of different extracts of various medicinal plants. *International Journal of Pharmaceutical Sciences and Research*, *7*(8), 3173-3184.

Lan, Y., Chi, X., Zhou, G., & Zhao, X. (2018). Antioxidants from Pedicularis longiflora var. tubiformis (Klotzsch) PC Tsoong. *Records of Natural Products*, *12*(4), 332-339.

Larrosa, M., Gonzalez-Sarrias, A., Garcia-Conesa, M.T., Tomas-Barberan, F.A., Espin, J.C., (2006). Urolithins, ellagic acid-derived metabolites produced by human colonic microflora, exhibit estrogenic and antiestrogenic activities. Journal of Agricultural and Food Chemistry 54, 1611–1620. Lee, C.J., Chen, L.G., Liang, W.L., Wang, C.C., 2010.

Lee, N. J., Choi, J. H., Koo, B. S., Ryu, S. Y., Han, Y. H., Lee, S. I., & Lee, D. U. (2005). Antimutagenicity and cytotoxicity of the constituents from the aerial parts of *Rumex acetosa*. *Biological and Pharmaceutical Bulletin*, *28*(11), 2158-2161.

Lepcha, L., Mandal, P., Misra, T. K., & Sharma, N. P. (2010). Comparative study of Plant Biodiversity and Physico-chemical Parameters of Soils of Landslide Prone areas. *International Journal of Ecology & Development*, *17*(10), 66-76.

Leu, Y. L., Kuo, S. M., Hwang, T. L., & Chiu, S. T. (2004). The inhibition of superoxide anion generation by neutrophils from *Viscum articulactum*. *Chemical and pharmaceutical bulletin*, *52*(7), 858-860.

Lewis, C. E., Walker, J. R., Lancaster, J. E., & Sutton, K. H. (1998). Determination of anthocyanins, flavonoids and phenolic acids in potatoes. I: Coloured cultivars of Solanum tuberosum L. *Journal of the Science of Food and Agriculture*, *77*(1), 45-57.

Li, D. H., Li, J. Y., Xue, C. M., Han, T., Sai, C. M., Wang, K. B., Lu, J. C., Jing, Y. K., Hua, H. M., & Li, Z. L. (2017). Antiproliferative Dimeric Aporphinoid Alkaloids from the Roots of *Thalictrum cultratum*. *Journal of Natural Products*, *80*(11), 2893-2904.

Li, J. Z., Qing, C., Chen, C. X., Hao, X. J., & Liu, H. Y. (2009). Cytotoxicity of cardenolides and cardenolide glycosides from *Asclepias curassavica*. *Bioorganic & medicinal chemistry letters*, *19*(7), 1956-1959.

Li, J., Wang, Z. W., Zhang, L., Liu, X., Chen, X. H., & Bi, K. S. (2008). HPLC analysis and pharmacokinetic study of quercitrin and isoquercitrin in rat plasma after administration of *Hypericum japonicum* thunb. extract. *Biomedical Chromatography*, *22*(4), 374-378.

Li, L., Li, M. H., Xu, L. J., Guo, N., Wu-Lan, T. N., Shi, R. B., Peng, Y. & Xiao, P. G. (2010). Distribution of seven polyphenols in several medicinal plants of Boraginaceae in China. *Journal of Medicinal Plants Research*, *4*(12), 1216-1221.

Li, S. P., Zhao, K. J., Ji, Z. N., Song, Z. H., Dong, T. T., Lo, C. K., Cheung, J.K., Zhu, S.Q. & Tsim, K. W. (2003). A polysaccharide isolated from *Cordyceps sinensis*, a traditional Chinese medicine, protects PC12 cells against hydrogen peroxide-induced injury. *Life sciences*, *73*(19), 2503-2513.

Li, X. H., & McLaughlin, J. L. (1989). Bioactive compounds from the root of *Myrsine africana*. *Journal of natural products*, *52*(3), 660-662.

Li, Y., Wu, J., Shi, R., Li, N., Xu, Z., & Sun, M. (2017). Antioxidative effects of *Rhodiola* genus: Phytochemistry and pharmacological mechanisms against the diseases. *Current topics in medicinal chemistry*, *17*(15), 1692-1708.

Liang, H. X., Dai, H. Q., Fu, H. A., Dong, X. P., Adebayo, A. H., Zhang, L. X., & Cheng, Y. X. (2010). Bioactive compounds from Rumex plants. *Phytochemistry Letters*, *3*(4), 181-184.

Liao, H., Dong, W., Shi, X., Liu, H., & Yuan, K. (2012). Analysis and comparison of the active components and antioxidant activities of extracts from *Abelmoschus esculentus* L. *Pharmacognosy magazine*, *8*(30), 156-161.

Lin, F. J., Yen, F. L., Chen, P. C., Wang, M. C., Lin, C. N., Lee, C. W., & Ko, H. H. (2014). HPLC-fingerprints and antioxidant constituents of *Phyla nodiflora*. *The Scientific World Journal*.

Lin, J. H. (1993). Anaphaloside, a New Acyl Flavonoid Glycoside from the Flowers of *Anaphalis Contorts* Hooker. *Journal of the Chinese chemical society*, *40*(1), 93-95.

Lin, J. H., Lin, Y. M., & Chen, F. C. 1976. A New Kaempferol‐3‐β‐D‐(6 ″‐0‐P‐Coumaroyl)‐Glucoside from Flowers of *Anaphalis Contorta* Hooker. *Journal of the Chinese Chemical Society*, *23*(1), 57-59.

Lin, L., Ni, B., Lin, H., Zhang, M., Li, X., Yin, X., Qu, C., & Ni, J. (2015). Traditional usages, botany, phytochemistry, pharmacology and toxicology of *Polygonum multiflorum* Thunb.: a review. *Journal of Ethnopharmacology*, *159*, 158-183.

Lin, S. Y., Wang, C. C., Lu, Y. L., Wu, W. C., & Hou, W. C. (2008). Antioxidant, anti-semicarbazide-sensitive amine oxidase, and anti-hypertensive activities of geraniin isolated from *Phyllanthus urinaria*. *Food and Chemical Toxicology*, *46*(7), 2485-2492.

Liu, X. C., Zhou, L., & Liu, Z. L. (2013). Identification of insecticidal constituents from the essential oil of *Valeriana jatamansi* Jones against Liposcelis bostrychophila Badonnel. *Journal of Chemistry*.

Liu, Y., Murakami, N., Ji, H., Abreu, P., & Zhang, S. (2007). Antimalarial Flavonol Glycosides from *Euphorbia hirta*. *Pharmaceutical Biology*, *45*(4), 278-281.

Lo Cantore, P., Iacobellis, N. S., De Marco, A., Capasso, F., & Senatore, F. (2004). Antibacterial activity of Coriandrum sativum L. and *Foeniculum vulgare* Miller var. *vulgare* (Miller) essential oils. *Journal of agricultural and food chemistry*, *52*(26), 7862-7866.

Lu, C., Li, Y., Li, L., Liang, L., & Shen, Y. (2012). Anti-inflammatory activities of fractions from *Geranium nepalense* and related polyphenols. *Drug discoveries & therapeutics*, *6*(4), 194-197.

Lukmandaru, G., & Takahashi, K. (2009). Radial distribution of quinones in plantation teak (*Tectona grandis* Lf). *Annals of Forest Science*, *66*(6), 605-605.

Ma, X., Zheng, C., Hu, C., Rahman, K., & Qin, L. (2011). The genus *Desmodium* (Fabaceae)-traditional uses in Chinese medicine, phytochemistry and pharmacology. *Journal of ethnopharmacology*, *138*(2), 314-332.

Maas, M., Hensel, A., da Costa, F. B., Brun, R., Kaiser, M., & Schmidt, T. J. (2011). An unusual dimeric guaianolide with antiprotozoal activity and further sesquiterpene lactones from *Eupatorium perfoliatum*. *Phytochemistry*, *72*(7), 635-644.

Määttä-Riihinen, K. R., Kamal-Eldin, A., & Törrönen, A. R. (2004). Identification and quantification of phenolic compounds in berries of *Fragaria* and *Rubus* species (family Rosaceae). *Journal of Agricultural and Food Chemistry*, *52*, 6178-6187.

Macías, F. A., López, A., Varela, R. M., Torres, A., & Molinillo, J. M. (2004). Bioactive lignans from a cultivar of Helianthus annuus. *Journal of agricultural and food chemistry*, *52*(21), 6443-6447.

Madan, S., Gullaiya, S., Singh, G. N., & Kumar, Y. (2013). Flemingia strobilifera: review on phytochemistry and pharmacological aspects. *International Journal of Phytopharmacology*, *4*(4), 255-262.

Madhusudanan, K. P., Banerjee, S., Khanuja, S. P., & Chattopadhyay, S. K. (2008). Analysis of hairy root culture of *Rauvolfia serpentina* using direct analysis in real time mass spectrometric technique. *Biomedical Chromatography*, *22*(6), 596-600.

Maganha, E. G., da Costa Halmenschlager, R., Rosa, R. M., Henriques, J. A. P., de Paula Ramos, A. L. L., & Saffi, J. (2010). Pharmacological evidences for the extracts and secondary metabolites from plants of the genus *Hibiscus*. *Food Chemistry*, *118*(1), 1-10.

Mai, N. T., Tuan, T. A., Huong, H. T., Minh, C. V., Ban, N. K., & Kiem, P. V. (2014). Bisbenzylisoquinoline alkaloids from *Mahonia nepalensis*. *Vietnam Journal of Chemistry*, *47*(3), 368.

Maisuthisakul, P., Pasuk, S. & Ritthiruangdej, P. (2008). Relationship between antioxidant properties and chemical composition of some Thai plants. *Journal of Food Composition and Analysis*, *21*(3), 229-240.

Maiyo, Z. C., Ngure, R. M., Matasyoh, J. C. & Chepkorir, R. (2010). Phytochemical constituents and antimicrobial activity of leaf extract of three Amaranthus plant species, *African Journal of Biotechnology*, *9*(21), 3178-3182.

Majumder, P., Sarkar, A. K., & Chakraborti, J. (1982). Isoflavidinin and iso-oxoflavidinin, two 9, 10-dihydrophenanthrenes from the orchids *Pholidota articulata*, *Otochilus porecta* and *Otochilus fusca*. *Phytochemistry*, *21*(11), 2713-2716.

Makheswari, M. U., & Sudarsanam, D. (2012). Database on antidiabetic indigenous plants of Tamil Nadhu, India. *Int J Pharm Sci Res*, *3*, 287-93.

Makino, T., Furuta, Y., Fujii, H., Nakagawa, T., Wakushima, H., Saito, K. I., & Kano, Y. (2001). Effect of oral treatment of *Perilla frutescens* and its constituents on type-I allergy in mice. *Biological and Pharmaceutical Bulletin*, *24*(10), 1206-1209.

Mali, P. Y., & Bhadane, V. V. (2010). Comparative account of screening of bioactive ingredients of *Premna integrifolia* Linn. with special reference to root by using various solvents. *J Pharm Res*, *3*, 1677-1679.

Malviya, S., Rawat, S., Kharia, A., & Verma, M. (2011). Medicinal attributes of *Acacia nilotica* Linn.-A comprehensive review on ethnopharmacological claims. *International Journal of Pharmacy & Life Sciences*, *2*(6), 830-837.

Mandal, G. D., & Nandi, A. K. (2013). Comparative pharmacognostic and phytochemical studies of two aphrodisiac plants–*Chlorophytum borivilianum* Santapau & Fernandes and *Chlorophytum tuberosum* (Roxb.) Baker. *International Journal of Pharmacy and Pharmaceutical Sciences*, *5*(4), 517-523.

Mandal, P., Misra, T. K., & Ghosal, M. (2009). Free-radical scavenging activity and phytochemical analysis in the leaf and stem of *Drymaria diandra* Blume. *Int J Integr Biol*, *7*(2), 80-84.

Manisha, P., Kanchan, S., Jovita, K., Koshy, M. K., & Shubhini, A. S. (2009). *Sida Veronicaefolia* as a source of natural antioxidant. *International Journal of Pharmaceutical Sciences and Drug Research*, *1*(3), 180-182.

Maniyar, Y., Bhixavatimath, P., & Agashikar, N. V. (2010). Antidiarrheal activity of flowers of Ixora coccinea Linn. in rats. *Journal of Ayurveda and integrative medicine*, *1*(4), 287-291.

Manjunath, M., Sharma, P. V. G. K. & Reddy, O. V. S. (2008). In vitro evaluation of antibacterial activity of *Actiniopteris radiate (Sw.) Link. J. Pharm Chem*, *2*(2), 112-117.

Manna, P., Bhattacharyya, S., Das, J., Ghosh, J., & Sil, P. C. (2011). Phytomedicinal role of Pithecellobium dulce against CCl4-mediated hepatic oxidative impairments and necrotic cell death. *Evidence-Based Complementary and Alternative Medicine*. doi:10.1093/ecam/neq065.

Marinova, D., Ribarova, F., & Atanassova, M. (2005). Total phenolics and total flavonoids in Bulgarian fruits and vegetables. *Journal of the university of chemical technology and metallurgy*, *40*(3), 255-260.

Martínez Vázquez, M., Ramírez Apan, T. O., Lazcano, M. E., & Bye, R. (1999). Anti-inflammatory active compounds from the n-hexane extract of *Euphorbia hirta*. *Journal of the Mexican Chemical Society*, *43*(3-4), 103-105.

Maruthupandian, A., & Mohan, V. R. (2011). GC-MS analysis of some bioactive constituents of *Pterocarpus marsupium* Roxb. *Int J Chem Tech Res*, *3*(3), 1652-1657.

Matasyoh, L. G., Matasyoh, J. C., Wachira, F. N., Kinyua, M. G., Muigai, A. W. T., & Mukiama, T. K. (2007). Chemical composition and antimicrobial activity of the essential oil of *Ocimum gratissimum* L. growing in Eastern Kenya. *African Journal of Biotechnology*, *6*(6), 760-765.

Materska, M., & Perucka, I. (2005). Antioxidant activity of the main phenolic compounds isolated from hot pepper fruit (*Capsicum annuum* L.). *Journal of Agricultural and Food Chemistry*, *53*(5), 1750-1756.

Mathappan, R., Joe, V. F., Prasanth, V. V., & Varirappan, K. (2010). Pharmacognostical and preliminary phytochemical studies of *Urena lobata* linn. *International Journal of Phytomedicine*, *2*(4), 408-411.

Mathela, C. S., Chanotiya, C. S., Sammal, S. S., Pant, A. K., & Pandey, S. (2005). Compositional diversity of terpenoids in the Himalayan Valeriana genera. *Chemistry & biodiversity*, *2*(9), 1174-1182.

Mathela, C. S., Joshi, R. K., Bisht, B. S., & Joshi, S. C. (2015). Nothoapiole and [alpha]-Asarone Rich Essential Oils from Himalayan *Pleurospermum angelicoides* Benth. *Records of Natural Products*, *9*(4), 546.

Mathela, C. S., Kharkwal, H., & Shah, G. C. (1994). Essential oil composition of some Himalayan Artemisia species. *Journal of Essential Oil Research*, *6*(4), 345-348.

Matic, M. (1956). The chemistry of plant cuticles: a study of cutin from *Agave americana* L. *Biochemical Journal*, *63*(1), 168.

Matsuda, H., Hirata, N., Kawaguchi, Y., Yamazaki, M., Naruto, S., Shibano, M. Masahiko Taniguchi, Kimiye Baba, & Kubo, M. (2005). Melanogenesis stimulation in murine B16 melanoma cells by umberiferae plant extracts and their coumarin constituents. *Biological and Pharmaceutical Bulletin*, *28*(7), 1229-1233.

Matthäus, B., & Fiebig, H. J. (1996). Simultaneous determination of isothiocyanates, indoles, and oxazolidinethiones in myrosinase digests of rapeseeds and rapeseed meal by HPLC. *Journal of agricultural and food chemistry*, *44*(12), 3894-3899.

Matthaus, B., & Özcan, M. M. (2014). Fatty acid, tocopherol and squalene contents of Rosaceae seed oils. *Botanical studies*, *55*(1), 48.

Maurya, R., Kaul, A., Bani, S., Zutshi, U., Khajuria, A., Saxena, A. K., Manahas, L. R., Kumar, Kapahi, B.K., Suri, O.P. & Qazi, G.N., (2003).  Pharmaceutical composition comprising extract from plant *Cryptolepis buchanani* for treating immunodeficiency. U.S. Patent 6,548,086, issued April 15, 2003.

McLaughlin, J. L., Miller, R. W., Powell, R. G., & Smith Jr, C. R. (1981). 19-Hydroxybaccatin III, 10-deacetylcephalomannine, and 10-deacetyltaxol: new antitumor taxanes from *Taxus wallichiana*. *Journal of Natural Products*, *44*(3), 312-319.

Meira, M., Silva, E. P. D., David, J. M., & David, J. P. (2012). Review of the genus *Ipomoea*: traditional uses, chemistry and biological activities. *Revista Brasileira de Farmacognosia*, *22*(3), 682-713.

Mir, M. A., Sawhney, S. S., & Jassal, M. M. S. (2013). Qualitative and quantitative analysis of phytochemicals of *Taraxacum officinale*. *Wudpecker Journal of Pharmacy and Pharmocology*, *2*(1):1-5.

Mir, S. A., Mishra, A. K., Reshi, Z. A., & Sharma, M. P. (2013). Preliminary phytochemical screening of some Pteridophytes from district Shopian (J&K). *International Journal of Pharmacy and Pharmaceutical Sciences*, *5*(4), 632-637.

Mir, S. A., Mishra, A. K., Reshi, Z. A., & Sharma, M. P. (2013). Preliminary phytochemical screening of some Pteridophytes from district Shopian (J&K). *International Journal of Pharmacy and Pharmaceutical Sciences*, *5*(4), 632-637.

Mishra, G., Srivastava, S., & Nagori, B. P. (2010). Pharmacological and therapeutic activity of Cissus quadrangularis: an overview. *International journal of pharmtech research*, *2*(2), 1298-1310.

Mishra, L. C., Singh, B. B., & Dagenais, S. (2000). Scientific basis for the therapeutic use of *Withania somnifera* (ashwagandha): a review. *Alternative medicine review*, *5*(4), 334-346.

Mishra, P. K., Shukla, R., Singh, P., Prakash, B., & Dubey, N. K. (2012). Antifungal and antiaflatoxigenic efficacy of *Caesulia axillaris* Roxb. essential oil against fungi deteriorating some herbal raw materials, and its antioxidant activity. *Industrial Crops and Products*, *36*(1), 74-80.

Mishra, R. K., Kumar, A., Shukla, A. C., Tiwari, P., & Dikshit, A. (2010). Quantitative and rapid antibacterial assay of *Micromeria biflora* Benth. leaf essential oil against Dental caries causing bacteria using phylogenetic approach. *Journal of Ecobiotechnology*, *2*(4), 22-26.

Mishra, T., Pal, M., Meena, S., Datta, D., Dixit, P., Kumar, A., Meena, B., Rana, T. S., & Upreti, D. K. (2016). Composition and in vitro cytotoxic activities of essential oil of *Hedychium spicatum* from different geographical regions of western Himalaya by principal components analysis. *Natural product research*, *30*(10), 1224-1227.

Mithraja, M. J., Marimuthu, J., Mahesh, M., Paul, Z. M., & Jeeva, S. (2011). Phytochemical studies on *Azolla pinnata* R. Br., *Marsilea minuta* L. and *Salvinia molesta* Mitch. *Asian Pacific Journal of Tropical Biomedicine*, *1*(1), 26-29.

Mitra, A., Sur, T. K., Upadhyay, S., Bhattacharyya, D., & Hazra, J. (2017). Effect of Swarna Jibanti (Coelogyne cristata Lindley) in alleviation of chronic fatigue syndrome in aged Wistar rats. *Journal of Ayurveda and integrative medicine*.

Miyamoto, K., Kishi, N., & Koshiura, R. (1987). Antitumor effect of agrimoniin, a tannin of *Agrimonia pilosa* Ledeb., on transplantable rodent tumors. *The Japanese Journal of Pharmacology*, *43*(2), 187-195.

Mizutani, K., Ohtani, K., Wei, J. X., Kasai, R., & Tanaka, O. (1984). Saponins from *Anemone rivularis*. *Planta medica*, *50*(4), 327-331.

Mohan, L., & Melkani, A. B. (2016). Therapeutic Impact of Volatiles from *Agrimonia aitchisonii* Schnobeck Temesy and *Pimpinella acuminata* (Edgew.) CB Clarke. *Journal of*, *3*(11), 1062-1066.

Mohanapriya, S., & Vijaiyansiva, G. (2013). Phytochemical Analysis and Antioxidant Potential of *Delphinium denudatum* Wall. *Journal of Modern Biotechnology*, *2*(3), 53-58.

Mohdaly, A. A., Smetanska, I., Ramadan, M. F., Sarhan, M. A., & Mahmoud, A. (2011). Antioxidant potential of sesame (*Sesamum indicum*) cake extract in stabilization of sunflower and soybean oils. *Industrial Crops and Products*, *34*(1), 952-959.

Momin, M. A. M., Bellah, S. F., Rahman, S. M. R., Rahman, A. A., Murshid, G. M. M., & Emran, T. B. (2014). Phytopharmacological evaluation of ethanol extract of *Sida cordifolia* L. roots. *Asian Pacific journal of tropical biomedicine*, *4*(1), 18-24.

Mondal, H., Saha, S., Awang, K., Hossain, H., Ablat, A., Islam, M. K., Jahan, I. A., Sadhu, S. K., Hossain, M. G., Shilpi, J. A. & Uddin, S. J. (2014). Central stimulating and analgesic activity of ethnolic extract of *Alternanthera sessilis* in mice. *BMC complementary and Alternative Medicine*, *14*(1), 398.

Moreira, M. D., Picanco, M. C., Barbosa, L. C. A., Guedes, R. N. C., Barros, E. C., & Campos, M. R. (2007). Compounds from *Ageratum conyzoides*: isolation, structural elucidation and insecticidal activity. *Pest management science*, *63*(6), 615-621.

Morita, M., Shitan, N., Sawada, K., Van Montagu, M. C., Inzé, D., Rischer, H., Goossens, A., Oksman-Caldentey, K.M., Moriyama, Y. , & Yazaki, K. (2009). Vacuolar transport of nicotine is mediated by a multidrug and toxic compound extrusion (MATE) transporter in *Nicotiana tabacum*. *Proceedings of the National Academy of Sciences*, *106*(7), 2447-2452.

Moronkola, D. O., Aboaba, S. A. & Choudhary, I. M. (2015). Composition of volatile oils from leaf, stem, root, fruit and flower of *Ruellia tuberose* L. (Acanthaceae) from Nigeria. *Journal of Medicinal Plants Research*, *8*(41), 1031-1037.

Mors, W. B., do Nascimento, M. C., Parente, J., da Silva, M. H., Melo, P. A., & Suarez-Kurtz, G. (1989). Neutralization of lethal and myotoxic activities of South American rattlesnake venom by extracts and constituents of the plant *Eclipta prostrata* (Asteraceae). *Toxicon*, *27*(9), 1003-1009.

Motamedi, H., Darabpour, E., Gholipour, M., & Seyyednejad, S. M. (2010). Antibacterial effect of ethanolic and methanolic extracts of *Plantago ovata* and *Oliveria decumbens* endemic in Iran against some pathogenic bacteria. *Int J Pharmacol*, *6*(2), 117-122.

Moteriya, P., Satasiya, R., & Chanda, S. (2015). Screening of phytochemical constituents in some ornamental flowers of Saurashtra region. *Journal of Pharmacognosy and Phytochemistry*, *3*(5), 112-120.

Motooka, R., Usami, A., Nakahashi, H., Koutari, S., Nakaya, S., Shimizu, R., Tsuji, K., Marumoto, S., & Miyazawa, M. (2015). Characteristic odor components of essential oils from Eurya japonica. *Journal of oleo science*, *64*(5), 577-584.

Mpiana, P. T., Mudogo, V., Nyamangombe, L., Kakule, M. K., Ngbolua, K. N., Atibu, E. K., ... & Ntumba, J. N. (2009). Antisickling activity and photodegradation effect of anthocyanins extracts from *Alchornea cordifolia* (SCHUMACH & Thonn.) and *Crotalaria retusa* L. *Ann. Afr. Med*, *2*(4), 240-245.

Muhammad, N., Saeed, M., & Khan, H. (2012). Antipyretic, analgesic and anti-inflammatory activity of *Viola betonicifolia* whole plant. *BMC complementary and alternative medicine*, *12*(1), 59.

Muhammad, N., Saeed, M., Aleem, A., & Khan, H. (2012). Ethnomedicinal, phytochemical and pharmacological profile of genus *Viola*. *Phytopharmacol*, *3*(1), 214-226.

Mukherjee, P., Kumar, V., Mal, M., & Houghton, P. (2007). In vitro acetylcholinesterase inhibitory activity of the essential oil from *Acorus calamus* and its main constituents. *Planta medica*, *73*(3), 283-285.

Murthy, K. S. R., Lakshmi, N., & Raghu Ramulu, D. (2011). Biological activity and phytochemical screening of the oleoresin of *Shorea robusta* Gaertn. f. *Tropical and subtropical Agroecosystems*, *14*(3), 787 - 791.

Naeem, M., Khan, M. M. A., Idrees, M., & Aftab, T. (2011). Triacontanol-mediated regulation of growth and other physiological attributes, active constituents and yield of *Mentha arvensis* L. *Plant growth regulation*, *65*(1), 195-206.

Nagatsu, A., Zhang, H., Mizukami, H., Okuyama, H., Sakakibara, J., Tokuda, H., & Nishino, H. (2000). Tyrosinase inhibitory and anti-tumor promoting activities of compounds isolated from safflower (*Carthamus tinctorius* L.) and cotton (*Gossypium hirsutum* L.) oil cakes. *Natural Product Letters*, *14*(3), 153-158.

Nagesh, K. S., & Shanthamma, C. (2009). Antibacterial activity of *Curculigo orchioides* rhizome extract on pathogenic bacteria. *African Journal of Microbiology Research*, *3*(1):5-9.

Nair, V. D., Panneerselvam, R., Gopi, R., & Hong-Bo, S. (2013). Elicitation of pharmacologically active phenolic compounds from *Rauvolfia serpentina* Benth. Ex. Kurtz. *Industrial crops and products*, *45*, 406-415.

Najafi, S. (2013). Phytochemical screening and antibacterial activity of leaf extract of *Ziziphus mauritiana* Lam. *International Research Journal of Applied and Basic Sciences*, *4*(10), 3274-3276.

Najmus-Saqib, Q., Alam, F., & Ahmad, M. (2009). Antimicrobial and cytotoxicity activities of the medicinal plant *Primula macrophylla*. *Journal of enzyme inhibition and medicinal chemistry*, *24*(3), 697-701.

Naman, C. B., Gupta, G., Varikuti, S., Chai, H., Doskotch, R. W., Satoskar, A. R., & Kinghorn, A. D. (2015). Northalrugosidine is a bisbenzyltetrahydroisoquinoline alkaloid from *Thalictrum alpinum* with in vivo antileishmanial activity. *Journal of natural products*, *78*(3), 552-556.

Namjooyan, F., Azemi, M. E., Mosaddegh, M., Cheraghali, A., Kobarfard, F., & Porzel, A. (2007). Screening of some Solanaceae plants for cytotoxic activity, and isolation and structure elucidation of a new steroid from the active fraction of *Physalis divarivata* D. Don. *Planta Medica*, *73*(09):422.

Namjoyan, F., Jahangiri, A., Azemi, M. E., & Mousavi, H. (2016). Inhibitory Effects of *Urginea maritima* (L.) Baker, *Zhumeria majdae* Rech. F. and Wendelbo and *Physalis divaricata* D. Don Ethanolic Extracts on Mushroom Tyrosinase. *Pharmaceutical Sciences*, *22*, 81-86.

Narendhirakannan, R. T., Kandaswamy, M., & Subramanian, S. (2005). Anti-inflammatory activity of *Cleome gynandra* L. on hematological and cellular constituents in adjuvant-induced arthritic rats. *Journal of medicinal food*, *8*(1), 93-99.

Naser, B., Bodinet, C., Tegtmeier, M., & Lindequist, U. (2005). *Thuja occidentalis* (Arbor vitae): a review of its pharmaceutical, pharmacological and clinical properties. *Evidence-based complementary and alternative medicine*, *2*(1), 69-78.

Nath, R., Roy, S., De, B., & Choudhury, M. D. (2013). Anticancer and antioxidant activity of *croton*: a review. *Int J Pharm Pharm Sci*, *5*(2), 63-70.

Nayak, B. S., Anderson, M., & Pereira, L. P. (2007). Evaluation of wound-healing potential of *Catharanthus roseus* leaf extract in rats. *Fitoterapia*, *78*(7-8), 540-544.

Nayak, N., Rath, S., Mishra, M. P., Ghosh, G., & Padhy, R. N. (2013). Antibacterial activity of the terrestrial fern *Lygodium flexuosum* (L.) Sw. against multidrug resistant enteric-and uro-pathogenic bacteria. *Journal of Acute Disease*, *2*(4), 270-276.

Naz, S., Farooq, U., Khan, A., Khan, H., Karim, N., Sarwar, R., Hussain, J. & Rauf, A. (2017). Antidepressent Effect of Two New Benzyl Derivatives from Wild Strawberry *Fragaria vesca* var. *nubicola* Lindl. ex Hook. f. *Frontiers in pharmacology*, *8*, 469.

Ndhlala, A. R., Kasiyamhuru, A., Mupure, C., Chitindingu, K., Benhura, M. A., & Muchuweti, M. (2007). Phenolic composition of *Flacourtia indica, Opuntia megacantha* and *Sclerocarya birrea*. *Food Chemistry*, *103*(1), 82-87.

Negi, J. S., Singh, P., & Rawat, B. (2011). Chemical constituents and biological importance of Swertia: a review. *Curr Res Chem*, *3*(1), 1-15.

Negi, J. S., Singh, P., Joshi, G. P., Rawat, M. S., & Bisht, V. K. (2010). Chemical constituents of *Asparagus*. *Pharmacognosy reviews*, *4*(8), 215.

Nenaah, G. 2013. Antimicrobial activity of *Calotropis procera* Ait.(Asclepiadaceae) and isolation of four flavonoid glycosides as the active constituents. *World Journal of Microbiology and Biotechnology*, *29*(7), 1255-1262.

Ngueyem, T. A., Brusotti, G., Caccialanza, G., & Finzi, P. V. (2009). The genus *Bridelia:* A phytochemical and ethnopharmacological review. *Journal of ethnopharmacology*, *124*(3), 339-349.

Nile, S. H., & Park, S. W. (2014). HPTLC analysis, antioxidant, anti-inflammatory and antiproliferative activities of *Arisaema tortuosum* tuber extract. *Pharmaceutical biology*, *52*(2), 221-227.

Nisa, S., Bibi, Y., Zia, M., Waheed, A., & Chaudhary, M. F. (2013). Anticancer investigations on *Carissa opaca* and *Toona ciliata* extracts against human breast carcinoma cell line. *Pak. J. Pharm. Sci*, *26*(5), 1009-1012.

Nisar, M., Khan, S. A., & Ali, I. (2013). GC-MS Analysis and Pharmacological Potential of Fixed Oil of *Eluphia dabia*. *Middle-East Journal of Scientific Research*, *14*(3), 375-380.

Nisha, P., Nazar, P. A., & Jayamurthy, P. (2009). A comparative study on antioxidant activities of different varieties of *Solanum melongena*. *Food and chemical toxicology*, *47*(10), 2640-2644.

Nishanthi, M., Mohanambal, E., Narendiran, S., Shankar, M., Aanandhi, M. V., & Vijayakumar, B. (2012). Extraction and preliminary phytochemical investigation on whole plant of *Peperomia tetraphylla* (G. Forst. Hook & Arn). *International Journal of Phytopharmacology*, *3*(2), 173-177.

Nithiyanantham, S., Selvakumar, S., & Siddhuraju, P. (2012). Total phenolic content and antioxidant activity of two different solvent extracts from raw and processed legumes, *Cicer arietinum* L. and *Pisum sativum* L. *Journal of food Composition and Analysis*, *27*(1), 52-60.

Njogu, P. M. (2007). Phytochemical investigation and antimicrobial activity of *Girardinia diversifolia* (Link) FRIIS (Urticaceae) (Doctoral dissertation, University of NAIROBI).

Nugroho, A. E., Andrie, M., Warditiani, N. K., Siswanto, E., promono, S. & Lukitaningsih, E. (2012). Antidiabetic antihiperlipidemic effect of *andrographis paniculata* (Burm. f.) Nees and andrographolide in high-fructose-fat-fed rats. *Indian Journal of Pharmacology*, *44*(3), 377.

Núñez Sellés, A. J., Vélez Castro, H. T., Agüero-Agüero, J., González-González, J., Naddeo, F., De Simone, F., & Rastrelli, L. (2002). Isolation and quantitative analysis of phenolic antioxidants, free sugars, and polyols from mango (*Mangifera indica* L.) stem bark aqueous decoction used in Cuba as a nutritional supplement. *Journal of Agricultural and Food Chemistry*, *50*(4), 762-766.

Nyarko, H. D., Barku, V. Y. & Batma, J. (2012). Antimicrobial examination of *cymbopogon citrates* and *Adiatum capillus-veneris* used in Ghanaian folkloric medicine. *International Journal of Life Science and Pharna Research*, *2*, 115-221.

Nyirimigabo, E., Xu, Y., Li, Y., Wang, Y., Agyemang, K., & Zhang, Y. (2015). A review on phytochemistry, pharmacology and toxicology studies of *Aconitum*. *Journal of Pharmacy and Pharmacology*, *67*(1), 1-19.

Olennikov, D. N., Tankhaeva, L. M., Stolbikova, A. V., & Petrov, E. V. (2011). Phenylpropanoids and polysaccharides from *Plantago depressa* and *P. media* growing in Buryatia. *Chemistry of Natural Compounds*, *47*(2), 165.

Oloyede, O. I. (2005). Chemical profile of unripe pulp of *Carica papaya*. *Pakistan Journal of Nutrition*, *4*(6), 379-381.

Oraon, A., & Sinha, B. N. (2012). Sub chronic toxicity potential of the alcoholic extract of *Biophytum reinwardtii* whole plant. *Journal of Natural Sciences Research*, *2*(6), 94-99.

Orhan, I., Kartal, M., Abu-Asaker, M., Şenol, F. S., Yilmaz, G., & Şener, B. (2009). Free radical scavenging properties and phenolic characterization of some edible plants. *Food Chemistry*, *114*(1), 276-281.

Orhan, I., Kartal, M., Abu-Asaker, M., Şenol, F. S., Yilmaz, G., & Şener, B. (2009). Free radical scavenging properties and phenolic characterization of some edible plants. *Food Chemistry*, *114*(1), 276-281.

Ou, Z. Q., Schmierer, D. M., Rades, T., Larsen, L., & McDowell, A. (2013). Application of an online post‐column derivatization HPLC‐DPPH assay to detect compounds responsible for antioxidant activity in *Sonchus oleraceus* L. leaf extracts. *Journal of Pharmacy and Pharmacology*, *65*(2), 271-279.

Ouerghemmi, S., Sebei, H., Siracusa, L., Ruberto, G., Saija, A., Cimino, F., & Cristani, M. (2016). Comparative study of phenolic composition and antioxidant activity of leaf extracts from three wild Rosa species grown in different Tunisia regions: *Rosa canina* L., *Rosa moschata* Herrm. and *Rosa sempervirens* L. *Industrial Crops and Products*, *94*, 167-177.

Özek, G. (2018). Chemical Diversity and Biological Potential of *Tanacetum praeteritum* subsp. *praeteritum* essential Oils. *Journal of the Turkish Chemical Society, Section A: Chemistry*, *5*(2), 493-510.

Padma, R., Parvathy, N. G., Renjith, V., Kalpana, P. R., & Rahate, P. (2013). Quantitative estimation of tannins, phenols, and antioxidant activity of methanolic extract of *Imperata cylindrica*. *Int J Res Pharm Sci*, *4*(1), 73-77.

Paduch, R., Matysik, G., Wójciak-Kosior, M., Kandefer-Szerszen, M., Skalska-Kaminska, A., Nowak-Kryska, M., & Niedziela, P. (2008). *Lamium Album* Extracts Express Free Radical Scavenging and Cytotoxic Activities. *Polish Journal of Environmental Studies*, *17*(4), 569-580.

Painuli, S.., Rai, N. & Kumar, N. (2015). GC-MS analysis of methanolic extract of leaves of *Rhododendron campanulatum*. *International Journal of Pharmacy and Pharmaceutical Sciences*, *7*(12), 299-303.

Pal, R. S., Kumar, R. A., Agrawal, P. K., & Bhatt, J. C. (2013). Antioxidant capacity and related phytochemicals analysis of methanolic extract of two wild edible fruits from north western Indian Himalaya. *Int J Pharm Bio Sci*, *4*(2), 113-123.

Pan, S. L. (Ed.). (2006). *Bupleurum species: scientific evaluation and clinical applications*. CRC Press.

Pandey, A. K., Mohan, M., Singh, P., & Tripathi, N. N. 2015. Chemical composition, antioxidant and antimicrobial activities of the essential oil of *Nepeta hindostana* (Roth) Haines from India. *Records of Natural Products*, 9(2):224-233.

Pandey, M. M., Rastogi, S., & Rawat, A. K. S. (2007). *Saussurea costus*: botanical, chemical and pharmacological review of an ayurvedic medicinal plant. *Journal of ethnopharmacology*, *110*(3), 379-390.

Pandey, N., & Barve, D. (2011). Phytochemical and pharmacological review on *Annona squamosa* Linn. *International Journal of Research in Pharmaceutical and Biomedical Sciences*, *2*(4), 1404-1412.

Pandey, N., & Tripathi, Y. B. (2010). Antioxidant activity of tuberosin isolated from *Pueraria tuberose* Linn. *Journal of inflammation*, *7*(1), 47.

Panthari, P., Kharkwal, H., Kharkwal, H., & Joshi, D. D. (2012). *Myrica nagi*: A review on active constituents, biological and therapeutic effects. *Int. J. Pharm. Pharm. Sci*, *4*, 38-42.

Parajuli, P., Joshee, N., Rimando, A. M., Mittal, S., & Yadav, A. K. (2009). In vitro antitumor mechanisms of various *Scutellaria* extracts and constituent flavonoids. *Planta medica*, *75*(1), 41-48.

Parekh, J., & Chanda, S. (2007). Antibacterial and phytochemical studies on twelve species of Indian medicinal plants. *African Journal of Biomedical Research*, *10*(2).

Parekh, J., Karathia, N., & Chanda, S. (2006). Evaluation of antibacterial activity and phytochemical analysis of *Bauhinia variegata* L. bark. *African Journal of Biomedical Research*, *9*(1), 53-56.

Parker, A. G., Peraza, G. G., Sena, J., Silva, E. S., Soares, M. C. F., Vaz, M. R. C., Furlong, E. B., & Muccillo-Baisch, A. L. (2007). Antinociceptive effects of the aqueous extract of *Brugmansia suaveolens* flowers in mice. *Biological research for nursing*, *8*(3), 234-239.

Parmar, V. S., Jha, A., Bisht, K. S., Taneja, P., Singh, S. K., Kumar, A., Jain, R., & Olsen, C. E. (1999). Review Article Number 138: Constituents of the yew trees. *Phytochemistry*, *50*(8), 1267-1304.

Parsaeimehr, A., Sargsyan, E., & Javidnia, K. (2010). A comparative study of the antibacterial, antifungal and antioxidant activity and total content of phenolic compounds of cell cultures and wild plants of three endemic species of *Ephedra*. *Molecules*, *15*(3), 1668-1678.

Parveen, N., Khan, N. U., & Singhal, K. C. (2002). In vitro antifilarial potential of the flower and stem extracts of *Leucas cephalotes* on cattle filarial parasite Setaria cervi. *Journal of Natural Remedies*, *2*(2), 155-163.

Pascual, M. E., Carretero, M. E., Slowing, K. V., & Villar, A. (2002). Simplified screening by TLC of plant drugs. *Pharmaceutical biology*, *40*(2), 139-143.

Pattanayak, S., Sharma, P., Dama, G., Nayak, S. S., & Panda, D. P. (2009). Anthelmintic activity of *Cajanus scarabaeoides* (L). *Pharmacology Online*, *1*, 35-39.

Paulke, A., Kremer, C., Wunder, C., Achenbach, J., Djahanschiri, B., Elias, A., Schwed, J.S., Hübner, H., Gmeiner, P., Proschak, E. & Toennes, S. W. (2013). *Argyreia nervosa* (Burm. f.): Receptor profiling of lysergic acid amide and other potential psychedelic LSD-like compounds by computational and binding assay approaches. *Journal of ethnopharmacology*, *148*(2), 492-497.

Paulsen, B. S. (2002). Biologically active polysaccharides as possible lead compounds. *Phytochemistry Reviews*, *1*(3), 379-387.

Pavanandt, K., Webster, H. K., Yongvanitchit, K., Kun‐anake, A., Dechatiwongse, T., Nutakul, W., & Bansiddhi, J. (1989). Schizontocidal activity of *Celastrus paniculatus* Willd. against *Plasmodium falciparum* in vitro. *Phytotherapy Research*, *3*(4), 136-139.

Pekamwar, S. S., Kalyankar, T. M., & Kokate, S. S. (2013). Pharmacological activities of Coccinia grandis. Journal of Applied Pharmaceutical Science, *3*(5), 114-119.

Pereira, C., Barros, L., Carvalho, A. M., & Ferreira, I. C. (2011). Nutritional composition and bioactive properties of commonly consumed wild greens: Potential sources for new trends in modern diets. *Food Research International*, *44*(9), 2634-2640.

Pereira, J. A., Oliveira, I., Sousa, A., Ferreira, I. C., Bento, A., & Estevinho, L. (2008). Bioactive properties and chemical composition of six walnut (*Juglans regia* L.) cultivars. *Food and chemical toxicology*, *46*(6), 2103-2111.

Perumal, P. C., Sophia, D., Raj, C. A., Ragavendran, P., Starlin, T., & Gopalakrishnan, V. K. (2012). In vitro antioxidant activities and HPTLC analysis of ethanolic extract of *Cayratia trifolia* (L.). *Asian Pacific Journal of tropical disease*, *2*, 952-956.

Perva-Uzunalić, A., Škerget, M., Knez, Ž., Weinreich, B., Otto, F., & Grüner, S. (2006). Extraction of active ingredients from green tea (*Camellia sinensis*): Extraction efficiency of major catechins and caffeine. *Food Chemistry*, *96*(4), 597-605.

Pettit, G. R., Hoard, M. S., Doubek, D. L., Schmidt, J. M., Pettit, R. K., Tackett, L. P., & Chapuis, J. C. (1996). Antineoplastic agents 338. The cancer cell growth inhibitory. Constituents of *Terminalia arjuna* (Combretaceae). *Journal of ethnopharmacology*, *53*(2), 57-63.

Pino, J. A., & Quijano, C. E. (2012). Study of the volatile compounds from plum (*Prunus domestica* L. cv. Horvin) and estimation of their contribution to the fruit aroma. *Food Science and Technology*, *32*(1), 76-83.

Plouvier, V. (1963). Distribution of aliphatic polyols and cyclitols. *Chemical plant taxonomy*, 313-336.

Pokharen, N., Dahal, S., & Anuradha, M. 2011. Phytochemical and antimicrobial studies of leaf extract of *Euphorbia neriifolia*. *Journal of Medicinal Plants Research*, *5*(24), 5785-5788.

Potdar, D., Hirwani, R. R., & Dhulap, S. (2012). Phyto-chemical and pharmacological applications of *Berberis aristata*. *Fitoterapia*, *83*(5), 817-830.

Prabakar, K., & Wembonyama, J. P. (2016). Phytochemical profiling of the aqueous leaf extracts of Blepharis maderaspatensis (L.) Heyne ex Roth its HPLC, GC-MS, and column chromatographic analysis. *Imperial Journal of Interdisciplinary Research*, *2*(7).

Prajapati, R., Kalariya, M., Umbarkar, R., Parmar, S., & Sheth, N. (2011). *Colocasia esculenta*: A potent indigenous plant. *International Journal of Nutrition, Pharmacology, Neurological Diseases*, *1*(2), 90.

Prasad, D. & Sati, S. P. (2010). A new flavanone from *Cyathula Tomentosa*. *Oriental Journal of Chemistry*, *26*(4), 1585-1587.

Prasad, K., Moulekhi, K., & Bisht, G. (2011). Chemical composition of the essential oil of *Pavetta indica* L. leaves. *Res. J. Phytochem*, *5*(1), 66-69.

Pratchayasakul, W., Pongchaidecha, A., Chattipakorn, N., & Chattipakorn, S. (2008). Ethnobotany & ethnopharmacology of *Tabernaemontana divaricata*. *Indian Journal of Medical Research*, *127*(4), 317.

Price, K.R., Bacon, J.R. & Rhodes, M.J. (1997). Effect of storage and domestic processing on content and composition of flavonol glucosides in onion (*Allium cepa*). *Journal of Agriculture and Food Chemistry*, *45*(3), 938-942.

Priya, K., & Ganjewala, D. (2007). Antibacterial activities and phytochemical analysis of different plant parts of *Nyctanthes arbor-tristis* (Linn.). *Research Journal of Phytochemistry*, *1*(2), 61-67.

Priya, K., Gupta, A., Mahajan, S., Agnihotri, R. K., & Sharma, R. (2015). Evaluation of antimicrobial properties of *Basella rubra* methanolic extracts on selected microorganisms. *Int J Pharm Sci Res*, *6*, 334-6.

Pu, Z., Yin, Z., Jia, R., Song, X., Xu, J., Wang, X., Chen, X. , & Luo, M. (2014). Preliminary Isolation and Antibacterial Activity of the Ethyl Acetate Extract of *Prinsepia utilis* Royle in Vitro. *Agricultural Sciences*, *5*(6), 540-545.

Purnima, B. M., & Kothiyal, P. (2015). A review article on phytochemistry and pharmacological profiles of *Nardostachys jatamansi* DC-medicinal herb. *Journal of pharmacognosy and phytochemistry*, *3*(5), 102-106.

Purohit, M. C., Singh, M., Purohit, R., Kumar, G., & Garhwal, S. (2017). Antimicrobial activity of methanolic extract of bark of *Prunus cornuta*. International Journal of Pharmaceutical Sciences and Research, *8*(8), 3563-3567.

Qin, G. W. (1998). Some progress on chemical studies of triterpenoid saponins from Chinese medicinal plants. *Current Organic Chemistry*, *2*, 613-625.

Queiroz, S. C., Cantrell, C. L., Duke, S. O., Wedge, D. E., Nandula, V. K., Moraes, R. M., & Cerdeira, A. L. (2012). Bioassay-directed isolation and identification of phytotoxic and fungitoxic acetylenes from *Conyza canadensis*. *Journal of agricultural and food chemistry*, *60*(23), 5893-5898.

Quettier-Deleu, C., Gressier, B., Vasseur, J., Dine, T., Brunet, C., Luyckx, M., Cazin, M., Cazin, J.C., Bailleul, F., & Trotin, F. (2000). Phenolic compounds and antioxidant activities of buckwheat (*Fagopyrum esculentum* Moench) hulls and flour. *Journal of ethnopharmacology*, *72*(1-2), 35-42.

Ragasa, C. Y., Lorena, G. S., Mandia, E. H., Raga, D. D., & Shen, C. C. (2013). Chemical constituents of *Abrus precatorius*. *Amer J Essent Oils Nat Prod*, *1*(2), 7-10.

Ragasa, C. Y., Wong, J., & Rideout, J. A. (2007). Monoterpene glycoside and flavonoids from *Blumea lacera*. *Journal of Natural Medicines*, *61*(4), 474-475.

Raghavendra, H. L., Prashith, K. T., Valleesha, N. C., Sudharshan, S. J., & Chinmaya, A. (2010). Screening for cytotoxic activity of methanol extract of *Putranjiva roxburghii* Wall (Euphorbiaceae) seeds. *Pharmacognosy Journal*, *2*(10), 335-337.

Raghavendra, M. P., Satish, S., & Raveesha, K. A. (2006). Phytochemical analysis and antibacterial activity of *Oxalis corniculata*; a known medicinal plant. *Myscience*, *1*(1), 72-78.

Rahman, N., Ahmad, M., Riaz, M., Mehjabeen, J. N., & Ahmad, R. (2013). Phytochemical, antimicrobial, insecticidal and brine shrimp lethality bioassay of the crude methanolic extract of Ajuga parviflora Benth. *Pak. J. Pharm. Sci*, *26*(4), 751-756.

Rahman, S., Ismail, M., Shah, M. R., Iriti, M., & Shahid, M. (2015). GC/MS analysis, free radical scavenging, anticancer and β-glucuronidase inhibitory activities of *Trillium govanianum* rhizome. *Bangladesh Journal of Pharmacology*, *10*(3), 577-583.

Rahmatullah, M., Hossain, M., Mahmud, A., Sultana, N., Mizanur, S., Mohammad, R., Islam, R., Khatoon, M.S., Jahan, S & Islam, F. (2013). Antihyperglycemic and antinociceptive activity evaluation of ‘khoyer’prepared from boiling the wood of *Acacia catechu* in water. *African Journal of Traditional, Complementary and Alternative Medicines*, *10*(4), 1-5.

Rajan, S., Gokila, M., Jency, P., Brindha, P., & Sujatha, R. K. (2011). Antioxidant and phytochemical properties of *Aegle marmelos* fruit pulp. *International journal of current pharmaceutical research*, *3*(2), 65-70.

Rajasekaran, A., Sivakumar, V., & Darlinquine, S. (2012). Evaluation of wound healing activity of *Ammannia baccifera* and *Blepharis maderaspatensis* leaf extracts on rats. *Revista Brasileira de Farmacognosia*, *22*(2), 418-427.

Rajendran, M. P., Pallaiyan, B. B., & Selvaraj, N. (2014). Chemical composition, antibacterial and antioxidant profile of essential oil from *Murraya koenigii* (L.) leaves. *Avicenna journal of phytomedicine*, *4*(3), 200-214.

Rajendran, N., Subramaniam, S., Lotha, R., Pemaiah, B., & Sivasubramanian, A. (2016). Isolation and Characterization of flavonoids and flavone glycosides from the ethnic traditional medicinal plant *Cotoneaster bacillaris* Wall. Ex Lindl. *Der. Pharm. Lett.*, *8*(5), 321-324.

Rajkumar, S., & Jebanesan, A. (2007). Repellent activity of selected plant essential oils against the malarial fever mosquito *Anopheles stephensi*. *Trop Biomed*, *24*(2), 71-75.

Rajkumar, T., & Sinha, B. N. (2010). Chromatographic finger print analysis of budmunchiamines in *Albizia amara* by HPTLC technique. *Int J Res Pharm Sci*, *1*(3), 313-316.

Raju, S., Subbaiah, N. V., Reddy, K. S., Das, A., & Murugan, K. B. (2011). Potential of *Pandanus odoratissimus* as a CNS depressant in Swiss albino mice. *Brazilian Journal of Pharmaceutical Sciences*, *47*(3), 629-634.

Ramamoorthy, P. K. T., LakshmanaShetty, R. H., Devidas, S., Mudduraj, V. T., & Vinayaka, K. S. (2004). Antifungal and cytotoxic activity of *Everniastrum cirrhatum* (Fr.) Hale. *Chiang Mai. J. Sci*, *39*(1), 76-83.

Ramesh, C. K., Rehman, A., Prabhakar, B. T., Avin, B. R., & Rao, S. J. (2011). Antioxidant potentials in sprouts vs. seeds of *Vigna radiata* and *Macrotyloma uniflorum*. *Journal of Applied Pharmaceutical Science*, *1*(7), 99-103.

Rana, F., & Avijit, M. (2012). Review on *Butea monosperma*. *Int. J. of Research in Pharmacy and Chemistry*, *2*, 1035-1039.

Rana, I. S., Rana, A. S., & Rajak, R. C. (2011). Evaluation of antifungal activity in essential oil of the *Syzygium aromaticum* (L.) by extraction, purification and analysis of its main component eugenol. *Brazilian Journal of Microbiology*, *42*(4), 1269-1277.

Rani, D., Khare, P. B., & Dantu, P. K. (2010). In vitro antibacterial and antifungal properties of aqueous and non-aqueous frond extracts of *Psilotum nudum, Nephrolepis biserrata* and *Nephrolepis cordifolia*. *Indian journal of pharmaceutical sciences*, *72*(6), 818-822.

Ranpal, S. (2009). An Assessment of Status and Antibacterial Properties of *Dactylorhiza hatagirea* in Annapurna Conservation Area (A case study of Paplekharka, Lete VDC, Mustang). B. Sc. Forestry Research Thesis Submitted to Tribhuvan University, Institute of Forestry, Pokhara, Nepal. *I Tribhuvan University Institute of Forestry*, 3.

Rao, Y. K., Lien, H. M., Lin, Y. H., Hsu, Y. M., Yeh, C. T., Chen, C. C., Lai, C. H., & Tzeng, Y. M. (2012). Antibacterial activities of *Anisomeles indica* constituents and their inhibition effect on Helicobacter pylori-induced inflammation in human gastric epithelial cells. *Food Chemistry*, *132*(2), 780-787.

Rasheed, A. N., Afifi, F. U., Shaedah, M. & Taha, M. O. (2004). Investigation of the active constituents of *Portulaca oleraceae* L.(Portulacaceae) growing in Jordan. *Pakistan Journal of Pharmaceutical Sciences*, *17*(1), 37-45.

Rashid, S., Kaloo, Z. A., Singh, S., & Bashir, I. (2014). Callus induction and shoot regeneration from rhizome explants of *Rheum webbianum* Royle-a threatened medicinal plant growing in Kashmir Himalaya. *Journal of Scientific and Innovative Research*, *3*(5), 515-518.

Rashmi, S., & Rajkumar, H. G. (2014). Preliminary phytochemical screening of different solvent extracts of lichens from Kodagu district, Karnataka. *Journal of Pharmacognosy and Phytochemistry*, *3*(4): 209-212.

Rattan, R., Kumari, A., Gautam, V., Fozdar, B. I., Sharma, U. & Kumar, D. (2016). Preliminary phytochemical screening, anoxidant, and antifungal activity of *Lepigathis cuspidate*. *International Journal of Drug Development and Research*, *8*(2).

Ravn, H. W., Mondolot, L., Kelly, M. T., & Lykke, A. M. (2015). Plantamajoside—A current review. *Phytochemistry Letters*, *12*, 42-53.

Rawat, J. M., Rawat, B., Chandra, A., & Nautiyal, S. (2013). Influence of plant growth regulators on indirect shoot organogenesis and secondary metabolite production in Aconitum violaceum Jacq. *African Journal of Biotechnology*, *12*(44), 6287-6293.

Raziq, N., Saeed, M., Ali, M. S., Zafar, S., & Ali, M. I. (2015). In vitro anti-oxidant potential of new metabolites from *Hypericum oblongifolium* (Guttiferae). *Natural product research*, *29*(24), 2265-2270.

Reddy, M. N., & Mishra, G. J. (2012). Preliminary phytochemical screening and antibacterial analysis of the leaf extracts of *Launaea procumbens* Roxb. *International Journal of Phytopharmacology*, *3*(2), 147-151.

Reddy, S. V., Srinivas, P. V., Praveen, B., Kishore, K. H., Raju, B. C., Murthy, U. S., & Rao, J. M. (2004). Antibacterial constituents from the berries of *Piper nigrum*. *Phytomedicine*, *11*(7-8), 697-700.

Reddy, U. D. C., Chawla, A. S., Deepak, M., Singh, D., & Handa, S. S. (1999). High pressure liquid chromatographic determination of bergenin and (+)-afzelechin from different parts of Paashaanbhed (*Bergenia ligulata* Yeo). *Phytochemical Analysis*, *10*(1), 44-47.

Regnault-Roger, C., & Hamraoui, A. (1995). Fumigant toxic activity and reproductive inhibition induced by monoterpenes on *Acanthoscelides obtectus* (Say)(Coleoptera), a bruchid of kidney bean (*Phaseolus vulgaris* L.). *Journal of Stored Products Research*, *31*(4), 291-299.

Ren, Z. Y., Zhang, Y., & Shi, Y. P. (2009). Simultaneous determination of nine flavonoids in *Anaphalis margaritacea* by capillary zone electrophoresis. *Talanta*, *78*(3), 959-963.

Rios, J. L., & Waterman, P. G. (1997). A review of the pharmacology and toxicology of *Astragalus*. *Phytotherapy Research*, *11*(6), 411-418.

Rivera‐Pastrana, D. M., Yahia, E. M., & González‐Aguilar, G. A. (2010). Phenolic and carotenoid profiles of papaya fruit (*Carica papaya* L.) and their contents under low temperature storage. *Journal of the Science of Food and Agriculture*, *90*(14), 2358-2365.

Robinson, G. M., & Robinson, R. (1931). A survey of anthocyanins. I. *Biochemical Journal*, *25*(5):1687-1705.

Rocha-Guzmán, N. E., Herzog, A., González-Laredo, R. F., Ibarra-Pérez, F. J., Zambrano-Galván, G., & Gallegos-Infante, J. A. (2007). Antioxidant and antimutagenic activity of phenolic compounds in three different colour groups of common bean cultivars (*Phaseolus vulgaris*). *Food Chemistry*, *103*(2), 521-527.

Roeder, E., & Wiedenfeld, H. (2009). Pyrrolizidine alkaloids in medicinal plants of Mongolia, Nepal and Tibet. *Die Pharmazie-An International Journal of Pharmaceutical Sciences*, *64*(11), 699-716.

Roeder, E., & Wiedenfeld, H. (2013). Plants containing pyrrolizidine alkaloids used in the Traditional Indian Medicine–including Ayurveda. *Die Pharmazie-An International Journal of Pharmaceutical Sciences*, *68*(2), 83-92.

Rokaya, M. B., Maršík, P., & Münzbergová, Z. (2012). Active constituents in *Rheum acuminatum* and *Rheum australe* (Polygonaceae) roots: A variation between cultivated and naturally growing plants. *Biochemical systematics and ecology*, *41*, 83-90.

Rupasinghe, H. V., Jackson, C. J. C., Poysa, V., Di Berardo, C., Bewley, J. D., & Jenkinson, J. (2003). Soyasapogenol A and B distribution in soybean (*Glycine max* L. Merr.) in relation to seed physiology, genetic variability, and growing location. *Journal of agricultural and food chemistry*, *51*(20), 5888-5894.

Sadilova, E., Stintzing, F. C., & Carle, R. (2006). Anthocyanins, colour and antioxidant properties of eggplant (*Solanum melongena* L.) and violet pepper (*Capsicum annuum* L.) peel extracts. *Zeitschrift für Naturforschung C*, *61*(7-8), 527-535.

Safeer, S., Qureshi, R., ul Hassan, U., Khalil, S., & Anwar, F. (2017). Ethnobotanical study on useful indigenous plants in Mahasheer National Park, AJK. *Journal of Coastal Life Medicine. Journal of Coastal Life Medicine*, *5*(3), 109-115.

Saha, J., Mitra, T., Gupta, K. & Mukherjee, S. (2012). Phytoconstituents and HPTLC analysis in *Saraca asoca* (Roxb.) Wilde. *International Journal of Pharmacy and Pharmaceutical Sciences*, *4*(1), 96-99.

Saha, S., Walia, S., Kumar, J., Dhingra, S. & Parmar, B. S. (2010).Screening for Feeding Deterrent and Insect Growth Regulatory Activity of Triterpenic Saponins from *Diploknema butyracea* and *Sapindus mukorossi*J. Agric. Food Chem., 58(1), 434–440.

Sahreen, S., Khan, M. R., & Khan, R. A. (2010). Evaluation of antioxidant activities of various solvent extracts of *Carissa opaca* fruits. *Food chemistry*, *122*(4), 1205-1211.

Sahreen, S., Khan, M. R., & Khan, R. A. (2011). Phenolic compounds and antioxidant activities of *Rumex hastatus* D. Don. Leaves. *Journal of Medicinal Plants Research*, *5*(13), 2755-2765.

Sahu, R., Itankar, P., Mishra, M., Maliye, A., & Sonekar, M. (2016). Phytochemical screening and HPTLC finger printing analysis of *Roscea procera*(kakoli) and *Lilium polyphyllum* (kshirkakoli). *International Journal of Pharmacy and Pharmaceutical Research*, *8*(1), 53-69.

Sahu, V. K., Irchhaiya, R., Shashi, A., & Gurjar, H. (2010). Phytochemical investigation and chromatographic evaluation of the ethanolic extract of whole plant extract of *Dendrophthoe falcata* (LF) Ettingsh. *International Journal of Pharmaceutical Sciences and Research*, *1*(1), 39-45.

Sajewicz, M., Rzepa, J., Hajnos, M., Wojtal, Ł., Staszek, D., Kowalska, T., & Waksmundzka-Hajnos, M. (2009). GC-MS study of the performance of different techniques for isolating the volatile fraction from sage (*Salvia* L.) species, and comparison of seasonal differences in the composition of this fraction. *Acta Chromatographica*, *21*(3), 453-471.

Saklani, S., & Chandra, S. (2011). Antimicrobial activity, nutritional profile and quantitative study of different fractions of *Ficus palmata*. *Intern. Res. J. Plant Sci*, *2*, 332-337.

Saklani, S., Mishra, A. P., Parcha, V., & Chandra, S. (2011). Phytochemical and antibacterial evaluation of *Satyrium nepalense* and *Saussurea simpsoniana*, the threatened medicinal herbs of Uttarakhand. *J Pharm Res*, *4*, 3866-3870.

Saleem, A., Husheem, M., Härkönen, P., & Pihlaja, K. (2002). Inhibition of cancer cell growth by crude extract and the phenolics of *Terminalia chebula* retz. fruit. *Journal of Ethnopharmacology*, *81*(3), 327-336.

Saleem, R., Ahmad, S. I., Ahmed, M., Faizi, Z., Zikr-ur-Rehman, S., Ali, M., & Faizi, S. (2003). Hypotensive activity and toxicology of constituents from *Bombax ceiba* stem bark. *Biological and Pharmaceutical Bulletin*, *26*(1), 41-46.

Salem, A. F. Z., Salem, M. Z., González-Ronquillo, M., Camacho, L. M., & Cipriano, M. (2011). Major chemical constituents of Leucaena leucocephala and Salix babylonica leaf extracts. *Journal of Tropical Agriculture*, *49*, 95-98.

Salman, S. M., Khan, S. B., Ali, S., e Shahwar, D., Siddique, M., Afridi, Z. K., & Lutfullah, G. (2015). *Himalrandia tetrasperma*, ethanolic extracts preliminary phytochemical analysis, antibacterial and antifungal activities.*Journal of Biodiversity and Environmental Sciences*, *7*(3), 100-109.

Samuelsen, A. B. (2000). The traditional uses, chemical constituents and biological activities of Plantago major L. A review. *Journal of ethnopharmacology*, *71*(1-2), 1-21.

Samyal, M. L., Ahuja, A., & Ahmed, Z. (2014). Evaluation of Antidiabetic Activity of Isolated Compound from *Ougeinia oojeinensis* bark extract in diabetic rats. *UK Journal of Pharmaceutical and Biosciences*, *2*(5), 27-33.

Sandhya, S., Sravanthi, E. V., & Vinod, K. R. (2013). Evaluation of a dermatological herbal hydrogel integrated with *Ipomea pes-tigridis* for anti acne activity. *inflammation*, *1*(5), 1778-1784.

Sandoval-Montemayor, N. E., García, A., Elizondo-Treviño, E., Garza-González, E., Alvarez, L., & del Rayo Camacho-Corona, M. (2012). Chemical composition of hexane extract of *Citrus aurantifolia* and anti-Mycobacterium tuberculosis activity of some of its constituents. *Molecules*, *17*(9), 11173-11184.

Sanghai, D. B., Kumar, S. V., Srinivasan, K. K., Aswatharam, H. N., & Shreedhara, C. S. (2013). Pharmacognostic and phytochemical investigation of the leaves of *Malvastrum coromandelianum* (L.) Garcke. *Ancient science of life*, *33*(1), 39-44.

Santosh, P., Venugopl, R., Nilakash, A. S., Kunjbihari, S., & Mangala, L. (2011). Antidepressant activity of methanolic extract of *Passiflora foetida* leaves in mice. *Int J Pharm Pharm Sci*, *3*(1), 112-115.

Saraf, A. (2010). Phytochemical and antimicrobial studies of medicinal plant *Costus speciosus* (Koen.). *Journal of Chemistry*, *7*, 405-413.

Saraf, A. (2010). Phytochemical and antimicrobial studies of medicinal plant *Costus speciosus* (Koen.). *Journal of Chemistry*, *7*(1), 405-413.

Sarker, S. D., & Nahar, L. (2004). Natural medicine: the genus Angelica. *Current medicinal chemistry*, *11*(11), 1479-1500.

Sati, S. C., Sati, N., & Sati, O. P. (2011). Bioactive constituents and medicinal importance of genus *Alnus*. *Pharmacognosy reviews*, *5*(10), 174.

Sautour, M., Mitaine-Offer, A. C., & Lacaille-Dubois, M. A. (2007). The *Dioscorea* genus: a review of bioactive steroid saponins. *Journal of natural medicines*, *61*(2), 91-101.

Savithramma, N., Rao, M. L., & Suhrulatha, D. (2011). Screening of medicinal plants for secondary metabolites. *Middle-East Journal of Scientific Research*, *8*(3), 579-584.

Savithramma, N., Rao, M. L., & Suhrulatha, D. (2011). Screening of medicinal plants for secondary metabolites. *Middle-East Journal of Scientific Research*, *8*(3), 579-584.

Schepetkin, I. A., Kirpotina, L. N., Jakiw, L., Khlebnikov, A. I., Blaskovich, C. L., Jutila, M. A., & Quinn, M. T. (2009). Immunomodulatory activity of oenothein B isolated from *Epilobium angustifolium*. *The Journal of Immunology*, *183*(10), 6754-6766.

Seal, T. (2016). Quantitative HPLC analysis of phenolic acids, flavonoids and ascorbic acid in four different solvent extracts of two wild edible leaves, *Sonchus arvensis* and *Oenanthe linearis* of North-Eastern region in India. *Journal of Applied Pharmaceutical Science*, *6*(2), 157-166.

Seal, T., & Chaudhuri, K. (2015). Antioxidant activities of five wild edible fruits of meghalaya state in india and effect of solvent extraction system. *International Journal of Pharmaceutical Sciences and Research*, *6*(12), 5134.

Selvam, C. & Jachak, S.M. (2004). A cyclooxygenase (COX) inhibitory biflavonoid from the seeds *of semecarpus anacardium*. *Journal of Ethnopharmacology*, *95*(2-3), 209-212.

Semwal, D. K., Badoni, R., Semwal, R., Kothiyal, S. K., Singh, G. J. P., & Rawat, U. (2010). The genus *Stephania* (Menispermaceae): Chemical and pharmacological perspectives. *Journal of Ethnopharmacology*, *132*(2), 369-383.

Semwal, D. K., Bamola, A., & Rawat, U. (2007). Chemical constituents from some antidiabetic plants. *Univ J Phytochem Ayur Heig*, *2*(3), 40-48.

Semwal, D. K., Bamola, A., & Rawat, U. (2007). Chemical constituents from some antidiabetic plants. *Univ J Phytochem Ayur Heig*, *2*(3), 40-48.

Semwal, D. K., Rawat, U., Bamola, A., & Semwal, R. (2009). Antimicrobial activity of *Phoebe lanceolata* and *Stephania glabra*; preliminary screening studies. *Journal of scientific research*, *1*(3), 662-666.

Semwal, D. K., Rawat, U., Bamola, A., & Semwal, R. (2009). Antimicrobial activity of *Phoebe lanceolata* and *Stephania glabra*; preliminary screening studies. *Journal of scientific research*, *1*(3), 662-666.

Semwal, P., Anthwal, P., Kapoor, T., & Thapliyal, A. (2014). Preliminary investigation of phytochemicals of *Saussurea obvallata* (brahm kamal) and *Pittosporum eriocarpum* (agni): two endangered medicinal plant species of Uttarakhand. *International Journal of Pharmacognosy*, *1*(4), 266-269.

Semwal, R. B., Semwal, D. K., Semwal, R., Singh, R., & Rawat, M. S. M. (2011). Chemical constituents from the stem bark of *Symplocos paniculata* Thunb. with antimicrobial, analgesic and anti-inflammatory activities. *Journal of ethnopharmacology*, *135*(1), 78-87.

Senatore, F., De Fusco, R., & Napolitano, F. (2001). *Eupatorium cannabinum* L. ssp. *cannabinum* (Asteraceae) essential oil: chemical composition and antibacterial activity. *Journal of Essential Oil Research*, *13*(6), 463-466.

Seyyednejad, S. M., Koochak, H., Darabpour, E., & Motamedi, H. (2010). A survey on *Hibiscus rosa—sinensis, Alcea rosea* L. and *Malva neglecta* Wallr as antibacterial agents. *Asian Pacific Journal of Tropical Medicine*, *3*(5), 351-355.

Shabir, M., Agnihotri, P., Husain, D., Tiwari, J. K., & Husain, T. (2017). On the current status of the genus Gentiana L.(Gentianaceae) in India.*Pleione 11*(1), 16-24.

Shadia, E., El-Aziz, A., Omer, E. A., & Sabra, A. S. (2007). Chemical composition of *Ocimum americanum* essential oil and its biological effects against*, Agrotis ipsilon*,(Lepidoptera: Noctuidae). *Research Journal of Agriculture and Biological Sciences*, *3*(6), 740-747.

Shafeeq, M. H., Omar-Zahid, L. A., & Sidkey, B. A. (2014). The antimicrobial activity of *Carissa carandas* L., *Ficus carica* L., and *Olea europaeae* L. leaves extracts on growth of some pathogenic microorganisms. *Journal of AlNahrain University*, *71*(4), 744-753.

Shah, N. A., Khan, M. R., & Nadhman, A. (2014). Antileishmanial, toxicity, and phytochemical evaluation of medicinal plants collected from Pakistan. *BioMed research international*, 2014.

Shah, N. A., Khan, M. R., Naz, K., & Khan, M. A. (2014). Antioxidant potential, DNA protection, and HPLC-DAD analysis of neglected medicinal *Jurinea dolomiaea* roots. *BioMed research international*.

Shah, Z., Ali, F., Ullah, H., Khan, D., Khan, S., Khan, R., & Ali, I. (2014). Biological Screening and Chemical Constituents of *Viburnum grandiflorum*. *J Chem Soc Pak*, *36*, 113-118.

Shahwar, D., Raza, M. A., Saeed, A., Riasat, M., Chattha, F. I., Javaid, M., & Ullah, S. (2012). Antioxidant potential of the extracts of *Putranjiva roxburghii, Conyza bonariensis, Woodfordia fruiticosa* and *Senecio chrysanthemoids*. *African Journal of Biotechnology*, *11*(18), 4288-4295.

Sharanabasappa, G. K., Santosh, M. K., Shaila, D., Seetharam, Y. N., & Sanjeevarao, I. (2007). Phytochemical Studies on *Bauhinia racemosa* Lam. *Bauhinia purpurea* Linn. and Hardwickia binata Roxb. *Journal of Chemistry*, *4*(1), 21-31.

Sharma, A. K., Dutt, D., Upadhyaya, J. S., & Roy, T. K. (2011). Anatomical, morphological, and chemical characterization of *Bambusa tulda*, *Dendrocalamus hamiltonii, Bambusa balcooa, Malocana baccifera, Bambusa arundinace*a and *Eucalyptus tereticornis*. *BioResources*, *6*(4), 5062-5073.

Sharma, A., & Cannoo, D. S. (2013). phytochemical composition of essential oils isolated from different species of genus *Nepeta* of labiatae family: a review. *Pharmacophore*, *4*(6), 181-211.

Sharma, A., & Patel, V. K. (2009). In vitro screening of the antibacterial activity and identification of bioactive compounds from plants against selected Vibrio spp. pathogens. *Turkish Journal of Biology*, *33*(2), 137-144.

Sharma, A., Chandra Sati, S., Prakash Sati, O., Dhobhal Sati, M., & Kumar Kothiyal, S. (2012). Genus *Euonymus*: chemical and pharmacological perception. *Mini-Reviews in Organic Chemistry*, *9*(4), 341-351.

Sharma, A., Khulbe, R., Sood, S., Agrawal, P. K., Bhatt, J. C., & Pattanayak, A. (2017). A Simple Method for the Characterization of Antioxidant Property of Different Extracts of Bark of Gethi (*Boehmeria rugulosa*). *Int. J. Curr. Microbiol. App. Sci*, *6*(4), 2635-2645.

Sharma, A., Sangameswaran, B., Jain, V., & Saluja, M. S. (2012). Hepatoprotective activity of *Adina cordifolia* against ethanol induce hepatotoxicity in rats.

Sharma, V. K., Chauhan, N. S., Lodhi, S., & Singhai, A. K. (2009). Anti-depressant activity of *Zizyphus xylopyrus*. *International journal of phytomedicine*, *1*(1), 12-17.

Sharma, V., Thakur, M., Chauhan, N. S., & Dixit, V. K. (2010). Effects of petroleum ether extract of *Anacyclus pyrethrum* DC. on sexual behavior in male rats. *Zhong Xi Yi Jie He Xue Bao*, *8*(8), 767-73.

Sharma, Y. N., Zaman, A., & Kidwai, A. R. (1964). Chemical examination of *Heracleum candicans*—I: Isolation and structure of a new furocoumarin—heraclenin. *Tetrahedron*, *20*(1), 87-90.

Shen, S., Wang, Q., & Li, Y. B. (2011). Studies on chemical constituents and pharmaceutical activity of *Achyranthes bidentata* Bl. *Strait Pharm J.,23*, 1-6.

Shen, Y. C., & Chen, C. H. (1989). Novel secoiridoid lactones from Jasminum multiflorum. *Journal of natural products*, *52*(5), 1060-1070.

Shilpi, J. A., Taufiq-Ur-Rahman, M., Uddin, S. J., Alam, M. S., Sadhu, S. K., & Seidel, V. (2006). Preliminary pharmacological screening of *Bixa orellana* L. leaves. *Journal of Ethnopharmacology*, *108*(2), 264-271.

Shim, Y. Y., Gui, B., Arnison, P. G., Wang, Y., & Reaney, M. J. (2014). Flaxseed (*Linum usitatissimum* L.) bioactive compounds and peptide nomenclature: a review. *Trends in food science & technology*, *38*(1), 5-20.

Shimoda, T., Nishihara, M., Ozawa, R., Takabayashi, J., & Arimura, G. I. (2012). The effect of genetically enriched (E)‐β‐ocimene and the role of floral scent in the attraction of the predatory mite *Phytoseiulus persimilis* to spider mite‐induced volatile blends of torenia. *New Phytologist*, *193*(4), 1009-1021.

Shrestha, P., Adhikari, S., Lamichhane, B., & Shrestha, B. G. (2015). Phytochemical screening of the medicinal plants of Nepal. *IOSR Journal of Environmental Science, Toxicology and Food Technology*, *1*(6), 11-17.

Shrestha, R. S., Adhikari, A., Marasini, B. P., Jha, R. N., & Choudhary, M. I. (2013). Novel inhibitors of urease from *Corydalis govaniana* Wall. *Phytochemistry Letters*, *6*(2), 228-231.

Shrestha, S., Natarajan, S., Park, J. H., Lee, D. Y., Cho, J. G., Kim, G. S., Jeon, Y. J., Yeon, S. W., Yang, D. C. & Baek, N. I. (2013). Potential neuroprotective flavonoid-based inhibitors of CDK5/p25 from *Rhus parviflora*. *Bioorganic and Medicinal Chemistry letters*, *23*(18), 5150-5154.

Shrestha, S., Park, J. H., Lee, D. Y., Cho, J. G., Cho, S., Yang, H. I., Yoon, M. S., Han, D. S. & Baek, N. I. (2012). *Rhus parviflora* and its biflavonoid constituent, rhusflavon, induce sleep through the positive allosteric modulation of GABAA-benzodiazepine receptors. *Journal of ethnopharmacology*, *142*(1), 213-220.

Shrinivas, S., Lakshmi, K. S., & Rajesh, T. (2009). Evaluation of antidiarrhoeal potentials of ethanolic extract of leaves of *Holoptelea integrifolia* in mice model. *International Journal of Pharmaceutical Research*, *1*(3), 832-836.

Shrivastava, N., & Patel, T. (2007). Clerodendrum and heathcare: an overview. *Medicinal and aromatic plant science and biotechnology*, *1*(1), 142-150.

Shuveksh, P. S., Ahmed, K., Padhye, S., Schobert, R., & Biersack, B. (2017). Chemical and biological aspects of the natural 1, 4-benzoquinone embelin and its (semi-) synthetic derivatives. *Current medicinal chemistry*, *24*(18), 1998-2009.

Siddiq, F., Fatima, I., Malik, A., Afza, N., Iqbal, L., Lateef, M., Hameed, S., & Khan, S. W. (2012). Biologically active bergenin derivatives from *Bergenia stracheyi*. *Chemistry & biodiversity*, *9*(1), 91-98.

Siddiqi, R., Naz, S., Ahmad, S., & Sayeed, S. A. (2011). Antimicrobial activity of the polyphenolic fractions derived from *Grewia asiatica, Eugenia jambolana* and *Carissa carandas*. *International journal of food science & technology*, *46*(2), 250-256.

Siddiqui, J. A., Sharan, K., Swarnkar, G., Rawat, P., Kumar, M., Manickavasagam, L., Maurya, R., Pierroz, D. , & Chattopadhyay, N. (2011). Quercetin-6-C-β-d-glucopyranoside isolated from *Ulmus wallichiana* planchon is more potent than quercetin in inhibiting osteoclastogenesis and mitigating ovariectomy-induced bone loss in rats. *Menopause*, *18*(2), 198-207.

Sidjui, L. S., Zeuko'o, E. M., Toghueo, R. M. K., Noté, O. P., Mahiou-Leddet, V., Herbette, G., Fekam, F.B., Ollivier, E. & Folefoc, G. N. (2014). Secondary metabolites from *Jacaranda mimosifolia* and *Kigelia africana* (Bignoniaceae) and their anticandidal activity. *Records of Natural Products*, *8*(3), 307-311.

Sikarwar, M. S., & Patil, M. B. (2010). Antidiabetic activity of *Pongamia pinnata* leaf extracts in alloxan-induced diabetic rats. *International journal of Ayurveda research*, *1*(4), 199.

Singh, A. (2007). *Didymocarpus pedicellata*: the lithontriptic ethnomedicine. *Ethnobotanical Leaflets*, *11*, 73-75.

Singh, A. P. (2005). Promising phytochemicals from Indian medicinal plants.*Ethnobotanical Leaflets9*, 15- 23.

Singh, A., & Duggal, S. (2009). Medicinal orchids-an overview. *Ethnobotanical leaflets*, *13*, 399-412.

Singh, D. V., Prajapati, S., Bajpai, S., Verma, R. K., Gupta, M. M., & Kumar, S. (2000). Simultaneous determination of important alkaloids in *Papaver somniferum* using reversed phase high performance liquid chromatography.*J. Liq. Chrom. & Rel. Technol*., *23*(11), 1757–1764.

Singh, G., Singh, O. P., & Maurya, S. (2002). Chemical and biocidal investigations on essential oils of some Indian *Curcuma* species. *Progress in Crystal Growth and Characterization of Materials*, *45*(1-2), 75-81.

Singh, G., Singh, O. P., Prasad, Y. R., De Lampasona, M. P., & Catalan, C. (2002). Studies on essential oils, Part 33: chemical and insecticidal investigations on leaf oil of *Coleus amboinicus* Lour. *Flavour and fragrance journal*, *17*(6), 440-442.

Singh, H. P., Mittal, S., Kaur, S., Batish, D. R., & Kohli, R. K. (2009). Characterization and antioxidant activity of essential oils from fresh and decaying leaves of *Eucalyptus tereticornis*. *Journal of agricultural and food chemistry*, *57*(15), 6962-6966.

Singh, K., Rup, P. J., Saxena, A. K., Khan, R. H., Ashraf, M. T., Kamboj, S. S., & Singh, J. (2006). A tuber lectin from *Arisaema helleborifolium* Schott with anti-insect activity against melon fruit fly, *Bactrocera cucurbitae* (Coquillett) and anti-cancer effect on human cancer cell lines. *Archives of Biochemistry and Biophysics*, *445*(1), 156-165.

Singh, P., Singh, R., Sati, N., Ahluwalia, V., & Sati, O. P. (2017). Phytochemical and Pharmacological Significance of Genus: *Impatiens*. *Int. J. Life. Sci. Scienti. Res*, *3*(1), 868-881.

Singh, P., Singh, R., Sati, N., Sati, O. P., & Kumar, N. (2015). A Review of Genus: *Jurinea*. *Int. J. Life. Sci. Scienti. Res*, *2*(1).

Singh, T. P., & Singh, O. M. (2011). Phytochemical and pharmacological profile of *Zanthoxylum armatum* DC.-an overview. Indian Journal of Natural Products and Resources, *2*(3), 275-285.

Singha, A. S., & Rana, A. K. (2012). Effect of surface modification of *Grewia optiva* fibres on their physicochemical and thermal properties. *Bulletin of Materials Science*, *35*(7), 1099-1110.

Singhatong, S., Leelarungrayub, D., & Chaiyasut, C. (2010). Antioxidant and toxicity activities of *Artocarpus lakoocha* Roxb. heartwood extract. *Journal of Medicinal Plants Research*, *4*(10), 947-953.

Sinha, S. C., Sahai, M. & Ray, A. B. (1986). Chemical constituents of *Rhus wallichi*. *Journal of Natural Products*, *49*(3), 546-546.

Skaltsa, H. (2003). Chemical constituents. In *Fenugreek* (pp. 148-177). CRC Press.

Soni, P., Siddiqui, A. A., Dwivedi, J., & Soni, V. (2012). Pharmacological properties of *Datura stramonium* L. as a potential medicinal tree: an overview. *Asian Pacific journal of tropical biomedicine*, *2*(12), 1002-1008.

Sovrlić, M., Vasiljević, P., Jušković, M., Mašković, P., & Manojlović, N. (2015). Phytochemical, antioxidant and antimicrobial profiles of extracts of *Daphne alpina* (Thymelaeaceae) L leaf and twig from Mt Kopaonik (Serbia). *Tropical Journal of Pharmaceutical Research*, *14*(7), 1239-1248.

Sowjanya, K. M., Narendra, K., Swathi, J., & Satya, K. (2013). Phytochemical extraction and antimicrobial efficiency of crude leaf extract of medicinal plant, *Cascabela thevetia*. *International Journal of Research in Pharmacy and Biomedical Science*, *4*(2), 465-470.

Sowndhararajan, K., & Kang, S. C. 2013. Free radical scavenging activity from different extracts of leaves of *Bauhinia vahlii* Wight & Arn. *Saudi journal of biological sciences*, *20*(4), 319-325.

Sreejith, P. S., Praseeja, R. J., & Asha, V. V. (2012). A review on the pharmacology and phytochemistry of traditional medicinal plant, *Glycosmis pentaphylla* (Retz.) Correa. *Journal of Pharmacy Research*, *55*(55), 2723-2728.

Srikanth, M., Devi, B., Kotirataiah, K., Ramanjaneyulu, M, Sulthana, P.N, Suma, R. R. (2018). Phytochemical screening and in-vitro antioxidant activity of Peristrophe paniculata. *Herb Med*. 4(1:1. doi: 10.21767/2472-0151.100033

Srinivasan, G. V., Unnikrishnan, K. P., Shree, A. R., & Balachandran, I. (2008). HPLC estimation of berberine in *Tinospora cordifolia* and *Tinospora sinensis*. *Indian journal of pharmaceutical sciences*, *70*(1), 96-99.

Sriset, Y., Jarukamjorn, K., & Chatuphonprasert, W. (2017). Pharmacological Activities of *Cryptolepis dubia* (Burm. f.) MR Almeida. *Isan Journal of Pharmaceutical Sciences, 13*(1), 1-10.

Srivastav, S., Singh, P., Mishra, G., Jha, K. K. & Khosa, R. L. (2011). *Achyranthes aspera*- An important medicinal plant: A review. *J Nat Prod Plant Resour,1*(1):1-14.

Srivastava, S. K., Singh Rawat, A. K., & Mehrotra, S. (2004). Pharmacognostic evaluation of the root of *Berberis asiatica*. *Pharmaceutical biology*, *42*(6), 467-473.

Srivastava, S., Misra, A., Kumar, D., Srivastava, A., Sood, A., & Rawat, A. K. S. (2015). Reversed-phase high-performance liquid chromatography-ultraviolet photodiode array detector validated simultaneous quantification of six bioactive phenolic acids in *Roscoea purpurea* tubers and their In vitro cytotoxic potential against various cell lines. *Pharmacognosy magazine*, *11,* 488-495.

Štajner, D., Popović, B. M., Čanadanović‐Brunet, J., & Anačkov, G. (2009). Exploring *Equisetum arvense* L., *Equisetum ramosissimum* L. and *Equisetum telmateia* L. as sources of natural antioxidants. *Phytotherapy research*, *23*(4), 546-550.

Stefanović, O. D., Tešić, J. D., & Čomić, L. R. (2015). *Melilotus albus* and *Dorycnium herbaceum* extracts as source of phenolic compounds and their antimicrobial, antibiofilm, and antioxidant potentials. *journal of food and drug analysis*, *23*(3), 417-424.

Stewart, A. V. (1996). Plantain (*Plantago lanceolata*)-a potential pasture species. In *Proceedings of the Conference-New Zealand Grassland Association* (pp. 77-86).

Strauss, A., Spengel, S. M., & Schaffner, W. (1995). Saponins from root cultures of *Phytolacca acinosa*. *Phytochemistry*, *38*(4), 861-865.

Subba Rao, M. V. S. S. T., & Muralikrishna, G. (2002). Evaluation of the antioxidant properties of free and bound phenolic acids from native and malted finger millet (Ragi, *Eleusine coracana* Indaf-15). *Journal of agricultural and food chemistry*, *50*(4), 889-892.

Suchaichit, N., Kanokmedhakul, S., Kanokmedhakul, K., Moosophon, P., Boonyarat, C., Plekratoke, K., Tearavarich, R., & Suchaichit, N. P. (2017). Phytochemical investigation and acetylcholinesterase inhibitory activity of bark of *Hymenodictyon orixense*. *Natural product research*, 1-4.

Sudan, R., Bhagat, M., Gupta, S., Singh, J., & Koul, A. (2014). Iron (FeII) chelation, ferric reducing antioxidant power, and immune modulating potential of *Arisaema jacquemontii* (Himalayan Cobra Lily). *BioMed research international*, *2014*.

Sultana, S., Nandi, J.K., Rahman, S., Jahan, R. & Rahmatullah, M. (2014). Preliminary antihyperglycemic and analgesic activity studies with *Angiopteris evecta* leaves in swiss albino mice. *World Journal of Pharmacy and Pharmaceutical Sciences*, *3*(10), 1-12.

Surendran, S., Eswaran, M. B., Vijayakumar, M., & Rao, C. V. (2011). In vitro and in vivo hepatoprotective activity of Cissampelos pareira against carbon-tetrachloride induced hepatic damage.

Suresh, K. (2008). Antimicrobial and Phytochemical Investigation of the Leaves of *Carica papaya* L., *Cynodon dactylon* (L.) Pers., *Euphorbia hirta* L., *Melia azedarach* L. and Psidium guajava L. *Ethnobotanical Leaflets*, *12*, 1184-1191.

Suriyavathana, M., Parameswari, G., & Shiyan, S. P. (2012). Biochemical and antimicrobial study of *Boerhavia erecta* and *Chromolaena odorata* (L.) King & Robinson. *International Journal of Pharmaceutical Sciences and Research*, *3*(2), 465.

Syrchina, A. I., & Semenov, A. A. (1982). Natural indanones. *Chemistry of Natural Compounds*, *18*(1), 1-11.

Syu, W. J., Don, M. J., Lee, G. H., & Sun, C. M. (2001). Cytotoxic and novel compounds from *Solanum indicum*. *Journal of natural products*, *64*(9), 1232-1233.

Tabanca, N., Ma, G., Pasco, D. S., Bedir, E., Kirimer, N., Baser, K., Khan, I. A. & Khan, S. I. (2007). Effect of essential oils and isolated compounds from Pimpinella species on NF‐κB: a target for antiinflammatory therapy. *Phytotherapy research*, *21*(8), 741-745.

Talagari, M. & Hullatti, K. (2015). In-vitro α- amylase and α- glucosidase inhibitory activity of *Adiantum caudatum* Linn. And *Celosia argentea* Linn. Extracts and fractions. *Indian Journal of Pharmacology*, *47*(4), 425.

Tamilarasan, M., Sivamani, S., Maheshwari, A. S., & Rajesh, T. P. (2015). Phytochemical screening and in vitro bioactivities of the extracts of aerial part of *Evolvulus nummularius*.*International Journal of Pharmacognosy and Phytochemical Research*,*7*(1), 111-116.

Tampieri, M. P., Galuppi, R., Macchioni, F., Carelle, M. S., Falcioni, L., Cioni, P. L., & Morelli, I. (2005). The inhibition of *Candida albicans* by selected essential oils and their major components. *Mycopathologia*, *159*(3), 339-345.

Tanaka, M., Misawa, E., Ito, Y., Habara, N., Nomaguchi, K., Yamada, M., Toida, T., Hayasawa, H., Takase, M., Inagaki, M., & Higuchi, R. (2006). Identification of five phytosterols from *Aloe vera* gel as anti-diabetic compounds. *Biological and Pharmaceutical Bulletin*, *29*(7), 1418-1422.

Tanaka, N., Yano, Y., Tatano, Y., & Kashiwada, Y. (2016). Hypatulins A and B, Meroterpenes from *Hypericum patulum*. *Organic letters*, *18*(20), 5360-5363.

Tandon, M., Shukla, Y. N., Tripathi, A. K., & Singh, S. C. (1998). Insect antifeedant principles from *Vernonia cinerea*. *Phytotherapy Research: An International Journal Devoted to Pharmacological and Toxicological Evaluation of Natural Product Derivatives*, *12*(3), 195-199.

Tang, Z., Zhou, Y., Zeng, Y., Zang, S., He, P., & Fang, Y. (2006). Capillary electrophoresis of the active ingredients of *Dioscorea bulbifera* L. and its medicinal preparations. *Chromatographia*, *63*(11-12), 617-622.

Tanruean, K., Poolprasert, P., Kumla, J., Suwannarach, N., & Lumyong, S. (2017). Bioactive compounds content and their biological properties of acetone extract of *Cuscuta reflexa* Roxb. grown on various host plants. *Natural product research*, 1-4.

Tava, A., Pecetti, L., Romani, M., Mella, M., & Avato, P. (2011). Triterpenoid glycosides from the leaves of two cultivars of *Medicago polymorpha* L. *Journal of agricultural and food chemistry*, *59*(11), 6142-6149.

Teng, B. S., Lu, Y. H., Wang, Z. T., Tao, X. Y., & Wei, D. Z. (2006). In vitro anti-tumor activity of isorhamnetin isolated from *Hippophae rhamnoides* L. against BEL-7402 cells. *Pharmacological research*, *54*(3), 186-194.

Thatoi, H. N., & Dutta, S. K. (2009). Antibacterial activity and phytochemical screening of leaf and bark extracts of *Vitex negundo* l. from similipal biosphere reserve, Orissa. *Journal of medicinal plants research*, *3*(4), 294-300.

Thirupathi, K., Kumar, S. S., Raju, V. S., Ravikumar, B., Krishna, D. R., & Mohan, G. K. (2008). A review of medicinal plants of the genus *Cordia:* Their chemistry and pharmacological uses. *Journal of Natural Remedies*, *8*(1), 1-10.

Thooyavan, G., & Karthikeyan, J. (2016). Phytochemical profiling and GC-MS analysis of *Butea monosperma* seed methanol extract. *Journal of Pharmacognosy and Phytochemistry*, *5*(5), 152-157.

Tilak, J. C., Adhikari, S., & Devasagayam, T. P. (2004). Antioxidant properties of *Plumbago zeylanica*, an Indian medicinal plant and its active ingredient, plumbagin. *Redox report*, *9*(4), 219-227.

Tiwari, K. L., Jadhav, S. K., & Joshi, V. (2011). An updated review on medicinal herb genus *Spilanthes*. *J Chin Integr Med*, *9*(11), 1170-1178.

Tomczyk, M., & Latté, K. P. (2009). Potentilla—A review of its phytochemical and pharmacological profile. *Journal of Ethnopharmacology*, *122*(2), 184-204.

Tomczyk, M., Pleszczyńska, M., & Wiater, A. (2010). Variation in total polyphenolics contents of aerial parts of *Potentilla* species and their anticariogenic activity. *Molecules*, *15*(7), 4639-4651.

Tona, L., K. Kambu, N. Ngimbi, K. Cimanga, and A. J. Vlietinck. (1998). Antiamoebic and phytochemical screening of some Congolese medicinal plants. *Journal of Ethnopharmacology, 61*(1), 57-65.

Tripathee, H. P., Sharma, R. P., Timilsina, Y. P., Pathak, R., & Devkota, K. P. (2012). An assessment of ethnomedicinal use, chemical constituents analysis and bioactivity evaluation on high altitude medicinal plant *Delphinium brunonianum* of Manang district. *Nepal Journal of Science and Technology*, *12*, 111-118.

Trivedi, A., Neeraj Sethiya, K., & Mishra, S. H. (2011). Preliminary pharmacognostic and phytochemical analysis of “Granthika”(*Leonotis nepetaefolia*): An Ayurvedic herb. *Indian Journal of Traditional Knowledge*, *10*(4), 682-688.

Trusheva, B., Todorov, I., Ninova, M., Najdenski, H., Daneshmand, A., & Bankova, V. (2010). Antibacterial mono-and sesquiterpene esters of benzoic acids from Iranian propolis. *Chemistry Central Journal*, *4*(1), 8.

Tsai, J. C., Huang, G. J., Chiu, T. H., Huang, S. S., Huang, S. C., Huang, T. H., ... & Lee, C. Y. (2011). Antioxidant activities of phenolic components from various plants of *Desmodium* species. *African Journal of Pharmacy and Pharmacology*, *5*(4), 468-476.

Tu, Y., Sun, L., Guo, M., & Chen, W. (2013). The medicinal uses of *Callicarpa* L. in traditional Chinese medicine: An ethnopharmacological, phytochemical and pharmacological review. *Journal of ethnopharmacology*, *146*(2), 465-481.

Turker, A. U., & Gurel, E. (2005). Common mullein (*Verbascum thapsus* L.): recent advances in research. *Phytotherapy Research*, *19*(9), 733-739.

Uddin, G., Rauf, A., Arfan, M., Ali, M., Qaisar, M., Saadiq, M., & Atif, M. (2012). Preliminary phytochemical screening and antioxidant activity of *Bergenia caliata*. *Middle-East J. Sci. Res*, *11*(8), 1140-1142.

Umachigi, S. P., Kumar, G. S., Jayaveera, K. N., & Dhanapal, R. (2007). Antimicrobial, wound healing and antioxidant activities of *Anthocephalus cadamba*. *African journal of traditional, complementary and alternative medicines*, *4*(4), 481-487.

Vaghasiya, Y., Dave, R., & Chanda, S. (2011). Phytochemical analysis of some medicinal plants from western region of India. *Res J Med Plant*, *5*(5), 567-576.

Vale, T. G., Matos, F. J. A., De Lima, T. C. M., & Viana, G. S. B. (1999). Behavioral effects of essential oils from *Lippia alba* (Mill.) NE Brown chemotypes. *Journal of Ethnopharmacology*, *67*(2), 127-133.

Valsaraj, R., Pushpangadan, P., Smitt, U. W., Adsersen, A., Christensen, S. B., Sittie, A., Nyman, U., Nielsen, C.  & Olsen, C. E. (1997). New anti-HIV-1, antimalarial, and antifungal compounds from *Terminalia bellerica*. *Journal of natural products*, *60*(7), 739-742.

Vanisree, M., Lee, C. Y., Lo, S. F., Nalawade, S. M., Lin, C. Y., & Tsay, H. S. (2004). Studies on the production of some important secondary metabolites from medicinal plants by plant tissue cultures. *Bot. Bull. Acad. Sin*, *45*(1), 1-22.

Vasas, A., & Hohmann, J. (2014). *Euphorbia* diterpenes: isolation, structure, biological activity, and synthesis (2008–2012). *Chemical reviews*, *114*(17), 8579-8612.

Vasconcelos, C. M. L. D., Araujo, M. S. D., Silva, B. A. D., & Conde-Garcia, E. A. (2005). Negative inotropic and chronotropic effects on the guinea pig atrium of extracts obtained from *Averrhoa carambola* L. leaves. *Brazilian journal of medical and biological research*, *38*(7), 1113-1122.

Velanganni, J., Kadamban, D., & Tangavelou, A. C. (2011). Phytochemical screening and antimicrobial activity of the stem of *Mallotus philippensis* (Lam.) Muell. Arg. Var. Philippensis (Euphorbiaceae). *International Journal of Pharmacology and Pharmaceutical Sciences*, *3*(2), 160-163.

Velappan, S., & Thangaraj, P. (2014). Phytochemical constituents and antiarthritic activity of *Ehretia laevis* roxb. *Journal of food biochemistry*, *38*(4), 433-443.

Velmurugan, C., Sundaram, T., Sampath Kumar, R., Vivek, B., SheshadriShekar, D., & Ashok Kumar, B. S. (2011). Anti diabetic and hypolipidemic activity of bark of ethanolic extract of *Ougeinia oojeinensis* (ROXB.). *Med J Malaysia*, *66*(1), 23-26.

Verma, D. K., Khan, F., Verma, C., & SonalikaAgrawal, M. A. (2017). Stem extract of *Opuntia cochenillifera* as green and sustainable corrosion inhibitor of mild steel in 0.5 MH2so4 solution. *Int J Nano Corr Sci and Engg*, *4*(1), 31-54.

Verma, P. C., Basu, V., Gupta, V., Saxena, G., & Ur Rahman, L. (2009). Pharmacology and chemistry of a potent hepatoprotective compound Picroliv isolated from the roots and rhizomes of *Picrorhiza kurroa* royle ex benth.(kutki). *Current pharmaceutical biotechnology*, *10*(6), 641-649.

Verma, R. K., Singh, A. K., Srivastava, P., Shanker, K., Kalra, A., & Gupta, M. M. (2009). Determination of novel plant growth promoting diterpenes in *Callicarpa macrophylla* by HPLC and HPTLC. *Journal of Liquid Chromatography & Related Technologies*, *32*(16), 2437-2450.

Verma, R. S., Padalia, R. C., & Chauhan, A. (2014). Leaf essential oil composition of *Inula cuspidata* (Wall. ex DC.) CB Clarke from India. *Journal of Essential Oil Research*, *26*(4), 233-237.

Vhuiyan, M. M. I., Biva, I. J., Saha, M. R., & Islam, M. S. (2008). Anti-diarrhoeal and CNS Depressant Activity of Methanolic Extract of *Saccharum spontaneum* Linn. *Stamford Journal of Pharmaceutical Sciences*, *1*(1), 63-68.

Viji, M., & Murugesan, S. 2010. Phytochemical Analysis and Antibacterial Activity of Medicinal Plant Cardiospermum halicacabum Linn. *Journal of Phytology*, *2*(1), 445-456.

Vikrant, A., & Arya, M. L. (2011). A review on anti-inflammatory plant barks. *International Journal of PharmTech Research*, *3*(2), 899-908.

Vohra, A., & Kaur, H. (2011). Chemical investigation of medicinal plant *Ajuga bracteosa*. *J Nat Prod Plant Resour*, *1*(1), 37-45.

Voon, H. C., Bhat, R., & Rusul, G. (2012). Flower extracts and their essential oils as potential antimicrobial agents for food uses and pharmaceutical applications. *Comprehensive Reviews in Food Science and Food Safety*, *11*(1), 34-55.

Voronkova, m., & Vysochina, G. (2014). *Bistorta* Scop. Genus (Polygonaceae): Chemical Composition and Biological Activity.*Chemistry for Sustainable Development22*, 207-212.

Vysochina, G. I., Kukushkina, T. A., Kotsupii, O. V., Zagurskaya, Y. V., & Bayandina, I. I. (2011). Flora of the forest-steppe zone of West Siberia as a source of biologically active compounds. *Contemporary Problems of Ecology*, *4*(2), 273-284.

Wagner, H., Geyer, B., Kiso, Y., Hikino, H., & Rao, G. S. (1986). Coumestans as the main active principles of the liver drugs *Eclipta alba* and *Wedelia calendulacea1*. *Planta Medica*, *52*(5), 370-374.

Wang, H., Zou, H., Ni, J., Kong, L., Gao, S., & Guo, B. (2000). Fractionation and analysis of *Artemisia capillaris* Thunb. by affinity chromatography with human serum albumin as stationary phase. *Journal of Chromatography A*, *870*(1-2), 501-510.

Wang, K. J., Zhang, Y. J., & Yang, C. R. (2005). Antioxidant phenolic constituents from *Fagopyrum dibotrys*. *Journal of ethnopharmacology*, *99*(2), 259-264.

Wang, L., Wang, Z., & Li, X. (2013). Preliminary phytochemical and biological activities study of solvent extracts from a cold-field fruit—*Malus baccata* (Linn.) Borkh. *Industrial crops and products*, *47*, 20-28.

Wang, X., Fang, X., Gan, H., Jiang, W., & Wu, M. (2016). Electrochemical Determination of Ternatin in Ternate Grape Fern Herb Based on the Graphene-Au Nanocomposite. *International Journal of Electrochemical Science*, *11*(11), 9369-9378.

Wang, Y., Chen, S. N., Pan, Y., Zhang, J., & Chen, Y. (1996). Diterpenoid alkaloids from *Delphinium caeruleum*. *Phytochemistry*, *42*(2), 569-571.

Wangchuk, P., Keller, P. A., Pyne, S. G., Taweechotipatr, M., Tonsomboon, A., Rattanajak, R., & Kamchonwongpaisan, S. (2011). Evaluation of an ethnopharmacologically selected Bhutanese medicinal plants for their major classes of phytochemicals and biological activities. *Journal of ethnopharmacology*, *137*(1), 730-742.

Wani, S. H., Amin, A., Rather, M. A., Parray, J., Parvaiz, A., & Qadri, R. A. (2012). Antibacterial and phytochemical screening of different extracts of five Iris species growing in Kashmir. *J. of Pharm. Res*, *5*(6), 3376-3378.

Wen, L., Wu, D., Jiang, Y., Prasad, K. N., Lin, S., Jiang, G., He, J., Zhao, M., Luo, W., & Yang, B. (2014). Identification of flavonoids in litchi (*Litchi chinensis* Sonn.) leaf and evaluation of anticancer activities. *Journal of functional foods*, *6,* 555-563.

Weng, X. C., & Wang, W. (2000). Antioxidant activity of compounds isolated from *Salvia plebeia*. *Food Chemistry*, *71*(4), 489-493.

Westenburg, H. E., Lee, K. J., Lee, S. K., Fong, H. H., Van Breemen, R. B., Pezzuto, J. M. & Kinghorn, A. D. (2000). Activity- guided isolation of antioxidative constituents of *Cotinus coggygria*. *Journal of Natural Products*, *63*(12), 1696-1698.

Williams, C. A., Goldstone, F., & Greenham, J. (1996). Flavonoids, cinnamic acids and coumarins from the different tissues and medicinal preparations of *Taraxacum officinale*. *Phytochemistry*, *42*(1), 121-127.

Wong, J. Y., Matanjun, P., Ooi, Y. B. H., & Chia, K. F. (2014). Evaluation of antioxidant activities in relation to total phenolics and flavonoids content of selected Malaysian wild edible plants by multivariate analysis. *International journal of food properties*, *17*(8), 1763-1778.

Wu, C. H., & Yen, G. C. (2004). Antigenotoxic properties of Cassia tea (Cassia tora L.): Mechanism of action and the influence of roasting process. *Life sciences*, *76*(1), 85-101.

Wu, F., Bian, D., Xia, Y., Gong, Z., Tan, Q., Chen, J., & Dai, Y. (2012). Identification of major active ingredients responsible for burn wound healing of *Centella asiatica* herbs. *Evidence-Based Complementary and Alternative Medicine*.

Wu, H., Haig, T., Pratley, J., Lemerle, D., & An, M. (2000). Distribution and exudation of allelochemicals in wheat *Triticum aestivum*. *Journal of Chemical Ecology*, *26*(9), 2141-2154.

Wu, J. B., Chun, Y. T., Ebizuka, Y., & Sankawa, U. (1991). Biologically active constituents of *Centipeda minima*: Sesquiterpenes of potential anti-allergy activity. *Chemical and pharmaceutical bulletin*, *39*(12), 3272-3275.

Wu, K. C., Huang, S. S., Kuo, Y. H., Ho, Y. L., Yang, C. S., Chang, Y. S., & Huang, G. J. (2017). Ugonin M, a *Helminthostachys zeylanica* constituent, prevents LPS-induced acute lung injury through TLR4-mediated MAPK and NF-κB signaling pathways. *Molecules*, *22*(4), 573.

Wu, Q. F., Wang, W., Dai, X. Y., Wang, Z. Y., Shen, Z. H., Ying, H. Z., & Yu, C. H. (2012). Chemical compositions and anti-influenza activities of essential oils from *Mosla dianthera*. *Journal of ethnopharmacology*, *139*(2), 668-671.

Wu, Z. Y., Li, H. Z., Wang, W. G., Li, H. M., Chen, R., Li, R. T., & Luo, H. R. (2011). Lyonin A, a New 9, 10‐Secograyanotoxin from *Lyonia ovalifolia*. *Chemistry & biodiversity*, *8*(6), 1182-1187.

Xia, L., Guo, Q., Tu, P., & Chai, X. (2015). The genus *Casearia*: a phytochemical and pharmacological overview. *Phytochemistry reviews*, *14*(1), 99-135.

Xie, H. G., Chen, H., Cao, B., Zhang, H. W., & Zou, Z. M. (2007). Cytotoxic germacranolide sesquiterpene from Inula cappa. *Chemical and Pharmaceutical Bulletin*, *55*(8), 1258-1260.

Xu, J., Guo, Y., Xie, C., Li, Y., Gao, J., Zhang, T., Hou, W., Fang, L. & Gui, L. (2011). Bioactive myrsinol diterpenoids from the roots of *Euphorbia prolifera*. *Journal of natural products*, *74*(10), 2224-2230.

Yadav, Y., Mohanty, P. K., & Kasture, S. B. (2011). Anti-inflammatory activity of hydroalcoholic extract of *Quisqualis indica* Linn. flower in rats. *International Journal of Pharmacy & Life Sciences*, *2*(8), 977-981.

Yamaguchi, H., Matsuura, H., Kasai, R., Mizutani, K., Fujino, H., Ohtani, K., Fuwa, T., & Tanaka, O. (1986). Application of borate ion-exchange mode high-performance liquid chromatography to separation of glycosides: *Saponins of ginseng*, *Sapindus mukurossi* Gaertn. and *Anemone rivularis* Buch.-Ham. *Chemical and pharmaceutical bulletin*, *34*(7), 2859-2867.

Yan, L. L., Zhang, Y. J., Gao, W. Y., Man, S. L., & Wang, Y. (2009). In vitro and in vivo anticancer activity of steroid saponins of *Paris polyphylla* var. yunnanensis. *Exp Oncol*, *31*(1), 27-32.

Yao, X. S., Ebizuka, Y., Noguchi, H., Kiuchi, F., Shibuya, M., Iitaka, Y., Seto, H. & Sankawa, U. (1991). Biologically active constituents of *Arnebia euchroma*: structure of arnebinol, an ansa-type monoterpenylbenzenoid with inhibitory activity on prostaglandin biosynthesis. *Chemical and pharmaceutical bulletin*, *39*(11), 2956-2961.

Yatoo, M. I., Dimri, U., Gopalakrishnan, A., Karthik, K., Gopi, M., Khandia, R., Saminathan, M., Saxena, A., Alagawany, M., Farag, M. R., & Munjal, A. (2017). Beneficial health applications and medicinal values of *Pedicularis* plants: A review. *Biomedicine & Pharmacotherapy*, *95*, 1301-1313.

Yokozawa, T., Kim, H. Y., Cho, E. J., Choi, J. S., & Chung, H. Y. (2002). Antioxidant effects of isorhamnetin 3, 7-di-O-β-D-glucopyranoside isolated from mustard leaf (*Brassica juncea*) in rats with streptozotocin-induced diabetes. *Journal of agricultural and food chemistry*, *50*(19), 5490-5495.

Yousuf, S., Bachheti, R. K., Joshi, A., Mathur, A. (2014). Evaluation of antioxidant potential and phytochemicals of *Morina longifolia*. *Int J Pharm Pharm Sci*, *6*(6), 208-212.

Yunusov, M. S. (1991). Diterpenoid alkaloids. *Natural Product Reports*, *8*(5), 499-526.

Zahan, R., Nahar, L., Mosaddik, A., Rashid, M. A., Hassan, A., & Ahmed, M. (2013). Evaluation of antioxidant and antitumor activities of Wrightia arborea. *Journal of Basic & Applied Sciences*, 9, 625.

Zahin, M., Ahmad, I., & Aqil, F. (2010). Antioxidant and antimutagenic activity of *Carum copticum* fruit extracts. *Toxicology in Vitro*, *24*(4), 1243-1249.

Zaini, N. A. M., Anwar, F., Hamid, A. A., & Saari, N. (2011). Kundur [*Benincasa hispida* (Thunb.) Cogn.]: A potential source for valuable nutrients and functional foods. *Food Research International*, *44*(7), 2368-2376.

Zakaria, Z. A., Ghani, Z. , Nor, R., Gopalan, H. K., Sulaiman, M. R., & Abdullah, F. C. (2006). Antinociceptive and anti-inflammatory activities of *Dicranopteris linearis* leaves chloroform extract in experimental animals. *Yakugaku Zasshi*, *126*(11), 1197-1203.

Zandi, P., Basu, S. K., Cetzal-Ix, W., Kordrostami, M., Chalaras, S. K., & Khatibai, L. B. (2017). Fenugreek (*Trigonella foenum-graecum* L.): An Important Medicinal and Aromatic Crop. In *Active Ingredients from Aromatic and Medicinal Plants*. InTech.

Zaveri, M., Khandhar, A., Patel, S., & Patel, A. (2010). Chemistry and pharmacology of *Piper longum* L. *International journal of pharmaceutical sciences review and research*, *5*(1), 67-76.

zda Vrchotováa, N., Seráa, B., & Dadákováb, E. (2008). Some phenolic compounds in Himalayan Knotweed. *J. Indian Chem. Soc*, *85*, 1-2.

Zeng, W. C., Zhang, Z., Gao, H., Jia, L. R., & He, Q. (2012). Chemical composition, antioxidant, and antimicrobial activities of essential oil from pine needle (*Cedrus deodara*). *Journal of food science*, *77*(7).

Zhang, H. J., Hu, Y. J., Xu, P., Liang, W. Q., Zhou, J., Liu, P. G., ... & Pu, J. B. (2016). Screening of Potential Xanthine Oxidase Inhibitors in *Gnaphalium hypoleucum* DC. by Immobilized Metal Affinity Chromatography and Ultrafiltration-Ultra Performance Liquid Chromatography-Mass Spectrometry. *Molecules*, *21*(9), 1242.

Zhang, J. J., Yang, X. W., Liu, X., Ma, J. Z., Liao, Y., & Xu, G. (2015). 1, 9-seco-bicyclic polyprenylated acylphloroglucinols from *hypericum uralum*. *Journal of natural products*, *78*(12), 3075-3079.

Zhang, W., Yao, M. N., Tang, H. F., Tian, X. R., Wang, M. C., Ji, L. J., & Xi, M. M. (2013). Triterpenoid saponins with anti-myocardial ischemia activity from the whole plants of *Clematis tangutica*. *Planta medica*, *79*(08), 673-679.

Zhang, Y. J., Nagao, T., Tanaka, T., Yang, C. R., Okabe, H., & Kouno, I. (2004). Antiproliferative activity of the main constituents from *Phyllanthus emblica*. *Biological and Pharmaceutical Bulletin*, *27*(2), 251-255.

Zhang, Z., Liao, L., Moore, J., Wu, T., & Wang, Z. (2009). Antioxidant phenolic compounds from walnut kernels (*Juglans regia* L.). *Food chemistry*, *113*(1), 160-165.

Zhao, X., Zheng, G. W., Niu, X. M., Li, W. Q., Wang, F. S., & Li, S. H. (2008). Terpenes from *Eupatorium adenophorum* and their allelopathic effects on Arabidopsis seeds germination. *Journal of agricultural and food chemistry*, *57*(2), 478-482.

Zhao, Y. L., He, Q. X., Li, Y., Wang, S. F., Liu, K. C., Yang, Y. P., & Li, X. L. (2010). Chemical constituents of Excoecaria acerifolia and their bioactivities. *Molecules*, *15*(4), 2178-2186.

Zheng, X., Wang, W., Piao, H., Xu, W., Shi, H., & Zhao, C. (2013). The genus *Gnaphalium* L.(Compositae): phytochemical and pharmacological characteristics. *Molecules*, *18*(7), 8298-8318.

Zhou, J. M., & Ibrahim, R. K. (2010). Tricin—a potential multifunctional nutraceutical. *Phytochemistry Reviews*, *9*(3), 413-424.

Zhu, M., Dong, X., & Guo, M. (2015). Phenolic profiling of Duchesnea indica combining macroporous resin chromatography (MRC) with HPLC-ESI-MS/MS and ESI-IT-MS. *Molecules*, *20*(12), 22463-22475.

Zingue, S., Tchoumtchoua, J., Ntsa, D. M., Sandjo, L. P., Cisilotto, J., Nde, C. B. M., Winter, E., Awounfack, C. F., Ndinteh, D. T., Clyne, C., & Njamen, D. 2016. Estrogenic and cytotoxic potentials of compounds isolated from *Millettia macrophylla* Benth (Fabaceae): towards a better understanding of its underlying mechanisms. *BMC complementary and alternative medicine*, *16*(1), 421.

Zulfiker, A. H. M., Rahman, M. M., Hossain, M. K., Hamid, K., Mazumder, M. E. H., & Rana, M. S. 2010. In vivo analgesic activity of ethanolic extracts of two medicinal plants-*Scoparia dulcis* L. and *Ficus racemosa* Linn. *Biol Med*, *2*(2), 42-48.
